# Composition and Diversity of the Fecal Microbiome and Inferred Fecal Metagenome Does Not Predict Subsequent Pneumonia Caused by *Rhodococcus equi* in Foals

**DOI:** 10.1371/journal.pone.0136586

**Published:** 2015-08-25

**Authors:** Canaan M. Whitfield-Cargile, Noah D. Cohen, Jan Suchodolski, M. Keith Chaffin, Cole M. McQueen, Carolyn E. Arnold, Scot E. Dowd, Glenn P. Blodgett

**Affiliations:** 1 Department of Large Animal Clinical Sciences, College of Veterinary Medicine & Biomedical Sciences, Texas A&M University, College Station, Texas, United States of America; 2 Department of Small Animal Clinical Sciences, College of Veterinary Medicine & Biomedical Sciences, Texas A&M University, College Station, Texas, United States of America; 3 Molecular Research DNA Laboratory (MR DNA), Shallowater, Texas, United States of America; 4 6666 Ranch, Guthrie, Texas, United States of America; The University of Melbourne, AUSTRALIA

## Abstract

In equids, susceptibility to disease caused by *Rhodococcus equi* occurs almost exclusively in foals. This distribution might be attributable to the age-dependent maturation of immunity following birth undergone by mammalian neonates that renders them especially susceptible to infectious diseases. Expansion and diversification of the neonatal microbiome contribute to development of immunity in the gut. Moreover, diminished diversity of the gastrointestinal microbiome has been associated with risk of infections and immune dysregulation. We thus hypothesized that varying composition or reduced diversity of the intestinal microbiome of neonatal foals would contribute to increased susceptibility of their developing *R*. *equi* pneumonia. The composition and diversity indices of the fecal microbiota at 3 and 5 weeks of age were compared among 3 groups of foals: 1) foals that *subsequently* developed *R*. *equi* pneumonia after sampling; 2) foals that *subsequently* developed ultrasonographic evidence of pulmonary abscess formation or consolidation but *not* clinical signs (subclinical group); and, 3) foals that developed neither clinical signs nor ultrasonographic evidence of pulmonary abscess formation or consolidation. No significant differences were found among groups at either sampling time, indicating absence of evidence of an influence of composition or diversity of the fecal microbiome, or predicted fecal metagenome, on susceptibility to subsequent *R*. *equi* pneumonia. A marked and significant difference identified between a relatively short interval of time appeared to reflect ongoing adaptation to transition from a milk diet to a diet including available forage (including hay) and access to concentrate fed to the mare.

## Introduction

Pneumonia is a major cause of disease and death in foals, and *Rhodococcus equi* is considered the most important cause of pneumonia among foals between approximately 1 and 6 months of age [1–3]. Susceptibility to *R*. *equi* pneumonia in horses appears to be age-related: the disease occurs almost exclusively among foals, whereas adult horses or other species of animals generally do not develop signs of infection unless they are immunocompromised [1–3]. One explanation for the age-restricted distribution of this disease is that mammalian neonates undergo an age-dependent maturation of their immune system following birth that renders newborns particularly vulnerable to infection [4–9]. Interaction with environmentally-derived microbes is considered to be a driving force for neonatal immune development [6, 7]. The intestinal microbiome of neonates is known to develop with age in various species, including foals [10–13]. In other species, expansion and diversification of the neonatal microbiome have been linked to development of intestinal function, including gut immunity [14, 15]. Furthermore, numerous studies have demonstrated that diminished diversity of the gastrointestinal microbiome leads to increased risk and severity of gastrointestinal diseases, systemic infections, and immune dysregulation [16]. Thus, we hypothesized that varying composition or reduced diversity of the neonatal foal intestinal microbiome would contribute to susceptibility of developing clinical signs of *R*. *equi* pneumonia. To test this hypothesis, we compared the composition and diversity indices of fecal samples collected at approximately 3 weeks and 5 weeks of age from 3 groups of foals: 1) foals that subsequently developed *R*. *equi* pneumonia after sampling; 2) foals that subsequently developed ultrasonographic evidence of pulmonary abscess formation or consolidation but not clinical signs; and, 3) foals that developed neither clinical signs nor ultrasonographic evidence of pulmonary abscess formation or consolidation.

Temporal changes in the foal microbiome remain ill-defined. To our knowledge, there are only 2 reports characterizing the microbiome of foals during the first month of life [13, 17]. While the fecal microbiome of foals changes rapidly and greatly during the first month of life [13, 17], current knowledge indicates that the fecal microbiome is relatively stable by 1 month of age. Thus, a second objective of our study was to better characterize the stability of the fecal microbiome in foals using sequential fecal samples collected over a 2-week interval spanning approximately 1 month of age.

## Materials and Methods

### Ethics statement

All protocols for this study were reviewed and approved by the Clinical Research Review Committee (CRRC Protocol 10–12) of the College of Veterinary Medicine & Biomedical Sciences, Texas A&M University. At the time this study was conducted, research involving client-owned animals at Texas A&M University was not subject to review by the Institutional Animal Care and Use Committee. All animals were monitored a minimum of twice daily by both veterinary staff and ranch personnel. All animals showing signs of pneumonia were examined by veterinary staff and treated as deemed appropriate by attending veterinarians, including the use of flunixin meglumine for analgesia. This study was conducted on private land (33°37′14″N 100°19′22″W) and specific permissions for use were granted by GPB. Written informed consent for participation was obtained for all foals included in the study, and the 6666 Ranch provided access to the foals included in this project. This study did not involve any endangered or protected species.

### Study population

The study was conducted at the 6666 Ranch because it agreed to provide access to foals, had history of *R*. *equi* pneumonia among foals with a cumulative incidence of ≥ 15% for the preceding 3 years, and because the farm’s veterinarian and general manager (GPB) had participated in a separate study during 2011 evaluating screening tests for *R*. *equi* pneumonia in foals; fecal samples for the study reported here were collected from the foals participating in the screening study [18]. For the study evaluating performance of screening tests, treatment for pneumonia was not initiated for any foal on the basis of screening test results alone, and the veterinarians making decisions about diagnosis and treatment of *R*. *equi* pneumonia were not informed of the results of screening tests. Bilateral thoracic ultrasonographic examination was performed on each foal at the farm at 2-week intervals, beginning at 3 weeks of age until either 19 weeks of age or until the foal developed clinical signs of pneumonia (as described below). The veterinarian performing the ultrasonographic examinations did not participate in either diagnosis or treatment of *R*. *equi* pneumonia. Ultrasonographic parameters recorded included: 1) the anatomic location (left versus right hemithorax; intercostal space; and, dorsal, middle, or ventral region); 2) maximal diameter of any areas of pulmonary abscesses or consolidation; and, 3) the total number of discrete lesions observed.

At the time of each sequential ultrasonographic examination, fecal samples were collected from foals, either by collecting freshly voided feces or by digital retrieval. When fresh fecal samples were collected, effort was made to obtain the central portion of the fecal matter (to limit environmental contamination). Fecal samples were double-bagged in sterile plastic sacks, frozen at -20°C for no more than 1 week, then shipped frozen and on ice to the Equine Infectious Disease Laboratory at Texas A&M University. Upon arrival, samples were evaluated for identifiers and date of collection (the 6666 Ranch was contacted to resolve any data that were missing or unclear), labelled, and stored in a freezer at -80°C. Data from samples (mare and foal identifiers, date collected, date received, weight of sample, and freezer location) were logged into a computerized spreadsheet for data management.

All foals [N = 270] born during 2011 at the ranch were eligible to be included in the study. Farm personnel monitored the foals daily for clinical signs of pneumonia until 20 weeks of age. Clinical signs indicative of pneumonia included fever, lethargy, signs of depressed attitude, cough, nasal discharge, polysynovitis, tachypnea, increased respiratory effort, respiratory distress, and detection of a tracheal rattle or pulmonary crackles or wheezes during thoracic auscultation. Thoracic ultrasonography and collection of a trans-endoscopic tracheobronchial aspirate (TBA) sample with a commercially available triple-guarded catheter (Triple stage tracheal wash catheter, MILA International Inc., Erlanger, KY) were performed for each foal that developed clinical signs of pneumonia. The endoscope was disinfected between procedures with a 3.4% glutaraldehyde solution (CIDEX-PLUS, Advanced Sterilization Products, Irvine, CA), following a standard protocol used in our laboratory and known to be microbiocidal against*R*. *equi*. Each sample of TBA fluid was submitted for microbiologic culture and cytologic evaluation to the Texas Veterinary Medical Diagnostic Laboratory in College Station, Texas (culture) and Veterinary Clinical Pathology Laboratory, College of Veterinary Medicine and Biomedical Sciences, in College Station, Texas (cytology).

Foals with signs of pneumonia at 3 to 20 weeks of age, ultrasonographic evidence of peripheral pulmonary consolidation or abscesses at the time of examination for clinical signs of pneumonia, and *R*. *equi* isolated from TBA fluid via microbiologic culture, and cytological evidence of gram-positive intracellular coccobacilli in the TBA sample were defined as having *R*. *equi* pneumonia (**clinical group**; N = 43 [17%]). Foals having ultrasonographic evidence of peripheral pulmonary consolidation or abscesses, but lacking clinical signs of pneumonia were defined as being subclinical pneumonia foals (**subclinical group**; N = 156 [63%]) [19]. Foals having no clinical signs of pneumonia and no ultrasonographic evidence of pulmonary consolidation or abscessation were considered unaffected foals (**healthy group**; N = 49 [20%]). For purposes of this study, 25 clinical group foals were randomly selected from the 43 foals in the clinical group. These 25 foals were birthdate-matched both to 25 foals from the subclinical group and to 25 foals from the healthy group. Fecal samples from 2 time-points for each foal were selected for sequencing. The first sample for all foals (time 1) was the first fecal sample collected (3 weeks of age), and the second fecal sample was collected at 5 weeks of age. For foals that developed *R*. *equi* pneumonia, the second sample always preceded the onset of clinical signs of disease (i.e., before any treatment was instituted and before inappetance or other clinical signs of disease were observed).

### DNA extraction

The fecal sample was removed from storage at -80°C and 200 mg of feces was harvested from the frozen sample. Genomic DNA was extracted from using a commercially available fecal DNA extraction kit (QIAamp Fast DNA Stool Mini Kit, Qiagen) according to manufacturer’s protocol with slight modifications. Briefly, 200 mg of frozen feces was placed in a 2-ml tube that contained 1 ml Inhibitex buffer and 50 mg each of sterile/DNAase free 0.1- and 0.5-mm silica zirconium beads. This was then homogenized for 90 seconds at 6.5 m/sec with FastPrep FP120 cell disrupter (Qbiogene, Carlsbad,CA). The sample was then heated at 70°C for 10 minutes prior to following the manufacturer’s protocol for DNA extraction. DNA was suspended in tris-EDTA buffer (Integrated DNA Technologies, Coralville, Iowa) and stored at -80°C.

### Sequencing of 16S rRNA gene

Amplification and sequencing of the V4 variable region 16S rRNA gene was performed at MR DNA (www.mrdnalab.com, Shallowater, TX, USA) [20]. Briefly samples were barcoded and PCR primers 515F/806R were used in a 28 cycle PCR using the HotStarTaq Plus Master Mix Kit (Qiagen, USA) under the following conditions: 94°C for 3 minutes, followed by 28 cycles of 94°C for 30 seconds, 53°C for 40 seconds and 72°C for 1 minute, with a final elongation step at 72°C for 5 minutes. A DNA library was prepared according to Illumina TruSeq DNA library preparation protocol. Sequencing was performed on a MiSeq (Illumina) following the manufacturer’s guidelines. Sequence data was uploaded into NCBI GenBank database submission number SUB995994.

### Analysis of sequencing data

The software Quantitative Insights Into Microbial Ecology (QIIME v1.9 (http://qiime.sourceforge.net) was used for data processing and analysis [21]. The raw sequence data were de-multiplexed, and low quality reads were filtered using database’s default parameters. Chimeric sequences were detected using Uchime and removed prior to further analysis [22]. Sequences were then assigned to operational taxonomic units (OTUs) using an open-reference OTU picking protocol in QIIME [23] against the Greengenes database filtered at 97% identity [24, 25]. To adjust for uneven sequencing depth among the samples, each sample was rarefied to an even sequencing depth per sample prior to further analysis.

Alpha rarefaction, beta diversity measures, richness, taxonomic summaries, and tests for significance were calculated and plotted using QIIME. Diversity indices were compared using a generalized linear model with a given index as the outcome variable and the 3 health groups as independent variables, with post hoc testing for pairwise differences among groups using the method of Sidak. The same approach was used to compare differences between times. Differences in microbial communities among the groups were investigated by visual assessment of clustering on principal coordinates analysis (PCoA) plots, and by analysis of similarity (ANOSIM) [26] calculated on unweighted and weighted UniFrac distance metrics [27] using available QIIME scripts. When ANOSIM identified significant differences among groups, then similarity percentage (SIMPER) [26] was used with Bray Curtis dissimilarity metric [28] to examine which features contributed to the differences among groups. SIMPER was performed with PAST v3.05 [29]. Differences in the proportions of bacteria making up the composition of the microbiota in different groups was also determined by the Mann-Whitney U test when only 2 comparisons were made (i.e., time differences) and the Kruskal-Wallis test when multiple comparisons were made (e.g., differences between the 3 phenotypes of disease). For all analyses, significance was set at P ≤ 0.05 after correction using the Benjamini-Hochberg FDR procedure. The groups compared were: 1) healthy, subclinical, and clinical foals at time 1 (age 3 weeks); 2) healthy, subclinical, and clinical foals at time 2 (age 5 weeks); and, 3) all foals at time 1 compared with all foals at time 2, regardless of health status.

The software Phylogenetic Investigation of Communities by Reconstruction of Unobserved States (PICRUSt) was used to predict the metagenome [30]. Sequencing data were prepared as described above, but sequences were then clustered into OTUs using a closed-reference OTU picking protocol at the 97% sequencing identity level. The resulting OTU table was normalized by the expected copy number(s) of the 16s rRNA gene in each OTU. PICRUSt was then used to predict the metagenome. Each sample was rarefied to an even sequencing depth to adjust for uneven sequencing depth prior to further analysis. Differences in the metagenomes among the groups were investigated by visual assessment of clustering on principal coordinates analysis (PCoA) plots, and by analysis of similarity (ANOSIM) calculated on Bray Curtis dissimilarity metric using the available QIIME scripts. The KEGG orthologies and pathways identified [31] via PICRUSt were analyzed and compared as described above.

## Results

### Data

**Fig 1:**
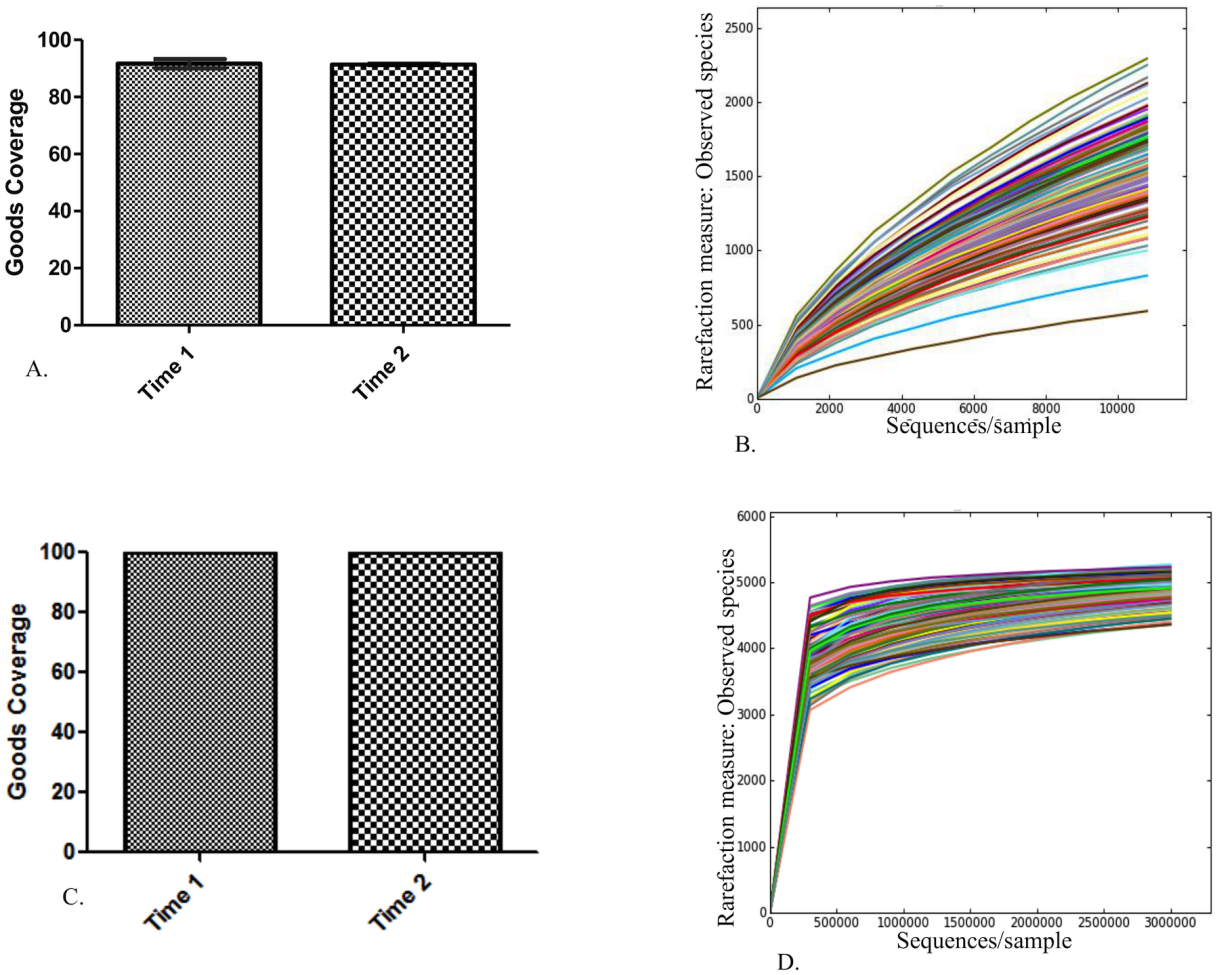
Data filtering and even sampling depth suggest adequate coverage for subsequent analysis. A) Bar charts of estimated goods coverage at an even sampling depth of 10,800 reads/sample at time 1 (dotted pattern) and time 2 (checkerboard pattern). Error bars represent the standard deviation. B) Alpha rarefaction curves for all samples showing numbers of observed species at each sampling depth on the y-axis and sequences/sample on the x-axis up to a sampling depth of 10,800 sequences/sample. C) Bar charts of estimated goods coverage at an even sampling depth of 3,000,000 predicted KEGG orthologies/sample at time 1 (dotted pattern) and time 2 (checkerboard pattern). Error bars represent the standard deviation. D) Alpha rarefaction curves for all samples showing numbers of observed species (KEGG orthologies) at each sampling depth on the y-axis and sequences/sample on the x-axis up to a sampling depth of 3,000,000 sequences/sample.

A total of 16,386,847 sequences were obtained from the sequencing facility. After quality filtering, 9,471,315 reads remained with a median of 65,108 sequences per sample (range, 3 to 136,547 sequences per sample). The Usearch61 method of chimera detection was used to identify 820,087 chimeric sequences, and these were removed prior to OTU picking. Open reference OTU picking was used to cluster sequences into 86,866 unique OTUs with a median of 57,740 counts/sample (range, 9 to 121,213 counts/sample) with a total count of 8,532,117. Very low frequency OTUs (i.e., OTUs represented fewer than 4 times) or OTUs present in fewer than 4 animals were removed. This reduced the number of OTUs into which sequences were clustered to 37,184 with a total count of 8,389,450. Although this filtering reduced the number of OTUs by 58% it resulted in only a 2% decrease in the number of counts (i.e., 98% of the counts were retained). Finally, an even sequencing depth of 10,800 reads per sample was selected. Although alpha rarefaction curves had not plateaued at this sampling depth, Good’s coverage index estimates indicated that over 90% of the species were represented at both time points (median, 91%; Fig 1A and 1B). Following quality filtering, OTU picking, removal of ultra-low abundance OTUs, and selecting an even sampling depth of 10,800 reads/sample, 139 samples remained for analysis.

Closed reference OTU picking was used to identify OTUs for use in PICRUSt to predict the metagenome. Closed reference OTU picking resulted in sequences being clustered into 11,300 unique OTUs with a total count of 7,026,925 (median, 47,894 counts/sample). These were filtered as above, such that removal of OTUs identified fewer than 4 times reduced the number of unique OTUs to 6,923 and reduced the total OTU count to 7,020,091; removal of OTUs identified in fewer than 4 animals further reduced the number of unique OTUs to 6,393 and total count to 7,016,295. The resulting OTU table was normalized by the expected copy number(s) of the 16S rRNA gene for each OTU prior to metagenome predictions. Metagenome predictions resulted in OTUs being clustered into 6,909 unique KEGG orthologies. An even sampling depth of 3,000,000 reads per sample was chosen. Alpha rarefaction curves and Good’s coverage index suggest 3,000,000 reads/sample adequately covered the predicted metagenome (Fig 1C and 1D).

### Differences in fecal microbiota among the health groups at time 1

**Fig 2:**
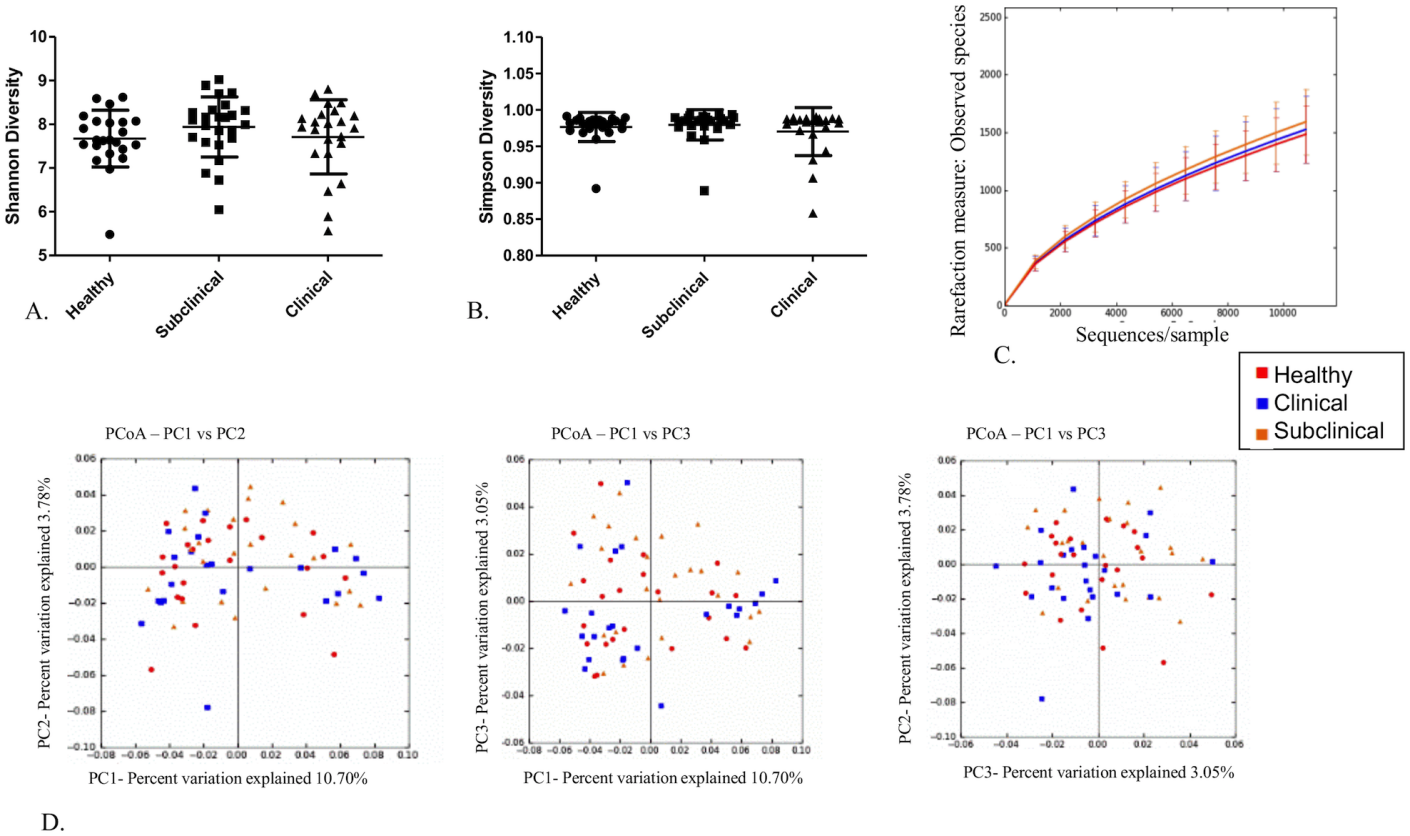
No differences in the fecal microbiota among the health groups at time 1. A) Scatter dot plot of Shannon diversity index for all health groups; healthy (dots), subclinical (squares), and clinical (triangles) at time 1. Horizontal lines represent the mean for each group and error bars represent the standard deviation. There were no statistical differences among the groups. B) Scatter dot plot of Simpson diversity index for all health groups; healthy (dots), subclinical (squares), and clinical (triangles) at time 1. Horizontal lines represent the mean for each group and error bars represent the standard deviation. There were no statistical differences among the groups. C) Alpha rarefication curves for each of the health groups; healthy (red), clinical (blue), subclinical (orange) at time 1 showing numbers of observed species at each sampling depth on the y-axis and sequences/sample on the x-axis up to 10,800 sequences/sample. Error bars represent standard deviations of each group at the specified sampling depth. D) Principal coordinate analysis of unweighted Unifrac distance metric for all heath groups; healthy (red), clinical (blue), subclinical (orange) at time 1. There were no differences among the groups.

There were no significant differences in the diversity indices or richness calculated for the health groups at time 1 **(** Fig 2A–2C). Moreover principal coordinate analysis (PCoA) plots based on the unweighted UniFrac distance metric revealed no apparent clustering of the health groups at time 1 (Fig 2D). ANOSIM analysis of both the weighted (R -0.0023, P-value 0.502) and unweighted (R = 0.0029; P = 0.379) Unifrac distance metric revealed no significant difference among the health groups at time 1, and Kruskal-Wallis analysis revealed no significant differences in abundances of any OTUs among the health groups at time 1(data not shown).

### Differences in predicted metagenome among health groups at time 1

**Fig 3:**
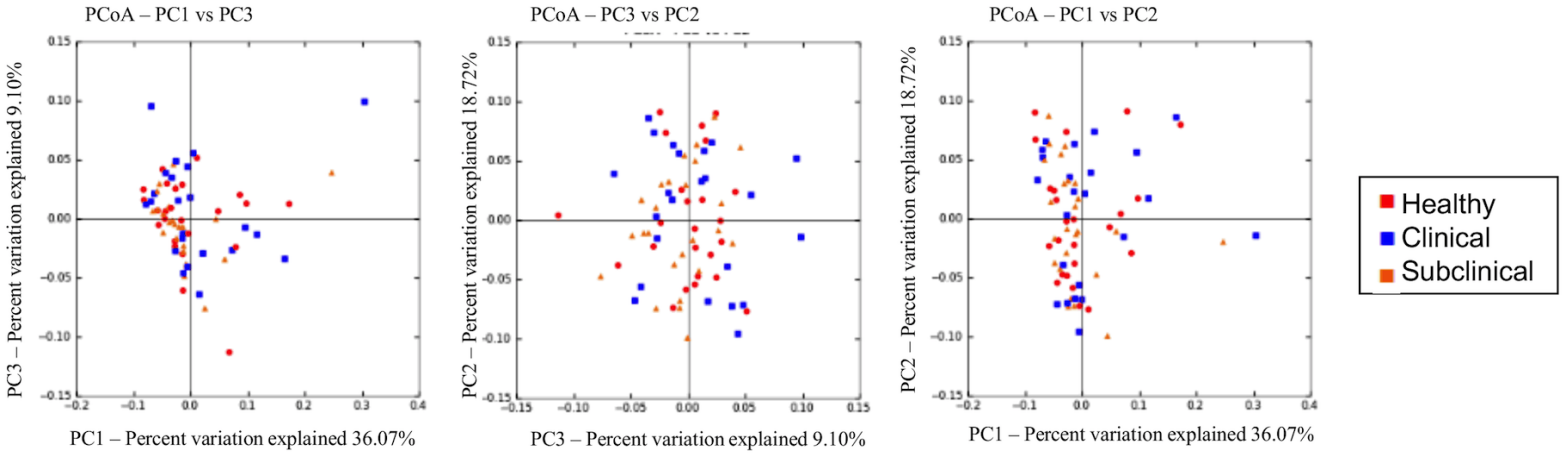
Principal coordinate analysis plots of the predicted metagenome for health groups at time 1 based on the Bray Curtis dissimilarity metric. Principal coordinate analysis of the Bray Curtis dissimilarity metric of the predicted metagenome for all heath groups; healthy (red), clinical (blue), subclinical (orange) at time 1. There were no differences among the groups.

Principal coordinate analysis plots based on the Bray Curtis dissimilarity metric revealed no graphical evidence of clustering of the predicted metagenome of the health groups at time 1 (Fig 3). ANOSIM analysis of the dissimilarity metric revealed no significant difference in the predicted metagenome among the health groups at time 1 (R = 0.0016; P = 0.370). In addition, Kruskal-Wallis testing revealed no significant differences in the abundance of any KEGG pathways among the health groups at time 1 (data not shown).

### Differences in fecal microbiota among the health groups at time 2

**Fig 4:**
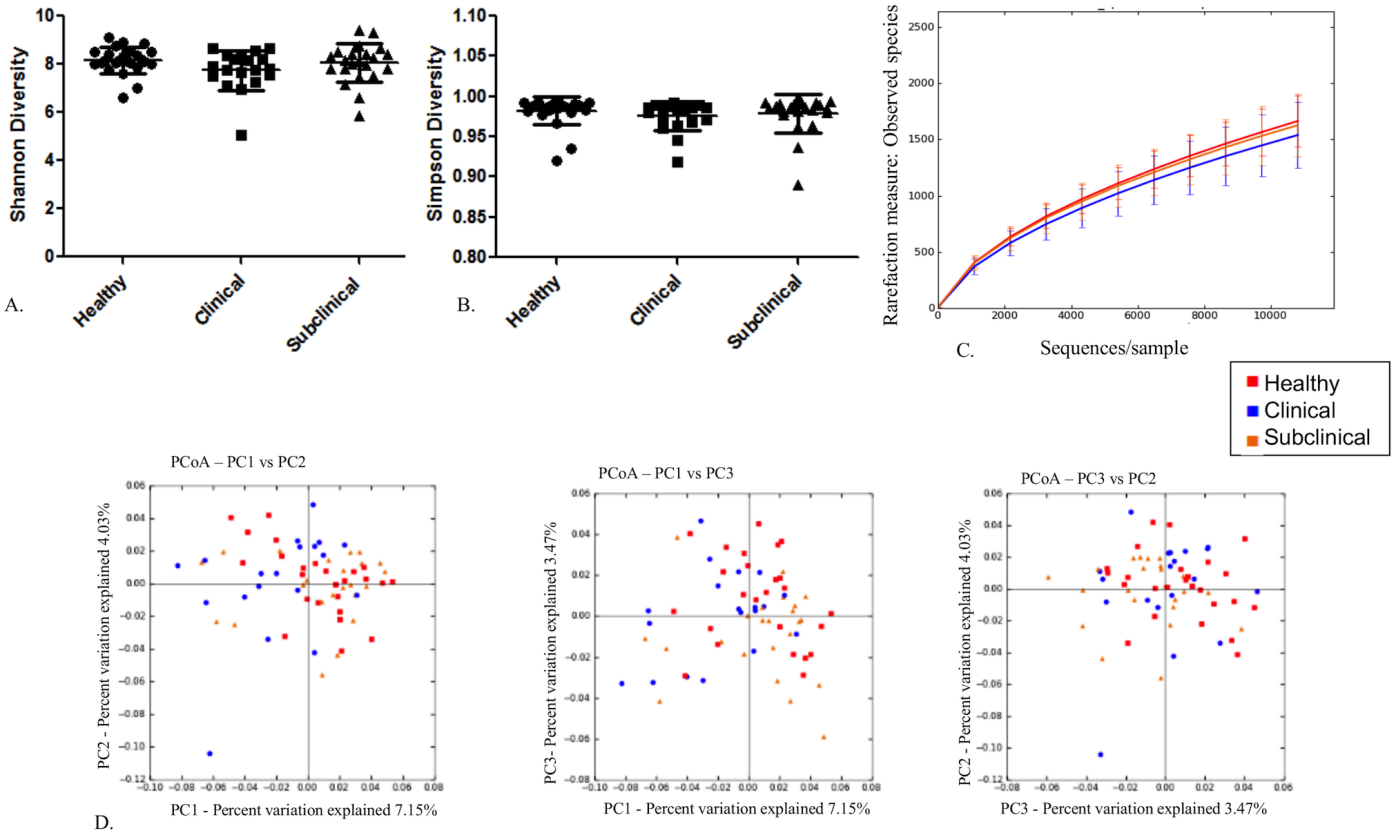
No differences in the fecal microbiota among the health groups at time 2. A) Scatter dot plot of Shannon diversity index for all health groups; healthy (dots), subclinical (squares), and clinical (triangles) at time 2. Horizontal lines represent the mean for each group and error bars represent the standard deviation. There were no statistical differences among the groups. B) Scatter dot plot of Simpson diversity index for all health groups; healthy (dots), subclinical (squares), and clinical (triangles) at time 2. Horizontal lines represent the mean for each group and error bars represent the standard deviation. There were no statistical differences among the groups. C) Alpha rarefication curves for each of the health groups; healthy (red), clinical (blue), subclinical (orange) at time 2 showing numbers of observed species at each sampling depth on the y-axis and sequences/sample on the x-axis up to 10,800 sequences/sample. Error bars represent standard deviations of each group at the specified sampling depth. D) Principal coordinate analysis of unweighted Unifrac distance metric for all heath groups; healthy (red), clinical (blue), subclinical (orange) at time 2. There were no differences among the groups.

There were no differences in the diversity indices or richness calculated for the health groups at time 2 (Fig 4A–4C). Moreover, principal coordinate analysis (PCoA) plots based on the unweighted UniFrac distance metric revealed no apparent clustering of the health groups at time 2 (Fig 4D). The unweighted Unifrac distance metric was small but significantly different than 0 using ANOSIM analysis of the distance metric at time 2 (R = 0.072; P = 0.005). When using the weighted distance metric, however, the distance metric was small and there was no significant difference among the groups at time 2 (R = 0.0303; P = 0.083). Kruskal-Wallis tests revealed no significant differences in abundances of any OTUs among the health groups at time 2 (data not shown).

### Differences in predicted metagenome among health groups at time 2

**Fig 5:**
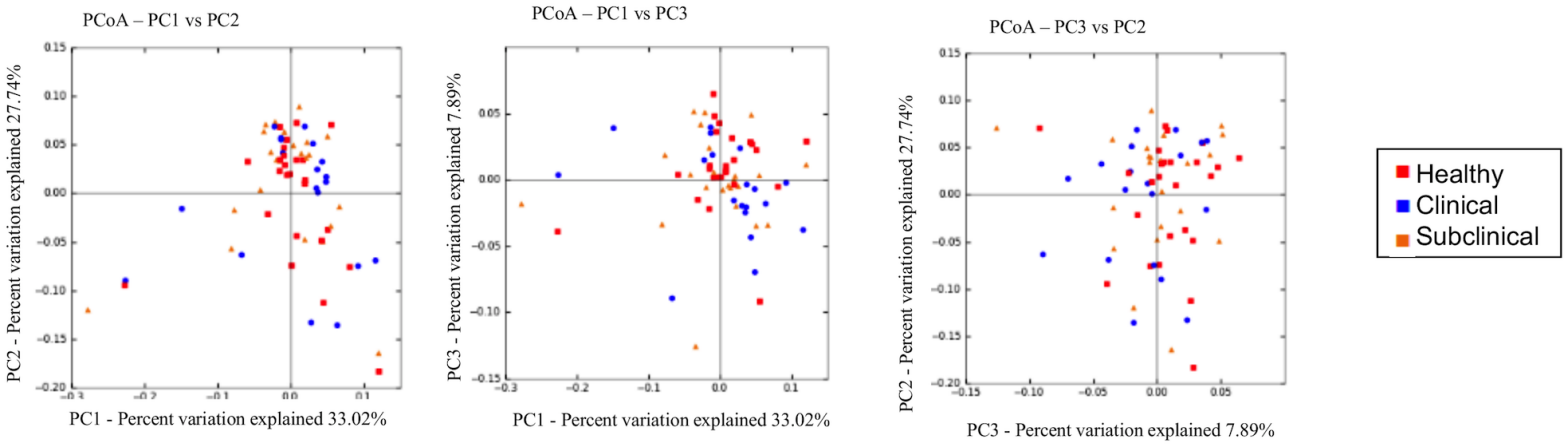
Principal coordinate analysis plots of the predicted metagenome for health groups at time 2 based on the Bray Curtis dissimilarity metric. Principal coordinate analysis of the Bray Curtis dissimilarity metric of the predicted metagenome for all heath groups; healthy (red), clinical (blue), subclinical (orange) at time 2. There were no differences among the groups.

Principal coordinate analysis plots based on the Bray Curtis dissimilarity metric revealed no visual clustering of the predicted metagenome of the health groups at time 2 (Fig 5). ANOSIM analysis of the distance metrics revealed no significant difference in the predicted metagenome among the health groups at time 1 (R = 0.0149; P = 0.212). In addition, Kruskal-Wallis test revealed no significant differences in the abundance of any KEGG pathways among the health groups at time 2 (data not shown).

### Differences in fecal microbiota between time 1 and time 2

**Fig 6:**
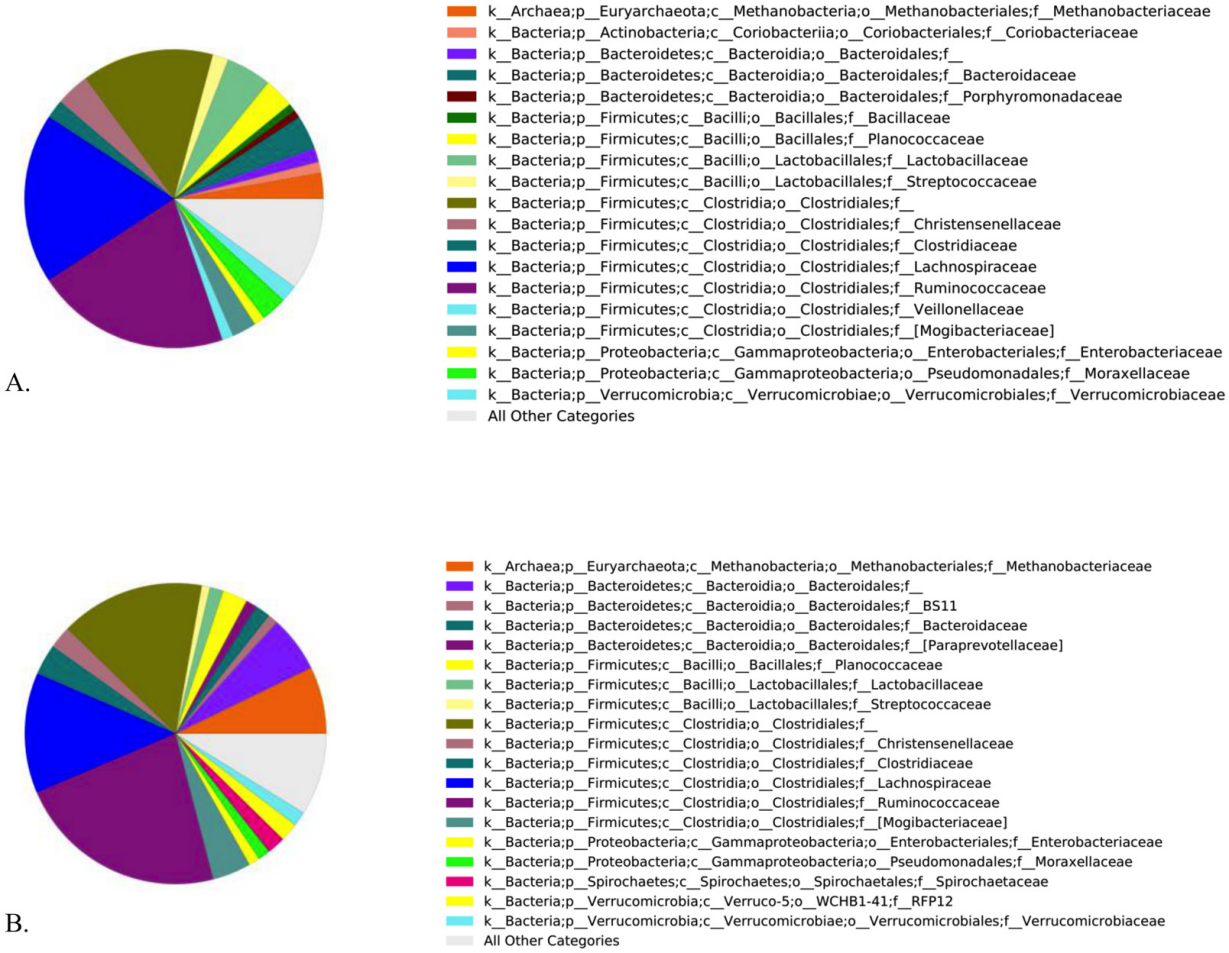
Major bacterial families present at (A) time 1 and (B) time 2. A) Pie charts showing the distribution of the major (>1% mean abundance) of the families of bacteria and archea present at time 1. Each Family is colored by a different color and the color for each Family and the Phylum to which it belongs is shown in the figure legend accompanying the pie chart. B) Pie charts showing the distribution of the major (>1% mean abundance) of the families of bacteria and archea present at time 2. Each Family is colored by a different color and the color for each family and the phylum to which it belongs is shown in the figure legend accompanying the pie chart.

**Fig 7:**
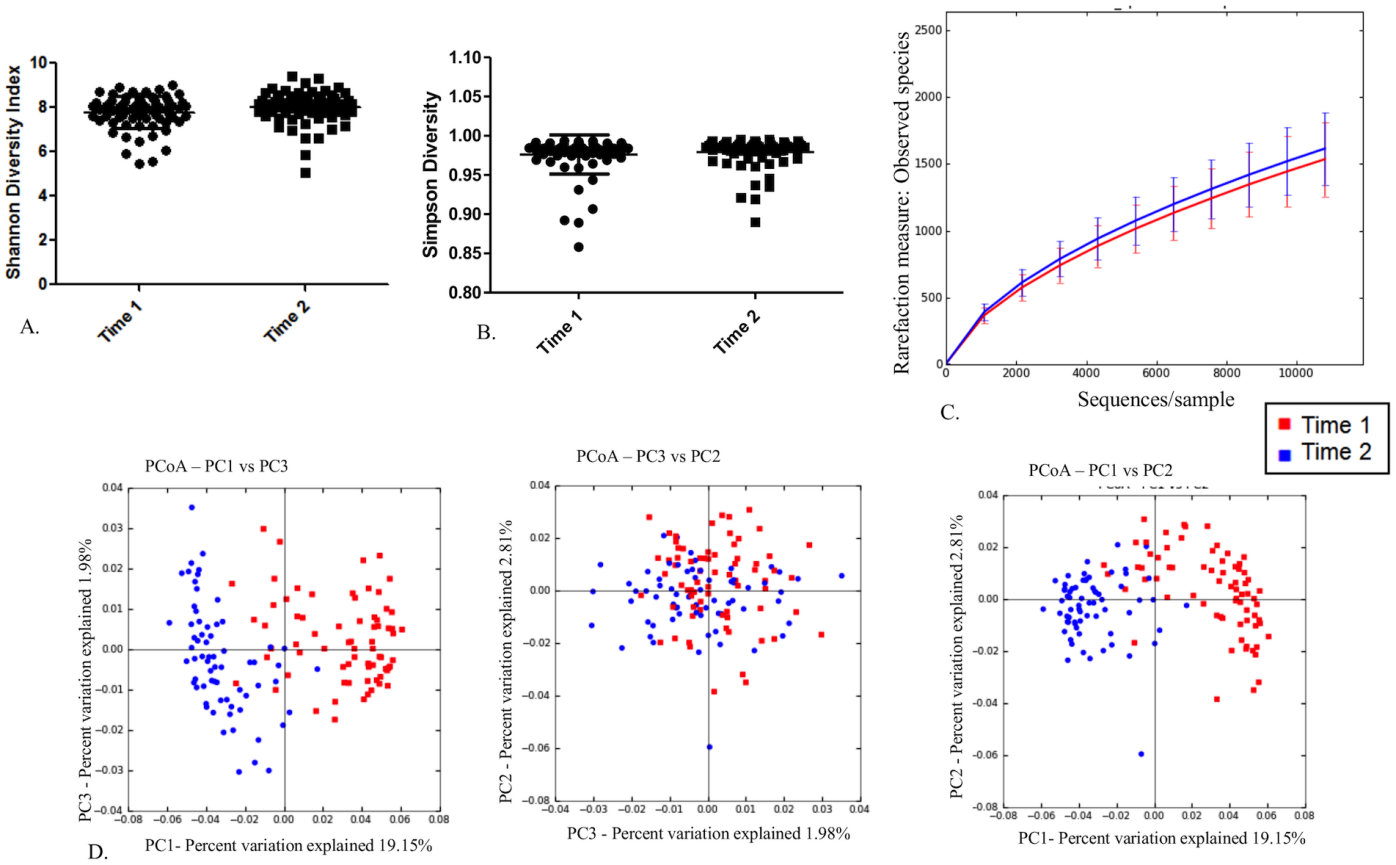
No differences in the diversity or richness of the fecal microbiota between time 1 and time 2 but the composition of the fecal microbiota is different between time 1 and time 2. A) Scatter dot plot of Shannon diversity index for time 1 (dots) and time 2 (squares). Horizontal line represents mean and error bars represent the standard deviation. There were no statistical differences between the groups. B) Scatter dot plot of Simpson diversity index for time 1 (dots) and time 2 (squares). Horizontal line represents mean and error bars represent the standard deviation. There were no statistical differences between the groups. C) Alpha rarefication curves for time 1 (red) and time 2 (blue) showing numbers of observed species at each sampling depth on the y-axis and sequences/sample on the x-axis up to 10,800 sequences/sample. Error bars represent standard deviations of each group at the specified sampling depth. D) Principal coordinate analysis of unweighted Unifrac distance metric for time 1 (red) and time 2 (blue). There was obvious clustering of the time 1 samples and the time 2 samples.

The distribution of the major (frequency > 1%) families of microbes present at time 1 and time 2 were plotted as pie charts (Fig 6). There were no differences in the diversity indices or richness calculated for horses at times 1 or 2 irrespective of health status (Fig 7A–7C). The PCoA plots of the first 3 principal coordinates based on the unweighted UniFrac distance metric revealed obvious visual clustering of the health groups by time (Fig 7D). ANOSIM revealed significant differences of both the unweighted Unifrac distance metric (R = 0.6945; P = 0.001) and weighted Unifrac distance metric (R = 0.2566; P = 0.001). In order to identify bacterial taxa that contributed to the difference between times, we performed SIMPER analysis at the levels of both the phyla and family. Mean change in abundance with time and percent contribution to differences in the Bray Curtis dissimilarity metric were tabulated (Table 1 for Phyla and S1 Table for Family). Mann-Whitney U tests on the proportion of OTUs per sample revealed many differences in the abundance of OTUs between time 1 and time 2 samples (S2 Table).

**Table 1:** SIMPER analysis identifying the % contribution of each phylum to the Bray Curtis dissimilarity metric between time 1 and time 2.

Phyla	% Contribution	Cumulative %	Mean abundance time 1	Mean abundance time 2	Change in abundance with time
k__Bacteria;p__Firmicutes	31.71	31.71	77.00%	70.10%	-6.90%
k__Bacteria;p__Bacteroidetes	18.23	49.94	8.27%	10.80%	2.53%
k__Bacteria;p__Proteobacteria	14.89	64.82	5.32%	3.02%	-2.30%
k__Archaea;p__Euryarchaeota	13.84	78.66	2.82%	7.14%	4.32%
k__Bacteria;p__Verrucomicrobia	7.509	86.17	2.35%	3.65%	1.30%
k__Bacteria;p__Spirochaetes	4.411	90.58	0.49%	2.11%	1.62%
k__Bacteria;p__Actinobacteria	2.868	93.45	1.88%	1.20%	-0.68%
k__Bacteria;p__Chlamydiae	1.484	94.93	0.21%	0.51%	0.30%
k__Bacteria;p__WPS-2	1.142	96.07	0.43%	0.09%	-0.34%
k__Bacteria;p__Fusobacteria	0.8746	96.95	0.31%	0.19%	-0.13%
k__Bacteria;p__Tenericutes	0.5851	97.53	0.32%	0.17%	-0.15%
Unassigned;Other	0.5642	98.1	0.23%	0.34%	0.10%
k__Bacteria;p__Fibrobacteres	0.4846	98.58	0.02%	0.21%	0.19%

Similarity percentage analysis of the phyla differences between time 1 and time 2. The first column identifies the phylum explained by that row, the second column shows the % dissimilarity explained by that phylum, the third column tallies the cumulative Bray Curtis dissimilarity metric for the phyla thus far represented in the table, and the last three columns show mean abundance at time 1, mean abundance at time 2, and change in mean abundance respectively

### Differences in predicted metagenome between time 1 and time 2

**Fig 8:**
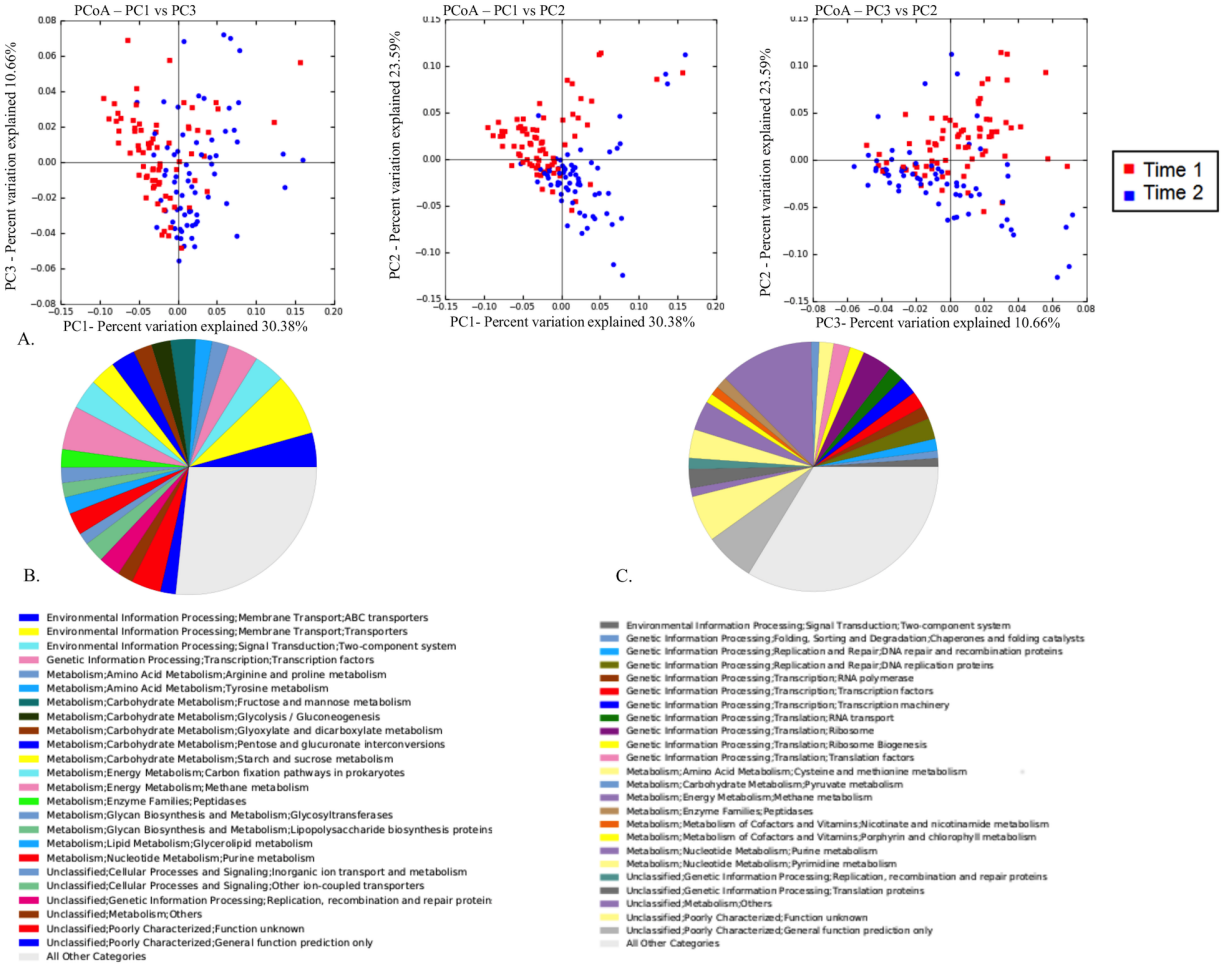
The composition of the predicted metagenome changes between time 1 and time 2. (A) Principal coordinate analysis of the Bray Curtis dissimilarity metric of the predicted metagenome for time 1 (red) and time 2 (blue). There was obvious visual clustering of the predicted metagenome at time 1 and time 2. B) Pie chart showing the predicted functional pathways that were significantly (Mann Whitney U test FDR P value < 0.05) decreased >2 fold between time 1 and time 2. The figure legend accompanying the figure explains which pathways are represented by which colors. C) Pie chart showing the predicted functional pathways that were significantly (Mann Whitney U test FDR P value < 0.05) increased >2 fold between time 1 and time 2. The figure legend accompanying the figure explains which pathways are represented by which colors.

As expected from the observed time-dependent fecal microbiota differences, there also were time-dependent differences in the predicted metagenome revealed by PCoA of the Bray Curtis dissimilarity metric (Fig 8A). ANOSIM results calculated on the Bray Curtis dissimilarity metric revealed significant differences of the predicted metagenome at time 1 and time 2 (R = 0.2376; P = 0.001). There were 367 KEGG orthologies that were significantly (adjusted P < 0.05) decreased more than 2-fold between time 1 and time 2, and 486 KEGG orthologs were significantly (adjusted P < 0.05) increased by more than 2-fold between time 1 and time 2 (S3 Table). The different pathways to which these KEGG orthologies belong are represented as pie charts (Fig 8B and 8C). Approximately half (13) of the top 25 predicted functional pathways that were decreased by 2-fold or more between 3 and 5 weeks were related to metabolism. Moreover, the majority of these decreased metabolic pathways were related to metabolism of simple sugars (sucrose, glucose, fructose, and mannose) and amino acids (tyrosine, arginine, and proline). Predicted functional pathways that were increased more than 2-fold over the same time period were evenly distributed among metabolic pathways, transcriptional pathways, and a myriad of unclassified or unknown pathways.

## Discussion

The distribution of virulent *R*.*equi* at equine breeding farms appears to be widespread, and foals are exposed to the bacterium from birth [1–3, 32–41]. Commensurate with this widespread exposure, many if not most foals at affected breeding farms appear to develop pulmonary abscess formation or consolidation attributed to *R*. *equi* without developing disease (i.e., subclinical pneumonia); however, most of these foals with subclinical pulmonary lesions do not develop clinical signs of disease [18, 42–45]. It is unclear why, among foals from the same environment with presumably similar exposure to virulent *R*. *equi*, some develop clinical signs of pneunonia while others either develop subclinical pneumonia or remain free of disease and pulmonary lesions. One possible explanation for this phenomenon is variation in immune function among foals. All foals are born with an immune system that is naïve, lacking circulating immunoglobulins, and with various diminutions in innate and adaptive immune responses [4, 5]. The intestinal microbiome in neonates (including foals) is known to expand and diversify following birth, and this expansion and diversification of the microbiome is known to influence various host developmental processes including intestinal and systemic immunity to infection [10–16]. Thus, we hypothesized that the difference in susceptibility to *R*. *equi* pneumonia among foals might be attributable to differences in the composition or diversity of the intestinal microbiome resulting in less developed immune responses in some foals that rendered these foals more susceptible to infection. To test this hypothesis, we compared the fecal microbiome using samples collected prior to the onset of clinical disease in 75 foals: 25 foals that developed *R*. *equi* pneumonia; 25 that developed subclinical pneumonia presumed to be attributed to *R*. *equi* infection; and, 25 healthy foals (i.e., foals free of clinical signs and ultrasonographic evidence of pulmonary lesions). In addition to being appropriate for predictive purposes, the strategy of collecting samples prior to the onset of disease also had the advantage of avoiding the influence of disease or any antimicrobial treatment for disease on the fecal microbiome; antimicrobial treatment, systemic inflammatory responses, inflammation, and reduced appetite can alter the intestinal environment and consequently the microbiota [46]. Thus, collecting samples prior to onset of disease and treatment obviated the confounding effects of these factors on the association of the fecal microbiome composition and diversity with *R*. *equi* pneumonia. Our goal was to examine changes in the fecal microbiome during a brief interval after the initial rapid colonization of the foal’s gastrointestinal tract (i.e., around age 1 month) when the foals’ microbiota were more stable but before any confounding effects of illness or treatment might have impacted the microbiota.

Our results did not provide strong evidence against the null hypothesis of no difference in the microbiota among the 3 clinical groups (*R*. *equi* pneumonia, subclinical pneumonia, or healthy). At either time-point, the diversity of organisms was similar among the 3 groups (Figs 3A–3B and 5A–5B) and there was no significant difference in any of the diversity indices evaluated among groups. At the first sample-time (3 weeks of age), neither PCoA, alpha diversity indices, nor measure of overall richness (Fig 3) revealed evidence of significant differences among groups. At time 2 (5 weeks), there was a weak but statistically significant difference detected when using ANOSIM to determine similarity of the Unifrac unweighted distance metric based on health status (Fig 5C). When OTUs were weighted for abundance, however, this association was no longer significant. In light of the graphical and statistical results, we interpret these findings to indicate that there were no clinically important differences in the fecal microbiome among the groups at either time-point.

A strong and significant difference in the 2 sample time-points was observed in both the composition of the fecal microbiota and the predicted metagenome. This finding was somewhat surprising to us considering that most (88%; 22/25) of these samples were collected 2 weeks apart. The observed changes were largely characterized by a decrease in members of the phylum Firmicutes and an increase in members of the phylum Bacteroidetes. In fact, 50% of the dissimilarity between the fecal microbiota from time 1 and time 2 was explained by changes in members of these 2 phyla. A recent study [47] identified the core microbiota of the large intestine and feces of adult horses. In that study, the relative abundance of Firmicutes and Bacteroidetes in feces was nearly equal at 46% and 43% respectively. Among our foals, the relative abundance of Firmicutes was 73% and the relative abundance of Bacteroidetes was 10%; therefore, the decreased abundance of Firmicutes and increased abundance of Bacteroidetes between the later and earlier samples in our study might have reflected continuation of a gradual transition to an adult horse-like composition of the fecal microbiota. These findings support the notion that the microbiota of foals is rapidly changing up to 7 weeks of age and thus may influence immune development. Foals gradually consume less milk and more solid feedstuffs between birth and ~21 weeks of age as they transition to becoming more reliant on hind-gut fermentation for meeting caloric requirements [48, 49]. Clearly, as a foal’s diet changes, their microbiota adapts to changes in dietary composition. Despite the fact that this physiological transition is occurring over a period of months, we noted large changes during a period as short as 2 weeks between 3 and 5 weeks of age. Other studies have demonstrated that the foals fecal microbiota community changes gradually with age but appears to climax around 30 days of age with no additional significant changes between 30 and 60 days of age [17]. Our second (latter) sample was collected from 5-week-old foals; therefore, it is possible that we have characterized the final stages of maturation of the fecal microbiota. Alternatively, our results indicate that the fecal microbiota of foals might be continuing to change significantly beyond the first month of life. Interestingly, these temporal changes in the microbiota were not accompanied by a significant change in overall diversity or richness. Thus, the observed compositional changes were not attributable to acquiring a greater diversity of organisms comprising the fecal microbiome but instead to altered abundance of the populations present.

One plausible explanation for the finding that the composition but not diversity changed between times 1 and 2 might was that it reflected a functional change in the microbiota. Indeed, the changes in the fecal microbiota composition between time 1 and time 2 were reflected in the predicted functional pathways of the microbiota. We examined the alterations in the functional pathways of the predicted metagenome in order to determine how fecal microbiota changes may affect the host. Interestingly, many of the predicted functional pathways that were observed to be altered during this time were related to metabolism. There appeared to be a decrease in predicted functional pathways related to metabolism of simple sugars found in milk and to an increase in metabolic pathways related to fermentation and more complex energy metabolism (Fig 8). These metabolic pathway changes suggest that indeed the microbiota is changing to accommodate the changing diet of these foals. It is possible that the changes observed in the functional pathways also influenced immune development and function as age-related immunologic changes have been observed over the same timeframe as the foals in our study [8]. Unfortunately, immune function of foals in this study was not assessed.

Our study had a number of important limitations. First, the sample size was relatively modest. Nevertheless, these data represent the largest group of foals whose microbiome has been evaluated to date. Second, the timing of samples might have been inappropriate. We selected 3 weeks and 5 weeks of age by convenience in order to examine the narrow window after foals’ had acquired a microbiota that was relatively stable but before any confounding effects of illness might have been present. Epidemiological evidence exists that many foals become infected early in life [50, 51], and that foals are more susceptible to infection with *R*. *equi* during the first week or 2 of life [52]. Thus, we might have selected sample time-points that were after the age at which susceptibility was most important and, possibly, most apparent; put another way, the critical period for evaluation might have been earlier in life when the composition and diversity of the microbiota were less complex [13]. Third, we examined only the fecal microbiome. Although fecal samples are often used as indicators of the intestinal microbiota, it has been shown that fecal microbiota data do not always correlate with samples taken from regions of the gastrointestinal tract, and that variation in the microbiota occurs among different anatomical sites of the intestinal tract. For example, fecal samples might not have reflected the microbiota inhabiting the intestinal mucosa; the mucosal microbiota might be more important for immune development because it is more intimately associated with the immune cells of the host’s intestinal tract. Finally, our overall hypothesis was that varying composition or reduced diversity of the neonatal foal intestinal microbiome would contribute to susceptibility of developing clinical signs of *R*. *equi* pneumonia presumably because of differences in immune function related to differences in the intestinal microbiome. Unfortunately, we did not assess immune function of the study foals. Thus, had we observed a difference in the intestinal microbiota we would have failed to demonstrate a difference in the immunological response. Alternatively, we cannot exclude the possibility that differences in immune function existed between affected and unaffected foals at or between sampling times despite lack of apparent difference in the composition or diversity of the fecal microbiota. Future studies in which immune function and microbiota characterization are performed contemporaneously are recommended to obviate this important limitation.

Our population was limited to a single farm in Texas. We do not know to what extent our results from foals in western Texas may be extrapolated to other populations of foals or even other farms in the same region. Last, we classified the microbiota based on amplification of the V4 hypervariable region of the 16S rRNA gene. While this region has been recommended by some investigators[53], V4 is not considered by all investigators to be ideal for microbiomic analysis of differences by phenotype [54]. Thus, using only the V4 region to classify microbes might have limited our ability to detect differences among foals in the various health groups.

Although we failed to identify differences among our study groups, it remains possible and plausible that changes in the intestinal microbiome influence maturation of the immune system in foals because it has been shown in other species that immune development is strongly influenced by the intestinal microbiota. Thus, our absence of evidence favoring our hypothesis of differences among clinical groups is not the same as evidence of absence of differences among the groups. We did observe a marked and significant difference between a relatively short interval of time that appeared to reflect adaptation to transition from a milk diet to a diet that includes available forage (including hay) and access to concentrate fed to the mare.

## Supporting Information

S1 Table SIMPER analysis identifying the % contribution of each family to the Bray Curtis dissimilarity metric between time 1 and time 2. Similarity percentage analysis of the family differences between time 1 and time 2. The first column identifies the family explained by that row, the second column shows average Bray Curtis dissimilarity between time 1 and time 2 for that family, the third column shows the % dissimilarity explained by that family, the fourth column tallies the cumulative Bray Curtis dissimilarity metric for the family thus far represented in the table, and the last three columns show mean abundance at time 1, mean abundance at time 2, and change in mean abundance respectively.
(PDF)

S2 Table OTUs that were identified as being significantly different between time 1 and time 2 via Mann-Whitney U test. The first column provides OTU number, the second column shows taxonomic assignment, the third column provides FDR corrected P-value, and the final 2 columns provide mean proportion for time 1 and time 2 respectively.
(PDF)

S3 Table KEGG orthologs significantly increased and decreased > 2 fold between time 1 and time 2. The first column list increased KEGG orthologs and the secondond column list decreased KEGG orthologs.
(PDF)
