# Supplementary material for: Composition and Diversity of the Fecal Microbiome and Inferred Fecal Metagenome Does Not Predict Subsequent Pneumonia Caused by Rhodococcus equi in Foals
Source: PLoS One. 2015 Aug 25;10(8):e0136586. doi: 10.1371/journal.pone.0136586 (PMC4549325; doi:10.1371/journal.pone.0136586)
Supplement: S1 Table — Similarity percentage analysis of the family differences between time 1 and time 2. The first column identifies the family explained by that row, the second column shows average Bray Curtis dissimilarity between time 1 and time 2 for that family, the third column shows the % dissimilarity explained by that family, the fourth column tallies the cumulative Bray Curtis dissimilarity metric for the family thus far represented in the table, and the last three columns show mean abundance at time 1, mean abundance at time 2, and change in mean abundance respectively. (PDF) [file pone.0136586.s001.pdf]

Family	% Contribution	Cumulative %	Mean time 1	Mean time 2	Change in abundance with time
k__Bacteria;p__Firmicutes;c__Clostridia;o__Clostridiales; f__Ruminococcaceae	11.52	11.52	21.00%	22.70%	1.70%
k__Bacteria;p__Firmicutes;c__Clostridia;o__Clostridiales; f__Lachnospiraceae	10.63	22.15	18.40%	13.00%	-5.40%
k__Bacteria;p__Firmicutes;c__Clostridia;o__Clostridiales;f__	7.833	29.98	14.20%	15.50%	1.30%
k__Archaea;p__Euryarchaeota;c__Methanobacteria;o__Methanobacteriales; f__Methanobacteriaceae	6.694	36.68	2.76%	7.18%	4.42%
k__Bacteria;p__Bacteroidetes;c__Bacteroidia;o__Bacteroidales;f__	6.124	42.8	1.55%	6.00%	4.45%
k__Bacteria;p__Firmicutes;c__Bacilli;o__Bacillales; f__Planococcaceae	5.518	48.32	3.32%	2.65%	-0.67%
k__Bacteria;p__Firmicutes;c__Bacilli;o__Lactobacillales;f__Lactobacillaceae	4.825	53.15	4.98%	1.45%	-3.53%
k__Bacteria;p__Proteobacteria;c__Gammaproteobacteria;o__Pseudomonadales;f__Moraxellaceae	4.316	57.46	2.85%	1.32%	-1.53%
k__Bacteria;p__Bacteroidetes;c__Bacteroidia;o__Bacteroidales; f__Bacteroidaceae	3.806	61.27	3.75%	1.59%	-2.16%
k__Bacteria;p__Firmicutes;c__Clostridia;o__Clostridiales; f__Christensenellaceae	3.483	64.75	3.40%	2.71%	-0.69%
k__Bacteria;p__Firmicutes;c__Clostridia;o__Clostridiales; f__[Mogibacteriaceae]	2.996	67.75	2.64%	4.11%	1.47%
k__Bacteria;p__Firmicutes;c__Clostridia;o__Clostridiales;f__Clostridiaceae	2.574	70.32	2.02%	3.31%	1.29%
k__Bacteria;p__Verrucomicrobia;c__Verrucomicrobiae;o__Verrucomicrobiales; f__Verrucomicrobiaceae	2.489	72.81	1.87%	1.55%	-0.32%
k__Bacteria;p__Proteobacteria;c__Gammaproteobacteria;o__Enterobacteriales; f__Enterobacteriaceae	2.251	75.06	1.19%	1.16%	-0.03%
k__Bacteria;p__Verrucomicrobia;c__Verruco-5;o__WCHB1-41;f__RFP12	2.06	77.12	0.46%	1.97%	1.51%
k__Bacteria;p__Spirochaetes;c__Spirochaetes;o__Spirochaetales; f__Spirochaetaceae	1.877	79	0.40%	1.91%	1.51%
k__Bacteria;p__Firmicute	1.831	80.83	1.75%	0.87%	-0.88%

s;c__Bacilli;o__Lactobacillales;f__Streptococcaceae					
k__Bacteria;p__Bacteroidetes;c__Bacteroidia;o__Bacteroidales;f__Parapre	1.342	82.17	0.65%	1.20%	0.55%
k__Bacteria;p__Bacteroidetes;c__Bacteroidia;o__Bacteroidales;f__BS11	1.306	83.48	0.51%	1.07%	0.56%
k__Bacteria;p__Firmicutes;c__Clostridia;o__Clostridiales;f__Veillonellaceae	1.298	84.77	1.24%	0.74%	-0.50%
k__Bacteria;p__Firmicutes;c__Bacilli;o__Bacillales; f__Bacillaceae	1.27	86.04	0.86%	0.56%	-0.30%
k__Bacteria;p__Bacteroidetes;c__Bacteroidia;o__Bacteroidales; f__Porphyromonadaceae	0.9022	86.95	0.84%	0.24%	-0.60%
k__Bacteria;p__Actinobacteria;c__Coriobacteriia;o__Coriobacteriales; f__Coriobacteriaceae	0.8914	87.84	1.19%	0.85%	-0.34%
k__Bacteria;p__Firmicutes;c__Bacilli;o__Lactobacillales; f__Carnobacteriaceae	0.7014	88.54	0.60%	0.06%	-0.54%
k__Bacteria;p__Chlamydiae;c__Chlamydiia;o__Chlamydiales;f__Chlamydiaceae	0.6959	89.24	0.21%	0.50%	0.29%
k__Bacteria;p__Firmicutes;c__Clostridia;o__Clostridiales; f__Dehalobacteriaceae	0.6345	89.87	0.52%	0.63%	0.12%
k__Bacteria;p__Proteobacteria;c__Deltaproteobacteria;o__Desulfo	0.6073	90.48	0.58%	0.12%	-0.45%
k__Bacteria;p__WPS-2;c__;o__;f__	0.54	91.02	0.43%	0.09%	-0.34%
k__Bacteria;p__Bacteroidetes;c__Bacteroidia;o__Bacteroidales;f__[Odoribacteraceae]	0.5115	91.53	0.46%	0.09%	-0.37%
k__Bacteria;p__Actinobacteria;c__Actinobacteria; o__Actinomycetales; f__Micrococcaceae	0.5029	92.03	0.42%	0.09%	-0.33%
k__Bacteria;p__Firmicutes;c__Clostridia;o__Clostridiales;Other	0.4865	92.52	0.55%	0.27%	-0.29%
k__Bacteria;p__Fusobacteria;c__Fusobacteriia;o__Fusobacteriales;f__Fusobacteriaceae	0.4355	92.95	0.31%	0.21%	-0.10%
k__Bacteria;p__Bacteroidetes;c__Bacteroidia;o__Bacteroidales;f__S24-7	0.4073	93.36	0.12%	0.33%	0.21%
k__Bacteria;p__Firmicutes;c__Bacilli;o__Lactobacillales;f__Enterococcaceae	0.3939	93.75	0.22%	0.19%	-0.03%
k__Bacteria;p__Firmicutes;c__Clostridia;o__Clostridiales;f__Eubacteriaceae	0.3889	94.14	0.15%	0.36%	0.22%
k__Bacteria;p__Firmicutes;c__Clostridia;o__Clostri	0.2873	94.43	0.14%	0.14%	0.01%

diales;f__[Tissierellaceae]					
k__Bacteria;p__Firmicutes;c__Erysipelotrichi;o__Erysipelotrichales; f__Erysipelotrichaceae	0.2737	94.7	0.30%	0.19%	-0.11%
k__Bacteria;p__Firmicutes;c__Bacilli;o__Lactobacillales; f__Leuconostocaceae	0.2686	94.97	0.10%	0.16%	0.06%
k__Bacteria;p__Bacteroidetes;c__Bacteroidia;o__Bacteroidales; f__Rikenellaceae	0.2671	95.24	0.24%	0.04%	-0.19%
Unassigned;Other;Other;Other;Other	0.2656	95.51	0.23%	0.33%	0.10%
k__Bacteria;p__Spirochaetes;c__MVP-15;o__PL-11B10;f__	0.2652	95.77	0.09%	0.18%	0.09%
k__Bacteria;p__Proteobacteria;c__Epsilonproteobacteria;o__Campylobacterales; f__Campylobacteraceae	0.2623	96.03	0.22%	0.08%	-0.14%
k__Bacteria;p__Fibrobacteres;c__Fibrobacteria;o__Fibrobacterales; f__Fibrobacteraceae	0.2264	96.26	0.02%	0.21%	0.19%
k__Bacteria;p__Proteobacteria;c__Betaproteobacteria;o__Tremblayales;f__	0.2188	96.48	0.11%	0.12%	0.01%
k__Bacteria;p__Firmicutes;c__Bacilli;o__Bacillales; f__Staphylococcaceae	0.2187	96.7	0.20%	0.01%	-0.19%
k__Bacteria;p__Tenericutes;c__Mollicutes;o__RF39;f__	0.2133	96.91	0.23%	0.13%	-0.11%
k__Bacteria;p__Actinobacteria;c__Actinobacteria; o__Actinomycetales; f__Actinomycetaceae	0.2113	97.12	0.07%	0.19%	0.13%
k__Bacteria;p__Firmicutes;c__Clostridia;o__Clostridiales; f__Peptostreptococcaceae	0.1853	97.31	0.08%	0.11%	0.03%
k__Bacteria;p__Armatimonadetes;c__SJA-176;o__RB046;f__	0.1843	97.49	0.08%	0.15%	0.08%
k__Bacteria;p__Synergistetes;c__Synergistia;o__Synergistales;f__Synergistaceae	0.1799	97.67	0.09%	0.12%	0.03%
k__Archaea;p__Euryarchaeota;c__Methanomicrobia;o__Methanomicrobiales; f__Methanocorpusculaceae	0.1774	97.85	0.06%	0.14%	0.08%
k__Bacteria;p__Bacteroidetes;c__Bacteroidia;o__Bacteroidales;f__Pre	0.1415	97.99	0.07%	0.11%	0.04%
k__Bacteria;p__Firmicutes;c__Clostridia;o__Clostridiales;f__Peptococcaceae	0.1397	98.13	0.13%	0.08%	-0.05%

k__Bacteria;p__Bacteroidetes;c__Bacteroidia;o__Bacteroidales;f__RF16	0.129	98.26	0.05%	0.10%	0.06%
k__Bacteria;p__Proteobacteria;c__Alphaproteobacteria;o__RF32;f__	0.1204	98.38	0.10%	0.03%	-0.07%
k__Bacteria;p__Firmicutes;c__Bacilli;o__Lactobacillales;Other	0.1127	98.49	0.07%	0.05%	-0.01%
k__Bacteria;p__Proteobacteria;c__Alphaproteobacteria;o__;f__	0.1101	98.6	0.10%	0.01%	-0.08%
k__Bacteria;p__Proteobacteria;c__Epsilonproteobacteria;o__Campylobacterales; f__Helicobacteraceae	0.1076	98.71	0.05%	0.08%	0.03%
k__Bacteria;p__Planctomycetes;c__Planctomycetia;o__Pirellulales; f__Pirellulaceae	0.09007	98.8	0.06%	0.09%	0.03%
k__Bacteria;p__Tenericutes;c__RF3;o__ML615J-28;f__	0.08261	98.88	0.07%	0.02%	-0.04%
k__Bacteria;p__Actinobacteria;c__Actinobacteria; o__Actinomycetales; f__Corynebacteriaceae	0.06411	98.95	0.06%	0.01%	-0.04%
