# Supplementary material for: Composition and Diversity of the Fecal Microbiome and Inferred Fecal Metagenome Does Not Predict Subsequent Pneumonia Caused by Rhodococcus equi in Foals
Source: PLoS One. 2015 Aug 25;10(8):e0136586. doi: 10.1371/journal.pone.0136586 (PMC4549325; doi:10.1371/journal.pone.0136586)
Supplement: S2 Table — The first column provides OTU number, the second column shows taxonomic assignment, the third column provides FDR corrected P-value, and the final 2 columns provide mean proportion for time 1 and time 2 respectively. (PDF) [file pone.0136586.s002.pdf]

OTU Number	taxonomy	FDR corrected p-value	Time 1 mean	Time 2 mean
330566	k__Bacteria;p__Firmicutes;c__Clostridia;o__Clostridiales;f__Lachnospiraceae;g__Blautia;s__	1.88E-20	5.04E+01	2.39E-01
New.ReferenceOTU350	k__Bacteria;p__Firmicutes;c__Clostridia;o__Clostridiales;f__Ruminococcaceae;g__;s__	1.88E-20	5.63E-02	1.98E+01
4396426	k__Bacteria;p__Firmicutes;c__Clostridia;o__Clostridiales;f__Lachnospiraceae;g__;s__	2.10E-20	4.50E+01	3.28E-01
295023	k__Bacteria;p__Firmicutes;c__Clostridia;o__Clostridiales;f__Lachnospiraceae;g__Blautia;s__	2.96E-18	5.03E+01	1.06E+00
4472551	k__Bacteria;p__Firmicutes;c__Clostridia;o__Clostridiales;f__Lachnospiraceae;g__Blautia;s__producta	8.35E-18	5.46E+01	2.73E+00
326936	k__Bacteria;p__Firmicutes;c__Clostridia;o__Clostridiales;f__Lachnospiraceae;g__Blautia;s__	3.96E-17	5.82E+01	1.34E+00
3185810	k__Bacteria;p__Firmicutes;c__Clostridia;o__Clostridiales;f__;g__;s__	3.96E-17	7.35E+01	2.79E+00
529180	k__Bacteria;p__Firmicutes;c__Clostridia;o__Clostridiales;f__Lachnospiraceae;g__Coprococcus;s__	7.67E-17	5.33E+01	5.99E+00
287744	k__Bacteria;p__Firmicutes;c__Clostridia;o__Clostridiales;f__[Mogibacteriaceae];g__;s__	8.56E-17	2.17E+01	1.60E+00
293330	k__Bacteria;p__Firmicutes;c__Clostridia;o__Clostridiales;f__Lachnospiraceae;g__Blautia;s__	1.83E-16	1.12E+02	4.48E+00
182653	k__Bacteria;p__Firmicutes;c__Clostridia;o__Clostridiales;f__Lachnospiraceae;g__Dorea;s__	2.95E-16	1.66E+01	4.93E-01
183487	k__Bacteria;p__Firmicutes;c__Clostridia;o__Clostridiales;f__Lachnospiraceae;g__Blautia;s__producta	4.31E-16	2.06E+01	1.19E+00
4412103	k__Bacteria;p__Firmicutes;c__Clostridia;o__Clostridiales;f__Lachnospiraceae;g__Dorea;s__	4.74E-16	1.05E+01	8.21E-01
847728	k__Bacteria;p__Firmicutes;c__Clostridia;o__Clostridiales;f__Ruminococcaceae;g__Ruminococcus;s__	5.42E-16	3.42E+01	4.03E-01
4391326	k__Bacteria;p__Firmicutes;c__Clostridia;o__Clostridiales;f__Lachnospiraceae;g__Dorea;s__	6.73E-16	1.96E+01	1.18E+00
111098	k__Bacteria;p__Firmicutes;c__Clostridia;o__Clostridiales;f__Ruminococcaceae;g__;s__	6.75E-16	3.13E+00	3.49E+01
301305	k__Bacteria;p__Bacteroidetes;c__Bacteroidia;o__Bacteroidales;f__;g__;s__	7.68E-16	1.41E-02	2.74E+01
4451907	k__Bacteria;p__Firmicutes;c__Clostridia;o__Clostridiales;f__Lachnospiraceae	1.13E-15	1.29E+01	8.51E-01
4453773	k__Bacteria;p__Proteobacteria;c__Deltaproteobacteria;o__Desulfovibrionales;f__Desulfovibrionaceae;g__Desulfovibrio;s__	1.17E-15	4.18E+01	2.09E+00
4387469	k__Bacteria;p__Firmicutes;c__Clostridia;o__Clostridiales;f__Ruminococcaceae;g__;s__	1.36E-15	2.90E+01	1.19E-01
4443846	k__Bacteria;p__Firmicutes;c__Clostridia;o__Clostridiales;f__Lachnospiraceae	2.98E-15	3.26E+01	2.30E+00
4306262	k__Bacteria;p__Verrucomicrobia;c__Verrucomicrobiae;o__Verrucomicrobiales;f__Verrucomicrobiaceae;g__Akkermansia;s__muciniphila	2.98E-15	1.22E+02	4.20E+01
591439	k__Bacteria;p__Firmicutes;c__Clostridia;o__Clostridiales;f__[Mogibacteriaceae];g__;s__	4.30E-15	8.10E+00	1.94E-01
157772	k__Bacteria;p__Firmicutes;c__Clostridia;o__Clostridiales;f__Ruminococcaceae;g__Oscillospira;s__	4.30E-15	7.24E+00	8.96E-02
326476	k__Bacteria;p__Firmicutes;c__Clostridia;o__Clostridiales;f__Lachnospiraceae;g__;s__	6.35E-15	3.38E-01	1.84E+01
267605	k__Bacteria;p__Firmicutes;c__Clostridia;o__Clostridiales;f__Lachnospiraceae;g__;s__	7.69E-15	5.18E+00	5.22E+01
295127	k__Bacteria;p__Spirochaetes;c__Spirochaetes;o__Spirochaetales;f__Spirochaetaceae;g__Treponema;s__	8.11E-15	1.03E+00	2.27E+01
269509	k__Bacteria;p__Firmicutes;c__Bacilli;o__Lactobacillales;f__Streptococcaceae;g__Streptococcus;s__minor	1.06E-14	2.68E+01	1.57E+00
New.ReferenceOTU878	k__Bacteria;p__WPS-2;c__;o__;f__;g__;s__	1.17E-14	4.44E+01	1.06E+00
801844	k__Bacteria;p__Firmicutes;c__Clostridia;o__Clostridiales;f__;g__;s__	1.48E-14	2.00E+00	3.21E+01
New.ReferenceOTU5	k__Bacteria;p__Firmicutes;c__Clostridia;o__Clostridiales;f__Lachnospiraceae;g_	1.59E-14	1.03E+	4.48E-

98	_Blautia;s__producta		01	01
49406	k__Bacteria;p__Firmicutes;c__Clostridia;o__Clostridiales;f__;g__;s__	2.08E-14	2.62E+01	1.28E+00
721569	k__Bacteria;p__Firmicutes;c__Clostridia;o__Clostridiales;f__Ruminococcaceae;g__;s__	2.08E-14	1.81E+01	3.88E-01
4390863	k__Bacteria;p__Firmicutes;c__Clostridia;o__Clostridiales;f__;g__;s__	2.08E-14	1.60E+01	9.70E-01
25695	k__Bacteria;p__Firmicutes;c__Clostridia;o__Clostridiales;f__Clostridiaceae;g__Clostridium;s__	2.54E-14	1.45E+01	4.18E-01
4455257	k__Bacteria;p__Fusobacteria;c__Fusobacteriia;o__Fusobacteriales;f__Fusobacteriaceae;g__Fusobacterium;s__	3.34E-14	9.82E+00	2.39E-01
4450214	k__Bacteria;p__Firmicutes;c__Clostridia;o__Clostridiales;f__Ruminococcaceae;g__;s__	4.76E-14	3.26E+01	1.15E+00
New.ReferenceOTU904	k__Bacteria;p__Firmicutes;c__Clostridia;o__Clostridiales;f__Lachnospiraceae;g__;s__	8.41E-14	3.28E+00	2.81E+01
4404405	k__Bacteria;p__Firmicutes;c__Bacilli;o__Lactobacillales;f__Carnobacteriaceae;g__Desemzia;s__	1.22E-13	4.16E+01	3.51E+00
323903	k__Bacteria;p__Firmicutes;c__Clostridia;o__Clostridiales;f__Ruminococcaceae;g__;s__	1.24E-13	1.52E+00	1.73E+01
145009	k__Bacteria;p__Firmicutes;c__Bacilli;o__Lactobacillales;f__Streptococcaceae;g__Streptococcus;s__	1.65E-13	1.65E+00	2.05E+01
330252	k__Bacteria;p__Firmicutes;c__Clostridia;o__Clostridiales;f__Ruminococcaceae;g__;s__	2.73E-13	7.18E-01	9.16E+00
317004	k__Bacteria;p__Firmicutes;c__Clostridia;o__Clostridiales;f__;g__;s__	3.15E-13	1.84E+01	1.02E+02
258613	k__Bacteria;p__Firmicutes;c__Clostridia;o__Clostridiales;f__;g__;s__	3.47E-13	5.23E+00	3.47E+01
312227	k__Bacteria;p__Bacteroidetes;c__Bacteroidia;o__Bacteroidales;f__;g__;s__	3.53E-13	9.58E-01	1.06E+01
4459388	k__Bacteria;p__Firmicutes;c__Clostridia;o__Clostridiales;f__Lachnospiraceae;g__;s__	3.54E-13	1.22E+01	2.39E-01
314825	k__Bacteria;p__Bacteroidetes;c__Bacteroidia;o__Bacteroidales;f__;g__;s__	3.71E-13	1.07E+00	2.53E+01
538947	k__Bacteria;p__Firmicutes;c__Clostridia;o__Clostridiales;f__Lachnospiraceae;g__;s__	4.09E-13	8.61E+01	1.93E+01
1129180	k__Bacteria;p__Firmicutes;c__Clostridia;o__Clostridiales;f__;g__;s__	4.30E-13	6.76E-01	9.24E+00
4303852	k__Bacteria;p__Firmicutes;c__Clostridia;o__Clostridiales;f__Ruminococcaceae;g__;s__	4.51E-13	1.03E+00	1.07E+01
292062	k__Bacteria;p__Firmicutes;c__Clostridia;o__Clostridiales;f__Ruminococcaceae;g__;s__	4.51E-13	2.39E+00	1.47E+01
313495	k__Bacteria;p__Firmicutes;c__Clostridia;o__Clostridiales;f__Lachnospiraceae;g__Coprococcus;s__	5.02E-13	4.35E+01	6.16E+00
663226	k__Bacteria;p__Firmicutes;c__Clostridia;o__Clostridiales;f__Veillonellaceae;g__Anaerovibrio;s__	5.65E-13	3.09E+01	5.82E-01
319512	k__Bacteria;p__Firmicutes;c__Clostridia;o__Clostridiales;f__Ruminococcaceae;g__;s__	6.11E-13	1.96E+00	1.03E+01
296644	k__Bacteria;p__Bacteroidetes;c__Bacteroidia;o__Bacteroidales;f__;g__;s__	6.77E-13	1.12E+01	8.51E+01
273272	k__Bacteria;p__Firmicutes;c__Clostridia;o__Clostridiales;f__;g__;s__	7.33E-13	5.92E-01	1.27E+01
New.ReferenceOTU765	k__Bacteria;p__Bacteroidetes;c__Bacteroidia;o__Bacteroidales;f__;g__;s__	7.45E-13	1.13E-01	9.61E+00
563764	k__Bacteria;p__Firmicutes;c__Bacilli;o__Bacillales;f__Planococcaceae;g__;s__	7.99E-13	4.61E+01	4.57E+00
296811	k__Bacteria;p__Verrucomicrobia;c__Verruco-5;o__WCHB1-41;f__RFP12;g__;s__	8.65E-13	2.06E+00	1.07E+01
166515	k__Bacteria;p__Firmicutes;c__Clostridia;o__Clostridiales;f__Ruminococcaceae;g__;s__	9.17E-13	1.02E+01	0.00E+00
207816	k__Bacteria;p__Firmicutes;c__Clostridia;o__Clostridiales;f__Ruminococcaceae;g__;s__	9.51E-13	8.45E-01	1.09E+01
4364464	k__Bacteria;p__Firmicutes;c__Clostridia;o__Clostridiales;f__Lachnospiraceae;g__[Ruminococcus]	9.51E-13	1.21E+01	8.51E-01

441934	k__Bacteria;p__Firmicutes;c__Clostridia;o__Clostridiales;f__;g__;s__	1.02E-12	2.18E+00	1.31E+01
302906	k__Bacteria;p__Fibrobacteres;c__Fibrobacteria;o__Fibrobacterales;f__Fibrobacteraceae;g__Fibrobacter;s__succinogenes	1.64E-12	8.59E-01	1.52E+01
1132942	k__Bacteria;p__Firmicutes;c__Clostridia;o__Clostridiales;f__Ruminococcaceae;g__;s__	1.67E-12	6.38E+00	1.04E-01
312156	k__Bacteria;p__Firmicutes;c__Clostridia;o__Clostridiales;f__;g__;s__	1.85E-12	6.51E+00	1.64E-01
330992	k__Bacteria;p__Firmicutes;c__Clostridia;o__Clostridiales;f__Lachnospiraceae;g__;s__	1.85E-12	4.61E+00	4.87E+01
New.ReferenceOTU189	k__Bacteria;p__Bacteroidetes;c__Bacteroidia;o__Bacteroidales;f__S24-7;g__;s__	2.39E-12	0.00E+00	1.19E+01
4402903	k__Bacteria;p__Firmicutes;c__Clostridia;o__Clostridiales;f__Christensenellaceae;g__;s__	2.57E-12	5.23E+01	7.16E+00
174147	k__Bacteria;p__Firmicutes;c__Clostridia;o__Clostridiales;f__Ruminococcaceae;g__Ruminococcus;s__	2.60E-12	1.28E+01	1.49E-02
4331360	k__Bacteria;p__Firmicutes;c__Clostridia;o__Clostridiales;f__Lachnospiraceae;g__;s__	2.60E-12	9.32E+00	3.43E-01
335789	k__Bacteria;p__Firmicutes;c__Clostridia;o__Clostridiales;f__Lachnospiraceae;g__Blautia;s__	2.63E-12	3.69E+00	1.49E-02
661758	k__Bacteria;p__Spirochaetes;c__Spirochaetes;o__Spirochaetales;f__Spirochaetaceae;g__Treponema;s__	2.65E-12	8.76E+00	5.40E+01
New.ReferenceOTU461	k__Bacteria;p__Firmicutes;c__Clostridia;o__Clostridiales;f__;g__;s__	2.65E-12	6.54E+00	2.94E+01
339121	k__Bacteria;p__Firmicutes;c__Clostridia;o__Clostridiales;f__Ruminococcaceae;g__;s__	2.65E-12	1.14E+01	4.47E+01
109593	k__Bacteria;p__Firmicutes;c__Clostridia;o__Clostridiales;f__Lachnospiraceae;g__;s__	3.05E-12	9.15E-01	8.27E+00
97263	k__Bacteria;p__Firmicutes;c__Clostridia;o__Clostridiales;f__Ruminococcaceae;g__;s__	3.21E-12	1.05E+01	1.04E-01
337606	k__Bacteria;p__Bacteroidetes;c__Bacteroidia;o__Bacteroidales;f__Bacteroidaceae;g__BF311;s__	3.28E-12	1.17E+00	1.12E+01
4472174	k__Bacteria;p__Firmicutes;c__Clostridia;o__Clostridiales;f__Lachnospiraceae;g__[Ruminococcus];s__	3.54E-12	2.09E+01	1.43E+00
291938	k__Bacteria;p__Firmicutes;c__Clostridia;o__Clostridiales;f__Lachnospiraceae;g__;s__	5.62E-12	4.44E+01	1.41E+02
354566	k__Bacteria;p__Bacteroidetes;c__Bacteroidia;o__Bacteroidales;f__;g__;s__	7.31E-12	1.45E+00	1.57E+01
196462	k__Bacteria;p__Firmicutes;c__Clostridia;o__Clostridiales	7.59E-12	2.85E+01	3.24E+00
673084	k__Bacteria;p__Firmicutes;c__Clostridia;o__Clostridiales;f__[Mogibacteriaceae];g__;s__	7.94E-12	1.10E+01	1.19E+00
338757	k__Bacteria;p__Firmicutes;c__Bacilli;o__Lactobacillales;f__Lactobacillaceae;g__Lactobacillus;s__mucosae	8.78E-12	2.70E+01	6.21E+00
4437746	k__Bacteria;p__Firmicutes;c__Clostridia;o__Clostridiales;f__;g__;s__	1.04E-11	4.51E+00	8.96E-02
184497	k__Bacteria;p__Firmicutes;c__Clostridia;o__Clostridiales;f__;g__;s__	1.04E-11	6.86E+00	3.13E-01
350041	k__Bacteria;p__Firmicutes;c__Clostridia;o__Clostridiales;f__Lachnospiraceae;g__;s__	1.08E-11	8.93E+00	3.53E+01
1077751	k__Bacteria;p__Firmicutes;c__Clostridia;o__Clostridiales;f__;g__;s__	1.16E-11	8.07E+00	2.53E+01
607825	k__Bacteria;p__Firmicutes;c__Clostridia;o__Clostridiales;f__;g__;s__	1.26E-11	1.27E+00	9.75E+00
297662	k__Bacteria;p__Firmicutes;c__Clostridia;o__Clostridiales;f__;g__;s__	1.26E-11	6.70E+00	2.84E+01
303299	k__Bacteria;p__Bacteroidetes;c__Bacteroidia;o__Bacteroidales;f__;g__;s__	1.33E-11	1.34E+00	1.31E+01
314680	k__Bacteria;p__Firmicutes;c__Clostridia;o__Clostridiales;f__[Mogibacteriaceae];g__;s__	1.40E-11	2.15E+00	9.76E+00
345763	k__Bacteria;p__Firmicutes;c__Clostridia;o__Clostridiales;f__Ruminococcaceae;g__;s__	1.40E-11	1.47E+01	1.82E+00
268220	k__Bacteria;p__Firmicutes;c__Clostridia;o__Clostridiales;f__;g__;s__	1.48E-11	9.25E+00	2.24E-01

300401	k__Bacteria;p__Firmicutes;c__Clostridia;o__Clostridiales;f__;g__;s__	2.90E-11	7.44E+00	2.46E+01
1107755	k__Bacteria;p__Firmicutes;c__Clostridia;o__Clostridiales;f__;g__;s__	3.65E-11	4.87E+00	3.73E-01
185570	k__Bacteria;p__Firmicutes;c__Clostridia;o__Clostridiales;f__Lachnospiraceae;g__[Ruminococcus];s__gnavus	4.34E-11	1.20E+01	1.15E+00
359984	k__Bacteria;p__Firmicutes;c__Clostridia;o__Clostridiales;f__Ruminococcaceae;g__;s__	4.87E-11	2.02E+01	2.15E+00
533847	k__Bacteria;p__Firmicutes;c__Clostridia;o__Clostridiales;f__Ruminococcaceae;g__Oscillospira;s__	5.27E-11	5.46E+00	0.00E+00
230232	k__Bacteria;p__Firmicutes;c__Clostridia;o__Clostridiales;f__Lachnospiraceae;g__Dorea;s__	6.58E-11	2.92E+01	6.07E+00
298659	k__Bacteria;p__Firmicutes;c__Clostridia;o__Clostridiales;f__;g__;s__	6.63E-11	1.89E+01	5.88E+01
4404401	k__Bacteria;p__Firmicutes;c__Bacilli;o__Bacillales;f__Staphylococcaceae;g__Jeotgalicoccus;s__	6.92E-11	7.32E+00	4.48E-02
305056	k__Bacteria;p__Firmicutes;c__Clostridia;o__Clostridiales;f__Ruminococcaceae;g__Ruminococcus;s__	6.99E-11	8.59E-01	1.39E+01
291085	k__Bacteria;p__Verrucomicrobia;c__Verruco-5;o__WCHB1-41;f__RFP12;g__;s__	7.34E-11	5.10E+00	2.43E+01
4435400	k__Bacteria;p__Firmicutes;c__Clostridia;o__Clostridiales;f__Lachnospiraceae	8.03E-11	6.62E+00	1.09E+00
355930	k__Bacteria;p__Bacteroidetes;c__Bacteroidia;o__Bacteroidales;f__;g__;s__	9.62E-11	1.40E+01	6.28E+01
New.ReferenceOTU923	k__Bacteria;p__Firmicutes;c__Clostridia;o__Clostridiales;f__;g__;s__	1.01E-10	9.06E+00	8.96E-02
290762	k__Bacteria;p__Firmicutes;c__Clostridia;o__Clostridiales;f__Lachnospiraceae;g__Coprococcus;s__	1.34E-10	9.06E+00	1.04E+00
174033	k__Bacteria;p__Firmicutes;c__Clostridia;o__Clostridiales;f__;g__;s__	1.37E-10	2.00E+00	1.19E-01
295539	k__Bacteria;p__Firmicutes;c__Clostridia;o__Clostridiales;f__Lachnospiraceae;g__;s__	1.41E-10	1.52E+00	1.50E+01
191458	k__Bacteria;p__Firmicutes;c__Bacilli;o__Lactobacillales;f__Streptococcaceae;g__Streptococcus;s__	1.45E-10	1.56E+01	6.42E-01
188348	k__Bacteria;p__Firmicutes;c__Clostridia;o__Clostridiales;f__Ruminococcaceae;g__;s__	1.54E-10	5.28E+00	8.96E-02
288583	k__Bacteria;p__Firmicutes;c__Clostridia;o__Clostridiales;f__Ruminococcaceae;g__;s__	1.54E-10	5.63E-01	7.87E+00
4473883	k__Bacteria;p__Firmicutes;c__Bacilli;o__Lactobacillales;f__Streptococcaceae;g__Streptococcus;s__	1.69E-10	1.96E+01	3.21E+00
303269	k__Bacteria;p__Firmicutes;c__Clostridia;o__Clostridiales;f__;g__;s__	1.72E-10	7.90E+00	4.18E-01
739387	k__Bacteria;p__Firmicutes;c__Clostridia;o__Clostridiales;f__;g__;s__	2.03E-10	1.63E+01	7.61E-01
294205	k__Bacteria;p__Verrucomicrobia;c__Verruco-5;o__WCHB1-41;f__RFP12;g__;s__	2.03E-10	6.93E+00	2.61E+01
292150	k__Bacteria;p__Bacteroidetes;c__Bacteroidia;o__Bacteroidales;f__;g__;s__	2.07E-10	1.83E-01	7.37E+00
4408801	k__Bacteria;p__Firmicutes;c__Clostridia;o__Clostridiales;f__Ruminococcaceae;g__Oscillospira;s__	2.10E-10	7.39E+00	1.19E-01
352840	k__Bacteria;p__Firmicutes;c__Clostridia;o__Clostridiales;f__Lachnospiraceae;g__;s__	2.11E-10	2.68E+00	2.68E+01
287578	k__Bacteria;p__Verrucomicrobia;c__Verruco-5;o__WCHB1-41;f__RFP12;g__;s__	2.11E-10	2.30E+00	1.22E+01
187338	k__Bacteria;p__Firmicutes;c__Clostridia;o__Clostridiales;f__Lachnospiraceae;g__Dorea;s__	2.18E-10	3.41E+00	2.09E-01
324511	k__Bacteria;p__Spirochaetes;c__Spirochaetes;o__Spirochaetales;f__Spirochaetaceae;g__Treponema;s__	2.18E-10	1.99E+00	1.28E+01
851733	k__Bacteria;p__Firmicutes;c__Bacilli;o__Lactobacillales;f__Lactobacillaceae;g__Lactobacillus;s__	2.18E-10	1.92E+02	3.00E+01
298695	k__Bacteria;p__Firmicutes;c__Clostridia;o__Clostridiales;f__Ruminococcaceae;g__;s__	2.24E-10	2.39E-01	5.58E+00
185802	k__Bacteria;p__Firmicutes;c__Clostridia;o__Clostridiales;f__Ruminococcaceae;g__;s__	2.70E-10	3.54E+01	1.00E+01

109491	k__Bacteria;p__Firmicutes;c__Clostridia;o__Clostridiales;f__Ruminococcaceae;g__;s__	3.08E-10	5.51E+01	1.89E+02
1105016	k__Bacteria;p__Firmicutes;c__Clostridia;o__Clostridiales;f__Ruminococcaceae;g__;s__	3.31E-10	1.15E+01	1.33E+00
293210	k__Bacteria;p__Bacteroidetes;c__Bacteroidia;o__Bacteroidales;f__[Paraprevotellaceae];g__;s__	3.31E-10	1.13E+01	4.20E+01
New.ReferenceOTU841	k__Bacteria;p__Firmicutes;c__Clostridia;o__Clostridiales;f__;g__;s__	3.56E-10	9.30E-01	4.76E+00
184534	k__Bacteria;p__Firmicutes;c__Clostridia;o__Clostridiales;f__Christensenellaceae;g__;s__	3.73E-10	4.57E+01	4.85E+00
292738	k__Bacteria;p__Bacteroidetes;c__Bacteroidia;o__Bacteroidales;f__BS11;g__;s__	3.73E-10	6.55E+00	3.13E+01
New.ReferenceOTU154	k__Bacteria;p__Firmicutes;c__Clostridia;o__Clostridiales;f__;g__;s__	3.73E-10	5.92E+00	1.06E+00
338781	k__Bacteria;p__Firmicutes;c__Clostridia;o__Clostridiales;f__Ruminococcaceae;g__;s__	3.73E-10	7.55E+00	2.76E+01
4439469	k__Bacteria;p__Firmicutes;c__Clostridia;o__Clostridiales;f__Ruminococcaceae;g__;s__	3.73E-10	1.43E+01	2.33E+00
New.ReferenceOTU639	k__Bacteria;p__Chlamydiae;c__Chlamydiia;o__Chlamydiales;f__Chlamydiaceae;g__;s__	3.77E-10	3.75E+00	3.92E+01
155884	k__Bacteria;p__Firmicutes;c__Clostridia;o__Clostridiales;f__Ruminococcaceae;g__Ruminococcus;s__	4.35E-10	2.54E-01	3.96E+00
672440	k__Bacteria;p__Firmicutes;c__Clostridia;o__Clostridiales;f__;g__;s__	4.40E-10	3.85E+00	1.85E+01
253380	k__Bacteria;p__Firmicutes;c__Clostridia;o__Clostridiales;f__Lachnospiraceae;g__Dorea;s__	4.43E-10	6.40E+01	1.27E+01
4299396	k__Bacteria;p__Firmicutes;c__Clostridia;o__Clostridiales;f__Ruminococcaceae;g__;s__	4.43E-10	1.61E+01	5.22E+01
170462	k__Bacteria;p__Firmicutes;c__Clostridia;o__Clostridiales;f__Lachnospiraceae;g__[Ruminococcus];s__	4.61E-10	4.70E+00	0.00E+00
New.ReferenceOTU689	k__Bacteria;p__Firmicutes;c__Clostridia;o__Clostridiales;f__;g__;s__	4.86E-10	2.82E+00	1.16E+01
3327894	k__Bacteria;p__Bacteroidetes;c__Bacteroidia;o__Bacteroidales;f__Bacteroidaceae;g__Bacteroides;s__uniformis	5.15E-10	8.55E+00	4.03E-01
109634	k__Bacteria;p__Firmicutes;c__Clostridia;o__Clostridiales;f__[Mogibacteriaceae];g__Mogibacterium;s__	5.78E-10	8.13E+00	4.24E+01
251970	k__Bacteria;p__Firmicutes;c__Clostridia;o__Clostridiales;f__Ruminococcaceae;g__;s__	6.52E-10	1.08E+01	3.31E+01
465433	k__Bacteria;p__Bacteroidetes;c__Bacteroidia;o__Bacteroidales;f__Rikenellaceae;g__;s__	6.70E-10	6.75E+00	2.39E-01
85903	k__Bacteria;p__Firmicutes;c__Clostridia;o__Clostridiales;f__Ruminococcaceae;g__;s__	6.70E-10	9.04E+00	1.70E+00
270676	k__Bacteria;p__Firmicutes;c__Clostridia;o__Clostridiales;f__Lachnospiraceae;g__;s__	6.78E-10	4.23E+00	1.84E+01
258389	k__Bacteria;p__Firmicutes;c__Clostridia;o__Clostridiales;f__Ruminococcaceae;g__;s__	6.92E-10	4.61E+00	1.93E+01
New.ReferenceOTU738	k__Bacteria;p__Verrucomicrobia;c__Verruco-5;o__WCHB1-41;f__RFP12;g__;s__	7.05E-10	9.72E-01	4.82E+00
294182	k__Bacteria;p__Bacteroidetes;c__Bacteroidia;o__Bacteroidales;f__;g__;s__	7.05E-10	2.68E-01	6.54E+00
289474	k__Bacteria;p__Firmicutes;c__Clostridia;o__Clostridiales;f__Ruminococcaceae;g__;s__	7.05E-10	1.97E-01	3.34E+00
458550	k__Bacteria;p__Firmicutes;c__Clostridia;o__Clostridiales;f__;g__;s__	8.44E-10	3.94E-01	5.84E+00
298279	k__Bacteria;p__Firmicutes;c__Clostridia;o__Clostridiales;f__;g__;s__	8.63E-10	7.46E-01	4.13E+00
New.ReferenceOTU38	k__Bacteria;p__Firmicutes;c__Clostridia;o__Clostridiales;f__Ruminococcaceae;g__;s__	1.12E-09	5.21E-01	4.85E+00
New.ReferenceOTU246	k__Bacteria;p__Firmicutes;c__Clostridia;o__Clostridiales;f__;g__;s__	1.14E-09	6.15E+00	1.49E-01
New.ReferenceOTU102	k__Bacteria;p__Firmicutes;c__Clostridia;o__Clostridiales;f__Christensenellaceae;g__;s__	1.14E-09	1.41E-01	2.57E+00
New.ReferenceOTU556	k__Bacteria;p__Firmicutes;c__Clostridia;o__Clostridiales;f__;g__;s__	1.19E-09	2.06E+00	7.49E+00

4441855	k__Bacteria;p__Firmicutes;c__Bacilli;o__Lactobacillales;f__Streptococcaceae;g__Streptococcus;s__	1.25E-09	8.83E+00	1.25E+00
589903	k__Bacteria;p__Firmicutes;c__Clostridia;o__Clostridiales;f__;g__;s__	1.25E-09	1.41E-02	5.57E+00
4446740	k__Bacteria;p__Firmicutes;c__Clostridia;o__Clostridiales;f__Ruminococcaceae;g__;s__	1.34E-09	1.62E+01	5.58E+01
262024	k__Bacteria;p__Firmicutes;c__Clostridia;o__Clostridiales;f__Ruminococcaceae	1.40E-09	1.04E+00	4.28E+00
110492	k__Bacteria;p__Firmicutes;c__Clostridia;o__Clostridiales;f__;g__;s__	1.43E-09	2.94E+01	8.59E+01
4476773	k__Bacteria;p__Firmicutes;c__Clostridia;o__Clostridiales;f__;g__;s__	1.45E-09	2.52E+01	4.99E+00
New.ReferenceOTU516	k__Bacteria;p__Spirochaetes;c__Spirochaetes;o__Spirochaetales;f__Spirochaetaceae;g__Treponema;s__	1.65E-09	6.62E-01	5.48E+00
236487	k__Bacteria;p__Bacteroidetes;c__Bacteroidia;o__Bacteroidales;f__S24-7;g__;s__	1.74E-09	0.00E+00	6.96E+00
104130	k__Archaea;p__Euryarchaeota;c__Methanobacteria;o__Methanobacteriales;f__Methanobacteriaceae;g__Methanobrevibacter;s__	1.74E-09	3.94E-01	3.06E+00
166689	k__Bacteria;p__Firmicutes;c__Clostridia;o__Clostridiales;f__Ruminococcaceae;g__;s__	1.75E-09	2.55E+00	5.97E-02
298621	k__Bacteria;p__Firmicutes;c__Clostridia;o__Clostridiales;f__Veillonellaceae;g__;s__	1.75E-09	1.15E+00	1.14E+01
350523	k__Bacteria;p__Firmicutes;c__Clostridia;o__Clostridiales;f__Lachnospiraceae;g__;s__	2.18E-09	4.76E+00	1.64E-01
54563	k__Bacteria;p__Firmicutes;c__Clostridia;o__Clostridiales;f__Lachnospiraceae;g__[Ruminococcus];s__gnavus	2.30E-09	1.19E+01	1.60E+00
182044	k__Bacteria;p__Firmicutes;c__Clostridia;o__Clostridiales;f__Ruminococcaceae;g__;s__	2.38E-09	4.39E+00	5.97E-01
353696	k__Bacteria;p__Firmicutes;c__Clostridia;o__Clostridiales;f__Christensenellaceae;g__;s__	2.47E-09	7.62E+00	5.21E+00
295822	k__Bacteria;p__Verrucomicrobia;c__Verruco-5;o__WCHB1-41;f__RFP12;g__;s__	2.71E-09	2.42E+00	1.11E+01
301719	k__Bacteria;p__Verrucomicrobia;c__Verruco-5;o__WCHB1-41;f__RFP12;g__;s__	2.79E-09	9.44E-01	4.10E+00
4451049	k__Bacteria;p__Firmicutes;c__Clostridia;o__Clostridiales;f__Christensenellaceae;g__;s__	2.85E-09	2.14E+01	1.12E+00
350502	k__Bacteria;p__Firmicutes;c__Clostridia;o__Clostridiales;f__Ruminococcaceae;g__;s__	3.09E-09	4.39E+00	1.45E+01
111019	k__Bacteria;p__Firmicutes;c__Clostridia;o__Clostridiales;f__Ruminococcaceae;g__;s__	3.16E-09	4.46E+00	1.94E+01
187470	k__Bacteria;p__Firmicutes;c__Clostridia;o__Clostridiales;f__Lachnospiraceae;g__Dorea;s__	3.82E-09	6.90E+00	1.18E+00
4465124	k__Bacteria;p__Firmicutes;c__Clostridia;o__Clostridiales;f__Ruminococcaceae;g__;s__	4.50E-09	3.27E+01	2.22E+00
237444	k__Bacteria;p__Firmicutes;c__Bacilli;o__Lactobacillales;f__Streptococcaceae;g__Streptococcus;s__	4.70E-09	2.19E+01	3.58E+00
4315471	k__Bacteria;p__Firmicutes;c__Clostridia;o__Clostridiales;f__Ruminococcaceae;g__Ruminococcus;s__	4.70E-09	3.42E+00	2.23E+01
110136	k__Bacteria;p__Firmicutes;c__Clostridia;o__Clostridiales;f__Dehalobacteriaceae;g__Dehalobacterium;s__	4.70E-09	6.48E-01	4.10E+00
25005	k__Bacteria;p__Firmicutes;c__Clostridia;o__Clostridiales;f__Lachnospiraceae;g__Roseburia;s__	4.93E-09	1.14E+01	1.36E+00
350363	k__Bacteria;p__Firmicutes;c__Clostridia;o__Clostridiales;f__Lachnospiraceae;g__;s__	5.62E-09	9.44E-01	4.70E+00
4347520	k__Bacteria;p__Firmicutes;c__Clostridia;o__Clostridiales;f__;g__;s__	5.62E-09	4.00E+00	4.48E-02
16204	k__Bacteria;p__Firmicutes;c__Clostridia;o__Clostridiales;f__Clostridiaceae;g__Sarcina;s__	6.02E-09	7.10E+01	1.94E+02
3600504	k__Bacteria;p__Bacteroidetes;c__Bacteroidia;o__Bacteroidales;f__Bacteroidaceae;g__Bacteroides;s__	6.04E-09	7.80E+01	2.15E+01
289929	k__Bacteria;p__Firmicutes;c__Clostridia;o__Clostridiales;f__Lachnospiraceae;g__;s__	6.05E-09	3.76E+00	1.08E+01
300297	k__Bacteria;p__Firmicutes;c__Clostridia;o__Clostridiales;f__Lachnospiraceae;g__Dorea;s__	6.17E-09	4.10E+00	3.88E-01

4438608	k__Bacteria;p__Firmicutes;c__Bacilli;o__Bacillales;f__Planococcaceae;g__Solibacillus;s__	7.03E-09	5.09E+01	1.34E+01
111024	k__Bacteria;p__Firmicutes;c__Clostridia;o__Clostridiales;f__;g__;s__	7.56E-09	1.04E+00	6.87E+00
291292	k__Bacteria;p__Verrucomicrobia;c__Verruco-5;o__WCHB1-41;f__RFP12;g__;s__	7.66E-09	4.37E-01	3.72E+00
4393532	k__Bacteria;p__Actinobacteria;c__Coriobacteriia;o__Coriobacteriales;f__Coriobacteriaceae;g__Eggerthella;s__lenta	8.03E-09	9.92E+00	3.07E+00
294812	k__Bacteria;p__Firmicutes;c__Clostridia;o__Clostridiales;f__Ruminococcaceae;g__;s__	8.07E-09	9.80E+00	4.93E-01
311293	k__Bacteria;p__Firmicutes;c__Clostridia;o__Clostridiales;f__Lachnospiraceae;g__;s__	8.75E-09	5.86E+00	3.28E-01
294229	k__Bacteria;p__Firmicutes;c__Clostridia;o__Clostridiales;f__;g__;s__	8.90E-09	5.21E-01	2.88E+00
4466707	k__Bacteria;p__Firmicutes;c__Clostridia;o__Clostridiales;f__Lachnospiraceae;g__Coprococcus;s__	8.92E-09	6.86E+00	1.07E+00
327491	k__Bacteria;p__Firmicutes;c__Clostridia;o__Clostridiales;f__Christensenellaceae;g__;s__	9.44E-09	4.65E+00	1.47E+01
308086	k__Bacteria;p__Firmicutes;c__Clostridia;o__Clostridiales;f__Christensenellaceae;g__;s__	9.83E-09	1.27E-01	2.39E+00
1667433	k__Bacteria;p__Firmicutes;c__Clostridia;o__Clostridiales;f__Lachnospiraceae;g__Dorea;s__	1.03E-08	7.85E+00	2.69E-01
183207	k__Bacteria;p__Firmicutes;c__Clostridia;o__Clostridiales;f__Lachnospiraceae;g__[Ruminococcus];s__	1.08E-08	4.72E+00	1.04E-01
New.ReferenceOTU790	k__Bacteria;p__Bacteroidetes;c__Bacteroidia;o__Bacteroidales;f__Bacteroidaceae;g__Bacteroides;s__	1.09E-08	1.90E+00	1.64E-01
287400	k__Bacteria;p__Spirochaetes;c__Spirochaetes;o__Spirochaetales;f__Spirochaetaceae;g__Treponema;s__	1.11E-08	4.08E-01	2.67E+00
312588	k__Bacteria;p__Firmicutes;c__Clostridia;o__Clostridiales;f__Lachnospiraceae;g__;s__	1.11E-08	1.83E-01	1.73E+00
3486634	k__Bacteria;p__Bacteroidetes;c__Bacteroidia;o__Bacteroidales;f__Bacteroidaceae;g__Bacteroides;s__uniformis	1.11E-08	4.30E+00	1.21E+00
297496	k__Bacteria;p__Firmicutes;c__Clostridia;o__Clostridiales;f__Ruminococcaceae;g__;s__	1.15E-08	4.99E+00	2.05E+01
550631	k__Bacteria;p__Firmicutes;c__Clostridia;o__Clostridiales;f__;g__;s__	1.16E-08	1.83E-01	1.48E+00
312882	k__Bacteria;p__Firmicutes;c__Clostridia;o__Clostridiales	1.46E-08	5.24E+00	6.57E-01
320072	k__Bacteria;p__Firmicutes;c__Clostridia;o__Clostridiales;f__Ruminococcaceae;g__;s__	1.51E-08	5.94E+00	8.10E+00
259357	k__Bacteria;p__Firmicutes;c__Clostridia;o__Clostridiales;f__Lachnospiraceae;g__Blautia;s__producta	1.72E-08	3.42E+00	2.99E-01
1046979	k__Bacteria;p__Firmicutes;c__Clostridia;o__Clostridiales;f__Ruminococcaceae;g__;s__	1.72E-08	1.55E-01	3.25E+00
258980	k__Bacteria;p__Firmicutes;c__Clostridia;o__Clostridiales;f__;g__;s__	1.74E-08	3.56E+00	5.97E-02
New.ReferenceOTU363	k__Bacteria;p__Spirochaetes;c__Spirochaetes;o__Spirochaetales;f__Spirochaetaceae;g__Treponema;s__	1.77E-08	4.87E+00	1.34E-01
349123	k__Bacteria;p__Firmicutes;c__Clostridia;o__Clostridiales;f__Christensenellaceae;g__;s__	1.97E-08	4.11E+00	1.64E-01
199451	k__Bacteria;p__Firmicutes;c__Clostridia;o__Clostridiales;f__Lachnospiraceae;g__Blautia;s__producta	1.99E-08	1.76E+00	1.04E-01
330276	k__Bacteria;p__Bacteroidetes;c__Bacteroidia;o__Bacteroidales;f__;g__;s__	2.03E-08	6.76E-01	9.61E+00
294860	k__Bacteria;p__Firmicutes;c__Clostridia;o__Clostridiales;f__[Mogibacteriaceae];g__;s__	2.05E-08	9.58E-01	4.61E+00
594307	k__Bacteria;p__WPS-2;c__;o__;f__;g__;s__	2.17E-08	3.38E-01	4.21E+00
339532	k__Bacteria;p__Actinobacteria;c__Actinobacteria;o__Bifidobacteriales;f__Bifidobacteriaceae;g__;s__	2.17E-08	4.75E+00	4.18E-01
1951283	k__Bacteria;p__Firmicutes;c__Bacilli;o__Bacillales;f__Planococcaceae;g__;s__	2.19E-08	2.27E+01	1.78E+00
310611	k__Bacteria;p__Firmicutes;c__Clostridia;o__Clostridiales;f__;g__;s__	2.19E-08	2.79E+00	8.87E+00

New.ReferenceOTU82	k__Bacteria;p__Spirochaetes;c__Spirochaetes;o__Spirochaetales;f__Spirochaetaceae;g__Treponema;s__	2.21E-08	1.10E+01	3.87E+01
194215	k__Bacteria;p__Firmicutes;c__Clostridia;o__Clostridiales;f__Ruminococcaceae;g__;s__	2.24E-08	1.58E+01	1.42E+00
336983	k__Bacteria;p__Firmicutes;c__Clostridia;o__Clostridiales;f__Lachnospiraceae;g__Blautia;s__	2.24E-08	4.17E+00	7.46E-02
New.ReferenceOTU895	k__Archaea;p__Euryarchaeota;c__Methanobacteria;o__Methanobacteriales;f__Methanobacteriaceae;g__Methanobrevibacter;s__	2.26E-08	1.15E+01	3.75E+01
352529	k__Bacteria;p__Firmicutes;c__Clostridia;o__Clostridiales;f__Lachnospiraceae;g__[Ruminococcus];s__torques	2.29E-08	6.92E+00	6.72E-01
175336	k__Bacteria;p__Firmicutes;c__Clostridia;o__Clostridiales;f__Ruminococcaceae;g__Oscillospira;s__	2.33E-08	6.08E+00	2.99E-02
295136	k__Bacteria;p__Verrucomicrobia;c__Verruco-5;o__WCHB1-41;f__RFP12;g__;s__	2.33E-08	2.66E+00	7.61E+00
New.ReferenceOTU709	k__Bacteria;p__Firmicutes;c__Clostridia;o__Clostridiales;f__;g__;s__	2.38E-08	2.39E-01	2.97E+00
215731	k__Bacteria;p__Firmicutes;c__Clostridia;o__Clostridiales;f__;g__;s__	2.45E-08	1.76E+00	1.47E+01
289966	k__Bacteria;p__Firmicutes;c__Clostridia;o__Clostridiales;f__[Mogibacteriaceae];g__;s__	2.45E-08	1.51E+00	6.52E+00
4446053	k__Bacteria;p__Proteobacteria;c__Alphaproteobacteria;o__;f__;g__;s__	2.51E-08	7.66E+00	5.97E-02
New.ReferenceOTU125	k__Bacteria;p__Verrucomicrobia;c__Verruco-5;o__WCHB1-41;f__RFP12;g__;s__	2.60E-08	1.55E-01	3.61E+00
183849	k__Bacteria;p__Firmicutes;c__Clostridia;o__Clostridiales;f__Lachnospiraceae;g__Blautia;s__producta	2.84E-08	3.20E+00	3.28E-01
1751298	k__Bacteria;p__Firmicutes;c__Clostridia;o__Clostridiales;f__Lachnospiraceae;g__;s__	2.95E-08	6.25E+00	1.49E-02
298517	k__Bacteria;p__Firmicutes;c__Clostridia;o__Clostridiales;f__Ruminococcaceae;g__;s__	3.05E-08	3.10E-01	3.42E+00
318204	k__Bacteria;p__Firmicutes;c__Clostridia;o__Clostridiales;f__;g__;s__	3.06E-08	5.28E+00	1.35E+01
4360363	k__Bacteria;p__Firmicutes;c__Clostridia;o__Clostridiales;f__;g__;s__	3.23E-08	4.68E+00	1.30E+01
302525	k__Bacteria;p__Firmicutes;c__Clostridia;o__Clostridiales;f__;g__;s__	3.37E-08	1.13E+01	4.03E-01
4307122	k__Bacteria;p__Bacteroidetes;c__Bacteroidia;o__Bacteroidales;f__[Odoribacteraceae];g__Odoribacter;s__	3.47E-08	1.01E+01	1.84E+00
180577	k__Bacteria;p__Firmicutes;c__Clostridia;o__Clostridiales;f__Lachnospiraceae;g__Dorea;s__	3.47E-08	1.99E+00	5.97E-02
291303	k__Bacteria;p__Firmicutes;c__Clostridia;o__Clostridiales;f__Lachnospiraceae;g__;s__	3.51E-08	5.63E-02	1.36E+00
4476780	k__Bacteria;p__Bacteroidetes;c__Bacteroidia;o__Bacteroidales;f__Rikenellaceae;g__;s__	3.61E-08	5.72E+00	1.61E+00
New.ReferenceOTU478	k__Bacteria;p__Bacteroidetes;c__Bacteroidia;o__Bacteroidales;f__[Odoribacteraceae];g__Odoribacter;s__	3.75E-08	1.08E+01	3.00E+00
512522	k__Bacteria;p__Firmicutes;c__Clostridia;o__Clostridiales;f__;g__;s__	3.86E-08	4.76E+00	1.28E+01
178839	k__Bacteria;p__Firmicutes;c__Clostridia;o__Clostridiales;f__[Mogibacteriaceae];g__;s__	4.06E-08	4.68E+00	4.48E-02
New.ReferenceOTU722	k__Bacteria;p__Bacteroidetes;c__Bacteroidia;o__Bacteroidales;f__;g__;s__	4.38E-08	1.00E+00	6.45E+00
301621	k__Bacteria;p__Verrucomicrobia;c__Verruco-5;o__WCHB1-41;f__RFP12;g__;s__	4.38E-08	2.24E+00	9.84E+00
301994	k__Bacteria;p__Bacteroidetes;c__Bacteroidia;o__Bacteroidales;f__;g__;s__	4.51E-08	2.06E+00	7.42E+00
300123	k__Bacteria;p__Firmicutes;c__Clostridia;o__Clostridiales;f__Lachnospiraceae;g__Coprococcus;s__	4.51E-08	2.82E-02	6.61E+00
178712	k__Bacteria;p__Firmicutes;c__Clostridia;o__Clostridiales;f__;g__;s__	5.40E-08	7.03E+01	2.31E+01
291133	k__Bacteria;p__Verrucomicrobia;c__Verruco-5;o__WCHB1-41;f__RFP12;g__;s__	5.40E-08	2.68E-01	1.69E+00
110847	k__Bacteria;p__Firmicutes;c__Clostridia;o__Clostridiales;f__Christensenellaceae;g__;s__	5.49E-08	5.73E+00	2.91E+01

4423553	k__Bacteria;p__Firmicutes;c__Clostridia;o__Clostridiales;f__Ruminococcaceae;g__;s__	5.52E-08	1.80E+01	5.46E+00
New.ReferenceOTU767	k__Bacteria;p__Firmicutes;c__Clostridia;o__Clostridiales;f__Ruminococcaceae;g__;s__	5.52E-08	1.80E+00	7.97E+00
295856	k__Bacteria;p__Firmicutes;c__Clostridia;o__Clostridiales;f__Ruminococcaceae;g__;s__	5.55E-08	8.45E-02	1.30E+00
510286	k__Bacteria;p__Firmicutes;c__Clostridia;o__Clostridiales;f__Lachnospiraceae;g__;s__	5.71E-08	1.49E+00	7.46E-02
3439403	k__Bacteria;p__Bacteroidetes;c__Bacteroidia;o__Bacteroidales;f__Bacteroidaceae;g__Bacteroides;s__	6.00E-08	1.01E+01	2.58E+00
196333	k__Bacteria;p__Firmicutes;c__Clostridia;o__Clostridiales;f__Lachnospiraceae;g__Blautia;s__producta	6.14E-08	1.04E+00	1.49E-02
291743	k__Bacteria;p__Firmicutes;c__Clostridia;o__Clostridiales;f__;g__;s__	6.43E-08	5.28E+00	2.31E+01
194597	k__Bacteria;p__Firmicutes;c__Clostridia;o__Clostridiales;f__;g__;s__	6.89E-08	4.52E+00	0.00E+00
339988	k__Bacteria;p__Firmicutes;c__Clostridia;o__Clostridiales;f__Ruminococcaceae;g__;s__	7.24E-08	1.11E+01	3.50E+01
New.ReferenceOTU215	k__Bacteria;p__Firmicutes;c__Clostridia;o__Clostridiales;f__Ruminococcaceae;g__Ruminococcus;s__	7.61E-08	1.07E+00	1.36E+01
355922	k__Bacteria;p__Firmicutes;c__Clostridia;o__Clostridiales;f__Ruminococcaceae;g__;s__	7.61E-08	4.23E-01	2.03E+00
349093	k__Bacteria;p__Firmicutes;c__Clostridia;o__Clostridiales;f__;g__;s__	7.66E-08	1.18E+00	5.04E+00
New.ReferenceOTU947	k__Bacteria;p__Firmicutes;c__Clostridia;o__Clostridiales;f__;g__;s__	7.80E-08	2.15E+00	1.04E-01
194584	k__Bacteria;p__Firmicutes;c__Clostridia;o__Clostridiales;f__Ruminococcaceae;g__;s__	7.92E-08	6.77E+00	1.45E+00
351163	k__Bacteria;p__Firmicutes;c__Clostridia;o__Clostridiales;f__Lachnospiraceae;g__Blautia;s__producta	7.95E-08	1.39E+00	5.97E-02
New.ReferenceOTU618	k__Bacteria;p__Firmicutes;c__Clostridia;o__Clostridiales;f__Lachnospiraceae	7.95E-08	2.01E+00	3.28E-01
185420	k__Bacteria;p__Bacteroidetes;c__Bacteroidia;o__Bacteroidales;f__Bacteroidaceae;g__Bacteroides;s__	8.19E-08	9.44E+00	1.01E+00
290418	k__Bacteria;p__Spirochaetes;c__Spirochaetes;o__Spirochaetales;f__Spirochaetaceae;g__Treponema;s__	9.06E-08	9.30E-01	4.96E+00
1952	k__Bacteria;p__Bacteroidetes;c__Bacteroidia;o__Bacteroidales;f__Porphyromonadaceae;g__Parabacteroides;s__	9.67E-08	1.66E+01	2.04E+00
291535	k__Bacteria;p__Firmicutes;c__Clostridia;o__Clostridiales;f__Lachnospiraceae;g__;s__	9.89E-08	9.94E+00	4.78E+01
3563235	k__Bacteria;p__Bacteroidetes;c__Bacteroidia;o__Bacteroidales;f__Bacteroidaceae;g__Bacteroides;s__	1.05E-07	4.17E+01	1.23E+01
106860	k__Bacteria;p__Firmicutes;c__Clostridia;o__Clostridiales;f__Lachnospiraceae;g__;s__	1.10E-07	1.41E-01	3.25E+00
350360	k__Bacteria;p__Bacteroidetes;c__Bacteroidia;o__Bacteroidales;f__;g__;s__	1.10E-07	5.92E-01	7.19E+00
526583	k__Bacteria;p__Firmicutes;c__Clostridia;o__Clostridiales;f__Peptostreptococcaceae;g__;s__	1.18E-07	3.83E+00	3.28E-01
153168	k__Archaea;p__Euryarchaeota;c__Methanobacteria;o__Methanobacteriales;f__Methanobacteriaceae;g__Methanobrevibacter;s__	1.22E-07	1.68E+02	5.37E+02
320863	k__Bacteria;p__Firmicutes;c__Clostridia;o__Clostridiales;f__Lachnospiraceae;g__;s__	1.23E-07	7.89E-01	5.94E+00
303347	k__Bacteria;p__Bacteroidetes;c__Bacteroidia;o__Bacteroidales;f__;g__;s__	1.23E-07	4.23E-02	1.45E+00
297210	k__Bacteria;p__Firmicutes;c__Clostridia;o__Clostridiales;f__;g__;s__	1.23E-07	9.01E-01	2.52E+00
327184	k__Bacteria;p__Firmicutes;c__Clostridia;o__Clostridiales;f__;g__;s__	1.25E-07	1.32E+00	9.76E+00
208479	k__Bacteria;p__Bacteroidetes;c__Bacteroidia;o__Bacteroidales;f__[Odoribacteraceae];g__Butyricimonas;s__	1.26E-07	1.25E+01	1.60E+00
310601	k__Bacteria;p__Firmicutes;c__Clostridia;o__Clostridiales;f__Christensenellaceae;g__;s__	1.40E-07	1.49E+00	4.55E+00
293605	k__Bacteria;p__Bacteroidetes;c__Bacteroidia;o__Bacteroidales;f__;g__;s__	1.42E-07	3.63E+00	2.28E+01

300139	k__Bacteria;p__Firmicutes;c__Clostridia;o__Clostridiales;f__;g__;s__	1.43E-07	5.92E-01	4.58E+00
3588390	k__Bacteria;p__Bacteroidetes;c__Bacteroidia;o__Bacteroidales;f__Bacteroidaceae;g__Bacteroides;s__	1.44E-07	1.99E+01	5.39E+00
214036	k__Bacteria;p__Firmicutes;c__Clostridia;o__Clostridiales;f__[Mogibacteriaceae];g__;s__	1.44E-07	1.87E+00	1.79E-01
234959	k__Bacteria;p__Tenericutes;c__Mollicutes;o__RF39;f__;g__;s__	1.47E-07	6.00E+00	3.28E-01
New.CleanUp.ReferenceOTU178859	Unassigned	1.47E-07	2.54E-01	2.76E+00
301334	k__Bacteria;p__Verrucomicrobia;c__Verruco-5;o__WCHB1-41;f__RFP12;g__;s__	1.48E-07	9.86E-01	4.19E+00
331281	k__Bacteria;p__Firmicutes;c__Clostridia;o__Clostridiales;f__Clostridiaceae;g__;s__	1.48E-07	2.46E+01	1.14E+01
289217	k__Bacteria;p__Spirochaetes;c__Spirochaetes;o__Spirochaetales;f__Spirochaetaceae;g__Treponema;s__	1.53E-07	1.41E-01	1.18E+00
New.ReferenceOTU292	k__Bacteria;p__Bacteroidetes;c__Bacteroidia;o__Bacteroidales;f__;g__;s__	1.59E-07	3.66E-01	3.70E+00
298714	k__Bacteria;p__Firmicutes;c__Clostridia;o__Clostridiales;f__Lachnospiraceae;g__;s__	1.59E-07	2.13E+00	1.18E+01
New.ReferenceOTU42	k__Bacteria;p__Firmicutes;c__Clostridia;o__Clostridiales;f__Eubacteriaceae;g__Pseudoramibacter_Eubacterium;s__	1.61E-07	2.49E+00	1.50E+01
297140	k__Bacteria;p__Spirochaetes;c__Spirochaetes;o__Spirochaetales;f__Spirochaetaceae;g__Treponema;s__	1.67E-07	1.17E+00	4.63E+00
338105	k__Bacteria;p__Firmicutes;c__Clostridia;o__Clostridiales	1.68E-07	2.07E+00	2.54E-01
New.ReferenceOTU232	k__Bacteria;p__Planctomycetes;c__Planctomycetia;o__Pirellulales;f__Pirellulaceae;g__;s__	1.77E-07	8.45E-02	1.79E+00
New.ReferenceOTU518	k__Bacteria;p__Bacteroidetes;c__Bacteroidia;o__Bacteroidales;f__;g__;s__	1.77E-07	8.03E-01	6.34E+00
2256425	k__Bacteria;p__Firmicutes;c__Clostridia;o__Clostridiales;f__Christensenellaceae;g__;s__	1.77E-07	3.28E+00	4.33E-01
293925	k__Bacteria;p__Verrucomicrobia;c__Verruco-5;o__WCHB1-41;f__RFP12;g__;s__	1.94E-07	3.52E-01	1.82E+00
350909	k__Bacteria;p__Bacteroidetes;c__Bacteroidia;o__Bacteroidales;f__;g__;s__	1.94E-07	7.18E-01	3.64E+00
New.ReferenceOTU23	k__Bacteria;p__Firmicutes;c__Clostridia;o__Clostridiales;f__Christensenellaceae;g__;s__	1.98E-07	1.58E+00	4.45E+00
4442899	k__Bacteria;p__Firmicutes;c__Clostridia;o__Clostridiales;f__;g__;s__	2.09E-07	7.28E+00	7.46E-02
New.ReferenceOTU14	k__Bacteria;p__Firmicutes;c__Clostridia;o__Clostridiales;f__;g__;s__	2.19E-07	1.59E+00	5.51E+00
New.ReferenceOTU611	k__Bacteria;p__Firmicutes;c__Clostridia;o__Clostridiales;f__Ruminococcaceae;g__Ruminococcus;s__	2.22E-07	2.82E-02	4.82E+00
352027	k__Bacteria;p__Firmicutes;c__Clostridia;o__Clostridiales;f__[Mogibacteriaceae];g__;s__	2.23E-07	1.54E+00	6.85E+00
292215	k__Bacteria;p__Firmicutes;c__Clostridia;o__Clostridiales;f__;g__;s__	2.25E-07	5.49E-01	2.51E+00
292458	k__Bacteria;p__Spirochaetes;c__Spirochaetes;o__Spirochaetales;f__Spirochaetaceae;g__Treponema;s__	2.26E-07	1.83E-01	1.76E+00
326785	k__Bacteria;p__Firmicutes;c__Clostridia;o__Clostridiales;f__Ruminococcaceae;g__;s__	2.37E-07	1.84E+01	5.03E+01
320587	k__Bacteria;p__Firmicutes;c__Clostridia;o__Clostridiales;f__Ruminococcaceae;g__Ruminococcus;s__	2.38E-07	7.37E+00	2.06E+01
575681	k__Bacteria;p__Firmicutes;c__Clostridia;o__Clostridiales;f__Christensenellaceae;g__;s__	2.48E-07	7.04E-02	8.49E+00
3421266	k__Bacteria;p__Firmicutes;c__Clostridia;o__Clostridiales;f__Lachnospiraceae;g__[Ruminococcus];s__	2.51E-07	4.15E+00	1.49E-02
263895	k__Bacteria;p__Firmicutes;c__Clostridia;o__Clostridiales;f__Peptococcaceae;g__rc4-4;s__	2.73E-07	2.28E+00	3.73E-01
178770	k__Bacteria;p__Firmicutes;c__Clostridia;o__Clostridiales;f__Lachnospiraceae;g__Dorea;s__	2.75E-07	9.72E-01	0.00E+00
299042	k__Bacteria;p__Firmicutes;c__Clostridia;o__Clostridiales;f__;g__;s__	2.75E-07	1.44E+01	3.00E+01

289426	k__Bacteria;p__Firmicutes;c__Clostridia;o__Clostridiales;f__Ruminococcaceae;g__Oscillospira;s__	2.82E-07	1.41E-02	2.54E+00
1141834	k__Bacteria;p__Verrucomicrobia;c__Verruco-5;o__WCHB1-41;f__RFP12;g__;s__	2.86E-07	1.20E+00	5.13E+00
4396292	k__Bacteria;p__Firmicutes;c__Clostridia;o__Clostridiales;f__Lachnospiraceae;g__;s__	2.86E-07	2.80E+00	8.96E-02
354563	k__Bacteria;p__Firmicutes;c__Clostridia;o__Clostridiales;f__Lachnospiraceae;g__;s__	2.91E-07	9.30E-01	5.81E+00
4436975	k__Bacteria;p__Firmicutes;c__Bacilli;o__Lactobacillales;f__Streptococcaceae;g__Streptococcus;s__	2.91E-07	1.21E+01	1.48E+00
179657	k__Bacteria;p__Firmicutes;c__Clostridia;o__Clostridiales;f__Lachnospiraceae;g__Blautia;s__producta	3.00E-07	3.31E+00	4.78E-01
2771899	k__Bacteria;p__Firmicutes;c__Clostridia;o__Clostridiales;f__Ruminococcaceae;g__;s__	3.01E-07	4.61E+00	1.34E-01
347801	k__Bacteria;p__Firmicutes;c__Clostridia;o__Clostridiales;f__Lachnospiraceae;g__;s__	3.02E-07	6.77E+00	1.52E+00
New.ReferenceOTU310	k__Bacteria;p__Firmicutes;c__Clostridia;o__Clostridiales;f__Lachnospiraceae;g__;s__	3.31E-07	6.48E-01	4.52E+00
New.ReferenceOTU811	k__Bacteria;p__Spirochaetes;c__Spirochaetes;o__Spirochaetales;f__Spirochaetaceae;g__Treponema;s__	3.44E-07	3.66E-01	2.16E+00
292057	k__Bacteria;p__Firmicutes;c__Bacilli;o__Lactobacillales;f__Lactobacillaceae;g__Lactobacillus;s__	3.52E-07	8.07E+00	2.99E-01
345353	k__Bacteria;p__Firmicutes;c__Clostridia;o__Clostridiales;f__Ruminococcaceae;g__;s__	3.53E-07	1.07E+00	3.64E+00
183450	k__Bacteria;p__Firmicutes;c__Clostridia;o__Clostridiales;f__Lachnospiraceae;g__Blautia;s__	3.55E-07	1.52E+00	2.69E-01
4267520	k__Bacteria;p__Firmicutes;c__Clostridia;o__Clostridiales;f__Ruminococcaceae;g__;s__	3.85E-07	2.72E+00	1.64E-01
180585	k__Bacteria;p__Firmicutes;c__Clostridia;o__Clostridiales;f__;g__;s__	3.97E-07	3.34E+00	5.37E-01
295951	k__Bacteria;p__Firmicutes;c__Clostridia;o__Clostridiales;f__Lachnospiraceae;g__;s__	4.03E-07	2.42E+00	9.01E+00
590836	k__Bacteria;p__Firmicutes;c__Bacilli;o__Lactobacillales;f__Lactobacillaceae;g__Lactobacillus;s__	4.25E-07	1.75E+01	6.66E+00
New.ReferenceOTU993	k__Bacteria;p__Firmicutes;c__Clostridia;o__Clostridiales;f__Ruminococcaceae;g__;s__	4.33E-07	2.01E+00	6.12E+00
New.ReferenceOTU564	k__Bacteria;p__Firmicutes;c__Clostridia;o__Clostridiales;f__;g__;s__	4.45E-07	1.25E+01	1.87E+01
302375	k__Bacteria;p__Firmicutes;c__Clostridia;o__Clostridiales;f__[Mogibacteriaceae];g__;s__	4.53E-07	1.85E+00	5.39E+00
344208	k__Bacteria;p__Firmicutes;c__Clostridia;o__Clostridiales;f__Ruminococcaceae;g__;s__	4.75E-07	3.66E-01	2.67E+00
2272351	k__Bacteria;p__Firmicutes;c__Clostridia;o__Clostridiales;f__;g__;s__	4.77E-07	4.04E+00	4.48E-02
4296763	k__Bacteria;p__Firmicutes;c__Clostridia;o__Clostridiales;f__;g__;s__	4.83E-07	5.63E-01	4.33E+00
321517	k__Bacteria;p__Firmicutes;c__Clostridia;o__Clostridiales;f__Ruminococcaceae;g__Ruminococcus;s__	4.96E-07	1.24E+00	8.15E+00
New.ReferenceOTU69	k__Bacteria;p__Firmicutes;c__Clostridia;o__Clostridiales;f__;g__;s__	5.05E-07	1.31E+00	6.51E+00
306288	k__Bacteria;p__Firmicutes;c__Clostridia;o__Clostridiales;f__Lachnospiraceae;g__Coprococcus;s__	5.12E-07	3.54E+00	5.22E-01
175087	k__Bacteria;p__Firmicutes;c__Clostridia;o__Clostridiales	5.15E-07	1.24E+00	1.19E-01
333114	k__Bacteria;p__Firmicutes;c__Clostridia;o__Clostridiales	5.19E-07	1.07E+00	0.00E+00
1147637	k__Bacteria;p__Actinobacteria;c__Coriobacteriia;o__Coriobacteriales;f__Coriobacteriaceae;g__Slackia;s__	5.19E-07	4.11E+00	0.00E+00
New.ReferenceOTU984	k__Bacteria;p__Firmicutes;c__Clostridia;o__Clostridiales;f__;g__;s__	5.51E-07	1.41E-02	2.81E+00
323737	k__Bacteria;p__Firmicutes;c__Clostridia;o__Clostridiales;f__Ruminococcaceae;g__;s__	5.57E-07	1.55E-01	1.03E+00
110910	k__Bacteria;p__Firmicutes;c__Clostridia;o__Clostridiales;f__Christensenellaceae;g__;s__	5.59E-07	1.24E+01	1.89E+01

3426658	k__Bacteria;p__Bacteroidetes;c__Bacteroidia;o__Bacteroidales;f__Bacteroidaceae;g__Bacteroides;s__	5.66E-07	1.00E+01	2.64E+00
New.CleanUp.ReferenceOTU24359	k__Bacteria;p__Spirochaetes;c__Spirochaetes;o__Spirochaetales;f__Spirochaetaceae;g__Treponema;s__	5.75E-07	4.23E-02	1.69E+00
301127	k__Bacteria;p__Chloroflexi;c__Anaerolineae;o__Anaerolineales;f__Anaerolinaceae;g__SHD-231;s__	5.86E-07	5.07E-01	3.34E+00
180107	k__Bacteria;p__Firmicutes;c__Clostridia;o__Clostridiales;f__Ruminococcaceae;g__Ruminococcus;s__	5.92E-07	2.92E+00	3.43E-01
New.ReferenceOTU179	k__Bacteria;p__Bacteroidetes;c__Bacteroidia;o__Bacteroidales;f__[Paraprevotellaceae];g__;s__	5.97E-07	7.75E-01	6.15E+00
331675	k__Bacteria;p__Verrucomicrobia;c__Verruco-5;o__LD1-PB3;f__;g__;s__	6.08E-07	1.41E-02	6.07E+00
300250	k__Bacteria;p__Firmicutes;c__Clostridia;o__Clostridiales	6.20E-07	4.08E-01	3.94E+00
288119	k__Bacteria;p__Bacteroidetes;c__Bacteroidia;o__Bacteroidales;f__BS11;g__;s__	6.36E-07	6.20E-01	8.75E+00
395587	k__Bacteria;p__Firmicutes;c__Clostridia;o__Clostridiales;f__Ruminococcaceae;g__;s__	6.38E-07	4.86E+00	1.49E-01
360636	k__Bacteria;p__Firmicutes;c__Erysipelotrichi;o__Erysipelotrichales;f__Erysipelotrichaceae;g__;s__	6.38E-07	9.48E+00	1.99E+00
304757	k__Bacteria;p__Firmicutes;c__Clostridia;o__Clostridiales;f__Lachnospiraceae;g__Blautia;s__	6.87E-07	2.69E+00	2.99E-02
New.ReferenceOTU444	k__Bacteria;p__Firmicutes;c__Clostridia;o__Clostridiales;f__Lachnospiraceae;g__Coprococcus;s__	6.98E-07	2.59E+00	5.09E+00
296079	k__Bacteria;p__Bacteroidetes;c__Bacteroidia;o__Bacteroidales;f__;g__;s__	7.02E-07	3.44E+00	1.20E+01
746079	k__Bacteria;p__Bacteroidetes;c__Bacteroidia;o__Bacteroidales;f__;g__;s__	7.06E-07	5.63E-02	3.99E+00
288873	k__Bacteria;p__Bacteroidetes;c__Bacteroidia;o__Bacteroidales;f__;g__;s__	7.25E-07	2.20E+00	7.64E+00
365297	k__Bacteria;p__Firmicutes;c__Clostridia;o__Clostridiales;f__;g__;s__	7.35E-07	3.75E+00	3.58E-01
New.ReferenceOTU712	k__Bacteria;p__Firmicutes;c__Clostridia;o__Clostridiales;f__[Mogibacteriaceae];g__;s__	7.41E-07	7.45E+00	1.60E+01
328254	k__Bacteria;p__Bacteroidetes;c__Bacteroidia;o__Bacteroidales;f__;g__;s__	7.49E-07	9.58E-01	2.96E+00
288541	k__Bacteria;p__Bacteroidetes;c__Bacteroidia;o__Bacteroidales;f__;g__;s__	7.54E-07	4.92E+00	1.79E+01
3154070	k__Bacteria;p__Bacteroidetes;c__Bacteroidia;o__Bacteroidales;f__Bacteroidaceae;g__Bacteroides;s__	7.54E-07	2.42E+00	6.72E-01
308338	k__Bacteria;p__Firmicutes;c__Clostridia;o__Clostridiales;f__Lachnospiraceae;g__Dorea;s__	7.54E-07	7.31E+01	2.25E+01
293133	k__Bacteria;p__Firmicutes;c__Clostridia;o__Clostridiales;f__[Mogibacteriaceae];g__;s__	7.72E-07	2.69E+00	5.64E+00
101237	k__Bacteria;p__Firmicutes;c__Clostridia;o__Clostridiales;f__Lachnospiraceae;g__Coprococcus;s__	7.76E-07	1.03E+01	3.07E+01
4468892	k__Bacteria;p__Firmicutes;c__Clostridia;o__Clostridiales;f__Ruminococcaceae;g__;s__	7.90E-07	9.58E-01	8.96E-02
305417	k__Bacteria;p__Firmicutes;c__Clostridia;o__Clostridiales;f__;g__;s__	8.03E-07	9.86E-01	3.73E+00
344306	k__Bacteria;p__Bacteroidetes;c__Bacteroidia;o__Bacteroidales;f__Bacteroidaceae;g__BF311;s__	8.26E-07	5.77E-01	9.01E+00
321606	k__Bacteria;p__Verrucomicrobia;c__Verrucomicrobiae;o__Verrucomicrobiales;f__Verrucomicrobiaceae;g__Akkermansia;s__	8.27E-07	1.80E+01	5.09E+01
121873	k__Bacteria;p__Firmicutes;c__Clostridia;o__Clostridiales;f__Dehalobacteriaceae;g__Dehalobacterium;s__	8.90E-07	5.21E-01	2.34E+00
182886	k__Bacteria;p__Bacteroidetes;c__Bacteroidia;o__Bacteroidales;f__Bacteroidaceae;g__Bacteroides;s__uniformis	8.90E-07	9.30E-01	1.49E-02
New.ReferenceOTU221	k__Bacteria;p__Bacteroidetes;c__Bacteroidia;o__Bacteroidales;f__;g__;s__	8.98E-07	2.96E-01	2.67E+00
110563	k__Bacteria;p__Firmicutes;c__Clostridia;o__Clostridiales;f__Ruminococcaceae;g__;s__	8.98E-07	8.55E+00	2.51E+01
New.ReferenceOTU156	k__Bacteria;p__Verrucomicrobia;c__Verruco-5;o__WCHB1-41;f__RFP12;g__;s__	8.98E-07	2.96E-01	1.82E+00

New.CleanUp.ReferenceOTU176789	k__Bacteria;p__Firmicutes;c__Clostridia;o__Clostridiales;f__;g__;s__	8.98E-07	2.11E-01	2.96E+00
288438	k__Bacteria;p__Firmicutes;c__Clostridia;o__Clostridiales;f__;g__;s__	9.27E-07	5.63E+00	5.07E-01
New.CleanUp.ReferenceOTU111068	k__Bacteria;p__Firmicutes;c__Clostridia;o__Clostridiales;f__Clostridiaceae;g__Clostridium;s__	9.32E-07	1.15E+00	1.49E-02
275237	k__Bacteria;p__Firmicutes;c__Clostridia;o__Clostridiales;f__Veillonellaceae;g__Phascolarctobacterium;s__	9.37E-07	2.72E+01	1.76E+00
310409	k__Bacteria;p__Firmicutes;c__Clostridia;o__Clostridiales;f__Lachnospiraceae;g__;s__	9.64E-07	2.62E+00	5.34E+00
4370257	k__Bacteria;p__Firmicutes;c__Clostridia;o__Clostridiales;f__Ruminococcaceae;g__;s__	9.66E-07	5.07E-01	3.85E+00
336333	k__Bacteria;p__Firmicutes;c__Clostridia;o__Clostridiales;f__Eubacteriaceae;g__Anaerofustis;s__	1.02E-06	9.15E-01	3.78E+00
328905	k__Bacteria;p__Firmicutes;c__Clostridia;o__Clostridiales;f__Ruminococcaceae;g__Oscillospira;s__	1.08E-06	1.90E+00	8.96E-02
301137	k__Bacteria;p__Actinobacteria;c__Coriobacteriia;o__Coriobacteriales;f__Coriobacteriaceae;g__;s__	1.08E-06	1.28E+00	3.82E+00
290862	k__Bacteria;p__Verrucomicrobia;c__Verruco-5;o__WCHB1-41;f__RFP12;g__;s__	1.09E-06	1.83E-01	1.52E+00
351533	k__Bacteria;p__Bacteroidetes;c__Bacteroidia;o__Bacteroidales;f__;g__;s__	1.09E-06	3.38E-01	3.52E+00
291494	k__Bacteria;p__Firmicutes;c__Clostridia;o__Clostridiales;f__;g__;s__	1.10E-06	7.04E-02	1.27E+00
155951	k__Bacteria;p__Firmicutes;c__Clostridia;o__Clostridiales;f__Ruminococcaceae;g__Ruminococcus;s__	1.11E-06	1.72E+00	7.39E+00
295070	k__Bacteria;p__Firmicutes;c__Clostridia;o__Clostridiales;f__;g__;s__	1.11E-06	1.89E+00	5.18E+00
New.ReferenceOTU398	k__Bacteria;p__Bacteroidetes;c__Bacteroidia;o__Bacteroidales;f__Bacteroidaceae;g__;s__	1.12E-06	2.06E+00	8.16E+00
320353	k__Bacteria;p__Verrucomicrobia;c__Verruco-5;o__WCHB1-41;f__RFP12;g__;s__	1.12E-06	3.83E+00	6.96E+00
288494	k__Bacteria;p__Verrucomicrobia;c__Verrucomicrobiae;o__Verrucomicrobiales;f__Verrucomicrobiaceae;g__Akkermansia;s__	1.12E-06	2.75E+00	1.45E+01
102221	k__Bacteria;p__Firmicutes;c__Clostridia;o__Clostridiales;f__Lachnospiraceae;g__;s__	1.14E-06	3.46E+00	9.46E+00
288594	k__Bacteria;p__Spirochaetes;c__Spirochaetes;o__Spirochaetales;f__Spirochaetaceae;g__Treponema;s__	1.15E-06	6.76E-01	3.21E+00
New.ReferenceOTU281	k__Bacteria;p__Firmicutes;c__Clostridia;o__Clostridiales;f__Lachnospiraceae;g__;s__	1.15E-06	5.49E-01	2.49E+00
New.ReferenceOTU972	k__Bacteria;p__Bacteroidetes;c__Bacteroidia;o__Bacteroidales;f__[Paraprevotellaceae];g__YRC22;s__	1.20E-06	2.07E+00	8.52E+00
New.ReferenceOTU809	k__Bacteria;p__Actinobacteria;c__Actinobacteria;o__Actinomycetales;f__Actinomycetaceae;g__Arcanobacterium;s__	1.26E-06	1.24E+00	6.42E+00
307890	k__Bacteria;p__Firmicutes;c__Clostridia;o__Clostridiales;f__Lachnospiraceae;g__Blautia;s__	1.28E-06	2.21E+00	2.99E-02
358030	k__Bacteria;p__Firmicutes;c__Clostridia;o__Clostridiales;f__Ruminococcaceae;g__;s__	1.29E-06	1.90E+00	1.04E-01
New.ReferenceOTU402	k__Bacteria;p__Firmicutes;c__Clostridia;o__Clostridiales;f__;g__;s__	1.33E-06	3.89E+00	2.99E-02
150412	k__Bacteria;p__Bacteroidetes;c__Bacteroidia;o__Bacteroidales;f__Porphyromonadaceae;g__Parabacteroides;s__	1.39E-06	6.86E+00	6.12E-01
New.ReferenceOTU12	k__Bacteria;p__Verrucomicrobia;c__Verrucomicrobiae;o__Verrucomicrobiales;f__Verrucomicrobiaceae;g__Akkermansia;s__	1.42E-06	1.68E+00	1.66E+01
288557	k__Bacteria;p__Firmicutes;c__Clostridia;o__Clostridiales;f__Ruminococcaceae;g__;s__	1.45E-06	1.73E+00	5.25E+00
16006	k__Bacteria;p__Firmicutes;c__Clostridia;o__Clostridiales;f__;g__;s__	1.50E-06	3.20E+00	6.12E-01
New.ReferenceOTU468	k__Bacteria;p__Verrucomicrobia;c__Verruco-5;o__WCHB1-41;f__RFP12;g__;s__	1.56E-06	3.24E-01	1.78E+00
New.ReferenceOTU754	Unassigned	1.57E-06	7.18E-01	3.09E+00
215468	k__Bacteria;p__Firmicutes;c__Clostridia;o__Clostridiales;f__Ruminococcaceae;g__;s__	1.68E-06	1.13E-01	1.25E+00

208949	k__Bacteria;p__Bacteroidetes;c__Bacteroidia;o__Bacteroidales;f__Bacteroidaceae;g__Bacteroides;s__	1.74E-06	1.30E+00	1.88E+01
New.ReferenceOTU163	k__Bacteria;p__Firmicutes;c__Clostridia;o__Clostridiales;f__Ruminococcaceae;g__;s__	1.75E-06	1.42E+00	7.46E-02
291412	k__Bacteria;p__Bacteroidetes;c__Bacteroidia;o__Bacteroidales;f__;g__;s__	1.81E-06	1.97E+00	5.54E+00
New.ReferenceOTU31	k__Bacteria;p__Firmicutes;c__Clostridia;o__Clostridiales;f__Lachnospiraceae;g__Blautia;s__producta	1.81E-06	7.32E-01	0.00E+00
1066464	k__Bacteria;p__Verrucomicrobia;c__Verruco-5;o__WCHB1-41;f__RFP12;g__;s__	1.88E-06	2.82E-01	1.90E+00
4425214	k__Bacteria;p__Firmicutes;c__Bacilli;o__Lactobacillales;f__Streptococcaceae;g__Streptococcus;s__	1.89E-06	5.17E+00	5.52E-01
New.ReferenceOTU788	k__Bacteria;p__Firmicutes;c__Clostridia;o__Clostridiales	1.95E-06	9.86E-02	1.10E+00
343633	k__Bacteria;p__Actinobacteria;c__Coriobacteriia;o__Coriobacteriales;f__Coriobacteriaceae;g__;s__	1.98E-06	1.38E+00	5.30E+00
332718	k__Bacteria;p__Firmicutes;c__Bacilli;o__Lactobacillales;f__Streptococcaceae;g__Streptococcus;s__	2.04E-06	6.03E+00	1.31E+00
360730	k__Bacteria;p__Bacteroidetes;c__Bacteroidia;o__Bacteroidales;f__[Odoribacteraceae];g__Butyricimonas;s__	2.11E-06	4.59E+00	4.93E-01
297744	k__Bacteria;p__Firmicutes;c__Clostridia;o__Clostridiales;f__[Mogibacteriaceae];g__;s__	2.11E-06	5.21E-01	2.40E+00
290559	k__Bacteria;p__Firmicutes;c__Clostridia;o__Clostridiales;f__;g__;s__	2.18E-06	6.48E+00	1.27E+01
3134492	k__Bacteria;p__Firmicutes;c__Clostridia;o__Clostridiales;f__Lachnospiraceae;g__;s__	2.20E-06	1.99E+00	7.16E-01
294370	k__Bacteria;p__Firmicutes;c__Clostridia;o__Clostridiales;f__Lachnospiraceae;g__;s__	2.22E-06	1.18E+01	1.48E+00
320221	k__Bacteria;p__Firmicutes;c__Clostridia;o__Clostridiales	2.27E-06	4.37E-01	2.57E+00
213401	k__Bacteria;p__Firmicutes;c__Clostridia;o__Clostridiales;f__;g__;s__	2.34E-06	4.13E+00	2.09E-01
544996	k__Bacteria;p__Firmicutes;c__Clostridia;o__Clostridiales;f__Ruminococcaceae;g__Oscillospira;s__	2.34E-06	3.08E+00	1.04E-01
191738	k__Bacteria;p__Firmicutes;c__Clostridia;o__Clostridiales;f__;g__;s__	2.40E-06	4.77E+00	2.24E-01
4364405	k__Bacteria;p__Firmicutes;c__Clostridia;o__Clostridiales;f__Ruminococcaceae;g__;s__	2.47E-06	1.48E+01	3.73E+00
354652	k__Bacteria;p__Bacteroidetes;c__Bacteroidia;o__Bacteroidales;f__;g__;s__	2.49E-06	1.59E+00	6.96E+00
New.ReferenceOTU583	k__Bacteria;p__Firmicutes;c__Bacilli;o__Lactobacillales;f__Lactobacillaceae;g__Lactobacillus;s__	2.58E-06	2.61E+00	3.13E-01
306056	k__Bacteria;p__Firmicutes;c__Clostridia;o__Clostridiales;f__;g__;s__	2.61E-06	2.73E+00	6.96E+00
322729	k__Bacteria;p__Firmicutes;c__Clostridia;o__Clostridiales;f__Ruminococcaceae;g__;s__	2.66E-06	5.01E+00	1.76E+01
New.ReferenceOTU455	k__Bacteria;p__Firmicutes;c__Clostridia;o__Clostridiales;f__Christensenellaceae;g__;s__	2.68E-06	3.10E-01	1.79E+00
289923	k__Bacteria;p__Actinobacteria;c__Actinobacteria;o__Actinomycetales;f__Micrococcaceae;g__Arthrobacter;s__	2.72E-06	1.04E+01	1.07E+00
4450291	k__Bacteria;p__Firmicutes;c__Clostridia;o__Clostridiales;f__Ruminococcaceae;g__;s__	2.75E-06	3.38E-01	3.07E+00
New.ReferenceOTU35	k__Bacteria;p__Bacteroidetes;c__Bacteroidia;o__Bacteroidales;f__;g__;s__	2.89E-06	5.77E-01	3.69E+00
575557	k__Bacteria;p__Firmicutes;c__Erysipelotrichi;o__Erysipelotrichales;f__Erysipelotrichaceae;g__Coprobacillus;s__	3.00E-06	3.03E+00	2.99E-02
525215	k__Bacteria;p__Firmicutes;c__Clostridia;o__Clostridiales;f__Ruminococcaceae;g__Faecalibacterium;s__prausnitzii	3.01E-06	4.32E+00	8.06E-01
290654	k__Bacteria;p__Synergistetes;c__Synergistia;o__Synergistales;f__Synergistaceae;g__;s__	3.16E-06	1.72E+00	6.96E+00
342085	k__Bacteria;p__Firmicutes;c__Clostridia;o__Clostridiales;f__Lachnospiraceae;g__;s__	3.17E-06	7.89E+00	2.09E-01
295853	k__Bacteria;p__Firmicutes;c__Clostridia;o__Clostridiales;f__[Mogibacteriaceae];g__;s__	3.19E-06	3.18E+00	6.13E+00

4365130	k__Bacteria;p__Bacteroidetes;c__Bacteroidia;o__Bacteroidales;f__Porphyromonadaceae;g__Parabacteroides;s__distasonis	3.21E-06	7.23E+00	6.87E-01
New.ReferenceOTU659	k__Bacteria;p__Firmicutes;c__Clostridia;o__Clostridiales;f__;g__;s__	3.31E-06	9.44E-01	2.21E+00
312384	k__Bacteria;p__Verrucomicrobia;c__Verruco-5;o__WCHB1-41;f__RFP12;g__;s__	3.33E-06	4.79E-01	2.51E+00
179067	k__Bacteria;p__Firmicutes;c__Clostridia;o__Clostridiales;f__Lachnospiraceae;g__Blautia;s__producta	3.33E-06	4.30E+00	7.31E-01
344860	k__Bacteria;p__Firmicutes;c__Clostridia;o__Clostridiales;f__Christensenellaceae;g__;s__	3.33E-06	1.41E-01	2.34E+00
353775	k__Bacteria;p__Firmicutes;c__Clostridia;o__Clostridiales;f__Lachnospiraceae;g__;s__	3.33E-06	6.20E-01	2.42E+00
150477	k__Archaea;p__Euryarchaeota;c__Methanobacteria;o__Methanobacteriales;f__Methanobacteriaceae;g__Methanosphaera;s__	3.33E-06	3.10E+00	1.79E-01
662852	k__Bacteria;p__Firmicutes;c__Clostridia;o__Clostridiales;f__;g__;s__	3.38E-06	5.46E+00	1.20E+01
351095	k__Bacteria;p__Firmicutes;c__Clostridia;o__Clostridiales;f__Lachnospiraceae	3.43E-06	8.03E-01	0.00E+00
294246	k__Bacteria;p__Firmicutes;c__Clostridia;o__Clostridiales;f__Ruminococcaceae;g__;s__	3.43E-06	1.65E+01	2.00E+00
2264288	k__Bacteria;p__Firmicutes;c__Bacilli;o__Lactobacillales;f__Carnobacteriaceae;g__Desemzia;s__	3.46E-06	4.07E+00	1.64E-01
554463	k__Bacteria;p__Firmicutes;c__Clostridia;o__Clostridiales;f__Ruminococcaceae;g__;s__	3.49E-06	2.11E-01	2.60E+00
4437748	k__Bacteria;p__Firmicutes;c__Clostridia;o__Clostridiales;f__;g__;s__	3.49E-06	9.72E-01	0.00E+00
265563	k__Bacteria;p__Firmicutes;c__Clostridia;o__Clostridiales;f__Lachnospiraceae;g__Dorea;s__	3.49E-06	1.79E+00	2.09E-01
106258	k__Bacteria;p__Firmicutes;c__Clostridia;o__Clostridiales;f__;g__;s__	3.49E-06	2.06E+00	0.00E+00
110836	k__Bacteria;p__Firmicutes;c__Clostridia;o__Clostridiales;f__Ruminococcaceae;g__;s__	3.53E-06	1.04E+00	3.12E+00
126	k__Archaea;p__Euryarchaeota;c__Methanobacteria;o__Methanobacteriales;f__Methanobacteriaceae;g__Methanobrevibacter;s__	3.53E-06	2.01E+01	4.33E+01
351773	k__Bacteria;p__Firmicutes;c__Clostridia;o__Clostridiales;f__Ruminococcaceae;g__;s__	3.54E-06	5.07E-01	2.06E+00
296516	k__Bacteria;p__Firmicutes;c__Clostridia;o__Clostridiales;f__Lachnospiraceae;g__;s__	3.81E-06	3.35E+00	6.06E+00
New.CleanUp.ReferenceOTU53482	k__Bacteria;p__Firmicutes;c__Clostridia;o__Clostridiales;f__Lachnospiraceae;g__;s__	3.84E-06	2.82E-02	1.25E+00
New.ReferenceOTU528	k__Bacteria;p__Firmicutes;c__Clostridia;o__Clostridiales;f__;g__;s__	3.87E-06	8.87E-01	4.54E+00
New.ReferenceOTU622	k__Bacteria;p__Firmicutes;c__Clostridia;o__Clostridiales;f__Ruminococcaceae;g__;s__	3.88E-06	2.27E+01	4.76E+01
4446741	k__Bacteria;p__Firmicutes;c__Clostridia;o__Clostridiales;f__Lachnospiraceae;g__;s__	3.96E-06	7.46E-01	2.52E+00
340960	k__Bacteria;p__Firmicutes;c__Bacilli;o__Lactobacillales;f__Lactobacillaceae;g__Lactobacillus;s__	3.98E-06	5.94E+00	6.42E-01
4449427	k__Bacteria;p__Firmicutes;c__Clostridia;o__Clostridiales;f__Lachnospiraceae;g__[Ruminococcus];s__	3.98E-06	4.00E+00	2.09E-01
367176	k__Bacteria;p__Firmicutes;c__Clostridia;o__Clostridiales;f__Ruminococcaceae;g__Oscillospira;s__	3.98E-06	1.07E+00	5.97E-02
346761	k__Bacteria;p__Firmicutes;c__Clostridia;o__Clostridiales;f__Ruminococcaceae;g__;s__	3.98E-06	2.52E+00	3.58E-01
293994	k__Bacteria;p__Firmicutes;c__Erysipelotrichi;o__Erysipelotrichales;f__Erysipelotrichaceae;g__p-75-a5;s__	3.98E-06	9.15E-01	2.48E+00
667010	k__Bacteria;p__Firmicutes;c__Clostridia;o__Clostridiales;f__;g__;s__	4.12E-06	1.72E+00	1.20E+01
887025	k__Bacteria;p__Firmicutes;c__Clostridia;o__Clostridiales;f__Ruminococcaceae;g__;s__	4.15E-06	7.18E-01	2.01E+00
New.ReferenceOTU531	k__Bacteria;p__Firmicutes;c__Clostridia;o__Clostridiales;f__Lachnospiraceae	4.21E-06	1.08E+00	4.48E-02
299508	k__Bacteria;p__Firmicutes;c__Clostridia;o__Clostridiales;f__Veillonellaceae;g__;s__	4.23E-06	7.89E-01	2.87E+00

297408	k__Bacteria;p__Verrucomicrobia;c__Verruco-5;o__WCHB1-41;f__RFP12;g__;s__	4.48E-06	4.51E-01	2.55E+00
196249	k__Bacteria;p__Firmicutes;c__Clostridia;o__Clostridiales;f__Lachnospiraceae;g__Dorea;s__	4.51E-06	1.01E+00	4.48E-02
289096	k__Bacteria;p__Spirochaetes;c__Spirochaetes;o__Spirochaetales;f__Spirochaetaceae;g__Treponema;s__	4.54E-06	2.68E-01	2.06E+00
332290	k__Bacteria;p__Bacteroidetes;c__Bacteroidia;o__Bacteroidales;f__;g__;s__	4.58E-06	1.83E-01	2.13E+00
309195	k__Bacteria;p__Firmicutes;c__Clostridia;o__Clostridiales;f__Lachnospiraceae;g__;s__	4.71E-06	7.18E-01	2.51E+00
313400	k__Bacteria;p__Firmicutes;c__Clostridia;o__Clostridiales;f__;g__;s__	4.75E-06	6.01E+00	2.83E+01
539647	k__Bacteria;p__Firmicutes;c__Bacilli;o__Lactobacillales;f__Lactobacillaceae;g__Lactobacillus;s__	4.76E-06	7.45E+00	2.28E+00
4446320	k__Bacteria;p__Firmicutes;c__Clostridia;o__Clostridiales;f__Clostridiaceae;g__;s__	4.87E-06	1.27E-01	8.27E+00
4456605	k__Bacteria;p__Firmicutes;c__Clostridia;o__Clostridiales;f__Lachnospiraceae;g__;s__	4.87E-06	5.35E-01	2.27E+00
New.CleanUp.ReferenceOTU130624	k__Bacteria;p__Firmicutes;c__Clostridia;o__Clostridiales;f__Lachnospiraceae;g__;s__	4.92E-06	1.41E-02	5.54E+00
293147	k__Bacteria;p__Firmicutes;c__Clostridia;o__Clostridiales;f__Ruminococcaceae;g__;s__	5.11E-06	4.23E-02	1.43E+00
2123717	k__Bacteria;p__Firmicutes;c__Clostridia;o__Clostridiales;f__Lachnospiraceae;g__Roseburia;s__	5.14E-06	2.58E+00	1.37E+00
New.CleanUp.ReferenceOTU39107	k__Bacteria;p__Firmicutes;c__Clostridia;o__Clostridiales;f__Lachnospiraceae;g__;s__	5.18E-06	1.06E+00	2.99E-02
269913	k__Bacteria;p__Firmicutes;c__Clostridia;o__Clostridiales;f__Ruminococcaceae;g__;s__	5.54E-06	1.69E+00	6.61E+00
301226	k__Bacteria;p__Firmicutes;c__Clostridia;o__Clostridiales;f__Clostridiaceae;g__;s__	5.54E-06	7.63E+00	1.69E+00
317818	k__Bacteria;p__Firmicutes;c__Clostridia;o__Clostridiales;f__Lachnospiraceae;g__;s__	5.54E-06	1.85E+00	4.40E+00
577170	k__Bacteria;p__Bacteroidetes;c__Bacteroidia;o__Bacteroidales;f__Bacteroidaceae;g__Bacteroides;s__	6.11E-06	3.01E+00	3.88E-01
304909	k__Bacteria;p__Bacteroidetes;c__Bacteroidia;o__Bacteroidales;f__;g__;s__	6.60E-06	9.86E-01	3.85E+00
328617	k__Bacteria;p__Bacteroidetes;c__Bacteroidia;o__Bacteroidales;f__Bacteroidaceae;g__Bacteroides	6.60E-06	7.44E+00	3.42E+00
New.CleanUp.ReferenceOTU125526	k__Bacteria;p__Firmicutes;c__Clostridia;o__Clostridiales;f__Christensenellaceae;g__;s__	6.74E-06	5.63E-01	3.58E+00
299176	k__Bacteria;p__Actinobacteria;c__Actinobacteria;o__Actinomycetales;f__Micrococcaceae;g__Arthrobacter;s__	7.15E-06	1.20E+01	1.43E+00
317813	k__Bacteria;p__Firmicutes;c__Clostridia;o__Clostridiales;f__Ruminococcaceae;g__;s__	7.21E-06	3.30E+00	6.97E+00
297576	k__Bacteria;p__Bacteroidetes;c__Bacteroidia;o__Bacteroidales;f__;g__;s__	7.25E-06	1.83E-01	1.69E+00
New.ReferenceOTU187	k__Bacteria;p__Verrucomicrobia;c__Verruco-5;o__WCHB1-41;f__RFP12;g__;s__	7.33E-06	7.46E-01	2.64E+00
320847	k__Bacteria;p__Bacteroidetes;c__Bacteroidia;o__Bacteroidales;f__;g__;s__	7.44E-06	1.52E+00	4.13E+00
290804	k__Bacteria;p__Actinobacteria;c__Coriobacteriia;o__Coriobacteriales;f__Coriobacteriaceae;g__;s__	7.56E-06	1.10E+01	9.85E-01
301640	k__Bacteria;p__Firmicutes;c__Clostridia;o__Clostridiales;f__;g__;s__	8.19E-06	2.52E+00	1.07E+01
348065	k__Bacteria;p__Firmicutes;c__Clostridia;o__Clostridiales;f__;g__;s__	8.25E-06	1.83E+00	1.49E-01
110579	k__Bacteria;p__Firmicutes;c__Clostridia;o__Clostridiales;f__[Mogibacteriaceae];g__;s__	8.35E-06	1.41E+00	4.48E-02
192244	k__Bacteria;p__Firmicutes;c__Clostridia;o__Clostridiales;f__Ruminococcaceae;g__;s__	8.38E-06	1.94E+00	2.99E-01
133892	k__Bacteria;p__Firmicutes;c__Bacilli;o__Lactobacillales;f__Lactobacillaceae;g__Lactobacillus;s__	8.46E-06	2.72E+00	2.24E-01
191476	k__Bacteria;p__Firmicutes;c__Clostridia;o__Clostridiales;f__Lachnospiraceae;g__[Ruminococcus];s__gnavus	8.84E-06	2.70E+00	1.49E-01

New.ReferenceOTU168	k__Bacteria;p__Firmicutes;c__Clostridia;o__Clostridiales;f__Ruminococcaceae;g__;s__	9.11E-06	1.83E-01	1.16E+00
295444	k__Bacteria;p__Firmicutes;c__Clostridia;o__Clostridiales;f__Lachnospiraceae;g__;s__	9.28E-06	2.54E-01	3.45E+00
330285	k__Bacteria;p__Firmicutes;c__Clostridia;o__Clostridiales;f__Ruminococcaceae;g__;s__	9.45E-06	4.80E+00	1.13E+00
New.ReferenceOTU651	k__Bacteria;p__Firmicutes;c__Clostridia;o__Clostridiales;f__Lachnospiraceae;g__;s__	9.58E-06	8.31E-01	2.39E+00
346457	k__Bacteria;p__Firmicutes;c__Clostridia;o__Clostridiales;f__;g__;s__	9.60E-06	3.10E-01	1.54E+00
619919	k__Bacteria;p__Firmicutes;c__Clostridia;o__Clostridiales;f__;g__;s__	9.60E-06	2.92E+00	6.45E+00
17311	k__Bacteria;p__Firmicutes;c__Clostridia;o__Clostridiales;f__Christensenellaceae;g__;s__	9.66E-06	1.32E+00	1.34E-01
New.CleanUp.ReferenceOTU127362	k__Bacteria;p__Firmicutes;c__Clostridia;o__Clostridiales;f__Christensenellaceae;g__;s__	9.73E-06	9.58E-01	4.48E-02
652823	k__Bacteria;p__Firmicutes;c__Clostridia;o__Clostridiales;f__Ruminococcaceae;g__;s__	9.80E-06	4.79E+00	3.58E-01
299047	k__Bacteria;p__Firmicutes;c__Clostridia;o__Clostridiales;f__Dehalobacteriaceae;g__;s__	9.80E-06	1.51E+01	2.45E+01
3943182	k__Bacteria;p__Bacteroidetes;c__Bacteroidia;o__Bacteroidales;f__Bacteroidaceae;g__Bacteroides;s__	9.80E-06	1.87E+00	5.97E-02
332860	k__Bacteria;p__Firmicutes;c__Clostridia;o__Clostridiales;f__Ruminococcaceae;g__;s__	1.01E-05	2.39E-01	1.51E+00
232222	k__Bacteria;p__Firmicutes;c__Clostridia;o__Clostridiales;f__Ruminococcaceae;g__;s__	1.01E-05	2.49E+00	1.49E-01
New.ReferenceOTU807	k__Bacteria;p__Firmicutes;c__Clostridia;o__Clostridiales;f__Ruminococcaceae;g__;s__	1.09E-05	2.82E-02	3.10E+00
352192	k__Bacteria;p__Firmicutes;c__Clostridia;o__Clostridiales;f__;g__;s__	1.10E-05	4.23E-02	3.51E+00
336874	k__Bacteria;p__Bacteroidetes;c__Bacteroidia;o__Bacteroidales;f__;g__;s__	1.11E-05	1.20E+00	3.43E+00
555595	k__Bacteria;p__Firmicutes;c__Clostridia;o__Clostridiales;f__Ruminococcaceae;g__;s__	1.11E-05	2.76E+00	7.70E+00
316357	k__Bacteria;p__Firmicutes;c__Clostridia;o__Clostridiales;f__Lachnospiraceae;g__;s__	1.14E-05	3.38E-01	2.60E+00
99173	k__Bacteria;p__Firmicutes;c__Clostridia;o__Clostridiales;f__Lachnospiraceae;g__Coprococcus;s__	1.16E-05	1.38E+00	7.10E+00
197072	k__Bacteria;p__Bacteroidetes;c__Bacteroidia;o__Bacteroidales;f__Bacteroidaceae;g__Bacteroides;s__	1.17E-05	1.61E+00	2.84E-01
350224	k__Bacteria;p__Firmicutes;c__Clostridia;o__Clostridiales;f__Ruminococcaceae;g__Ruminococcus;s__flavefaciens	1.22E-05	1.59E+00	0.00E+00
New.ReferenceOTU262	k__Bacteria;p__Bacteroidetes;c__Bacteroidia;o__Bacteroidales;f__;g__;s__	1.27E-05	1.97E-01	1.42E+00
297914	k__Bacteria;p__Bacteroidetes;c__Bacteroidia;o__Bacteroidales;f__;g__;s__	1.27E-05	2.11E-01	1.16E+00
New.CleanUp.ReferenceOTU158319	k__Bacteria;p__Firmicutes;c__Clostridia;o__Clostridiales;f__Ruminococcaceae;g__Ruminococcus;s__	1.28E-05	1.69E-01	1.72E+00
2235671	k__Bacteria;p__Firmicutes;c__Clostridia;o__Clostridiales;f__Lachnospiraceae;g__Blautia;s__producta	1.29E-05	2.25E+00	1.49E-02
New.CleanUp.ReferenceOTU6013	k__Bacteria;p__Bacteroidetes;c__Bacteroidia;o__Bacteroidales;f__;g__;s__	1.32E-05	2.11E-01	1.12E+00
325850	k__Bacteria;p__Proteobacteria;c__Alphaproteobacteria;o__RF32;f__;g__;s__	1.39E-05	4.11E+00	3.58E-01
4427335	k__Bacteria;p__Firmicutes;c__Clostridia;o__Clostridiales;f__;g__;s__	1.40E-05	2.68E-01	2.90E+00
569824	k__Bacteria;p__Firmicutes;c__Clostridia;o__Clostridiales;f__Ruminococcaceae;g__;s__	1.40E-05	5.92E-01	2.24E+00
New.ReferenceOTU787	k__Bacteria;p__Verrucomicrobia;c__Verruco-5;o__WCHB1-41;f__RFP12;g__;s__	1.44E-05	1.52E+00	4.39E+00
665703	k__Bacteria;p__Firmicutes;c__Clostridia;o__Clostridiales;f__;g__;s__	1.44E-05	7.76E+00	7.61E-01
New.CleanUp.ReferenceOTU96704	k__Bacteria;p__Firmicutes;c__Clostridia;o__Clostridiales;f__;g__;s__	1.49E-05	2.14E+00	4.18E-01

287496	k__Bacteria;p__Firmicutes;c__Clostridia;o__Clostridiales;f__Ruminococcaceae;g__;s__	1.50E-05	1.70E+00	5.49E+00
4395096	k__Bacteria;p__Bacteroidetes;c__Bacteroidia;o__Bacteroidales;f__Bacteroidaceae;g__Bacteroides;s__ovatus	1.52E-05	1.34E+00	2.99E-01
918313	k__Bacteria;p__Firmicutes;c__Clostridia;o__Clostridiales;f__;g__;s__	1.59E-05	9.86E-02	1.94E+00
New.ReferenceOTU868	k__Bacteria;p__Bacteroidetes;c__Bacteroidia;o__Bacteroidales;f__;g__;s__	1.59E-05	2.39E-01	1.87E+00
837396	k__Bacteria;p__Firmicutes;c__Clostridia;o__Clostridiales;f__;g__;s__	1.62E-05	1.10E+00	4.43E+00
299778	k__Bacteria;p__Bacteroidetes;c__Bacteroidia;o__Bacteroidales;f__Porphyromonadaceae;g__Paludibacter;s__	1.63E-05	2.11E-01	2.39E+00
336836	k__Bacteria;p__Firmicutes;c__Clostridia;o__Clostridiales;f__;g__;s__	1.64E-05	1.38E+01	2.09E+01
91359	k__Bacteria;p__Firmicutes;c__Clostridia;o__Clostridiales;f__Lachnospiraceae;g__Lachnospira;s__	1.66E-05	3.06E+00	8.96E-02
105434	k__Bacteria;p__Firmicutes;c__Clostridia;o__Clostridiales;f__;g__;s__	1.67E-05	3.52E-01	1.63E+00
New.ReferenceOTU143	k__Bacteria;p__Bacteroidetes;c__Bacteroidia;o__Bacteroidales;f__;g__;s__	1.69E-05	5.63E-02	1.00E+00
2985051	k__Bacteria;p__Actinobacteria;c__Coriobacteriia;o__Coriobacteriales;f__Coriobacteriaceae;g__;s__	1.69E-05	2.35E+00	1.34E-01
New.CleanUp.ReferenceOTU20812	k__Bacteria;p__Firmicutes;c__Clostridia;o__Clostridiales;f__Ruminococcaceae;g__;s__	1.72E-05	1.83E-01	7.46E-01
111771	k__Bacteria;p__Firmicutes;c__Clostridia;o__Clostridiales;f__;g__;s__	1.72E-05	1.32E+00	1.49E-01
208802	k__Bacteria;p__Firmicutes;c__Clostridia;o__Clostridiales;f__Christensenellaceae;g__;s__	1.73E-05	1.24E+01	6.69E+00
36226	k__Bacteria;p__Firmicutes;c__Clostridia;o__Clostridiales;f__Clostridiaceae;g__;s__	1.74E-05	9.10E+00	1.69E+00
New.ReferenceOTU481	k__Bacteria;p__Firmicutes;c__Clostridia;o__Clostridiales;f__[Mogibacteriaceae];g__;s__	1.80E-05	2.86E+00	2.09E-01
300646	k__Bacteria;p__Firmicutes;c__Clostridia;o__Clostridiales;f__;g__;s__	1.81E-05	1.23E+00	3.99E+00
819353	k__Bacteria;p__Firmicutes;c__Clostridia;o__Clostridiales;f__Ruminococcaceae;g__;s__	1.81E-05	1.06E+01	3.25E+00
289070	k__Bacteria;p__Spirochaetes;c__Spirochaetes;o__Spirochaetales;f__Spirochaetaceae;g__Treponema;s__	1.81E-05	3.94E-01	2.19E+00
110060	k__Bacteria;p__Firmicutes;c__Clostridia;o__Clostridiales;f__Ruminococcaceae;g__;s__	1.81E-05	4.30E+00	9.10E-01
308884	k__Bacteria;p__Firmicutes;c__Clostridia;o__Clostridiales;f__;g__;s__	1.92E-05	1.41E-02	1.51E+00
317515	k__Bacteria;p__Firmicutes;c__Clostridia;o__Clostridiales;f__;g__;s__	1.96E-05	7.00E+00	3.03E+00
178845	k__Bacteria;p__Firmicutes;c__Clostridia;o__Clostridiales;f__Ruminococcaceae;g__;s__	1.96E-05	1.18E+01	7.61E-01
327635	k__Bacteria;p__Firmicutes;c__Clostridia;o__Clostridiales;f__;g__;s__	1.97E-05	5.78E+01	8.54E+01
New.ReferenceOTU783	k__Bacteria;p__WPS-2;c__;o__;f__;g__;s__	1.98E-05	1.41E-02	3.61E+00
287689	k__Bacteria;p__Firmicutes;c__Clostridia;o__Clostridiales;f__Lachnospiraceae;g__;s__	1.99E-05	2.25E-01	1.76E+00
301381	k__Bacteria;p__Spirochaetes;c__Spirochaetes;o__Spirochaetales;f__Spirochaetaceae;g__Treponema;s__	2.05E-05	1.46E+00	1.17E+01
4397737	k__Bacteria;p__Firmicutes;c__Clostridia;o__Clostridiales;f__Lachnospiraceae;g__Blautia	2.11E-05	1.99E+00	0.00E+00
New.ReferenceOTU749	k__Bacteria;p__Firmicutes;c__Clostridia;o__Clostridiales;f__;g__;s__	2.15E-05	5.49E+00	1.75E+00
4331760	k__Bacteria;p__Bacteroidetes;c__Bacteroidia;o__Bacteroidales;f__Rikenellaceae;g__;s__	2.17E-05	2.21E+00	2.09E-01
New.CleanUp.ReferenceOTU154152	k__Bacteria;p__Firmicutes;c__Clostridia;o__Clostridiales;f__;g__;s__	2.17E-05	3.11E+00	0.00E+00
306785	k__Bacteria;p__Firmicutes;c__Clostridia;o__Clostridiales;f__;g__;s__	2.19E-05	2.10E+00	4.94E+00

365574	k__Bacteria;p__Firmicutes;c__Clostridia;o__Clostridiales;f__Lachnospiraceae;g__Blautia;s__	2.22E-05	2.82E-01	1.57E+00
New.ReferenceOTU906	k__Bacteria;p__Firmicutes;c__Clostridia;o__Clostridiales;f__;g__;s__	2.22E-05	1.83E+00	6.33E+00
New.CleanUp.ReferenceOTU125682	k__Bacteria;p__Bacteroidetes;c__Bacteroidia;o__Bacteroidales;f__;g__;s__	2.22E-05	4.23E-02	1.57E+00
295654	k__Bacteria;p__Firmicutes;c__Bacilli;o__Lactobacillales;f__Lactobacillaceae;g__Lactobacillus;s__	2.22E-05	1.20E+01	3.28E+00
342703	k__Bacteria;p__Firmicutes;c__Clostridia;o__Clostridiales;f__Ruminococcaceae;g__;s__	2.24E-05	4.35E+00	2.99E-02
1066830	k__Bacteria;p__Firmicutes;c__Clostridia;o__Clostridiales;f__Ruminococcaceae;g__;s__	2.25E-05	1.80E+01	4.25E+00
4464445	k__Bacteria;p__Firmicutes;c__Clostridia;o__Clostridiales;f__Lachnospiraceae;g__;s__	2.29E-05	1.07E+00	1.49E-02
347783	k__Bacteria;p__Actinobacteria;c__Coriobacteriia;o__Coriobacteriales;f__Coriobacteriaceae;g__Slackia;s__	2.30E-05	5.05E+01	1.23E+01
New.ReferenceOTU180	k__Bacteria;p__Firmicutes;c__Clostridia;o__Clostridiales;f__;g__;s__	2.42E-05	2.96E-01	1.67E+00
292493	k__Bacteria;p__Firmicutes;c__Clostridia;o__Clostridiales;f__;g__;s__	2.42E-05	5.37E+01	9.64E+00
300038	k__Bacteria;p__Verrucomicrobia;c__Verruco-5;o__WCHB1-41;f__RFP12;g__;s__	2.48E-05	7.04E-02	1.21E+00
1140223	k__Bacteria;p__Firmicutes;c__Bacilli;o__Lactobacillales;f__Streptococcaceae;g__Streptococcus;s__	2.49E-05	8.66E+00	1.82E+00
4307897	k__Bacteria;p__Firmicutes;c__Bacilli;o__Lactobacillales;f__Streptococcaceae;g__Streptococcus;s__	2.52E-05	6.63E+00	2.09E+01
331392	k__Bacteria;p__Firmicutes;c__Clostridia;o__Clostridiales;f__Clostridiaceae;g__Clostridium;s__	2.56E-05	1.72E+00	1.35E+01
291431	k__Bacteria;p__Firmicutes;c__Erysipelotrichi;o__Erysipelotrichales;f__Erysipelotrichaceae;g__p-75-a5;s__	2.57E-05	2.11E-01	1.34E+00
301498	k__Bacteria;p__Firmicutes;c__Clostridia;o__Clostridiales;f__Lachnospiraceae;g__;s__	2.60E-05	7.27E+00	8.66E-01
289493	k__Bacteria;p__Firmicutes;c__Clostridia;o__Clostridiales;f__Lachnospiraceae;g__;s__	2.68E-05	7.04E-02	1.00E+00
New.CleanUp.ReferenceOTU49837	k__Bacteria;p__Firmicutes;c__Clostridia;o__Clostridiales;f__Ruminococcaceae;g__;s__	2.75E-05	2.82E-02	5.22E-01
New.ReferenceOTU55	k__Bacteria;p__Firmicutes;c__Clostridia;o__Clostridiales;f__Lachnospiraceae;g__Coprococcus;s__	2.90E-05	1.77E+00	5.82E-01
649107	k__Bacteria;p__Firmicutes;c__Clostridia;o__Clostridiales;f__;g__;s__	2.94E-05	1.31E+01	1.69E+01
798164	k__Bacteria;p__Firmicutes;c__Clostridia;o__Clostridiales;f__;g__;s__	2.95E-05	1.13E-01	1.49E+00
New.ReferenceOTU395	k__Bacteria;p__Bacteroidetes;c__Bacteroidia;o__Bacteroidales;f__Prevotellaceae;g__Prevotella;s__	3.01E-05	3.82E+00	1.79E-01
298108	k__Bacteria;p__Firmicutes;c__Clostridia;o__Clostridiales;f__Lachnospiraceae;g__;s__	3.13E-05	4.79E-01	2.93E+00
293682	k__Bacteria;p__Verrucomicrobia;c__Verruco-5;o__WCHB1-41;f__RFP12;g__;s__	3.16E-05	8.45E-01	2.90E+00
New.ReferenceOTU64	k__Bacteria;p__Firmicutes;c__Clostridia;o__Clostridiales;f__;g__;s__	3.20E-05	5.92E-01	8.57E+00
328238	k__Bacteria;p__Firmicutes;c__Clostridia;o__Clostridiales;f__;g__;s__	3.25E-05	5.92E-01	2.28E+00
4256470	k__Bacteria;p__Bacteroidetes;c__Bacteroidia;o__Bacteroidales;f__Bacteroidaceae;g__Bacteroides;s__	3.37E-05	5.96E+00	1.49E+00
189920	k__Bacteria;p__Bacteroidetes;c__Bacteroidia;o__Bacteroidales;f__Bacteroidaceae;g__Bacteroides;s__	3.37E-05	2.65E+00	6.72E-01
292128	k__Bacteria;p__Firmicutes;c__Clostridia;o__Clostridiales;f__[Mogibacteriaceae];g__Mogibacterium;s__	3.41E-05	4.23E+01	7.10E+01
185884	k__Bacteria;p__Firmicutes;c__Clostridia;o__Clostridiales;f__Lachnospiraceae;g__Blautia;s__producta	3.55E-05	1.08E+00	1.79E-01
331116	k__Bacteria;p__Firmicutes;c__Clostridia;o__Clostridiales;f__Lachnospiraceae;g__;s__	3.68E-05	2.49E+00	4.33E-01
563591	k__Bacteria;p__Firmicutes;c__Clostridia;o__Clostridiales;f__Lachnospiraceae;g__;s__	3.70E-05	0.00E+00	3.40E+00

New.CleanUp.ReferenceOTU104722	k__Bacteria;p__Firmicutes;c__Clostridia;o__Clostridiales;f__;g__;s__	3.76E-05	0.00E+00	1.36E+00
295527	k__Bacteria;p__Firmicutes;c__Clostridia;o__Clostridiales;f__;g__;s__	3.82E-05	9.87E+00	2.25E+01
New.ReferenceOTU175	k__Bacteria;p__Firmicutes;c__Clostridia;o__Clostridiales;f__Lachnospiraceae;g__Coprococcus;s__	3.88E-05	1.68E+00	1.64E-01
351498	k__Bacteria;p__Firmicutes;c__Clostridia;o__Clostridiales;f__[Mogibacteriaceae];g__;s__	4.00E-05	4.65E-01	2.88E+00
359533	k__Bacteria;p__Actinobacteria;c__Actinobacteria;o__Actinomycetales;f__Corynebacteriaceae;g__Corynebacterium;s__	4.01E-05	2.68E+00	1.94E-01
New.CleanUp.ReferenceOTU50244	k__Bacteria;p__Firmicutes;c__Clostridia;o__Clostridiales	4.01E-05	7.75E-01	0.00E+00
337167	k__Bacteria;p__Firmicutes;c__Clostridia;o__Clostridiales;f__Ruminococcaceae;g__;s__	4.03E-05	3.13E+00	7.67E+00
335759	k__Bacteria;p__Firmicutes;c__Clostridia;o__Clostridiales;f__Clostridiaceae;g__;s__	4.04E-05	1.62E+00	3.54E+00
New.ReferenceOTU96	k__Bacteria;p__Fusobacteria;c__Fusobacteriia;o__Fusobacteriales;f__Fusobacteriaceae;g__Fusobacterium;s__	4.04E-05	2.03E+00	8.12E+00
4060501	k__Bacteria;p__Firmicutes;c__Clostridia;o__Clostridiales;f__Peptococcaceae;g__;s__	4.04E-05	1.04E+00	0.00E+00
221429	k__Bacteria;p__Firmicutes;c__Clostridia;o__Clostridiales;f__Ruminococcaceae;g__;s__	4.09E-05	6.90E-01	2.69E+00
290240	k__Bacteria;p__Firmicutes;c__Clostridia;o__Clostridiales;f__Lachnospiraceae;g__Epulopiscium;s__	4.11E-05	1.11E+00	3.00E+01
592616	k__Bacteria;p__Firmicutes;c__Erysipelotrichi;o__Erysipelotrichales;f__Erysipelotrichaceae;g__;s__	4.11E-05	2.59E+00	0.00E+00
4296211	k__Bacteria;p__Firmicutes;c__Clostridia;o__Clostridiales;f__Lachnospiraceae;g__Blautia;s__	4.11E-05	4.75E+00	5.37E-01
4410401	k__Bacteria;p__Firmicutes;c__Clostridia;o__Clostridiales;f__Veillonellaceae;g__Veillonella;s__parvula	4.14E-05	9.85E+00	4.63E-01
New.ReferenceOTU29	k__Bacteria;p__Firmicutes;c__Clostridia;o__Clostridiales;f__Christensenellaceae;g__;s__	4.14E-05	6.48E-01	2.00E+00
188610	k__Bacteria;p__Firmicutes;c__Clostridia;o__Clostridiales;f__Lachnospiraceae;g__Blautia;s__producta	4.18E-05	7.89E-01	2.99E-02
299818	k__Bacteria;p__Firmicutes;c__Clostridia;o__Clostridiales;f__Lachnospiraceae;g__;s__	4.20E-05	1.80E+00	1.04E-01
293697	k__Bacteria;p__Firmicutes;c__Clostridia;o__Clostridiales;f__Clostridiaceae;g__;s__	4.24E-05	4.65E-01	2.66E+00
New.ReferenceOTU188	k__Bacteria;p__Firmicutes;c__Clostridia;o__Clostridiales;f__Ruminococcaceae;g__;s__	4.26E-05	2.35E+00	6.16E+00
New.ReferenceOTU645	k__Bacteria;p__Bacteroidetes;c__Bacteroidia;o__Bacteroidales;f__;g__;s__	4.29E-05	3.24E-01	2.90E+00
369705	k__Bacteria;p__Firmicutes;c__Bacilli;o__Bacillales;f__Staphylococcaceae;g__Jeotgalicoccus;s__	4.35E-05	1.38E+00	1.49E-02
1108747	k__Bacteria;p__Tenericutes;c__Mollicutes;o__RF39;f__;g__;s__	4.36E-05	2.11E+00	1.19E-01
159711	k__Bacteria;p__Proteobacteria;c__Gammaproteobacteria;o__Pseudomonadales;f__Moraxellaceae;g__Acinetobacter;s__lwoffii	4.46E-05	2.82E+02	1.38E+02
176318	k__Bacteria;p__Firmicutes;c__Clostridia;o__Clostridiales;f__Christensenellaceae;g__;s__	4.51E-05	8.64E+01	4.28E+01
25534	k__Bacteria;p__Firmicutes;c__Clostridia;o__Clostridiales;f__Lachnospiraceae;g__;s__	4.53E-05	4.52E+00	2.45E+00
297116	k__Bacteria;p__Firmicutes;c__Clostridia;o__Clostridiales;f__Ruminococcaceae;g__;s__	4.80E-05	1.10E+00	3.48E+00
4238405	k__Bacteria;p__Firmicutes;c__Clostridia;o__Clostridiales;f__;g__;s__	4.84E-05	3.08E+00	5.39E+00
109733	k__Bacteria;p__Firmicutes;c__Clostridia;o__Clostridiales;f__;g__;s__	5.15E-05	1.00E+00	2.93E+00
301331	k__Bacteria;p__Bacteroidetes;c__Bacteroidia;o__Bacteroidales;f__;g__;s__	5.34E-05	7.04E-02	1.69E+00
New.CleanUp.ReferenceOTU3961	k__Bacteria;p__Firmicutes;c__Clostridia;o__Clostridiales;f__Christensenellaceae;g__;s__	5.42E-05	9.86E-02	1.01E+00
277425	k__Bacteria;p__Firmicutes;c__Clostridia;o__Clostridiales;f__;g__;s__	5.43E-05	1.55E+00	5.61E+00

522353	k__Bacteria;p__Firmicutes;c__Erysipelotrichi;o__Erysipelotrichales;f__Erysipelotrichaceae;g__Coprobacillus;s__	5.51E-05	1.52E+00	2.99E-01
180468	k__Bacteria;p__Firmicutes;c__Clostridia;o__Clostridiales;f__Ruminococcaceae;g__Oscillospira;s__	5.51E-05	1.35E+00	2.69E-01
313967	k__Bacteria;p__Verrucomicrobia;c__Verruco-5;o__WCHB1-41;f__RFP12;g__;s__	5.54E-05	1.41E-01	1.79E+00
4464173	k__Bacteria;p__Firmicutes;c__Clostridia;o__Clostridiales;f__Lachnospiraceae;g__;s__	5.62E-05	8.63E+00	1.67E+00
New.ReferenceOTU946	k__Bacteria;p__Bacteroidetes;c__Bacteroidia;o__Bacteroidales;f__[Odoribacteraceae];g__Odoribacter;s__	5.75E-05	3.97E+00	8.21E-01
4310208	k__Bacteria;p__Firmicutes;c__Clostridia;o__Clostridiales;f__Veillonellaceae;g__Veillonella;s__parvula	5.82E-05	4.07E+00	4.03E-01
2177996	k__Bacteria;p__Firmicutes;c__Clostridia;o__Clostridiales;f__Lachnospiraceae;g__;s__	5.93E-05	2.75E+00	1.94E-01
4315790	k__Bacteria;p__Firmicutes;c__Clostridia;o__Clostridiales;f__Ruminococcaceae;g__;s__	6.12E-05	3.66E-01	1.75E+00
812475	k__Bacteria;p__Bacteroidetes;c__Bacteroidia;o__Bacteroidales;f__;g__;s__	6.12E-05	2.68E-01	1.43E+00
New.CleanUp.ReferenceOTU33321	k__Bacteria;p__Firmicutes;c__Clostridia;o__Clostridiales;f__Ruminococcaceae;g__;s__	6.24E-05	1.13E-01	1.24E+00
197988	k__Bacteria;p__Firmicutes;c__Clostridia;o__Clostridiales;f__Ruminococcaceae;g__Oscillospira;s__	6.27E-05	1.06E+01	2.45E+00
295333	k__Bacteria;p__Firmicutes;c__Clostridia;o__Clostridiales;f__Lachnospiraceae;g__;s__	6.27E-05	2.68E-01	8.66E-01
198930	k__Bacteria;p__Firmicutes;c__Clostridia;o__Clostridiales;f__Lachnospiraceae;g__Roseburia;s__	6.36E-05	1.87E+00	2.54E-01
293304	k__Bacteria;p__Bacteroidetes;c__Bacteroidia;o__Bacteroidales;f__;g__;s__	6.45E-05	6.48E-01	1.84E+00
4318194	k__Bacteria;p__Firmicutes;c__Clostridia;o__Clostridiales;f__;g__;s__	6.46E-05	4.08E-01	1.70E+00
New.ReferenceOTU5	k__Bacteria;p__Bacteroidetes;c__Bacteroidia;o__Bacteroidales;f__;g__;s__	6.53E-05	5.63E-02	7.16E-01
New.ReferenceOTU970	k__Bacteria;p__Firmicutes;c__Clostridia;o__Clostridiales;f__Christensenellaceae;g__;s__	6.53E-05	1.32E+00	1.34E-01
349572	k__Bacteria;p__Firmicutes;c__Clostridia;o__Clostridiales;f__Lachnospiraceae;g__;s__	6.59E-05	2.54E-01	1.76E+00
New.CleanUp.ReferenceOTU83830	k__Bacteria;p__Firmicutes;c__Clostridia;o__Clostridiales;f__Ruminococcaceae;g__;s__	6.71E-05	8.45E-02	6.72E-01
290322	k__Bacteria;p__Firmicutes;c__Clostridia;o__Clostridiales;f__Ruminococcaceae;g__;s__	6.71E-05	8.59E-01	3.36E+00
574421	k__Bacteria;p__Firmicutes;c__Clostridia;o__Clostridiales;f__Ruminococcaceae;g__Ruminococcus;s__	6.82E-05	3.42E+00	1.26E+01
302909	k__Bacteria;p__Firmicutes;c__Clostridia;o__Clostridiales;f__Lachnospiraceae;g__;s__	6.82E-05	5.25E+00	1.12E+01
110829	k__Bacteria;p__Firmicutes;c__Clostridia;o__Clostridiales;f__;g__;s__	6.87E-05	3.77E+00	6.79E+00
4433947	k__Bacteria;p__Bacteroidetes;c__Bacteroidia;o__Bacteroidales;f__Bacteroidaceae;g__Bacteroides;s__	6.94E-05	7.90E+00	1.58E+00
299858	k__Bacteria;p__Firmicutes;c__Clostridia;o__Clostridiales;f__;g__;s__	7.08E-05	1.37E+00	0.00E+00
289454	k__Bacteria;p__Firmicutes;c__Clostridia;o__Clostridiales;f__Christensenellaceae;g__;s__	7.10E-05	9.86E-02	1.36E+00
320207	k__Bacteria;p__Firmicutes;c__Clostridia;o__Clostridiales;f__Ruminococcaceae;g__;s__	7.18E-05	2.78E+01	4.39E+01
541334	k__Bacteria;p__Firmicutes;c__Clostridia;o__Clostridiales;f__Ruminococcaceae;g__Ruminococcus	7.22E-05	2.15E+00	1.00E+01
1041094	k__Bacteria;p__Firmicutes;c__Clostridia;o__Clostridiales;f__;g__;s__	7.23E-05	1.13E-01	1.63E+00
319788	k__Bacteria;p__Bacteroidetes;c__Bacteroidia;o__Bacteroidales;f__;g__;s__	7.29E-05	0.00E+00	6.27E-01
306306	k__Bacteria;p__Firmicutes;c__Bacilli;o__Lactobacillales;f__Lactobacillaceae;g__Lactobacillus;s__	7.33E-05	5.80E+00	1.43E+00
4347265	k__Bacteria;p__Firmicutes;c__Clostridia;o__Clostridiales;f__Clostridiaceae;g__;s__	7.33E-05	1.05E+01	4.07E+00

New.CleanUp.ReferenceOTU174844	k__Bacteria;p__Bacteroidetes;c__Bacteroidia;o__Bacteroidales	7.39E-05	2.11E-01	2.07E+00
346974	k__Bacteria;p__Firmicutes;c__Clostridia;o__Clostridiales;f__[Mogibacteriaceae];g__;s__	7.64E-05	6.47E+01	1.35E+02
New.ReferenceOTU584	k__Bacteria;p__Firmicutes;c__Clostridia;o__Clostridiales;f__;g__;s__	7.66E-05	1.65E+00	2.96E+00
212686	k__Bacteria;p__Firmicutes;c__Clostridia;o__Clostridiales;f__Ruminococcaceae;g__Oscillospira;s__	7.84E-05	1.80E+00	1.49E-02
313069	k__Bacteria;p__Firmicutes;c__Clostridia;o__Clostridiales;f__Clostridiaceae;g__;s__	8.22E-05	6.76E-01	2.99E-02
271602	k__Bacteria;p__Firmicutes;c__Clostridia;o__Clostridiales;f__;g__;s__	8.29E-05	4.17E+00	4.48E-01
310679	k__Bacteria;p__Bacteroidetes;c__Bacteroidia;o__Bacteroidales;f__;g__;s__	8.31E-05	7.45E+00	1.29E+01
321290	k__Bacteria;p__Firmicutes;c__Clostridia;o__Clostridiales;f__Lachnospiraceae;g__;s__	8.36E-05	9.15E-01	9.88E+00
186352	k__Bacteria;p__Bacteroidetes;c__Bacteroidia;o__Bacteroidales;f__Bacteroidaceae;g__Bacteroides;s__	8.55E-05	1.15E+00	1.19E-01
4324985	k__Bacteria;p__Firmicutes;c__Clostridia;o__Clostridiales;f__Ruminococcaceae;g__;s__	8.57E-05	2.77E+00	6.96E+00
3256931	k__Bacteria;p__Bacteroidetes;c__Bacteroidia;o__Bacteroidales;f__Bacteroidaceae;g__Bacteroides;s__	8.61E-05	9.72E-01	1.49E-01
New.ReferenceOTU980	k__Bacteria;p__Firmicutes;c__Clostridia;o__Clostridiales;f__Eubacteriaceae;g__Pseudoramibacter_Eubacterium;s__	8.73E-05	8.87E-01	4.15E+00
331575	k__Bacteria;p__Firmicutes;c__Clostridia;o__Clostridiales;f__Lachnospiraceae;g__Coprococcus;s__	8.90E-05	5.21E-01	1.69E+00
296406	k__Bacteria;p__Spirochaetes;c__Spirochaetes;o__Spirochaetales;f__Spirochaetaceae;g__Treponema;s__	9.02E-05	1.13E-01	8.21E-01
New.ReferenceOTU954	k__Bacteria;p__Firmicutes;c__Clostridia;o__Clostridiales;f__Lachnospiraceae;g__;s__	9.37E-05	2.11E-01	1.07E+00
2203165	k__Bacteria;p__Firmicutes;c__Clostridia;o__Clostridiales;f__Ruminococcaceae;g__Oscillospira;s__	9.37E-05	8.31E-01	7.46E-02
New.ReferenceOTU953	Unassigned	9.51E-05	2.62E+00	1.49E-01
New.CleanUp.ReferenceOTU79061	k__Bacteria;p__Verrucomicrobia;c__Verruco-5;o__WCHB1-41;f__RFP12;g__;s__	9.58E-05	2.11E-01	9.10E-01
New.ReferenceOTU499	k__Bacteria;p__Firmicutes;c__Clostridia;o__Clostridiales;f__Ruminococcaceae;g__Ruminococcus;s__	9.69E-05	3.94E-01	3.21E+00
New.ReferenceOTU770	k__Bacteria;p__Bacteroidetes;c__Bacteroidia;o__Bacteroidales;f__;g__;s__	9.77E-05	2.96E-01	2.61E+00
178304	k__Bacteria;p__Firmicutes;c__Clostridia;o__Clostridiales;f__Lachnospiraceae;g__;s__	9.81E-05	2.94E+00	7.46E-02
263546	k__Bacteria;p__Firmicutes;c__Clostridia;o__Clostridiales;f__Ruminococcaceae;g__Oscillospira;s__	1.01E-04	4.82E+00	1.84E+00
New.ReferenceOTU912	k__Archaea;p__Euryarchaeota;c__Methanobacteria;o__Methanobacteriales;f__Methanobacteriaceae;g__Methanobrevibacter;s__	1.04E-04	1.86E+00	4.16E+00
175485	k__Bacteria;p__Bacteroidetes;c__Bacteroidia;o__Bacteroidales;f__Bacteroidaceae;g__Bacteroides;s__fragilis	1.05E-04	1.90E+00	2.39E-01
4468466	k__Bacteria;p__Firmicutes;c__Clostridia;o__Clostridiales;f__Ruminococcaceae;g__;s__	1.06E-04	1.55E+01	4.07E+00
New.ReferenceOTU552	k__Bacteria;p__Firmicutes;c__Clostridia;o__Clostridiales;f__Ruminococcaceae;g__;s__	1.11E-04	8.87E-01	2.21E+00
New.ReferenceOTU795	k__Bacteria;p__Firmicutes;c__Clostridia;o__Clostridiales;f__[Mogibacteriaceae];g__Anaerovorax;s__	1.11E-04	3.38E+00	3.58E-01
4404830	k__Bacteria;p__Firmicutes;c__Clostridia;o__Clostridiales;f__Ruminococcaceae;g__;s__	1.12E-04	2.30E+00	4.48E-02
298624	k__Bacteria;p__Firmicutes;c__Clostridia;o__Clostridiales;f__Ruminococcaceae;g__;s__	1.13E-04	8.45E-02	1.00E+00
New.CleanUp.ReferenceOTU151937	k__Bacteria;p__Firmicutes;c__Clostridia;o__Clostridiales;f__Ruminococcaceae;g__;s__	1.13E-04	1.58E+00	2.84E-01
New.CleanUp.ReferenceOTU130268	k__Bacteria;p__Firmicutes;c__Clostridia;o__Clostridiales;f__Ruminococcaceae;g__;s__	1.14E-04	1.83E-01	1.54E+00
177740	k__Bacteria;p__Firmicutes;c__Clostridia;o__Clostridiales;f__Lachnospiraceae;g__Blautia;s__producta	1.16E-04	9.30E-01	5.97E-02

306368	k__Bacteria;p__Firmicutes;c__Clostridia;o__Clostridiales;f__;g__;s__	1.20E-04	1.45E+00	4.49E+00
101908	k__Bacteria;p__Spirochaetes;c__Spirochaetes;o__Spirochaetales;f__Spirochaetaceae;g__Treponema;s__	1.21E-04	1.55E-01	1.19E+00
4481195	k__Bacteria;p__Firmicutes;c__Clostridia;o__Clostridiales;f__Lachnospiraceae;g__;s__	1.25E-04	7.37E+00	1.57E+00
New.ReferenceOTU331	k__Archaea;p__Euryarchaeota;c__Methanobacteria;o__Methanobacteriales;f__Methanobacteriaceae;g__Methanobrevibacter;s__	1.25E-04	8.59E-01	1.90E+00
290275	k__Bacteria;p__Bacteroidetes;c__Bacteroidia;o__Bacteroidales;f__;g__;s__	1.27E-04	1.70E+00	5.66E+00
185299	k__Bacteria;p__Firmicutes;c__Clostridia;o__Clostridiales;f__Lachnospiraceae	1.28E-04	7.41E+00	2.07E+01
311952	k__Bacteria;p__Firmicutes;c__Clostridia;o__Clostridiales;f__Lachnospiraceae;g__;s__	1.30E-04	7.04E-02	1.99E+00
1110312	k__Bacteria;p__Firmicutes;c__Clostridia;o__Clostridiales;f__;g__;s__	1.30E-04	1.31E+00	1.49E-01
571642	k__Bacteria;p__Firmicutes;c__Clostridia;o__Clostridiales;f__Ruminococcaceae;g__;s__	1.31E-04	5.35E-01	0.00E+00
295055	k__Bacteria;p__Firmicutes;c__Clostridia;o__Clostridiales;f__Ruminococcaceae;g__;s__	1.33E-04	3.07E+00	4.39E+00
332126	k__Bacteria;p__Firmicutes;c__Clostridia;o__Clostridiales;f__Lachnospiraceae;g__;s__	1.34E-04	9.15E-01	4.00E+00
739826	k__Bacteria;p__Firmicutes;c__Clostridia;o__Clostridiales;f__Lachnospiraceae;g__;s__	1.34E-04	9.15E-01	2.99E-02
4469576	k__Bacteria;p__Firmicutes;c__Clostridia;o__Clostridiales;f__Lachnospiraceae;g__;s__	1.35E-04	3.11E+00	1.44E+01
New.ReferenceOTU66	k__Bacteria;p__Firmicutes;c__Clostridia;o__Clostridiales;f__Clostridiaceae;g__Sarcina;s__	1.36E-04	1.69E-01	7.16E-01
104144	k__Bacteria;p__Firmicutes;c__Clostridia;o__Clostridiales;f__Ruminococcaceae;g__;s__	1.37E-04	0.00E+00	3.73E-01
295871	k__Bacteria;p__Firmicutes;c__Clostridia;o__Clostridiales;f__Ruminococcaceae;g__;s__	1.37E-04	1.30E+00	3.22E+00
New.ReferenceOTU847	k__Bacteria;p__Firmicutes;c__Clostridia;o__Clostridiales;f__Peptococcaceae;g__Desulfurispora;s__	1.38E-04	0.00E+00	4.93E-01
New.CleanUp.ReferenceOTU47030	k__Bacteria;p__Firmicutes;c__Clostridia;o__Clostridiales;f__;g__;s__	1.38E-04	5.63E-02	5.52E-01
317814	k__Bacteria;p__Firmicutes;c__Clostridia;o__Clostridiales;f__Ruminococcaceae;g__;s__	1.38E-04	1.69E-01	1.82E+00
4440670	k__Bacteria;p__Firmicutes;c__Clostridia;o__Clostridiales;f__Veillonellaceae;g__Veillonella;s__	1.40E-04	3.39E+00	2.39E-01
New.CleanUp.ReferenceOTU131784	k__Bacteria;p__Firmicutes;c__Clostridia;o__Clostridiales;f__;g__;s__	1.42E-04	1.41E-02	1.27E+00
3422630	k__Bacteria;p__Firmicutes;c__Clostridia;o__Clostridiales;f__Ruminococcaceae;g__;s__	1.43E-04	2.14E+00	3.13E-01
344636	k__Bacteria;p__Firmicutes;c__Clostridia;o__Clostridiales;f__;g__;s__	1.43E-04	2.25E-01	1.13E+00
583656	k__Bacteria;p__Bacteroidetes;c__Bacteroidia;o__Bacteroidales;f__Bacteroidaceae;g__Bacteroides;s__	1.44E-04	9.01E-01	1.49E-02
New.CleanUp.ReferenceOTU157283	k__Bacteria;p__Firmicutes;c__Clostridia;o__Clostridiales;f__Lachnospiraceae;g__;s__	1.45E-04	6.76E-01	1.49E-02
1504042	k__Bacteria;p__Firmicutes;c__Clostridia;o__Clostridiales;f__Ruminococcaceae;g__Oscillospira;s__	1.45E-04	1.11E+00	1.49E-02
New.ReferenceOTU687	k__Archaea;p__Euryarchaeota;c__Methanobacteria;o__Methanobacteriales;f__Methanobacteriaceae;g__Methanobrevibacter;s__	1.47E-04	2.00E+00	7.33E+00
349656	k__Bacteria;p__Firmicutes;c__Clostridia;o__Clostridiales;f__;g__;s__	1.48E-04	1.17E+00	1.91E+00
New.CleanUp.ReferenceOTU24282	k__Bacteria;p__Firmicutes;c__Clostridia;o__Clostridiales;f__Lachnospiraceae;g__Dorea;s__	1.48E-04	5.63E-01	1.49E-02
4315783	k__Bacteria;p__Firmicutes;c__Clostridia;o__Clostridiales;f__Ruminococcaceae;g__;s__	1.49E-04	1.97E-01	8.51E-01
3244896	k__Bacteria;p__Firmicutes;c__Bacilli;o__Lactobacillales;f__Streptococcaceae;g__Streptococcus;s__luteciae	1.50E-04	2.95E+01	1.45E+01
New.ReferenceOTU506	k__Bacteria;p__Bacteroidetes;c__Bacteroidia;o__Bacteroidales;f__;g__;s__	1.54E-04	1.41E-01	1.37E+00

342287	k__Bacteria;p__Bacteroidetes;c__Bacteroidia;o__Bacteroidales;f__RF16;g__;s__	1.57E-04	2.11E-01	2.25E+00
335268	k__Bacteria;p__Firmicutes;c__Clostridia;o__Clostridiales;f__Ruminococcaceae;g__;s__	1.58E-04	2.54E-01	3.91E+00
1103978	k__Bacteria;p__Firmicutes;c__Clostridia;o__Clostridiales;f__Lachnospiraceae;g__;s__	1.60E-04	1.32E+00	3.22E+00
191389	k__Bacteria;p__Firmicutes;c__Clostridia;o__Clostridiales;f__[Mogibacteriaceae];g__;s__	1.62E-04	1.20E+00	8.96E-02
1517774	k__Bacteria;p__Firmicutes;c__Clostridia;o__Clostridiales;f__;g__;s__	1.62E-04	5.06E+00	4.93E-01
341899	k__Bacteria;p__Firmicutes;c__Clostridia;o__Clostridiales;f__Lachnospiraceae;g__Coprococcus;s__	1.64E-04	2.77E+00	3.73E-01
4438983	k__Archaea;p__Euryarchaeota;c__Methanobacteria;o__Methanobacteriales;f__Methanobacteriaceae;g__Methanobrevibacter;s__	1.64E-04	2.57E+01	2.54E+01
15728	k__Bacteria;p__Firmicutes;c__Erysipelotrichi;o__Erysipelotrichales;f__Erysipelotrichaceae;g__Holdemania;s__	1.67E-04	8.17E-01	4.48E-02
238944	k__Bacteria;p__Firmicutes;c__Bacilli;o__Bacillales;f__Bacillaceae;g__Bacillus;s__	1.69E-04	1.77E+01	8.57E+00
287493	k__Bacteria;p__Bacteroidetes;c__Bacteroidia;o__Bacteroidales;f__;g__;s__	1.70E-04	2.25E-01	3.61E+00
299081	k__Bacteria;p__Firmicutes;c__Clostridia;o__Clostridiales;f__Ruminococcaceae;g__;s__	1.71E-04	2.62E+00	2.54E-01
298910	k__Bacteria;p__Verrucomicrobia;c__Verruco-5;o__WCHB1-41;f__RFP12;g__;s__	1.78E-04	1.27E-01	8.06E-01
2730944	k__Bacteria;p__Bacteroidetes;c__Bacteroidia;o__Bacteroidales;f__Bacteroidaceae;g__Bacteroides;s__coprophilus	1.78E-04	2.82E-02	3.37E+00
3039313	k__Bacteria;p__Firmicutes;c__Clostridia;o__Clostridiales;f__Veillonellaceae;g__Megasphaera;s__	1.79E-04	1.14E+00	4.48E-02
4414725	k__Bacteria;p__Firmicutes;c__Clostridia;o__Clostridiales;f__Lachnospiraceae;g__Blautia;s__	1.81E-04	3.21E+00	2.24E-01
288899	k__Bacteria;p__Verrucomicrobia;c__Verruco-5;o__WCHB1-41;f__RFP12;g__;s__	1.81E-04	3.10E-01	2.63E+00
New.CleanUp.ReferenceOTU129428	k__Bacteria;p__Firmicutes;c__Clostridia;o__Clostridiales;f__Ruminococcaceae;g__;s__	1.83E-04	1.41E-01	8.51E-01
New.ReferenceOTU73	k__Bacteria;p__Firmicutes;c__Clostridia;o__Clostridiales;f__Lachnospiraceae;g__;s__	1.85E-04	2.96E-01	1.43E+00
296124	k__Bacteria;p__Bacteroidetes;c__Bacteroidia;o__Bacteroidales;f__;g__;s__	1.88E-04	1.23E+01	1.66E+01
New.ReferenceOTU308	k__Bacteria;p__Firmicutes;c__Clostridia;o__Clostridiales;f__Ruminococcaceae;g__Ruminococcus;s__	1.90E-04	7.18E-01	3.82E+00
319818	k__Bacteria;p__Firmicutes;c__Clostridia;o__Clostridiales;f__Ruminococcaceae;g__;s__	1.93E-04	8.69E+00	1.31E+00
343667	k__Bacteria;p__Firmicutes;c__Clostridia;o__Clostridiales;f__;g__;s__	1.95E-04	5.07E-01	2.04E+00
183620	k__Bacteria;p__Firmicutes;c__Clostridia;o__Clostridiales;f__Ruminococcaceae;g__;s__	1.95E-04	3.28E+00	6.52E+00
4449054	k__Bacteria;p__Bacteroidetes;c__Bacteroidia;o__Bacteroidales;f__Bacteroidaceae;g__Bacteroides;s__caccae	1.98E-04	4.82E+00	9.40E-01
684238	k__Bacteria;p__Firmicutes;c__Clostridia;o__Clostridiales;f__Ruminococcaceae;g__;s__	2.02E-04	2.68E-01	1.07E+00
1148019	k__Bacteria;p__Firmicutes;c__Bacilli;o__Bacillales;f__Planococcaceae	2.04E-04	4.21E+01	1.64E+01
314552	k__Bacteria;p__Bacteroidetes;c__Bacteroidia;o__Bacteroidales;f__;g__;s__	2.09E-04	2.42E+00	4.58E+00
177037	k__Bacteria;p__Firmicutes;c__Clostridia;o__Clostridiales;f__Lachnospiraceae;g__;s__	2.10E-04	3.25E+00	3.28E-01
348377	k__Bacteria;p__Firmicutes;c__Clostridia;o__Clostridiales;f__;g__;s__	2.11E-04	3.04E+00	1.58E+00
187233	k__Bacteria;p__Firmicutes;c__Bacilli;o__Lactobacillales;f__Lactobacillaceae;g__Lactobacillus;s__	2.14E-04	7.21E+00	3.22E+00
3550973	k__Bacteria;p__Firmicutes;c__Clostridia;o__Clostridiales;f__Ruminococcaceae;g__;s__	2.15E-04	3.07E+00	4.48E-02
309179	k__Bacteria;p__Firmicutes;c__Clostridia;o__Clostridiales;f__Clostridiaceae;g__;s__	2.15E-04	5.69E+00	9.13E+00

195252	k__Bacteria;p__Firmicutes;c__Clostridia;o__Clostridiales;f__Ruminococcaceae;g__;s__	2.19E-04	1.87E+01	4.30E+00
2298935	k__Bacteria;p__Firmicutes;c__Clostridia;o__Clostridiales;f__Lachnospiraceae;g__Roseburia;s__	2.20E-04	9.44E-01	3.58E-01
359872	k__Bacteria;p__Proteobacteria;c__Deltaproteobacteria;o__Desulfovibrionales;f__Desulfovibrionaceae;g__Bilophila;s__	2.25E-04	4.94E+00	7.01E-01
288224	k__Bacteria;p__Firmicutes;c__Clostridia;o__Clostridiales;f__Lachnospiraceae;g__;s__	2.28E-04	7.92E+00	1.70E+00
302321	k__Bacteria;p__Firmicutes;c__Clostridia;o__Clostridiales;f__Lachnospiraceae;g__;s__	2.29E-04	1.89E+00	0.00E+00
New.ReferenceOTU437	k__Bacteria;p__Firmicutes;c__Clostridia;o__Clostridiales;f__[Mogibacteriaceae];g__;s__	2.32E-04	4.37E-01	0.00E+00
759751	k__Bacteria;p__Firmicutes;c__Clostridia;o__Clostridiales;f__Lachnospiraceae;g__;s__	2.34E-04	9.58E-01	0.00E+00
New.CleanUp.ReferenceOTU114509	k__Bacteria;p__Firmicutes;c__Clostridia;o__Clostridiales;f__Ruminococcaceae;g__;s__	2.35E-04	5.49E-01	0.00E+00
New.CleanUp.ReferenceOTU9068	k__Bacteria;p__Firmicutes;c__Clostridia;o__Clostridiales;f__[Mogibacteriaceae];g__;s__	2.35E-04	7.32E-01	0.00E+00
4298529	k__Bacteria;p__Actinobacteria;c__Coriobacteriia;o__Coriobacteriales;f__Coriobacteriaceae;g__;s__	2.35E-04	1.27E-01	7.16E-01
4473788	k__Bacteria;p__Firmicutes;c__Clostridia;o__Clostridiales;f__Lachnospiraceae;g__;s__	2.35E-04	4.08E-01	2.09E+00
30872	k__Bacteria;p__Firmicutes;c__Clostridia;o__Clostridiales;f__Ruminococcaceae;g__;s__	2.35E-04	1.13E+00	0.00E+00
189820	k__Bacteria;p__Firmicutes;c__Clostridia;o__Clostridiales;f__Ruminococcaceae;g__Ruminococcus;s__	2.39E-04	1.51E+00	8.96E-02
580121	k__Bacteria;p__Firmicutes;c__Clostridia;o__Clostridiales;f__Ruminococcaceae;g__;s__	2.39E-04	5.49E-01	2.25E+00
771181	k__Bacteria;p__Firmicutes;c__Clostridia;o__Clostridiales;f__Ruminococcaceae;g__;s__	2.47E-04	9.86E-02	1.99E+00
New.CleanUp.ReferenceOTU110614	k__Bacteria;p__Firmicutes;c__Clostridia;o__Clostridiales;f__Christensenellaceae;g__;s__	2.50E-04	2.82E-02	4.63E-01
288839	k__Bacteria;p__Firmicutes;c__Clostridia;o__Clostridiales;f__Ruminococcaceae;g__;s__	2.52E-04	4.06E+00	8.61E+00
205613	k__Bacteria;p__Firmicutes;c__Clostridia;o__Clostridiales;f__Lachnospiraceae;g__Coprococcus;s__	2.54E-04	7.86E+00	3.27E+00
4362337	k__Bacteria;p__Firmicutes;c__Clostridia;o__Clostridiales;f__Lachnospiraceae;g__;s__	2.54E-04	1.51E+00	5.97E-02
300853	k__Bacteria;p__Bacteroidetes;c__Bacteroidia;o__Bacteroidales;f__[Paraprevotellaceae];g__CF231;s__	2.54E-04	0.00E+00	6.57E-01
New.ReferenceOTU617	k__Bacteria;p__Firmicutes;c__Clostridia;o__Clostridiales;f__Lachnospiraceae;g__;s__	2.54E-04	1.03E+00	3.28E-01
New.ReferenceOTU715	k__Bacteria;p__Bacteroidetes;c__Bacteroidia;o__Bacteroidales;f__[Paraprevotellaceae];g__YRC22;s__	2.54E-04	1.55E-01	2.34E+00
184249	k__Bacteria;p__Firmicutes;c__Clostridia;o__Clostridiales;f__;g__;s__	2.55E-04	9.15E-01	2.99E-02
301733	k__Bacteria;p__Firmicutes;c__Clostridia;o__Clostridiales;f__Ruminococcaceae;g__;s__	2.59E-04	7.04E-01	2.60E+00
New.CleanUp.ReferenceOTU18863	k__Bacteria;p__Firmicutes;c__Clostridia;o__Clostridiales;f__[Mogibacteriaceae];g__;s__	2.59E-04	4.23E-01	2.60E+00
235994	k__Bacteria;p__Bacteroidetes;c__Bacteroidia;o__Bacteroidales;f__;g__;s__	2.60E-04	1.55E-01	6.72E-01
584107	k__Bacteria;p__Firmicutes;c__Clostridia;o__Clostridiales;f__;g__;s__	2.62E-04	4.23E+00	1.07E+00
2943548	k__Bacteria;p__Firmicutes;c__Clostridia;o__Clostridiales;f__Ruminococcaceae;g__Ruminococcus;s__	2.63E-04	6.45E+00	1.54E+00
New.CleanUp.ReferenceOTU84961	k__Bacteria;p__Firmicutes;c__Clostridia;o__Clostridiales;f__Lachnospiraceae;g__;s__	2.64E-04	1.83E-01	1.19E+00
New.ReferenceOTU296	k__Archaea;p__Euryarchaeota;c__Methanomicrobia;o__Methanomicrobiales;f__Methanocorpusculaceae;g__Methanocorpusculum;s__	2.65E-04	5.85E+00	1.30E+01
299639	k__Bacteria;p__Firmicutes;c__Clostridia;o__Clostridiales;f__Lachnospiraceae;g__;s__	2.67E-04	1.10E+00	3.19E+00
New.CleanUp.ReferenceOTU84411	k__Bacteria;p__Firmicutes;c__Clostridia;o__Clostridiales;f__Lachnospiraceae;g__;s__	2.69E-04	2.82E-02	4.48E-01

New.ReferenceOTU443	k__Bacteria;p__Bacteroidetes;c__Bacteroidia;o__Bacteroidales;f__Bacteroidaceae;g__Bacteroides;s__	2.74E-04	8.87E-01	7.46E-02
51306	k__Bacteria;p__Firmicutes;c__Clostridia;o__Clostridiales;f__Ruminococcaceae;g__Faecalibacterium;s__prausnitzii	2.83E-04	1.37E+00	2.54E-01
288079	k__Bacteria;p__Firmicutes;c__Clostridia;o__Clostridiales;f__Ruminococcaceae;g__;s__	2.84E-04	2.93E+00	3.06E+00
4422039	k__Bacteria;p__Firmicutes;c__Clostridia;o__Clostridiales;f__Lachnospiraceae	3.08E-04	6.34E-01	4.48E-02
New.ReferenceOTU325	k__Bacteria;p__Actinobacteria;c__Coriobacteriia;o__Coriobacteriales;f__Coriobacteriaceae;g__;s__	3.19E-04	1.17E+00	3.40E+00
New.ReferenceOTU186	k__Bacteria;p__Firmicutes;c__Clostridia;o__Clostridiales;f__;g__;s__	3.20E-04	5.63E-01	3.07E+00
187050	k__Bacteria;p__Firmicutes;c__Clostridia;o__Clostridiales;f__Lachnospiraceae;g__;s__	3.22E-04	9.86E-01	1.19E-01
730939	k__Bacteria;p__Firmicutes;c__Clostridia;o__Clostridiales;f__;g__;s__	3.24E-04	4.73E+00	9.25E-01
4342104	k__Bacteria;p__Firmicutes;c__Clostridia;o__Clostridiales;f__Ruminococcaceae;g__Anaerotruncus;s__	3.25E-04	1.28E+00	2.69E-01
New.ReferenceOTU538	k__Bacteria;p__Bacteroidetes;c__Bacteroidia;o__Bacteroidales;f__;g__;s__	3.26E-04	1.11E+00	6.28E+00
3232988	k__Bacteria;p__Tenericutes;c__RF3;o__ML615J-28;f__;g__;s__	3.26E-04	7.89E-01	2.99E-02
New.ReferenceOTU669	k__Bacteria;p__Firmicutes;c__Clostridia;o__Clostridiales;f__Ruminococcaceae;g__;s__	3.34E-04	1.24E+00	2.84E-01
303313	k__Bacteria;p__Firmicutes;c__Clostridia;o__Clostridiales;f__Lachnospiraceae;g__;s__	3.36E-04	7.75E-01	8.96E-02
331489	k__Bacteria;p__Actinobacteria;c__Actinobacteria;o__Actinomycetales;f__Nocardiaceae;g__;s__	3.43E-04	2.82E-02	2.96E+00
New.ReferenceOTU128	k__Bacteria;p__Verrucomicrobia;c__Verruco-5;o__WCHB1-41;f__RFP12;g__;s__	3.44E-04	4.23E-02	2.57E+00
New.ReferenceOTU270	k__Bacteria;p__Firmicutes;c__Clostridia;o__Clostridiales;f__Ruminococcaceae;g__Ruminococcus;s__	3.44E-04	1.25E+00	4.37E+00
311692	k__Bacteria;p__Firmicutes;c__Clostridia;o__Clostridiales;f__Lachnospiraceae;g__;s__	3.44E-04	1.00E+00	1.19E-01
316781	k__Bacteria;p__Firmicutes;c__Clostridia;o__Clostridiales;f__Lachnospiraceae;g__;s__	3.44E-04	1.51E+00	1.67E+00
176617	k__Bacteria;p__Firmicutes;c__Clostridia;o__Clostridiales;f__Ruminococcaceae;g__;s__	3.44E-04	6.72E+00	5.67E-01
New.ReferenceOTU650	k__Archaea;p__Euryarchaeota;c__Methanobacteria;o__Methanobacteriales;f__Methanobacteriaceae;g__Methanobrevibacter;s__	3.45E-04	5.45E+00	1.23E+01
299607	k__Bacteria;p__Bacteroidetes;c__Bacteroidia;o__Bacteroidales;f__;g__;s__	3.45E-04	1.41E-01	1.19E+00
331449	k__Bacteria;p__Firmicutes;c__Clostridia;o__Clostridiales;f__;g__;s__	3.45E-04	1.69E-01	7.46E-01
301375	k__Bacteria;p__Firmicutes;c__Clostridia;o__Clostridiales;f__Ruminococcaceae;g__;s__	3.45E-04	2.23E+00	4.55E+00
New.ReferenceOTU300	k__Bacteria;p__Verrucomicrobia;c__Verruco-5;o__WCHB1-41;f__RFP12;g__;s__	3.45E-04	5.92E-01	3.19E+00
322281	k__Bacteria;p__Firmicutes;c__Clostridia;o__Clostridiales;f__Lachnospiraceae;g__;s__	3.46E-04	5.21E-01	2.51E+00
4378683	k__Bacteria;p__Firmicutes;c__Clostridia;o__Clostridiales;f__Lachnospiraceae;g__;s__	3.48E-04	1.72E+00	3.82E+00
4316060	k__Bacteria;p__Firmicutes;c__Clostridia;o__Clostridiales;f__Lachnospiraceae;g__Pseudobutyrivibrio;s__	3.57E-04	1.10E+00	2.04E+00
New.ReferenceOTU200	k__Bacteria;p__Firmicutes;c__Clostridia;o__Clostridiales;f__;g__;s__	3.64E-04	1.27E-01	1.04E+00
295350	k__Bacteria;p__Firmicutes;c__Clostridia;o__Clostridiales;f__;g__;s__	3.64E-04	7.04E-02	1.43E+00
320853	k__Bacteria;p__Firmicutes;c__Clostridia;o__Clostridiales;f__Clostridiaceae;g__;s__	3.65E-04	1.55E-01	8.36E-01
4323524	k__Bacteria;p__Firmicutes;c__Clostridia;o__Clostridiales;f__;g__;s__	3.66E-04	1.85E+00	2.54E-01
114821	k__Bacteria;p__Firmicutes;c__Clostridia;o__Clostridiales;f__Veillonellaceae;g__Veillonella;s__parvula	3.67E-04	2.11E+00	2.99E-01

New.ReferenceOTU882	k__Bacteria;p__Firmicutes;c__Clostridia;o__Clostridiales;f__Lachnospiraceae;g__;s__	3.92E-04	2.54E-01	1.19E+00
334682	k__Bacteria;p__Bacteroidetes;c__Bacteroidia;o__Bacteroidales;f__RF16;g__;s__	3.94E-04	1.13E-01	9.40E-01
191551	k__Bacteria;p__Firmicutes;c__Clostridia;o__Clostridiales;f__Lachnospiraceae;g__[Ruminococcus];s__	4.09E-04	1.03E+00	2.99E-02
180813	k__Bacteria;p__Firmicutes;c__Clostridia;o__Clostridiales;f__;g__;s__	4.09E-04	3.66E-01	0.00E+00
New.CleanUp.ReferenceOTU91080	k__Bacteria;p__Firmicutes;c__Clostridia;o__Clostridiales;f__;g__;s__	4.11E-04	4.23E-01	0.00E+00
New.CleanUp.ReferenceOTU155828	k__Bacteria;p__Firmicutes;c__Clostridia;o__Clostridiales;f__Ruminococcaceae;g__;s__	4.13E-04	2.48E+00	0.00E+00
4419621	k__Bacteria;p__Proteobacteria;c__Betaproteobacteria;o__Burkholderiales;f__Alcaligenaceae;g__Sutterella;s__	4.23E-04	1.85E+00	7.46E-02
New.CleanUp.ReferenceOTU95826	k__Bacteria;p__Firmicutes;c__Clostridia;o__Clostridiales;f__Ruminococcaceae;g__;s__	4.43E-04	5.63E-02	6.87E-01
3195723	k__Bacteria;p__Firmicutes;c__Clostridia;o__Clostridiales;f__Ruminococcaceae;g__Oscillospira;s__	4.48E-04	4.85E+00	1.93E+00
329798	k__Bacteria;p__Firmicutes;c__Clostridia;o__Clostridiales;f__Veillonellaceae;g__Phascolarctobacterium;s__	4.63E-04	8.31E-01	1.49E-02
296278	k__Bacteria;p__Firmicutes;c__Clostridia;o__Clostridiales;f__Lachnospiraceae;g__;s__	4.63E-04	9.86E-01	3.01E+00
New.CleanUp.ReferenceOTU75562	k__Bacteria;p__Spirochaetes;c__Spirochaetes;o__Spirochaetales;f__Spirochaetaceae;g__Treponema;s__	4.66E-04	5.63E-02	9.55E-01
2506486	k__Bacteria;p__Firmicutes;c__Clostridia;o__Clostridiales;f__Ruminococcaceae;g__;s__	4.72E-04	4.65E-01	1.49E-02
New.ReferenceOTU201	k__Bacteria;p__Firmicutes;c__Clostridia;o__Clostridiales;f__;g__;s__	4.72E-04	1.97E-01	6.27E-01
New.CleanUp.ReferenceOTU48136	k__Bacteria;p__Firmicutes;c__Clostridia;o__Clostridiales;f__Christensenellaceae;g__;s__	4.75E-04	0.00E+00	2.99E-01
New.CleanUp.ReferenceOTU124987	k__Bacteria;p__Firmicutes;c__Clostridia;o__Clostridiales;f__Lachnospiraceae	4.77E-04	0.00E+00	3.28E-01
New.CleanUp.ReferenceOTU85297	k__Bacteria;p__Spirochaetes;c__Spirochaetes;o__Spirochaetales;f__Spirochaetaceae;g__Treponema;s__	4.79E-04	0.00E+00	5.22E-01
297150	k__Bacteria;p__Firmicutes;c__Clostridia;o__Clostridiales;f__Lachnospiraceae;g__Dorea;s__	4.80E-04	3.27E+00	1.00E+00
New.ReferenceOTU647	k__Bacteria;p__Firmicutes;c__Clostridia;o__Clostridiales;f__;g__;s__	4.80E-04	0.00E+00	3.88E-01
New.CleanUp.ReferenceOTU163990	k__Bacteria;p__Bacteroidetes;c__Bacteroidia;o__Bacteroidales;f__;g__;s__	4.80E-04	0.00E+00	6.72E-01
297133	k__Bacteria;p__Firmicutes;c__Clostridia;o__Clostridiales;f__Ruminococcaceae;g__;s__	4.83E-04	2.54E-01	1.43E+00
New.CleanUp.ReferenceOTU7725	k__Bacteria;p__Spirochaetes;c__Spirochaetes;o__Spirochaetales;f__Spirochaetaceae;g__Treponema;s__	4.83E-04	2.82E-02	4.18E-01
4396298	k__Bacteria;p__Firmicutes;c__Clostridia;o__Clostridiales;f__Ruminococcaceae;g__;s__	4.84E-04	1.83E-01	1.37E+00
301410	k__Bacteria;p__Firmicutes;c__Clostridia;o__Clostridiales;f__Ruminococcaceae;g__;s__	4.94E-04	1.27E-01	1.09E+00
330831	k__Bacteria;p__Bacteroidetes;c__Bacteroidia;o__Bacteroidales;f__BS11;g__;s__	4.94E-04	2.82E-02	1.75E+00
312217	k__Bacteria;p__Firmicutes;c__Clostridia;o__Clostridiales;f__Lachnospiraceae;g__;s__	4.94E-04	1.13E-01	6.87E-01
292488	k__Bacteria;p__Bacteroidetes;c__Bacteroidia;o__Bacteroidales;f__;g__;s__	4.98E-04	3.00E+00	3.45E+00
New.CleanUp.ReferenceOTU31519	k__Bacteria;p__Firmicutes;c__Clostridia;o__Clostridiales;f__Ruminococcaceae;g__;s__	4.99E-04	1.83E-01	1.39E+00
342213	k__Bacteria;p__Firmicutes;c__Clostridia;o__Clostridiales;f__Ruminococcaceae;g__;s__	4.99E-04	6.48E-01	1.54E+00
New.ReferenceOTU476	k__Bacteria;p__Firmicutes;c__Clostridia;o__Clostridiales;f__Ruminococcaceae;g__Ruminococcus;s__	5.15E-04	8.03E-01	2.64E+00
New.ReferenceOTU380	k__Bacteria;p__Bacteroidetes;c__Bacteroidia;o__Bacteroidales;f__;g__;s__	5.16E-04	1.55E-01	1.10E+00
293637	k__Bacteria;p__Firmicutes;c__Clostridia;o__Clostridiales;f__Lachnospiraceae;g__;s__	5.26E-04	2.11E-01	1.21E+00

4405146	k__Bacteria;p__Firmicutes;c__Clostridia;o__Clostridiales;f__;g__;s__	5.26E-04	6.51E+01	1.16E+01
297178	k__Bacteria;p__Firmicutes;c__Clostridia;o__Clostridiales;f__Lachnospiraceae;g__;s__	5.43E-04	1.83E-01	1.43E+00
New.CleanUp.ReferenceOTU177068	k__Bacteria;p__Firmicutes;c__Clostridia;o__Clostridiales;f__Lachnospiraceae	5.68E-04	5.77E-01	1.04E-01
297301	k__Bacteria;p__Bacteroidetes;c__Bacteroidia;o__Bacteroidales;f__;g__;s__	5.71E-04	3.94E-01	1.03E+00
721150	k__Bacteria;p__Bacteroidetes;c__Bacteroidia;o__Bacteroidales;f__[Paraprevotellaceae];g__YRC22;s__	5.72E-04	2.54E-01	8.51E-01
4371341	k__Bacteria;p__Firmicutes;c__Clostridia;o__Clostridiales;f__Veillonellaceae;g__Phascolarctobacterium;s__	5.77E-04	1.83E+01	4.00E+01
New.CleanUp.ReferenceOTU46514	k__Bacteria;p__Firmicutes;c__Clostridia;o__Clostridiales;f__Ruminococcaceae;g__;s__	5.90E-04	9.58E-01	1.34E-01
306305	k__Bacteria;p__Verrucomicrobia;c__Verruco-5;o__WCHB1-41;f__RFP12;g__;s__	5.92E-04	8.45E-02	5.52E-01
New.CleanUp.ReferenceOTU98374	k__Bacteria;p__Bacteroidetes;c__Bacteroidia;o__Bacteroidales;f__;g__;s__	5.98E-04	4.23E-02	5.52E-01
294323	k__Bacteria;p__Spirochaetes;c__Spirochaetes;o__Spirochaetales;f__Spirochaetaceae;g__Treponema;s__	6.11E-04	6.48E-01	2.01E+00
340727	k__Bacteria;p__Bacteroidetes;c__Bacteroidia;o__Bacteroidales;f__;g__;s__	6.16E-04	2.83E+00	5.37E+00
331309	k__Bacteria;p__Firmicutes;c__Clostridia;o__Clostridiales;f__;g__;s__	6.17E-04	4.37E-01	2.10E+00
332266	k__Bacteria;p__Firmicutes;c__Clostridia;o__Clostridiales;f__;g__;s__	6.24E-04	2.72E+00	6.24E+00
4302904	k__Bacteria;p__Bacteroidetes;c__Bacteroidia;o__Bacteroidales;f__Bacteroidaceae;g__Bacteroides;s__	6.30E-04	4.85E+00	2.24E-01
New.CleanUp.ReferenceOTU55952	k__Bacteria;p__Firmicutes;c__Clostridia;o__Clostridiales;f__Lachnospiraceae;g__Blautia;s__	6.35E-04	6.34E-01	8.96E-02
4372003	k__Bacteria;p__Bacteroidetes;c__Bacteroidia;o__Bacteroidales;f__Porphyromonadaceae;g__Parabacteroides;s__	6.43E-04	3.49E+01	1.01E+01
355630	k__Bacteria;p__Firmicutes;c__Clostridia;o__Clostridiales;f__Lachnospiraceae;g__;s__	6.48E-04	3.32E+00	4.48E-01
4445801	k__Bacteria;p__Firmicutes;c__Clostridia;o__Clostridiales;f__;g__;s__	6.59E-04	1.66E+00	3.33E+00
New.ReferenceOTU445	k__Bacteria;p__Bacteroidetes;c__Bacteroidia;o__Bacteroidales;f__;g__;s__	6.64E-04	1.90E+00	6.18E+00
New.CleanUp.ReferenceOTU55327	k__Bacteria;p__Firmicutes;c__Clostridia;o__Clostridiales;f__Ruminococcaceae;g__;s__	6.64E-04	7.04E-02	1.03E+00
New.ReferenceOTU896	k__Bacteria;p__Firmicutes;c__Clostridia;o__Clostridiales;f__Lachnospiraceae;g__;s__	6.69E-04	1.97E-01	5.37E-01
New.CleanUp.ReferenceOTU174505	k__Bacteria;p__Firmicutes;c__Clostridia;o__Clostridiales;f__Ruminococcaceae;g__;s__	6.77E-04	2.68E-01	8.51E-01
New.CleanUp.ReferenceOTU132857	k__Bacteria;p__Firmicutes;c__Clostridia;o__Clostridiales;f__Lachnospiraceae;g__;s__	6.81E-04	4.79E-01	7.46E-02
4413619	k__Bacteria;p__Firmicutes;c__Clostridia;o__Clostridiales;f__Lachnospiraceae	6.95E-04	1.13E+00	4.48E-02
New.CleanUp.ReferenceOTU128500	k__Bacteria;p__Firmicutes;c__Clostridia;o__Clostridiales;f__;g__;s__	6.95E-04	3.24E-01	1.16E+00
537216	k__Bacteria;p__Spirochaetes;c__Spirochaetes;o__Spirochaetales;f__Spirochaetaceae;g__Treponema;s__	7.19E-04	5.35E-01	1.66E+00
182245	k__Bacteria;p__Firmicutes;c__Clostridia;o__Clostridiales;f__Lachnospiraceae;g__[Ruminococcus];s__	7.23E-04	1.15E+00	0.00E+00
New.CleanUp.ReferenceOTU12256	k__Bacteria;p__Proteobacteria;c__Deltaproteobacteria;o__Desulfovibrionales;f__Desulfovibrionaceae;g__Desulfovibrio;s__	7.25E-04	3.38E-01	0.00E+00
New.ReferenceOTU1002	k__Bacteria;p__Firmicutes;c__Clostridia;o__Clostridiales;f__Ruminococcaceae;g__Ruminococcus;s__	7.29E-04	7.04E-01	0.00E+00
1129680	k__Bacteria;p__Firmicutes;c__Bacilli;o__Lactobacillales;f__Carnobacteriaceae;g__Carnobacterium;s__	7.32E-04	9.75E+00	2.04E+00
299992	k__Bacteria;p__Firmicutes;c__Clostridia;o__Clostridiales;f__Ruminococcaceae;g__;s__	7.32E-04	3.94E-01	1.28E+00
4458634	k__Bacteria;p__Firmicutes;c__Clostridia;o__Clostridiales;f__Lachnospiraceae;g__[Ruminococcus];s__	7.50E-04	1.28E+00	2.99E-02

349887	k__Bacteria;p__Firmicutes;c__Clostridia;o__Clostridiales;f__Ruminococcaceae;g__;s__	7.52E-04	9.86E-02	6.27E-01
New.CleanUp.ReferenceOTU44677	k__Bacteria;p__Firmicutes;c__Clostridia;o__Clostridiales;f__Lachnospiraceae	7.56E-04	1.31E+00	2.69E+00
300689	k__Bacteria;p__Firmicutes;c__Bacilli;o__Lactobacillales;f__Carnobacteriaceae	7.64E-04	1.80E+00	1.64E-01
244041	k__Bacteria;p__Firmicutes;c__Clostridia;o__Clostridiales;f__;g__;s__	7.69E-04	3.80E-01	1.43E+00
319786	k__Bacteria;p__Bacteroidetes;c__Bacteroidia;o__Bacteroidales;f__BS11;g__;s__	7.72E-04	4.23E-02	2.27E+00
New.ReferenceOTU914	k__Bacteria;p__Firmicutes;c__Clostridia;o__Clostridiales;f__Christensenellaceae;g__;s__	7.73E-04	7.32E-01	1.45E+00
295854	k__Bacteria;p__Firmicutes;c__Clostridia;o__Clostridiales;f__Lachnospiraceae;g__;s__	7.78E-04	6.34E-01	1.48E+00
4315785	k__Bacteria;p__Firmicutes;c__Clostridia;o__Clostridiales;f__Ruminococcaceae;g__;s__	7.97E-04	4.23E-02	5.67E-01
New.ReferenceOTU293	k__Bacteria;p__Firmicutes;c__Clostridia;o__Clostridiales;f__Clostridiaceae;g__Sarcina;s__	8.01E-04	5.63E-01	1.61E+00
190649	k__Bacteria;p__Firmicutes;c__Clostridia;o__Clostridiales;f__Ruminococcaceae;g__;s__	8.03E-04	1.93E+00	1.49E-01
131039	k__Bacteria;p__Firmicutes;c__Clostridia;o__Clostridiales;f__Ruminococcaceae;g__Oscillospira;s__	8.11E-04	3.35E+00	5.07E-01
350834	k__Bacteria;p__Firmicutes;c__Clostridia;o__Clostridiales;f__Christensenellaceae;g__;s__	8.11E-04	1.55E-01	5.52E-01
288026	k__Bacteria;p__Firmicutes;c__Clostridia;o__Clostridiales;f__;g__;s__	8.38E-04	5.07E-01	1.49E-02
194286	k__Bacteria;p__Bacteroidetes;c__Bacteroidia;o__Bacteroidales;f__Porphyromonadaceae;g__Parabacteroides;s__	8.38E-04	3.18E+00	1.19E-01
23625	k__Bacteria;p__Proteobacteria;c__Deltaproteobacteria;o__GMD14H09;f__;g__;s__	8.39E-04	1.65E+00	2.99E-02
175967	k__Bacteria;p__Firmicutes;c__Clostridia;o__Clostridiales;f__;g__;s__	8.58E-04	6.76E-01	2.99E-02
New.CleanUp.ReferenceOTU124455	k__Bacteria;p__Firmicutes;c__Bacilli;o__Lactobacillales;f__Lactobacillaceae;g__Lactobacillus;s__reuteri	8.62E-04	3.80E-01	1.49E-02
565033	k__Archaea;p__Euryarchaeota;c__Methanomicrobia;o__Methanomicrobiales;f__Methanocorpusculaceae;g__Methanocorpusculum;s__	8.65E-04	4.23E-01	2.07E+00
New.CleanUp.ReferenceOTU179654	k__Bacteria;p__Firmicutes;c__Clostridia;o__Clostridiales;f__;g__;s__	8.69E-04	4.08E-01	1.49E-02
3931537	k__Bacteria;p__Firmicutes;c__Clostridia;o__Clostridiales;f__Ruminococcaceae;g__;s__	8.71E-04	1.48E+00	3.73E-01
3409363	k__Bacteria;p__Firmicutes;c__Clostridia;o__Clostridiales;f__Lachnospiraceae;g__Dorea;s__	8.72E-04	4.93E-01	2.99E-02
4453304	k__Bacteria;p__Firmicutes;c__Clostridia;o__Clostridiales;f__Lachnospiraceae;g__;s__	8.72E-04	1.21E+00	7.61E-01
New.CleanUp.ReferenceOTU52247	k__Bacteria;p__Verrucomicrobia;c__Verruco-5;o__WCHB1-41;f__RFP12;g__;s__	8.76E-04	5.63E-02	2.06E+00
228140	k__Bacteria;p__Firmicutes;c__Clostridia;o__Clostridiales;f__Clostridiaceae;g__;s__	8.76E-04	2.80E+00	3.73E-01
1146771	k__Bacteria;p__Firmicutes;c__Clostridia;o__Clostridiales;f__Christensenellaceae;g__Christensenella;s__	8.78E-04	3.94E-01	1.49E-02
3407052	k__Bacteria;p__Bacteroidetes;c__Bacteroidia;o__Bacteroidales;f__Bacteroidaceae;g__Bacteroides;s__	8.79E-04	6.62E-01	3.70E+00
New.CleanUp.ReferenceOTU67939	k__Bacteria;p__Bacteroidetes;c__Bacteroidia;o__Bacteroidales;f__;g__;s__	8.85E-04	0.00E+00	2.12E+00
New.CleanUp.ReferenceOTU117040	k__Bacteria;p__Firmicutes;c__Clostridia;o__Clostridiales;f__Ruminococcaceae;g__Ruminococcus;s__	8.89E-04	0.00E+00	1.00E+00
4353913	k__Bacteria;p__Firmicutes;c__Clostridia;o__Clostridiales;f__;g__;s__	8.93E-04	8.31E-01	1.64E-01
New.ReferenceOTU452	k__Bacteria;p__Firmicutes;c__Clostridia;o__Clostridiales;f__Ruminococcaceae;g__;s__	9.02E-04	6.20E-01	5.97E-02
4452764	k__Bacteria;p__Firmicutes;c__Clostridia;o__Clostridiales;f__;g__;s__	9.10E-04	1.97E-01	8.81E-01
19080	k__Bacteria;p__Firmicutes;c__Clostridia;o__Clostridiales;f__Ruminococcaceae;g__;s__	9.17E-04	1.94E+00	4.78E-01

556163	k__Bacteria;p__Firmicutes;c__Clostridia;o__Clostridiales;f__Clostridiaceae;g__;s__	9.84E-04	1.41E-02	4.12E+00
543491	k__Bacteria;p__Actinobacteria;c__Actinobacteria;o__Actinomycetales;f__Micrococcaceae;g__;s__	1.00E-03	1.38E+00	2.99E-01
New.CleanUp.ReferenceOTU158944	k__Bacteria;p__Chloroflexi;c__Anaerolineae;o__Anaerolineales;f__Anaerolinaceae;g__SHD-231;s__	1.00E-03	1.41E-02	7.76E-01
341139	k__Bacteria;p__Firmicutes;c__Clostridia;o__Clostridiales;f__Lachnospiraceae;g__;s__	1.03E-03	2.28E+00	5.07E-01
297502	k__Bacteria;p__Firmicutes;c__Clostridia;o__Clostridiales;f__Lachnospiraceae;g__;s__	1.03E-03	1.08E+00	4.48E-02
303224	k__Bacteria;p__Firmicutes;c__Clostridia;o__Clostridiales;f__Lachnospiraceae;g__Blautia;s__	1.04E-03	7.54E+00	9.40E-01
New.ReferenceOTU448	k__Bacteria;p__Armatimonadetes;c__SJA-176;o__RB046;f__;g__;s__	1.06E-03	5.80E+00	1.27E+01
New.CleanUp.ReferenceOTU96977	k__Bacteria;p__Firmicutes;c__Clostridia;o__Clostridiales;f__Lachnospiraceae;g__;s__	1.09E-03	1.83E-01	8.66E-01
339530	k__Bacteria;p__Firmicutes;c__Clostridia;o__Clostridiales;f__;g__;s__	1.10E-03	2.96E-01	2.75E+00
338534	k__Bacteria;p__Bacteroidetes;c__Bacteroidia;o__Bacteroidales;f__;g__;s__	1.11E-03	1.69E-01	9.70E-01
310136	k__Bacteria;p__Firmicutes;c__Clostridia;o__Clostridiales;f__;g__;s__	1.11E-03	2.11E-01	1.04E+00
291054	k__Bacteria;p__Firmicutes;c__Clostridia;o__Clostridiales;f__Ruminococcaceae;g__;s__	1.11E-03	1.03E+00	3.73E+00
4339144	k__Bacteria;p__Bacteroidetes;c__Bacteroidia;o__Bacteroidales;f__[Odoribacteraceae];g__Butyricimonas;s__	1.12E-03	1.18E+00	3.73E-01
525378	k__Bacteria;p__Firmicutes;c__Clostridia;o__Clostridiales;f__Lachnospiraceae;g__Blautia;s__	1.12E-03	2.96E-01	7.76E-01
New.CleanUp.ReferenceOTU177938	k__Bacteria;p__Bacteroidetes;c__Bacteroidia;o__Bacteroidales;f__Prevotellaceae;g__Prevotella;s__	1.13E-03	1.13E-01	1.09E+00
4355718	k__Bacteria;p__Actinobacteria;c__Actinobacteria;o__Actinomycetales;f__Actinomycetaceae;g__Actinomyces;s__	1.13E-03	2.63E+00	7.55E+00
2510541	k__Bacteria;p__Firmicutes;c__Clostridia;o__Clostridiales;f__Lachnospiraceae;g__Coprococcus;s__	1.14E-03	3.94E-01	3.54E+00
325179	k__Bacteria;p__Bacteroidetes;c__Bacteroidia;o__Bacteroidales;f__;g__;s__	1.15E-03	3.80E-01	2.49E+00
757005	k__Bacteria;p__Firmicutes;c__Bacilli;o__Bacillales;f__Planococcaceae;g__Rummeliibacillus;s__	1.16E-03	3.68E+01	8.02E+01
New.CleanUp.ReferenceOTU6662	k__Bacteria;p__Tenericutes;c__Mollicutes;o__RF39;f__;g__;s__	1.19E-03	2.82E-02	7.16E-01
192385	k__Bacteria;p__Firmicutes;c__Clostridia;o__Clostridiales;f__;g__;s__	1.20E-03	6.06E-01	1.19E-01
295109	k__Bacteria;p__Verrucomicrobia;c__Verruco-5;o__WCHB1-41;f__RFP12;g__;s__	1.22E-03	2.82E-02	1.39E+00
4428037	k__Bacteria;p__Verrucomicrobia;c__Verruco-5;o__WCHB1-41;f__RFP12;g__;s__	1.22E-03	5.63E-01	1.60E+00
2875735	k__Bacteria;p__Bacteroidetes;c__Bacteroidia;o__Bacteroidales;f__Bacteroidaceae;g__Bacteroides;s__	1.24E-03	2.17E+00	8.06E-01
1857626	k__Bacteria;p__Firmicutes;c__Clostridia;o__Clostridiales;f__;g__;s__	1.26E-03	1.68E+00	0.00E+00
New.CleanUp.ReferenceOTU171806	k__Bacteria;p__Spirochaetes;c__Spirochaetes;o__Spirochaetales;f__Spirochaetaceae;g__Treponema;s__	1.27E-03	5.63E-02	4.03E-01
New.CleanUp.ReferenceOTU26099	k__Bacteria;p__Firmicutes;c__Clostridia;o__Clostridiales;f__Lachnospiraceae;g__Dorea;s__	1.27E-03	3.52E-01	0.00E+00
New.CleanUp.ReferenceOTU106721	k__Bacteria;p__Firmicutes;c__Clostridia;o__Clostridiales;f__Ruminococcaceae;g__Ruminococcus;s__	1.27E-03	1.01E+00	0.00E+00
New.CleanUp.ReferenceOTU56565	k__Bacteria;p__Firmicutes;c__Clostridia;o__Clostridiales;f__;g__;s__	1.27E-03	5.49E-01	0.00E+00
4301298	k__Bacteria;p__Bacteroidetes;c__Bacteroidia;o__Bacteroidales;f__Bacteroidaceae;g__Bacteroides;s__	1.28E-03	5.63E-01	0.00E+00
New.ReferenceOTU475	k__Bacteria;p__Firmicutes;c__Clostridia;o__Clostridiales;f__;g__;s__	1.28E-03	4.65E-01	1.51E+00
25562	k__Bacteria;p__Proteobacteria;c__Betaproteobacteria;o__Tremblayales;f__;g__;s__	1.30E-03	1.12E+01	1.29E+01

22371	k__Bacteria;p__Firmicutes;c__Clostridia;o__Clostridiales;f__Lachnospiraceae;g__;s__	1.32E-03	2.06E+00	7.46E-02
4338990	k__Bacteria;p__Firmicutes;c__Clostridia;o__Clostridiales;f__Ruminococcaceae;g__;s__	1.32E-03	1.97E-01	1.37E+00
212807	k__Bacteria;p__Firmicutes;c__Clostridia;o__Clostridiales;f__Ruminococcaceae;g__;s__	1.32E-03	9.30E-01	2.99E-02
292866	k__Bacteria;p__Firmicutes;c__Clostridia;o__Clostridiales;f__;g__;s__	1.32E-03	8.45E-02	4.18E-01
166911	k__Bacteria;p__Firmicutes;c__Bacilli;o__Lactobacillales;f__Lactobacillaceae;g__Lactobacillus;s__	1.33E-03	6.54E+00	3.07E+00
323189	k__Bacteria;p__Firmicutes;c__Clostridia;o__Clostridiales;f__;g__;s__	1.35E-03	4.23E-02	6.57E-01
297256	k__Bacteria;p__Firmicutes;c__Clostridia;o__Clostridiales;f__Ruminococcaceae;g__;s__	1.38E-03	1.14E+00	3.27E+00
326014	k__Bacteria;p__Firmicutes;c__Clostridia;o__Clostridiales;f__;g__;s__	1.39E-03	4.23E-02	5.07E-01
New.ReferenceOTU65	k__Bacteria;p__Firmicutes;c__Clostridia;o__Clostridiales;f__Ruminococcaceae;g__;s__	1.40E-03	1.41E-01	1.58E+00
2250985	k__Bacteria;p__Firmicutes;c__Clostridia;o__Clostridiales;f__Lachnospiraceae;g__Roseburia;s__	1.41E-03	1.46E+00	8.36E-01
292914	k__Bacteria;p__Bacteroidetes;c__Bacteroidia;o__Bacteroidales;f__;g__;s__	1.42E-03	1.27E-01	1.01E+00
New.CleanUp.ReferenceOTU30868	k__Bacteria;p__Firmicutes;c__Clostridia;o__Clostridiales;f__[Mogibacteriaceae];g__;s__	1.46E-03	7.04E-02	5.37E-01
295361	k__Bacteria;p__Firmicutes;c__Clostridia;o__Clostridiales;f__;g__;s__	1.48E-03	4.23E-01	1.49E-02
New.CleanUp.ReferenceOTU96981	k__Bacteria;p__Firmicutes;c__Clostridia;o__Clostridiales;f__Lachnospiraceae	1.48E-03	5.07E-01	1.49E-02
4447072	k__Bacteria;p__Bacteroidetes;c__Bacteroidia;o__Bacteroidales;f__Bacteroidaceae;g__Bacteroides;s__	1.50E-03	1.54E+00	4.93E-01
238830	k__Bacteria;p__Actinobacteria;c__Actinobacteria;o__Actinomycetales;f__Actinomycetaceae;g__Arcanobacterium;s__	1.50E-03	5.21E-01	3.36E+00
4307974	k__Bacteria;p__Firmicutes;c__Clostridia;o__Clostridiales;f__[Mogibacteriaceae];g__;s__	1.51E-03	5.49E-01	1.49E-02
New.CleanUp.ReferenceOTU138642	k__Bacteria;p__Bacteroidetes;c__Bacteroidia;o__Bacteroidales;f__;g__;s__	1.51E-03	9.86E-02	4.93E-01
293471	k__Bacteria;p__Firmicutes;c__Clostridia;o__Clostridiales;f__Ruminococcaceae;g__;s__	1.51E-03	5.89E+00	1.16E+01
4428676	k__Bacteria;p__Firmicutes;c__Clostridia;o__Clostridiales;f__Lachnospiraceae;g__Coprococcus;s__	1.52E-03	8.31E-01	1.49E-02
839964	k__Bacteria;p__Firmicutes;c__Clostridia;o__Clostridiales;f__Ruminococcaceae;g__Oscillospira;s__	1.52E-03	7.32E-01	1.49E-02
New.ReferenceOTU360	k__Bacteria;p__Bacteroidetes;c__Bacteroidia;o__Bacteroidales;f__;g__;s__	1.52E-03	8.73E-01	2.45E+00
4089774	k__Bacteria;p__Firmicutes;c__Clostridia;o__Clostridiales;f__Lachnospiraceae;g__Blautia;s__producta	1.53E-03	4.37E-01	1.49E-02
4433417	k__Bacteria;p__Firmicutes;c__Clostridia;o__Clostridiales;f__Lachnospiraceae	1.55E-03	3.38E-01	1.49E-02
528266	k__Bacteria;p__Firmicutes;c__Clostridia;o__Clostridiales;f__Lachnospiraceae;g__;s__	1.58E-03	4.23E-01	2.99E-02
4316193	k__Bacteria;p__Actinobacteria;c__Actinobacteria;o__Actinomycetales;f__Micrococcaceae;g__Arthrobacter;s__	1.60E-03	1.64E+01	5.57E+00
New.ReferenceOTU314	k__Bacteria;p__Firmicutes;c__Clostridia;o__Clostridiales;f__Ruminococcaceae;g__;s__	1.61E-03	3.23E+00	3.34E+00
174415	k__Bacteria;p__Firmicutes;c__Clostridia;o__Clostridiales;f__Lachnospiraceae;g__Dorea;s__	1.62E-03	1.39E+00	5.97E-02
New.ReferenceOTU757	k__Bacteria;p__Firmicutes;c__Clostridia;o__Clostridiales;f__Ruminococcaceae;g__;s__	1.62E-03	3.66E-01	8.66E-01
New.CleanUp.ReferenceOTU155616	k__Bacteria;p__Firmicutes;c__Clostridia;o__Clostridiales;f__;g__;s__	1.63E-03	0.00E+00	3.58E-01
New.ReferenceOTU637	k__Bacteria;p__Firmicutes;c__Clostridia;o__Clostridiales;f__;g__;s__	1.63E-03	6.34E-01	1.24E+00
300152	k__Bacteria;p__Firmicutes;c__Clostridia;o__Clostridiales;f__;g__;s__	1.65E-03	2.82E-02	4.78E-01

195081	k__Bacteria;p__Firmicutes;c__Clostridia;o__Clostridiales;f__Lachnospiraceae;g__Dorea;s__	1.68E-03	5.92E-01	7.46E-02
292216	k__Bacteria;p__Firmicutes;c__Clostridia;o__Clostridiales;f__Lachnospiraceae;g__Dorea;s__	1.70E-03	8.59E-01	1.49E-01
192437	k__Bacteria;p__Firmicutes;c__Clostridia;o__Clostridiales;f__;g__;s__	1.71E-03	2.96E-01	7.61E-01
New.ReferenceOTU537	k__Bacteria;p__Firmicutes;c__Clostridia;o__Clostridiales;f__Lachnospiraceae;g__Dorea;s__	1.71E-03	5.49E-01	5.97E-02
311412	k__Bacteria;p__Firmicutes;c__Clostridia;o__Clostridiales;f__;g__;s__	1.72E-03	1.13E+00	1.85E+00
New.ReferenceOTU77	k__Bacteria;p__Firmicutes;c__Clostridia;o__Clostridiales;f__Lachnospiraceae;g__;s__	1.72E-03	7.61E-01	8.96E-02
292529	k__Bacteria;p__Bacteroidetes;c__Bacteroidia;o__Bacteroidales;f__[Paraprevotellaceae];g__YRC22;s__	1.73E-03	2.82E-02	6.12E-01
584020	k__Bacteria;p__Firmicutes;c__Clostridia;o__Clostridiales;f__;g__;s__	1.73E-03	1.45E+00	2.99E-02
290864	k__Bacteria;p__Firmicutes;c__Erysipelotrichi;o__Erysipelotrichales;f__Erysipelotrichaceae;g__p-75-a5;s__	1.75E-03	9.86E-02	8.06E-01
356624	k__Bacteria;p__Firmicutes;c__Clostridia;o__Clostridiales;f__[Mogibacteriaceae];g__;s__	1.76E-03	8.03E-01	1.81E+00
999814	k__Bacteria;p__Firmicutes;c__Bacilli;o__Bacillales;f__Staphylococcaceae;g__Jeotgalicoccus;s__	1.77E-03	2.38E+00	4.48E-02
3265161	k__Bacteria;p__Firmicutes;c__Clostridia;o__Clostridiales;f__Lachnospiraceae;g__Roseburia;s__	1.77E-03	1.01E+00	3.43E-01
644244	k__Bacteria;p__Firmicutes;c__Clostridia;o__Clostridiales;f__Christensenellaceae;g__;s__	1.79E-03	4.13E+00	1.19E-01
290496	k__Bacteria;p__Verrucomicrobia;c__Verruco-5;o__WCHB1-41;f__RFP12;g__;s__	1.84E-03	1.41E-02	3.28E-01
268361	k__Bacteria;p__Proteobacteria;c__Gammaproteobacteria;o__Pseudomonadales;f__Moraxellaceae;g__;s__	1.86E-03	1.94E+00	7.46E-02
296717	k__Bacteria;p__Firmicutes;c__Clostridia;o__Clostridiales;f__;g__;s__	1.89E-03	5.85E+00	2.23E+01
167463	k__Bacteria;p__Firmicutes;c__Clostridia;o__Clostridiales;f__;g__;s__	1.92E-03	8.45E-02	5.22E-01
New.ReferenceOTU309	k__Bacteria;p__Firmicutes;c__Clostridia;o__Clostridiales;f__;g__;s__	1.93E-03	2.68E-01	1.34E+00
298804	k__Bacteria;p__Firmicutes;c__Clostridia;o__Clostridiales;f__Ruminococcaceae;g__;s__	1.95E-03	1.05E+02	1.03E+02
New.CleanUp.ReferenceOTU30377	k__Bacteria;p__Firmicutes;c__Clostridia;o__Clostridiales;f__[Mogibacteriaceae];g__Anaerovorax;s__	1.95E-03	1.55E-01	9.40E-01
New.CleanUp.ReferenceOTU109609	k__Bacteria;p__Synergistetes;c__Synergistia;o__Synergistales;f__Synergistaceae;g__;s__	1.97E-03	4.23E-02	1.67E+00
300620	k__Bacteria;p__Firmicutes;c__Clostridia;o__Clostridiales;f__Ruminococcaceae;g__;s__	1.97E-03	2.82E-02	7.16E-01
509596	k__Bacteria;p__Firmicutes;c__Clostridia;o__Clostridiales;f__;g__;s__	2.02E-03	2.54E-01	1.66E+00
306146	k__Bacteria;p__Firmicutes;c__Clostridia;o__Clostridiales;f__Clostridiaceae;g__;s__	2.03E-03	7.61E-01	2.84E+00
1945397	k__Bacteria;p__Firmicutes;c__Clostridia;o__Clostridiales;f__;g__;s__	2.03E-03	5.77E-01	5.97E-02
112158	k__Bacteria;p__Actinobacteria;c__Actinobacteria;o__Actinomycetales;f__Corynebacteriaceae;g__Corynebacterium;s__	2.04E-03	1.00E+00	7.46E-02
New.CleanUp.ReferenceOTU13023	k__Bacteria;p__Bacteroidetes;c__Bacteroidia;o__Bacteroidales;f__[Paraprevotellaceae];g__CF231;s__	2.06E-03	5.63E-02	9.55E-01
532922	k__Bacteria;p__Firmicutes;c__Clostridia;o__Clostridiales;f__Ruminococcaceae;g__Oscillospira;s__	2.09E-03	9.58E-01	7.46E-02
New.ReferenceOTU299	k__Bacteria;p__Fibrobacteres;c__Fibrobacteria;o__Fibrobacterales;f__Fibrobacteraceae;g__Fibrobacter;s__succinogenes	2.12E-03	1.41E-01	9.55E-01
291927	k__Bacteria;p__Bacteroidetes;c__Bacteroidia;o__Bacteroidales;f__Prevotellaceae;g__Prevotella;s__	2.16E-03	5.63E-02	5.07E-01
187404	k__Bacteria;p__Firmicutes;c__Clostridia;o__Clostridiales;f__Ruminococcaceae;g__;s__	2.17E-03	1.37E+00	8.96E-02
323106	k__Bacteria;p__Firmicutes;c__Clostridia;o__Clostridiales;f__Lachnospiraceae;g__;s__	2.18E-03	4.23E-01	0.00E+00

314710	k__Bacteria;p__Firmicutes;c__Clostridia;o__Clostridiales;f__;g__;s__	2.18E-03	2.11E-01	6.87E-01
290542	k__Bacteria;p__Firmicutes;c__Clostridia;o__Clostridiales;f__;g__;s__	2.19E-03	3.93E+00	9.90E+00
New.CleanUp.ReferenceOTU31803	Unassigned	2.19E-03	1.13E-01	1.66E+00
2829179	k__Bacteria;p__Firmicutes;c__Clostridia;o__Clostridiales;f__Veillonellaceae;g__Acidaminococcus;s__	2.19E-03	5.21E-01	0.00E+00
New.CleanUp.ReferenceOTU78219	k__Bacteria;p__Firmicutes;c__Clostridia;o__Clostridiales;f__Lachnospiraceae;g__Blautia;s__producta	2.20E-03	3.24E-01	0.00E+00
183686	k__Bacteria;p__Firmicutes;c__Clostridia;o__Clostridiales;f__Ruminococcaceae;g__;s__	2.20E-03	8.87E-01	0.00E+00
New.CleanUp.ReferenceOTU143072	k__Bacteria;p__Firmicutes;c__Clostridia;o__Clostridiales;f__[Mogibacteriaceae];g__;s__	2.21E-03	7.75E-01	0.00E+00
New.CleanUp.ReferenceOTU78716	k__Bacteria;p__Bacteroidetes;c__Bacteroidia;o__Bacteroidales;f__Bacteroidaceae;g__Bacteroides;s__fragilis	2.22E-03	7.32E-01	4.48E-02
New.CleanUp.ReferenceOTU155521	k__Bacteria;p__Bacteroidetes;c__Bacteroidia;o__Bacteroidales;f__;g__;s__	2.23E-03	4.23E-02	4.48E-01
177663	k__Bacteria;p__Firmicutes;c__Clostridia;o__Clostridiales;f__Ruminococcaceae;g__;s__	2.24E-03	6.99E+00	4.93E-01
New.CleanUp.ReferenceOTU180617	k__Bacteria;p__Firmicutes;c__Clostridia;o__Clostridiales;f__Ruminococcaceae;g__;s__	2.28E-03	3.66E-01	9.25E-01
New.CleanUp.ReferenceOTU100096	k__Bacteria;p__Firmicutes;c__Clostridia;o__Clostridiales;f__Ruminococcaceae;g__Ruminococcus;s__	2.31E-03	1.01E+00	2.39E+00
326721	k__Bacteria;p__Firmicutes;c__Bacilli;o__Lactobacillales;f__Lactobacillaceae;g__Lactobacillus;s__	2.33E-03	4.24E+00	1.18E+00
195937	k__Bacteria;p__Firmicutes;c__Clostridia;o__Clostridiales;f__Lachnospiraceae;g__Blautia;s__	2.34E-03	4.10E+00	8.81E-01
288004	k__Bacteria;p__Actinobacteria;c__Coriobacteriia;o__Coriobacteriales;f__Coriobacteriaceae;g__Collinsella;s__stercoris	2.38E-03	9.86E-02	1.21E+00
New.CleanUp.ReferenceOTU40450	Unassigned	2.44E-03	3.94E-01	1.42E+00
296528	k__Bacteria;p__Spirochaetes;c__Spirochaetes;o__Spirochaetales;f__Spirochaetaceae;g__Treponema;s__	2.45E-03	8.45E-02	4.18E-01
4352747	k__Bacteria;p__Bacteroidetes;c__Bacteroidia;o__Bacteroidales;f__Bacteroidaceae;g__Bacteroides;s__	2.48E-03	1.42E+00	2.99E-02
3472078	k__Bacteria;p__Bacteroidetes;c__Bacteroidia;o__Bacteroidales;f__Bacteroidaceae;g__Bacteroides;s__ovatus	2.49E-03	1.85E+00	3.28E-01
292255	k__Bacteria;p__Firmicutes;c__Clostridia;o__Clostridiales;f__;g__;s__	2.50E-03	3.24E-01	1.06E+00
4439360	k__Bacteria;p__Bacteroidetes;c__Bacteroidia;o__Bacteroidales;f__Bacteroidaceae;g__Bacteroides;s__	2.50E-03	6.24E+00	1.21E+00
New.CleanUp.ReferenceOTU71517	Unassigned	2.50E-03	5.63E-02	4.48E-01
293363	k__Bacteria;p__Fibrobacteres;c__Fibrobacteria;o__Fibrobacterales;f__Fibrobacteraceae;g__Fibrobacter;s__succinogenes	2.50E-03	1.41E-01	1.87E+00
New.ReferenceOTU957	k__Bacteria;p__Firmicutes;c__Clostridia;o__Clostridiales;f__Lachnospiraceae;g__Coprococcus;s__	2.51E-03	1.11E+00	2.30E+00
New.ReferenceOTU779	k__Bacteria;p__Firmicutes;c__Clostridia;o__Clostridiales;f__Clostridiaceae;g__;s__	2.53E-03	1.52E+00	3.52E+00
New.CleanUp.ReferenceOTU79069	k__Bacteria;p__Firmicutes;c__Clostridia;o__Clostridiales;f__Ruminococcaceae;g__;s__	2.53E-03	1.41E-01	8.96E-01
New.CleanUp.ReferenceOTU162067	k__Bacteria;p__Bacteroidetes;c__Bacteroidia;o__Bacteroidales;f__[Paraprevotellaceae];g__CF231;s__	2.55E-03	4.23E-02	4.78E-01
New.ReferenceOTU720	k__Bacteria;p__Actinobacteria;c__Coriobacteriia;o__Coriobacteriales;f__Coriobacteriaceae	2.56E-03	1.97E+00	4.45E+00
213337	k__Bacteria;p__Firmicutes;c__Clostridia;o__Clostridiales;f__[Mogibacteriaceae];g__;s__	2.56E-03	6.20E-01	1.22E+00
553080	k__Bacteria;p__Firmicutes;c__Clostridia;o__Clostridiales;f__Ruminococcaceae;g__;s__	2.56E-03	2.61E+00	1.04E-01
2386814	k__Bacteria;p__Firmicutes;c__Clostridia;o__Clostridiales;f__Lachnospiraceae;g__;s__	2.56E-03	2.39E-01	1.55E+00
47365	k__Bacteria;p__Firmicutes;c__Bacilli;o__Lactobacillales;f__Lactobacillaceae;g__Lactobacillus;s__	2.56E-03	2.43E+01	1.21E+01

New.ReferenceOTU412	k__Bacteria;p__Firmicutes;c__Clostridia;o__Clostridiales;f__Ruminococcaceae;g__;s__	2.58E-03	9.08E+00	1.47E+01
New.CleanUp.ReferenceOTU72309	k__Bacteria;p__Firmicutes;c__Clostridia;o__Clostridiales;f__Ruminococcaceae;g__;s__	2.58E-03	7.04E-01	1.49E-02
182188	k__Bacteria;p__Firmicutes;c__Clostridia;o__Clostridiales;f__;g__;s__	2.58E-03	1.04E+00	1.49E-02
New.CleanUp.ReferenceOTU92821	k__Bacteria;p__Firmicutes;c__Clostridia;o__Clostridiales;f__;g__;s__	2.61E-03	4.93E-01	1.49E-02
270722	k__Bacteria;p__Firmicutes;c__Clostridia;o__Clostridiales;f__Ruminococcaceae;g__Ruminococcus;s__flavefaciens	2.62E-03	1.10E+00	1.51E+00
New.CleanUp.ReferenceOTU13303	k__Bacteria;p__Firmicutes;c__Clostridia;o__Clostridiales;f__Lachnospiraceae;g__Coprococcus;s__	2.63E-03	5.35E-01	2.99E-02
347103	k__Bacteria;p__Bacteroidetes;c__Bacteroidia;o__Bacteroidales;f__;g__;s__	2.64E-03	2.96E-01	1.84E+00
192832	k__Bacteria;p__Firmicutes;c__Bacilli;o__Lactobacillales;f__Lactobacillaceae;g__Lactobacillus;s__	2.69E-03	2.96E-01	1.49E-02
320961	k__Bacteria;p__Firmicutes;c__Clostridia;o__Clostridiales;f__Ruminococcaceae;g__;s__	2.70E-03	2.31E+00	5.78E+00
630141	k__Bacteria;p__Firmicutes;c__Bacilli;o__Bacillales;f__Staphylococcaceae;g__Staphylococcus;s__	2.73E-03	2.32E+00	1.94E-01
295258	k__Bacteria;p__Firmicutes;c__Clostridia;o__Clostridiales;f__Ruminococcaceae;g__;s__	2.74E-03	2.56E+00	1.06E+00
New.CleanUp.ReferenceOTU24803	k__Bacteria;p__Firmicutes;c__Clostridia;o__Clostridiales;f__Lachnospiraceae;g__Blautia;s__producta	2.74E-03	3.80E-01	2.99E-02
331435	k__Bacteria;p__Firmicutes;c__Clostridia;o__Clostridiales;f__[Mogibacteriaceae];g__;s__	2.79E-03	3.39E+00	6.39E+00
2883968	k__Bacteria;p__Firmicutes;c__Clostridia;o__Clostridiales;f__;g__;s__	2.80E-03	6.54E+00	6.57E-01
4432463	k__Bacteria;p__Actinobacteria;c__Coriobacteriia;o__Coriobacteriales;f__Coriobacteriaceae;g__;s__	2.82E-03	7.32E-01	5.97E-02
323615	k__Bacteria;p__Firmicutes;c__Clostridia;o__Clostridiales;f__Clostridiaceae;g__;s__	2.90E-03	8.45E-02	2.34E+00
686211	k__Bacteria;p__Firmicutes;c__Clostridia;o__Clostridiales;f__Ruminococcaceae;g__Ruminococcus;s__flavefaciens	2.90E-03	1.01E+00	4.01E+00
New.CleanUp.ReferenceOTU105232	k__Bacteria;p__Firmicutes;c__Clostridia;o__Clostridiales;f__Christensenellaceae;g__;s__	2.92E-03	5.63E-02	1.15E+00
297402	k__Bacteria;p__Bacteroidetes;c__Bacteroidia;o__Bacteroidales;f__BS11;g__;s__	2.92E-03	4.04E+01	5.34E+01
316092	k__Bacteria;p__Bacteroidetes;c__Bacteroidia;o__Bacteroidales;f__Prevotellaceae;g__Prevotella;s__	2.94E-03	1.55E-01	1.09E+00
New.ReferenceOTU887	k__Bacteria;p__Firmicutes;c__Clostridia;o__Clostridiales;f__Lachnospiraceae;g__;s__	2.95E-03	0.00E+00	2.84E-01
4380450	k__Bacteria;p__Firmicutes;c__Clostridia;o__Clostridiales;f__Ruminococcaceae;g__Oscillospira;s__	2.95E-03	7.04E-01	2.99E-02
271582	k__Bacteria;p__SR1;c__;o__;f__;g__;s__	2.96E-03	0.00E+00	5.67E-01
302580	k__Bacteria;p__Verrucomicrobia;c__Verruco-5;o__WCHB1-41;f__RFP12;g__;s__	2.97E-03	4.23E-02	3.43E-01
4336939	k__Bacteria;p__Firmicutes;c__Clostridia;o__Clostridiales;f__Ruminococcaceae;g__;s__	2.98E-03	8.45E-02	5.67E-01
354924	k__Bacteria;p__Bacteroidetes;c__Bacteroidia;o__Bacteroidales;f__;g__;s__	3.00E-03	7.89E-01	2.04E+00
190658	k__Bacteria;p__Firmicutes;c__Clostridia;o__Clostridiales;f__Ruminococcaceae;g__Oscillospira;s__	3.00E-03	6.48E-01	4.33E-01
New.ReferenceOTU820	k__Bacteria;p__Proteobacteria;c__Deltaproteobacteria;o__Desulfovibrionales;f__Desulfovibrionaceae;g__Desulfovibrio;s__	3.01E-03	3.80E-01	2.42E+00
303247	k__Bacteria;p__Firmicutes;c__Clostridia;o__Clostridiales;f__Ruminococcaceae;g__;s__	3.01E-03	2.82E-02	7.01E-01
322719	k__Bacteria;p__Firmicutes;c__Clostridia;o__Clostridiales;f__Ruminococcaceae;g__;s__	3.01E-03	5.63E-02	4.78E-01
258447	k__Bacteria;p__Firmicutes;c__Clostridia;o__Clostridiales;f__;g__;s__	3.02E-03	1.01E+00	3.48E+00
292380	k__Bacteria;p__Verrucomicrobia;c__Verruco-5;o__WCHB1-41;f__RFP12;g__;s__	3.03E-03	8.59E-01	3.37E+00

287755	k__Bacteria;p__Firmicutes;c__Clostridia;o__Clostridiales;f__Lachnospiraceae;g__;s__	3.03E-03	9.86E-02	7.91E-01
331478	k__Bacteria;p__Firmicutes;c__Clostridia;o__Clostridiales;f__Ruminococcaceae;g__;s__	3.05E-03	8.45E-02	6.72E-01
98939	k__Bacteria;p__Firmicutes;c__Clostridia;o__Clostridiales;f__Clostridiaceae;g__Clostridium;s__	3.07E-03	2.11E-01	1.21E+00
785522	k__Bacteria;p__Firmicutes;c__Bacilli;o__Bacillales;f__Planococcaceae;g__Sporosarcina;s__	3.11E-03	2.89E+00	3.73E-01
571165	k__Bacteria;p__Firmicutes;c__Clostridia;o__Clostridiales;f__Ruminococcaceae;g__;s__	3.14E-03	3.00E+00	4.54E+00
317779	k__Bacteria;p__Bacteroidetes;c__Bacteroidia;o__Bacteroidales;f__[Paraprevotellaceae];g__CF231;s__	3.15E-03	2.00E+00	4.03E+00
New.ReferenceOTU340	k__Bacteria;p__Firmicutes;c__Clostridia;o__Clostridiales;f__;g__;s__	3.15E-03	1.13E-01	5.97E-01
New.ReferenceOTU938	k__Bacteria;p__Firmicutes;c__Clostridia;o__Clostridiales;f__Christensenellaceae;g__;s__	3.18E-03	1.41E-02	6.57E-01
204705	k__Bacteria;p__Firmicutes;c__Clostridia;o__Clostridiales;f__Ruminococcaceae;g__;s__	3.18E-03	1.41E-02	1.00E+00
191434	k__Bacteria;p__Firmicutes;c__Clostridia;o__Clostridiales;f__Lachnospiraceae;g__Dorea;s__	3.20E-03	4.51E-01	2.99E-02
4396297	k__Bacteria;p__Firmicutes;c__Clostridia;o__Clostridiales;f__Lachnospiraceae;g__;s__	3.20E-03	7.04E-01	4.48E-02
New.ReferenceOTU775	k__Bacteria;p__Firmicutes;c__Clostridia;o__Clostridiales;f__;g__;s__	3.20E-03	1.13E-01	6.57E-01
749798	k__Bacteria;p__Firmicutes;c__Clostridia;o__Clostridiales;f__Lachnospiraceae;g__;s__	3.20E-03	4.08E-01	1.21E+00
New.ReferenceOTU116	k__Bacteria;p__Firmicutes;c__Clostridia;o__Clostridiales;f__Ruminococcaceae;g__;s__	3.22E-03	9.58E-01	2.07E+00
306020	k__Bacteria;p__Firmicutes;c__Clostridia;o__Clostridiales;f__Ruminococcaceae;g__;s__	3.23E-03	1.27E-01	5.97E-01
294845	k__Bacteria;p__Verrucomicrobia;c__Verruco-5;o__WCHB1-41;f__RFP12;g__;s__	3.26E-03	2.39E-01	1.19E+00
New.CleanUp.ReferenceOTU32628	k__Bacteria;p__Firmicutes;c__Bacilli;o__Lactobacillales;f__Lactobacillaceae;g__Lactobacillus;s__	3.30E-03	8.45E-01	1.34E-01
289958	k__Bacteria;p__Firmicutes;c__Clostridia;o__Clostridiales;f__Ruminococcaceae;g__;s__	3.32E-03	2.85E+00	5.40E+00
294642	k__Bacteria;p__Bacteroidetes;c__Bacteroidia;o__Bacteroidales;f__BS11;g__;s__	3.32E-03	1.41E-02	5.37E-01
New.CleanUp.ReferenceOTU57539	k__Bacteria;p__Firmicutes;c__Clostridia;o__Clostridiales;f__Ruminococcaceae;g__;s__	3.32E-03	1.41E-02	3.43E-01
292175	k__Bacteria;p__Firmicutes;c__Bacilli;o__Lactobacillales;f__Lactobacillaceae;g__Lactobacillus;s__	3.34E-03	2.25E+00	6.57E-01
New.CleanUp.ReferenceOTU149931	k__Bacteria;p__Bacteroidetes;c__Bacteroidia;o__Bacteroidales;f__[Paraprevotellaceae];g__CF231;s__	3.38E-03	7.04E-02	6.57E-01
289752	k__Bacteria;p__Firmicutes;c__Clostridia;o__Clostridiales;f__Veillonellaceae;g__Phascolarctobacterium;s__	3.39E-03	2.21E+00	6.19E+00
162820	k__Bacteria;p__Firmicutes;c__Clostridia;o__Clostridiales;f__Lachnospiraceae;g__;s__	3.41E-03	2.11E-01	1.12E+00
New.ReferenceOTU498	k__Bacteria;p__Firmicutes;c__Clostridia;o__Clostridiales;f__Ruminococcaceae;g__;s__	3.45E-03	4.37E-01	7.76E-01
180659	k__Bacteria;p__Firmicutes;c__Clostridia;o__Clostridiales;f__Ruminococcaceae;g__Oscillospira;s__	3.48E-03	1.42E+00	5.52E-01
357046	k__Bacteria;p__Bacteroidetes;c__Bacteroidia;o__Bacteroidales;f__Rikenellaceae;g__;s__	3.48E-03	4.85E+00	6.27E-01
199286	k__Bacteria;p__Firmicutes;c__Clostridia;o__Clostridiales;f__Ruminococcaceae;g__;s__	3.49E-03	1.87E+00	2.39E-01
New.CleanUp.ReferenceOTU148523	k__Bacteria;p__Tenericutes;c__Mollicutes;o__RF39;f__;g__;s__	3.51E-03	1.38E+00	2.24E-01
New.ReferenceOTU992	k__Bacteria;p__Firmicutes;c__Clostridia;o__Clostridiales;f__[Mogibacteriaceae];g__;s__	3.52E-03	1.27E-01	6.12E-01
4397402	k__Bacteria;p__Firmicutes;c__Bacilli;o__Lactobacillales;f__Lactobacillaceae;g__Lactobacillus;s__	3.54E-03	1.41E+01	6.22E+00
300374	k__Bacteria;p__Firmicutes;c__Clostridia;o__Clostridiales;f__Ruminococcaceae;g__Oscillospira;s__	3.58E-03	3.38E-01	5.97E-02

4296701	k__Bacteria;p__Firmicutes;c__Clostridia;o__Clostridiales;f__Ruminococcaceae;g__Oscillospira;s__	3.60E-03	1.17E+00	1.34E-01
178809	k__Bacteria;p__Firmicutes;c__Clostridia;o__Clostridiales;f__Lachnospiraceae	3.62E-03	1.11E+00	1.49E-01
New.CleanUp.ReferenceOTU142481	k__Bacteria;p__Firmicutes;c__Clostridia	3.66E-03	2.11E-01	0.00E+00
268506	k__Bacteria;p__Firmicutes;c__Clostridia;o__Clostridiales;f__Veillonellaceae;g__Succiniclasticum;s__	3.67E-03	1.10E+00	0.00E+00
177593	k__Bacteria;p__Firmicutes;c__Clostridia;o__Clostridiales;f__Ruminococcaceae;g__Ruminococcus;s__	3.67E-03	2.96E-01	0.00E+00
New.CleanUp.ReferenceOTU7122	k__Bacteria;p__Firmicutes;c__Clostridia;o__Clostridiales;f__Lachnospiraceae;g__;s__	3.68E-03	2.54E-01	0.00E+00
New.CleanUp.ReferenceOTU101731	k__Bacteria;p__Firmicutes;c__Clostridia;o__Clostridiales;f__;g__;s__	3.69E-03	2.82E-01	0.00E+00
New.CleanUp.ReferenceOTU107694	k__Bacteria;p__Firmicutes;c__Clostridia;o__Clostridiales;f__Lachnospiraceae	3.69E-03	3.66E-01	0.00E+00
4459184	k__Bacteria;p__Firmicutes;c__Clostridia;o__Clostridiales;f__;g__;s__	3.69E-03	7.18E-01	0.00E+00
New.CleanUp.ReferenceOTU108600	k__Bacteria;p__Firmicutes;c__Clostridia;o__Clostridiales;f__Lachnospiraceae;g__;s__	3.69E-03	4.37E-01	0.00E+00
182033	k__Bacteria;p__Firmicutes;c__Clostridia;o__Clostridiales;f__Ruminococcaceae;g__Oscillospira;s__	3.69E-03	2.96E-01	0.00E+00
New.ReferenceOTU668	k__Bacteria;p__Bacteroidetes;c__Bacteroidia;o__Bacteroidales;f__Bacteroidaceae;g__Bacteroides;s__	3.69E-03	3.38E-01	0.00E+00
New.CleanUp.ReferenceOTU101301	k__Bacteria;p__Firmicutes;c__Clostridia;o__Clostridiales;f__;g__;s__	3.69E-03	3.52E-01	0.00E+00
184984	k__Bacteria;p__Firmicutes;c__Clostridia;o__Clostridiales;f__Lachnospiraceae;g__;s__	3.69E-03	5.21E-01	0.00E+00
110562	k__Bacteria;p__Firmicutes;c__Clostridia;o__Clostridiales;f__Ruminococcaceae;g__Ruminococcus;s__	3.70E-03	1.21E+00	0.00E+00
204629	k__Bacteria;p__Firmicutes;c__Clostridia;o__Clostridiales;f__Ruminococcaceae;g__;s__	3.70E-03	5.63E-02	4.63E-01
334395	k__Bacteria;p__Firmicutes;c__Clostridia;o__Clostridiales;f__;g__;s__	3.74E-03	2.27E+00	3.58E-01
3474081	k__Bacteria;p__Bacteroidetes;c__Bacteroidia;o__Bacteroidales;f__Bacteroidaceae;g__Bacteroides;s__fragilis	3.74E-03	5.39E+00	1.81E+00
304469	k__Bacteria;p__Firmicutes;c__Clostridia;o__Clostridiales;f__;g__;s__	3.78E-03	1.99E+00	2.09E+00
302427	k__Bacteria;p__Firmicutes;c__Clostridia;o__Clostridiales;f__Ruminococcaceae;g__;s__	3.78E-03	7.04E-02	1.28E+00
244120	k__Bacteria;p__Firmicutes;c__Clostridia;o__Clostridiales;f__Ruminococcaceae;g__;s__	3.79E-03	9.86E-02	7.61E-01
1135946	k__Bacteria;p__Proteobacteria;c__Epsilonproteobacteria;o__Campylobacterales;f__Helicobacteraceae;g__;s__	3.81E-03	2.49E+00	6.39E+00
278650	k__Bacteria;p__Cyanobacteria;c__4C0d-2;o__YS2;f__;g__;s__	3.85E-03	5.63E-02	4.33E-01
New.ReferenceOTU109	k__Bacteria;p__Tenericutes;c__RF3;o__ML615J-28;f__;g__;s__	3.87E-03	4.76E+00	2.03E+00
300390	k__Bacteria;p__Firmicutes;c__Clostridia;o__Clostridiales;f__;g__;s__	3.92E-03	9.86E-01	3.01E+00
New.ReferenceOTU252	k__Bacteria;p__Actinobacteria;c__Coriobacteriia;o__Coriobacteriales;f__Coriobacteriaceae;g__;s__	3.97E-03	7.61E-01	3.46E+00
362958	k__Bacteria;p__Firmicutes;c__Clostridia;o__Clostridiales;f__Lachnospiraceae;g__Blautia;s__	4.07E-03	7.89E-01	1.34E-01
95256	k__Bacteria;p__Firmicutes;c__Clostridia;o__Clostridiales;f__Ruminococcaceae;g__Ruminococcus;s__flavefaciens	4.08E-03	4.23E-02	4.78E-01
New.ReferenceOTU440	k__Bacteria;p__Spirochaetes;c__Spirochaetes;o__Spirochaetales;f__Spirochaetaceae;g__Treponema;s__	4.13E-03	1.13E-01	5.37E-01
4446739	k__Bacteria;p__Firmicutes;c__Clostridia;o__Clostridiales;f__Ruminococcaceae;g__;s__	4.13E-03	4.23E-02	2.99E-01
301555	k__Bacteria;p__Actinobacteria;c__Coriobacteriia;o__Coriobacteriales;f__Coriobacteriaceae;g__;s__	4.14E-03	8.31E-01	5.97E+00
New.CleanUp.ReferenceOTU137341	k__Bacteria;p__Firmicutes;c__Clostridia;o__Clostridiales;f__Lachnospiraceae;g__Blautia;s__	4.14E-03	2.82E-02	2.69E-01

593868	k__Bacteria;p__Tenericutes;c__Mollicutes;o__RF39;f__;g__;s__	4.15E-03	1.46E+00	2.24E-01
New.CleanUp.ReferenceOTU96505	k__Bacteria;p__Bacteroidetes;c__Bacteroidia;o__Bacteroidales;f__[Paraprevotellaceae];g__YRC22;s__	4.17E-03	2.82E-01	5.97E-01
4122608	k__Bacteria;p__Firmicutes;c__Clostridia;o__Clostridiales;f__Ruminococcaceae;g__;s__	4.17E-03	8.54E+00	1.72E+00
298788	k__Bacteria;p__Firmicutes;c__Clostridia;o__Clostridiales;f__Lachnospiraceae;g__;s__	4.24E-03	1.56E+00	1.79E-01
327282	k__Bacteria;p__Firmicutes;c__Clostridia;o__Clostridiales;f__;g__;s__	4.24E-03	9.30E-01	1.04E-01
4398588	k__Bacteria;p__Firmicutes;c__Clostridia;o__Clostridiales;f__Ruminococcaceae;g__;s__	4.26E-03	4.93E-01	2.99E-02
3090117	k__Bacteria;p__Bacteroidetes;c__Bacteroidia;o__Bacteroidales;f__[Barnesiellaceae];g__;s__	4.27E-03	1.66E+00	1.49E-02
New.ReferenceOTU301	k__Bacteria;p__Bacteroidetes;c__Bacteroidia;o__Bacteroidales;f__;g__;s__	4.31E-03	7.04E-01	1.15E+00
151006	k__Bacteria;p__Firmicutes;c__Clostridia;o__Clostridiales;f__Lachnospiraceae;g__Anaerostipes;s__	4.32E-03	5.63E-01	1.49E-02
New.ReferenceOTU911	k__Bacteria;p__Bacteroidetes;c__Bacteroidia;o__Bacteroidales;f__[Paraprevotellaceae];g__;s__	4.33E-03	5.35E-01	1.57E+00
347115	k__Bacteria;p__Firmicutes;c__Clostridia;o__Clostridiales;f__Lachnospiraceae;g__Blautia;s__	4.36E-03	1.97E-01	6.12E-01
New.CleanUp.ReferenceOTU19255	k__Bacteria;p__Firmicutes;c__Clostridia;o__Clostridiales;f__Lachnospiraceae;g__Dorea;s__	4.40E-03	6.76E-01	1.19E-01
276783	k__Bacteria;p__Firmicutes;c__Clostridia;o__Clostridiales;f__Lachnospiraceae;g__;s__	4.40E-03	7.46E-01	1.09E+00
New.CleanUp.ReferenceOTU31194	k__Bacteria;p__Spirochaetes;c__Spirochaetes;o__Spirochaetales;f__Spirochaetaceae;g__Treponema;s__	4.41E-03	2.82E-01	9.25E-01
273657	k__Bacteria;p__Firmicutes;c__Clostridia;o__Clostridiales;f__Peptococcaceae;g__rc4-4;s__	4.43E-03	1.20E+00	2.39E-01
New.ReferenceOTU784	k__Bacteria;p__Proteobacteria;c__Alphaproteobacteria;o__RF32;f__;g__;s__	4.44E-03	3.73E+00	4.63E-01
301447	k__Bacteria;p__Actinobacteria;c__Coriobacteriia;o__Coriobacteriales;f__Coriobacteriaceae;g__Adlercreutzia;s__	4.45E-03	3.76E+00	6.48E+00
177758	k__Bacteria;p__Firmicutes;c__Clostridia;o__Clostridiales;f__Lachnospiraceae;g__Dorea;s__	4.45E-03	5.21E-01	1.49E-02
288349	k__Bacteria;p__Firmicutes;c__Clostridia;o__Clostridiales;f__Ruminococcaceae;g__;s__	4.52E-03	5.77E-01	2.06E+00
New.CleanUp.ReferenceOTU63608	k__Bacteria;p__Actinobacteria;c__Coriobacteriia;o__Coriobacteriales;f__Coriobacteriaceae;g__;s__	4.64E-03	2.54E-01	8.36E-01
New.CleanUp.ReferenceOTU161788	k__Bacteria;p__Bacteroidetes;c__Bacteroidia;o__Bacteroidales;f__;g__;s__	4.66E-03	2.82E-01	9.10E-01
New.ReferenceOTU869	k__Bacteria;p__Bacteroidetes;c__Bacteroidia;o__Bacteroidales;f__;g__;s__	4.70E-03	2.54E-01	5.37E-01
355877	k__Bacteria;p__Firmicutes;c__Clostridia;o__Clostridiales;f__Lachnospiraceae;g__;s__	4.70E-03	2.96E-01	1.16E+00
New.CleanUp.ReferenceOTU38465	k__Bacteria;p__Firmicutes;c__Clostridia;o__Clostridiales;f__Lachnospiraceae;g__Coprococcus;s__	4.74E-03	7.04E-02	5.67E-01
New.ReferenceOTU261	k__Bacteria;p__Firmicutes;c__Clostridia;o__Clostridiales;f__Lachnospiraceae;g__;s__	4.74E-03	7.04E-01	1.04E+00
77458	k__Bacteria;p__Firmicutes;c__Clostridia;o__Clostridiales;f__Lachnospiraceae	4.77E-03	6.34E-01	4.48E-02
New.ReferenceOTU776	k__Bacteria;p__Actinobacteria;c__Coriobacteriia;o__Coriobacteriales;f__Coriobacteriaceae;g__Adlercreutzia;s__	4.77E-03	1.82E+00	3.39E+00
4405104	k__Bacteria;p__Firmicutes;c__Clostridia;o__Clostridiales;f__Lachnospiraceae;g__Coprococcus;s__	4.80E-03	1.10E+00	2.39E-01
296731	k__Bacteria;p__Firmicutes;c__Clostridia;o__Clostridiales;f__Ruminococcaceae;g__;s__	4.84E-03	1.04E+02	1.40E+02
820272	k__Bacteria;p__Firmicutes;c__Clostridia;o__Clostridiales;f__Lachnospiraceae;g__;s__	4.90E-03	4.65E-01	2.09E+00
New.ReferenceOTU740	k__Bacteria;p__Firmicutes;c__Clostridia;o__Clostridiales;f__Ruminococcaceae;g__Ruminococcus;s__	4.91E-03	5.92E-01	2.09E+00
316888	k__Bacteria;p__Firmicutes;c__Bacilli;o__Lactobacillales;f__Carnobacteriaceae;g__Desemzia;s__	5.04E-03	2.89E+00	2.24E-01

4370941	k__Bacteria;p__Firmicutes;c__Clostridia;o__Clostridiales;f__Ruminococcaceae;g__Oscillospira;s__	5.04E-03	8.59E-01	2.24E-01
319680	k__Bacteria;p__Firmicutes;c__Clostridia;o__Clostridiales;f__Lachnospiraceae;g__;s__	5.05E-03	1.17E+00	1.34E-01
1146865	k__Bacteria;p__Firmicutes;c__Bacilli;o__Lactobacillales;f__Streptococcaceae;g__Streptococcus;s__	5.10E-03	1.94E+00	6.87E-01
New.ReferenceOTU507	k__Bacteria;p__Firmicutes;c__Clostridia;o__Clostridiales;f__[Mogibacteriaceae];g__;s__	5.12E-03	9.01E-01	3.33E+00
New.CleanUp.ReferenceOTU57010	k__Bacteria;p__Firmicutes;c__Clostridia;o__Clostridiales;f__;g__;s__	5.13E-03	4.23E-02	3.58E-01
New.CleanUp.ReferenceOTU118948	k__Bacteria;p__Firmicutes;c__Clostridia;o__Clostridiales;f__Ruminococcaceae	5.19E-03	2.25E-01	5.97E-01
271320	k__Bacteria;p__Firmicutes;c__Clostridia;o__Clostridiales;f__[Mogibacteriaceae];g__;s__	5.19E-03	4.37E-01	1.79E+00
4345285	k__Bacteria;p__Firmicutes;c__Bacilli;o__Bacillales;f__Staphylococcaceae;g__Staphylococcus;s__	5.22E-03	2.75E+00	7.46E-02
New.CleanUp.ReferenceOTU42518	k__Bacteria;p__Bacteroidetes;c__Bacteroidia;o__Bacteroidales;f__[Paraprevotellaceae];g__YRC22;s__	5.24E-03	0.00E+00	7.01E-01
4326866	k__Bacteria;p__Firmicutes;c__Clostridia;o__Clostridiales;f__Ruminococcaceae;g__;s__	5.24E-03	3.04E+00	4.61E+00
4319005	k__Bacteria;p__Firmicutes;c__Clostridia;o__Clostridiales;f__;g__;s__	5.24E-03	0.00E+00	9.40E-01
New.CleanUp.ReferenceOTU52049	k__Bacteria;p__Bacteroidetes;c__Bacteroidia;o__Bacteroidales;f__;g__;s__	5.24E-03	0.00E+00	5.52E-01
339673	k__Bacteria;p__Firmicutes;c__Clostridia;o__Clostridiales;f__Christensenellaceae;g__;s__	5.28E-03	9.86E-02	1.04E+00
300458	k__Bacteria;p__Firmicutes;c__Clostridia;o__Clostridiales;f__Ruminococcaceae;g__;s__	5.28E-03	2.82E-01	8.06E-01
1995182	k__Bacteria;p__Firmicutes;c__Bacilli;o__Lactobacillales;f__Lactobacillaceae;g__Lactobacillus;s__	5.41E-03	2.36E+01	1.15E+01
193969	k__Bacteria;p__Firmicutes;c__Clostridia;o__Clostridiales;f__;g__;s__	5.43E-03	1.46E+00	1.49E-01
New.ReferenceOTU837	k__Bacteria;p__Firmicutes;c__Clostridia;o__Clostridiales;f__Ruminococcaceae;g__Ruminococcus;s__	5.49E-03	2.82E-02	6.72E-01
4372528	k__Bacteria;p__Firmicutes;c__Clostridia;o__Clostridiales;f__Clostridiaceae;g__SMB53;s__	5.49E-03	7.89E-01	8.96E-02
292285	k__Bacteria;p__Firmicutes;c__Clostridia;o__Clostridiales;f__;g__;s__	5.53E-03	1.69E-01	1.36E+00
290415	k__Bacteria;p__Firmicutes;c__Clostridia;o__Clostridiales;f__Ruminococcaceae;g__;s__	5.56E-03	1.59E+00	2.82E+00
290086	k__Bacteria;p__Firmicutes;c__Clostridia;o__Clostridiales;f__;g__;s__	5.61E-03	2.82E-02	4.03E-01
164216	k__Bacteria;p__Firmicutes;c__Clostridia;o__Clostridiales;f__Lachnospiraceae;g__;s__	5.64E-03	1.69E-01	5.82E-01
183147	k__Bacteria;p__Firmicutes;c__Clostridia;o__Clostridiales;f__Lachnospiraceae;g__;s__	5.70E-03	1.73E+00	3.43E-01
299592	k__Bacteria;p__Firmicutes;c__Clostridia;o__Clostridiales;f__Lachnospiraceae;g__Blautia;s__	5.72E-03	9.86E-02	6.57E-01
New.ReferenceOTU721	k__Bacteria;p__Firmicutes;c__Clostridia;o__Clostridiales;f__[Mogibacteriaceae];g__;s__	5.75E-03	3.24E-01	9.10E-01
299488	k__Bacteria;p__Bacteroidetes;c__Bacteroidia;o__Bacteroidales;f__;g__;s__	5.76E-03	1.89E+00	2.94E+00
1908049	k__Bacteria;p__Firmicutes;c__Clostridia;o__Clostridiales;f__Ruminococcaceae;g__;s__	5.83E-03	1.38E+01	2.43E+00
New.CleanUp.ReferenceOTU161145	k__Bacteria;p__Firmicutes;c__Clostridia;o__Clostridiales;f__[Mogibacteriaceae];g__;s__	5.90E-03	1.41E-02	6.27E-01
297730	k__Bacteria;p__Firmicutes;c__Clostridia;o__Clostridiales;f__Christensenellaceae;g__;s__	5.95E-03	8.45E-02	4.33E-01
New.CleanUp.ReferenceOTU22966	k__Bacteria;p__Firmicutes;c__Clostridia;o__Clostridiales;f__Lachnospiraceae;g__Blautia;s__producta	5.96E-03	1.41E-02	2.84E-01
4359317	k__Bacteria;p__Firmicutes;c__Bacilli;o__Lactobacillales;f__Streptococcaceae;g__Streptococcus;s__minor	6.02E-03	5.38E+00	1.59E+01
New.ReferenceOTU756	k__Bacteria;p__Firmicutes;c__Clostridia;o__Clostridiales;f__Lachnospiraceae;g__;s__	6.02E-03	6.76E-01	2.16E+00

105099	k__Bacteria;p__Firmicutes;c__Clostridia;o__Clostridiales;f__Lachnospiraceae;g__;s__	6.06E-03	2.82E-01	1.42E+00
110462	k__Bacteria;p__Firmicutes;c__Clostridia;o__Clostridiales;f__Christensenellaceae;g__;s__	6.08E-03	1.14E+00	2.84E-01
789561	k__Bacteria;p__Firmicutes;c__Clostridia;o__Clostridiales;f__;g__;s__	6.09E-03	3.66E-01	5.97E-02
New.CleanUp.ReferenceOTU63512	k__Bacteria;p__Firmicutes;c__Clostridia;o__Clostridiales;f__;g__;s__	6.09E-03	1.41E-02	2.54E-01
New.CleanUp.ReferenceOTU162252	k__Bacteria;p__Firmicutes;c__Clostridia;o__Clostridiales;f__Ruminococcaceae;g__;s__	6.15E-03	1.41E-02	2.99E-01
New.CleanUp.ReferenceOTU54737	k__Bacteria;p__Firmicutes;c__Bacilli;o__Lactobacillales;f__Lactobacillaceae;g__Lactobacillus;s__	6.18E-03	8.45E-01	1.49E-01
2532173	k__Bacteria;p__Firmicutes;c__Clostridia;o__Clostridiales;f__;g__;s__	6.18E-03	1.45E+00	0.00E+00
New.CleanUp.ReferenceOTU52958	k__Bacteria;p__Firmicutes;c__Clostridia;o__Clostridiales;f__;g__;s__	6.18E-03	1.97E-01	0.00E+00
289348	k__Bacteria;p__Firmicutes;c__Clostridia;o__Clostridiales;f__Lachnospiraceae;g__Coprococcus;s__	6.18E-03	3.21E+00	6.87E-01
573551	k__Bacteria;p__Firmicutes;c__Clostridia;o__Clostridiales;f__;g__;s__	6.18E-03	1.41E-01	7.61E-01
329761	k__Bacteria;p__Firmicutes;c__Clostridia;o__Clostridiales;f__;g__;s__	6.18E-03	2.11E-01	0.00E+00
4463030	k__Bacteria;p__Firmicutes;c__Clostridia;o__Clostridiales;f__Lachnospiraceae	6.18E-03	2.39E-01	0.00E+00
New.CleanUp.ReferenceOTU100792	k__Bacteria;p__Firmicutes;c__Clostridia;o__Clostridiales;f__;g__;s__	6.18E-03	2.11E-01	0.00E+00
New.ReferenceOTU372	k__Bacteria;p__Firmicutes;c__Clostridia;o__Clostridiales;f__Lachnospiraceae;g__;s__	6.19E-03	3.10E-01	0.00E+00
New.CleanUp.ReferenceOTU144625	k__Bacteria;p__Firmicutes;c__Clostridia;o__Clostridiales;f__Ruminococcaceae;g__;s__	6.19E-03	2.96E-01	0.00E+00
293097	k__Bacteria;p__Firmicutes;c__Clostridia;o__Clostridiales;f__Lachnospiraceae	6.19E-03	4.51E-01	0.00E+00
New.CleanUp.ReferenceOTU158189	k__Bacteria;p__Firmicutes;c__Clostridia;o__Clostridiales;f__Lachnospiraceae;g__;s__	6.20E-03	2.68E-01	0.00E+00
177153	k__Bacteria;p__Firmicutes;c__Clostridia;o__Clostridiales;f__;g__;s__	6.21E-03	3.10E-01	0.00E+00
182255	k__Bacteria;p__Bacteroidetes;c__Bacteroidia;o__Bacteroidales;f__Bacteroidaceae;g__Bacteroides;s__	6.22E-03	4.79E-01	8.96E-02
153291	k__Bacteria;p__Firmicutes;c__Clostridia;o__Clostridiales;f__;g__;s__	6.28E-03	5.66E+00	1.37E+00
306198	k__Bacteria;p__Firmicutes;c__Clostridia;o__Clostridiales;f__;g__;s__	6.30E-03	7.04E-02	1.25E+00
290679	k__Bacteria;p__Firmicutes;c__Clostridia;o__Clostridiales;f__;g__;s__	6.35E-03	1.69E-01	6.87E-01
300658	k__Bacteria;p__Firmicutes;c__Bacilli;o__Lactobacillales;f__Streptococcaceae;g__Streptococcus;s__	6.42E-03	1.27E+00	1.94E-01
New.ReferenceOTU523	k__Bacteria;p__Bacteroidetes;c__Bacteroidia;o__Bacteroidales;f__Prevotellaceae;g__Prevotella;s__	6.43E-03	3.10E-01	1.07E+00
3729748	k__Bacteria;p__Firmicutes;c__Clostridia;o__Clostridiales;f__Peptococcaceae;g__Peptococcus;s__	6.53E-03	1.27E-01	9.25E-01
110192	k__Bacteria;p__Firmicutes;c__Clostridia;o__Clostridiales;f__Ruminococcaceae;g__Oscillospira;s__	6.53E-03	9.30E-01	2.54E-01
293473	k__Bacteria;p__Firmicutes;c__Clostridia;o__Clostridiales;f__Ruminococcaceae;g__;s__	6.53E-03	1.32E+00	9.40E-01
74480	k__Bacteria;p__Firmicutes;c__Clostridia;o__Clostridiales;f__Ruminococcaceae;g__;s__	6.55E-03	6.48E-01	4.48E-02
4417708	k__Bacteria;p__Firmicutes;c__Clostridia;o__Clostridiales;f__;g__;s__	6.55E-03	1.41E+00	1.94E-01
190961	k__Bacteria;p__Firmicutes;c__Clostridia;o__Clostridiales;f__Lachnospiraceae	6.67E-03	1.29E+01	3.33E+00
4381548	k__Bacteria;p__Firmicutes;c__Clostridia;o__Clostridiales;f__Ruminococcaceae;g__;s__	6.69E-03	1.10E+00	5.85E+00
290607	k__Bacteria;p__Firmicutes;c__Clostridia;o__Clostridiales;f__Lachnospiraceae;g__;s__	6.70E-03	2.54E-01	4.52E+00

New.CleanUp.ReferenceOTU119821	k__Bacteria;p__Firmicutes;c__Clostridia;o__Clostridiales;f__Ruminococcaceae;g__;s__	6.80E-03	2.82E-02	3.58E-01
295417	k__Bacteria;p__Firmicutes;c__Clostridia;o__Clostridiales;f__Lachnospiraceae;g__;s__	6.81E-03	7.75E-01	1.48E+00
New.ReferenceOTU660	k__Bacteria;p__Firmicutes;c__Clostridia;o__Clostridiales;f__Eubacteriaceae;g__Pseudoramibacter_Eubacterium;s__	6.83E-03	9.72E-01	2.54E+00
New.CleanUp.ReferenceOTU20758	k__Bacteria;p__Spirochaetes;c__Spirochaetes;o__Spirochaetales;f__Spirochaetaceae;g__Treponema;s__	6.87E-03	2.82E-02	4.63E-01
New.CleanUp.ReferenceOTU76163	k__Bacteria;p__Firmicutes;c__Clostridia;o__Clostridiales;f__Lachnospiraceae;g__;s__	6.87E-03	2.82E-02	3.58E-01
191251	k__Bacteria;p__Bacteroidetes;c__Bacteroidia;o__Bacteroidales;f__Porphyromonadaceae;g__Parabacteroides;s__distasonis	6.88E-03	9.44E-01	1.34E-01
574551	k__Bacteria;p__Firmicutes;c__Clostridia;o__Clostridiales;f__Clostridiaceae;g__Clostridium;s__	6.89E-03	8.17E-01	7.46E-02
325340	k__Bacteria;p__Bacteroidetes;c__Bacteroidia;o__Bacteroidales;f__[Paraprevotellaceae];g__YRC22;s__	6.91E-03	1.13E-01	8.21E-01
New.CleanUp.ReferenceOTU28191	k__Bacteria;p__Firmicutes;c__Clostridia;o__Clostridiales;f__;g__;s__	6.92E-03	3.80E-01	7.46E-02
New.ReferenceOTU150	k__Bacteria;p__Firmicutes;c__Clostridia;o__Clostridiales;f__Lachnospiraceae;g__;s__	6.92E-03	1.13E-01	5.22E-01
New.CleanUp.ReferenceOTU23848	k__Bacteria;p__Firmicutes;c__Clostridia;o__Clostridiales;f__Ruminococcaceae;g__Oscillospira;s__	6.96E-03	7.18E-01	1.64E-01
363017	k__Bacteria;p__Firmicutes;c__Clostridia;o__Clostridiales;f__Ruminococcaceae;g__;s__	7.02E-03	2.82E-02	3.28E-01
New.CleanUp.ReferenceOTU122624	k__Bacteria;p__Firmicutes;c__Clostridia;o__Clostridiales	7.03E-03	2.82E-02	3.13E-01
509709	k__Bacteria;p__Firmicutes;c__Clostridia;o__Clostridiales;f__Lachnospiraceae;g__;s__	7.05E-03	6.83E+00	2.67E+00
New.ReferenceOTU321	k__Bacteria;p__Bacteroidetes;c__Bacteroidia;o__Bacteroidales;f__;g__;s__	7.17E-03	1.13E-01	1.64E+00
New.CleanUp.ReferenceOTU140005	k__Bacteria;p__Firmicutes;c__Clostridia;o__Clostridiales;f__Lachnospiraceae;g__Dorea;s__	7.17E-03	6.20E-01	4.48E-02
333939	k__Bacteria;p__Bacteroidetes;c__Bacteroidia;o__Bacteroidales;f__;g__;s__	7.19E-03	4.93E-01	7.91E-01
293393	k__Bacteria;p__Firmicutes;c__Clostridia;o__Clostridiales;f__Veillonellaceae;g__;s__	7.19E-03	4.23E-02	3.58E-01
188735	k__Bacteria;p__Bacteroidetes;c__Bacteroidia;o__Bacteroidales;f__Bacteroidaceae;g__Bacteroides;s__	7.20E-03	5.21E-01	1.49E-02
New.CleanUp.ReferenceOTU106879	k__Bacteria;p__Firmicutes;c__Clostridia;o__Clostridiales;f__Ruminococcaceae;g__;s__	7.24E-03	8.45E-02	4.18E-01
195166	k__Bacteria;p__Firmicutes;c__Clostridia;o__Clostridiales;f__Lachnospiraceae	7.27E-03	2.52E+00	4.57E+00
2420187	k__Bacteria;p__Firmicutes;c__Clostridia;o__Clostridiales;f__Lachnospiraceae;g__;s__	7.39E-03	4.93E-01	4.48E-02
New.CleanUp.ReferenceOTU40996	k__Bacteria;p__Firmicutes;c__Clostridia;o__Clostridiales;f__[Mogibacteriaceae];g__;s__	7.43E-03	2.82E-02	2.24E-01
552235	k__Bacteria;p__Firmicutes;c__Clostridia;o__Clostridiales;f__Christensenellaceae;g__Christensenella;s__	7.46E-03	4.23E-01	1.49E-02
New.CleanUp.ReferenceOTU13348	k__Bacteria;p__Firmicutes;c__Clostridia;o__Clostridiales;f__Lachnospiraceae;g__Blautia;s__	7.46E-03	5.77E-01	1.49E-02
628014	k__Bacteria;p__Firmicutes;c__Clostridia;o__Clostridiales;f__Lachnospiraceae;g__;s__	7.52E-03	7.04E-02	5.07E-01
344947	k__Bacteria;p__Firmicutes;c__Clostridia;o__Clostridiales;f__Lachnospiraceae;g__Blautia;s__	7.58E-03	6.62E-01	1.19E-01
4428313	k__Bacteria;p__Firmicutes;c__Bacilli;o__Lactobacillales;f__Lactobacillaceae;g__Lactobacillus;s__	7.60E-03	5.60E+01	2.80E+01
324975	k__Bacteria;p__Firmicutes;c__Clostridia;o__Clostridiales;f__Ruminococcaceae;g__;s__	7.64E-03	1.41E-01	6.57E-01
182764	k__Bacteria;p__Firmicutes;c__Bacilli;o__Lactobacillales;f__Lactobacillaceae;g__Lactobacillus;s__	7.72E-03	1.23E+01	6.30E+00
New.ReferenceOTU842	k__Bacteria;p__Bacteroidetes;c__Bacteroidia;o__Bacteroidales;f__Bacteroidaceae;g__BF311;s__	7.75E-03	2.25E-01	1.45E+00
290013	k__Bacteria;p__Firmicutes;c__Clostridia;o__Clostridiales;f__Lachnospiraceae;g__;s__	7.75E-03	1.27E-01	6.42E-01

298536	k__Bacteria;p__Firmicutes;c__Clostridia;o__Clostridiales;f__Lachnospiraceae;g__;s__	7.76E-03	1.08E+01	2.06E+00
New.CleanUp.ReferenceOTU153116	k__Bacteria;p__Proteobacteria;c__Deltaproteobacteria;o__Desulfovibrionales;f__Desulfovibrionaceae;g__Desulfovibrio;s__	7.76E-03	4.23E-01	1.49E-02
New.ReferenceOTU862	k__Bacteria;p__Firmicutes;c__Clostridia;o__Clostridiales;f__Clostridiaceae;g__Clostridium;s__	7.84E-03	1.58E+00	3.28E-01
New.ReferenceOTU280	k__Bacteria;p__Firmicutes;c__Clostridia;o__Clostridiales;f__Lachnospiraceae;g__;s__	7.85E-03	7.32E-01	9.25E-01
181167	k__Bacteria;p__Firmicutes;c__Clostridia;o__Clostridiales;f__Lachnospiraceae	7.86E-03	5.63E-01	2.99E-02
New.ReferenceOTU241	k__Bacteria;p__Firmicutes;c__Clostridia;o__Clostridiales;f__Lachnospiraceae;g__;s__	7.87E-03	1.27E-01	1.31E+00
158211	k__Bacteria;p__Firmicutes;c__Clostridia;o__Clostridiales;f__Lachnospiraceae;g__Blautia;s__	7.88E-03	2.25E-01	1.49E-02
New.ReferenceOTU539	k__Bacteria;p__Bacteroidetes;c__Bacteroidia;o__Bacteroidales;f__[Paraprevotellaceae];g__YRC22;s__	7.90E-03	3.89E+00	4.49E+00
196219	k__Bacteria;p__Bacteroidetes;c__Bacteroidia;o__Bacteroidales;f__Bacteroidaceae;g__Bacteroides;s__	7.91E-03	4.93E-01	7.46E-02
4420408	k__Bacteria;p__Bacteroidetes;c__Bacteroidia;o__Bacteroidales;f__Bacteroidaceae;g__Bacteroides;s__uniformis	7.92E-03	9.15E-01	5.97E-02
292753	k__Bacteria;p__Firmicutes;c__Clostridia;o__Clostridiales;f__Lachnospiraceae;g__;s__	8.03E-03	5.61E+00	9.28E+00
348574	k__Bacteria;p__Firmicutes;c__Clostridia;o__Clostridiales;f__Lachnospiraceae;g__;s__	8.14E-03	1.69E-01	7.01E-01
New.CleanUp.ReferenceOTU164171	k__Bacteria;p__Firmicutes;c__Clostridia;o__Clostridiales;f__;g__;s__	8.14E-03	5.35E-01	1.33E+00
New.CleanUp.ReferenceOTU88573	Unassigned	8.23E-03	5.63E-02	3.73E-01
New.ReferenceOTU884	k__Bacteria;p__Verrucomicrobia;c__Verruco-5;o__WCHB1-41;f__RFP12;g__;s__	8.45E-03	3.66E-01	1.33E+00
291949	k__Bacteria;p__Firmicutes;c__Clostridia;o__Clostridiales;f__Ruminococcaceae;g__;s__	8.46E-03	2.23E+00	3.52E+00
293067	k__Bacteria;p__Firmicutes;c__Clostridia;o__Clostridiales;f__Eubacteriaceae;g__Pseudoramibacter_Eubacterium;s__	8.62E-03	1.04E+00	2.61E+00
New.CleanUp.ReferenceOTU40642	k__Bacteria;p__Firmicutes;c__Erysipelotrichi;o__Erysipelotrichales;f__Erysipelotrichaceae;g__;s__	8.63E-03	1.15E+00	1.19E-01
New.CleanUp.ReferenceOTU122997	k__Bacteria;p__Firmicutes;c__Clostridia;o__Clostridiales;f__Christensenellaceae;g__;s__	8.63E-03	6.48E-01	1.94E-01
2148365	k__Bacteria;p__Firmicutes;c__Clostridia;o__Clostridiales;f__Lachnospiraceae;g__;s__	8.72E-03	2.08E+00	6.81E+00
New.ReferenceOTU397	k__Bacteria;p__Firmicutes;c__Clostridia;o__Clostridiales;f__;g__;s__	8.77E-03	9.86E-01	2.69E-01
295824	k__Bacteria;p__Firmicutes;c__Clostridia;o__Clostridiales;f__;g__;s__	8.79E-03	8.45E-02	8.36E-01
New.ReferenceOTU849	k__Bacteria;p__Firmicutes;c__Clostridia;o__Clostridiales;f__;g__;s__	8.79E-03	2.49E+00	8.96E-02
New.ReferenceOTU633	k__Bacteria;p__Firmicutes;c__Clostridia;o__Clostridiales;f__;g__;s__	8.86E-03	4.79E-01	2.49E+00
321348	k__Bacteria;p__Firmicutes;c__Clostridia;o__Clostridiales;f__Christensenellaceae;g__;s__	8.92E-03	1.55E-01	8.06E-01
New.ReferenceOTU335	k__Bacteria;p__Firmicutes;c__Clostridia;o__Clostridiales;f__Eubacteriaceae;g__Pseudoramibacter_Eubacterium;s__	9.10E-03	2.27E+00	4.21E+00
2545906	k__Bacteria;p__Bacteroidetes;c__Bacteroidia;o__Bacteroidales;f__Porphyromonadaceae;g__Parabacteroides;s__distasonis	9.14E-03	2.44E+00	4.63E-01
4435305	k__Bacteria;p__Firmicutes;c__Clostridia;o__Clostridiales;f__Ruminococcaceae;g__;s__	9.19E-03	6.90E-01	1.24E+00
New.ReferenceOTU178	k__Bacteria;p__Firmicutes;c__Clostridia;o__Clostridiales;f__Ruminococcaceae;g__Ruminococcus;s__	9.20E-03	2.68E-01	2.84E+00
New.CleanUp.ReferenceOTU87504	k__Bacteria;p__Actinobacteria;c__Coriobacteriia;o__Coriobacteriales;f__Coriobacteriaceae;g__;s__	9.21E-03	0.00E+00	1.94E-01
560677	k__Bacteria;p__Bacteroidetes;c__Bacteroidia;o__Bacteroidales;f__;g__;s__	9.21E-03	0.00E+00	5.67E-01
New.CleanUp.ReferenceOTU117661	k__Bacteria;p__Bacteroidetes;c__Bacteroidia;o__Bacteroidales;f__;g__;s__	9.22E-03	0.00E+00	2.69E-01

182647	k__Bacteria;p__Firmicutes;c__Clostridia;o__Clostridiales;f__;g__;s__	9.22E-03	0.00E+00	5.67E-01
New.CleanUp.ReferenceOTU14343	k__Bacteria;p__Firmicutes;c__Clostridia;o__Clostridiales;f__Ruminococcaceae;g__;s__	9.22E-03	0.00E+00	3.43E-01
298743	k__Bacteria;p__Bacteroidetes;c__Bacteroidia;o__Bacteroidales;f__RF16;g__;s__	9.22E-03	0.00E+00	5.22E-01
New.CleanUp.ReferenceOTU141183	k__Bacteria;p__Firmicutes;c__Clostridia;o__Clostridiales;f__;g__;s__	9.22E-03	0.00E+00	3.88E-01
New.CleanUp.ReferenceOTU91395	k__Bacteria;p__Firmicutes;c__Clostridia;o__Clostridiales;f__Ruminococcaceae;g__;s__	9.22E-03	0.00E+00	1.04E+00
185281	k__Bacteria;p__Firmicutes;c__Clostridia;o__Clostridiales;f__Lachnospiraceae;g__Dorea;s__	9.24E-03	4.37E-01	7.46E-02
New.CleanUp.ReferenceOTU121174	k__Bacteria;p__Firmicutes;c__Clostridia;o__Clostridiales;f__;g__;s__	9.29E-03	8.45E-02	3.73E-01
354971	k__Bacteria;p__Firmicutes;c__Bacilli;o__Lactobacillales;f__Lactobacillaceae;g__Lactobacillus;s__	9.38E-03	9.58E-01	1.94E-01
New.CleanUp.ReferenceOTU127670	k__Bacteria;p__Bacteroidetes;c__Bacteroidia;o__Bacteroidales;f__;g__;s__	9.46E-03	2.82E-02	3.73E-01
4409280	k__Bacteria;p__Firmicutes;c__Clostridia;o__Clostridiales;f__;g__;s__	9.53E-03	2.54E-01	1.01E+00
196453	k__Bacteria;p__Firmicutes;c__Clostridia;o__Clostridiales;f__Christensenellaceae;g__;s__	9.57E-03	3.38E-01	4.48E-02
192262	k__Bacteria;p__Firmicutes;c__Clostridia;o__Clostridiales;f__Ruminococcaceae;g__Ruminococcus;s__	9.59E-03	3.10E-01	4.48E-02
New.CleanUp.ReferenceOTU131032	k__Bacteria;p__Bacteroidetes;c__Bacteroidia;o__Bacteroidales;f__;g__;s__	9.79E-03	5.63E-02	1.06E+00
594119	k__Bacteria;p__Firmicutes;c__Clostridia;o__Clostridiales;f__Ruminococcaceae;g__;s__	9.80E-03	1.55E+00	7.91E-01
290146	k__Bacteria;p__Firmicutes;c__Clostridia;o__Clostridiales;f__Lachnospiraceae;g__Blautia;s__	9.80E-03	2.04E+00	4.09E+00
299426	k__Bacteria;p__Bacteroidetes;c__Bacteroidia;o__Bacteroidales;f__;g__;s__	9.89E-03	5.92E-01	3.54E+00
1522739	k__Bacteria;p__Firmicutes;c__Clostridia;o__Clostridiales;f__Clostridiaceae;g__;s__	9.91E-03	6.48E-01	1.79E-01
346119	k__Bacteria;p__Firmicutes;c__Clostridia;o__Clostridiales;f__Ruminococcaceae;g__;s__	9.97E-03	8.45E-02	6.12E-01
4467134	k__Bacteria;p__Verrucomicrobia;c__Verruco-5;o__WCHB1-41;f__RFP12;g__;s__	1.01E-02	1.69E-01	7.31E-01
343926	k__Bacteria;p__Verrucomicrobia;c__Verruco-5;o__WCHB1-41;f__RFP12;g__;s__	1.01E-02	2.82E-02	7.16E-01
New.CleanUp.ReferenceOTU164269	k__Bacteria;p__Firmicutes;c__Clostridia;o__Clostridiales;f__Ruminococcaceae;g__;s__	1.02E-02	2.39E-01	9.85E-01
725212	k__Bacteria;p__Firmicutes;c__Clostridia;o__Clostridiales;f__;g__;s__	1.02E-02	2.15E+00	7.01E-01
New.ReferenceOTU320	k__Bacteria;p__Firmicutes;c__Erysipelotrichi;o__Erysipelotrichales;f__Erysipelotrichaceae;g__p-75-a5;s__	1.03E-02	1.69E-01	7.91E-01
288106	k__Bacteria;p__Fibrobacteres;c__Fibrobacteria;o__Fibrobacterales;f__Fibrobacteraceae;g__Fibrobacter;s__succinogenes	1.03E-02	3.94E-01	1.51E+00
287724	k__Bacteria;p__Firmicutes;c__Clostridia;o__Clostridiales;f__Ruminococcaceae;g__;s__	1.03E-02	1.27E-01	5.97E-01
1654477	k__Bacteria;p__Fusobacteria;c__Fusobacteriia;o__Fusobacteriales;f__Fusobacteriaceae;g__Fusobacterium;s__	1.03E-02	5.65E+00	9.70E-01
309641	k__Bacteria;p__Bacteroidetes;c__Bacteroidia;o__Bacteroidales;f__;g__;s__	1.03E-02	1.41E-02	3.96E+00
New.ReferenceOTU817	k__Bacteria;p__Firmicutes;c__Clostridia;o__Clostridiales;f__Christensenellaceae;g__;s__	1.03E-02	3.52E-01	8.96E-01
New.CleanUp.ReferenceOTU42501	k__Bacteria;p__Firmicutes;c__Clostridia;o__Clostridiales;f__Ruminococcaceae;g__;s__	1.03E-02	1.07E+00	2.09E-01
179319	k__Bacteria;p__Firmicutes;c__Clostridia;o__Clostridiales;f__Lachnospiraceae;g__Dorea;s__	1.03E-02	2.39E-01	0.00E+00
1136387	k__Bacteria;p__Actinobacteria;c__Actinobacteria;o__Bifidobacteriales;f__Bifidobacteriaceae;g__;s__	1.03E-02	8.03E-01	8.96E-02
336232	k__Bacteria;p__Firmicutes;c__Clostridia;o__Clostridiales;f__Clostridiaceae;g__Sarcina;s__	1.03E-02	1.97E-01	7.16E-01

314853	k__Bacteria;p__Firmicutes;c__Clostridia;o__Clostridiales;f__[Mogibacteriaceae];g__;s__	1.03E-02	2.11E-01	0.00E+00
New.CleanUp.ReferenceOTU139005	k__Bacteria;p__Firmicutes;c__Bacilli;o__Lactobacillales;f__Carnobacteriaceae;g__Desemzia;s__	1.03E-02	2.25E-01	0.00E+00
New.CleanUp.ReferenceOTU127248	k__Bacteria;p__Firmicutes;c__Clostridia;o__Clostridiales;f__Lachnospiraceae	1.03E-02	2.39E-01	0.00E+00
85594	k__Bacteria;p__Firmicutes;c__Clostridia;o__Clostridiales;f__Veillonellaceae;g__Veillonella;s__dispar	1.03E-02	3.80E-01	0.00E+00
New.ReferenceOTU941	k__Bacteria;p__Firmicutes;c__Clostridia;o__Clostridiales;f__Lachnospiraceae;g__;s__	1.03E-02	4.23E-01	0.00E+00
296487	k__Bacteria;p__Firmicutes;c__Clostridia;o__Clostridiales;f__Lachnospiraceae;g__;s__	1.03E-02	2.54E-01	0.00E+00
197624	k__Bacteria;p__Firmicutes;c__Clostridia;o__Clostridiales;f__Ruminococcaceae;g__Oscillospira;s__	1.03E-02	3.52E-01	0.00E+00
New.CleanUp.ReferenceOTU160061	k__Bacteria;p__Firmicutes;c__Clostridia;o__Clostridiales;f__Ruminococcaceae;g__;s__	1.03E-02	4.08E-01	0.00E+00
New.ReferenceOTU420	k__Bacteria;p__Firmicutes;c__Clostridia;o__Clostridiales	1.03E-02	2.68E-01	0.00E+00
171547	k__Bacteria;p__Firmicutes;c__Clostridia;o__Clostridiales;f__Ruminococcaceae;g__;s__	1.03E-02	3.94E-01	0.00E+00
New.ReferenceOTU433	k__Bacteria;p__Actinobacteria;c__Coriobacteriia;o__Coriobacteriales;f__Coriobacteriaceae;g__;s__	1.03E-02	7.04E-02	3.43E-01
4356080	k__Bacteria;p__Bacteroidetes;c__Bacteroidia;o__Bacteroidales;f__[Barnesiellaceae];g__;s__	1.03E-02	6.34E-01	0.00E+00
845396	k__Bacteria;p__Firmicutes;c__Clostridia;o__Clostridiales;f__;g__;s__	1.03E-02	5.92E-01	0.00E+00
720093	k__Bacteria;p__Firmicutes;c__Clostridia;o__Clostridiales;f__Ruminococcaceae;g__;s__	1.03E-02	8.73E-01	0.00E+00
New.ReferenceOTU45	k__Bacteria;p__Firmicutes;c__Clostridia;o__Clostridiales;f__Ruminococcaceae;g__;s__	1.05E-02	1.28E+00	2.24E-01
New.CleanUp.ReferenceOTU55699	k__Bacteria;p__Actinobacteria;c__Coriobacteriia;o__Coriobacteriales;f__Coriobacteriaceae;g__;s__	1.05E-02	1.41E-02	2.69E-01
New.ReferenceOTU742	k__Bacteria;p__Bacteroidetes;c__Bacteroidia;o__Bacteroidales;f__;g__;s__	1.05E-02	4.08E-01	1.15E+00
413275	k__Bacteria;p__Firmicutes;c__Clostridia;o__Clostridiales;f__Ruminococcaceae;g__;s__	1.06E-02	4.17E+00	2.24E-01
301182	k__Bacteria;p__Firmicutes;c__Clostridia;o__Clostridiales;f__Ruminococcaceae;g__;s__	1.06E-02	1.41E-02	2.69E-01
New.CleanUp.ReferenceOTU19178	k__Bacteria;p__Firmicutes;c__Clostridia;o__Clostridiales;f__Lachnospiraceae;g__;s__	1.06E-02	1.41E-02	2.84E-01
New.ReferenceOTU121	k__Bacteria;p__Firmicutes;c__Clostridia;o__Clostridiales;f__Lachnospiraceae	1.08E-02	5.92E-01	1.49E-01
New.CleanUp.ReferenceOTU8329	k__Bacteria;p__Firmicutes;c__Clostridia;o__Clostridiales;f__Ruminococcaceae;g__Ruminococcus;s__	1.09E-02	8.45E-02	5.82E-01
105109	k__Bacteria;p__Firmicutes;c__Clostridia;o__Clostridiales;f__;g__;s__	1.09E-02	1.39E+00	3.34E+00
4357353	k__Bacteria;p__Firmicutes;c__Clostridia;o__Clostridiales;f__;g__;s__	1.09E-02	8.17E-01	4.48E-02
New.ReferenceOTU84	k__Bacteria;p__Firmicutes;c__Clostridia;o__Clostridiales;f__;g__;s__	1.10E-02	1.83E-01	7.31E-01
111003	k__Bacteria;p__Firmicutes;c__Clostridia;o__Clostridiales;f__Lachnospiraceae;g__;s__	1.11E-02	5.63E+00	1.19E+01
296661	k__Bacteria;p__Firmicutes;c__Clostridium;o__Clostridiales;f__Lachnospiraceae;g__;s__	1.12E-02	8.45E-02	3.58E-01
4434268	k__Bacteria;p__Firmicutes;c__Clostridia;o__Clostridiales;f__Eubacteriaceae;g__Pseudoramibacter_Eubacterium;s__	1.12E-02	1.87E+00	2.99E-02
302670	k__Bacteria;p__Spirochaetes;c__Spirochaetes;o__Spirochaetales;f__Spirochaetaceae;g__Treponema;s__	1.12E-02	3.66E-01	1.28E+00
New.CleanUp.ReferenceOTU78421	k__Bacteria;p__Firmicutes;c__Clostridia;o__Clostridiales;f__Lachnospiraceae;g__Blautia;s__	1.13E-02	4.08E-01	3.16E+00
4436046	k__Bacteria;p__Firmicutes;c__Clostridia;o__Clostridiales;f__Lachnospiraceae;g__Dorea;s__	1.13E-02	1.15E+01	2.91E+00
296697	k__Bacteria;p__Firmicutes;c__Clostridia;o__Clostridiales;f__Lachnospiraceae;g__;s__	1.13E-02	4.23E-02	3.88E-01

99331	k__Bacteria;p__Firmicutes;c__Bacilli;o__Bacillales;f__Bacillaceae;g__Bacillus;s__	1.14E-02	2.90E+01	1.42E+01
305146	k__Bacteria;p__Firmicutes;c__Clostridia;o__Clostridiales;f__Ruminococcaceae;g__;s__	1.14E-02	2.39E-01	2.13E+00
290037	k__Bacteria;p__Armatimonadetes;c__SJA-176;o__RB046;f__;g__;s__	1.15E-02	4.08E-01	6.72E-01
365536	k__Bacteria;p__Firmicutes;c__Clostridia;o__Clostridiales;f__Ruminococcaceae;g__Oscillospira;s__	1.15E-02	3.94E-01	4.48E-02
305214	k__Bacteria;p__Firmicutes;c__Clostridia;o__Clostridiales;f__;g__;s__	1.15E-02	1.41E-01	1.24E+00
42492	k__Bacteria;p__Firmicutes;c__Clostridia;o__Clostridiales;f__Lachnospiraceae;g__;s__	1.16E-02	7.46E-01	1.64E+00
New.CleanUp.ReferenceOTU74444	k__Bacteria;p__Proteobacteria;c__Alphaproteobacteria;o__RF32;f__;g__;s__	1.16E-02	2.82E-02	6.72E-01
299108	k__Bacteria;p__Firmicutes;c__Clostridia;o__Clostridiales;f__[Mogibacteriaceae];g__Mogibacterium;s__	1.17E-02	2.75E+00	4.51E+00
147100	k__Bacteria;p__Firmicutes;c__Clostridia;o__Clostridiales;f__Ruminococcaceae;g__;s__	1.18E-02	1.72E+00	2.54E-01
New.ReferenceOTU353	k__Archaea;p__Euryarchaeota;c__Methanobacteria;o__Methanobacteriales;f__Methanobacteriaceae;g__Methanobrevibacter;s__	1.18E-02	5.21E-01	1.33E+00
New.ReferenceOTU454	k__Bacteria;p__Bacteroidetes;c__Bacteroidia;o__Bacteroidales;f__[Paraprevotellaceae];g__CF231;s__	1.18E-02	3.96E+00	1.34E-01
New.ReferenceOTU20	k__Bacteria;p__Firmicutes;c__Clostridia;o__Clostridiales;f__Christensenellaceae;g__;s__	1.19E-02	8.03E-01	1.94E-01
1030770	k__Bacteria;p__Tenericutes;c__Mollicutes;o__RF39;f__;g__;s__	1.20E-02	8.45E-02	1.01E+00
196898	k__Bacteria;p__Firmicutes;c__Clostridia;o__Clostridiales;f__Ruminococcaceae;g__;s__	1.21E-02	3.38E-01	8.96E-02
300618	k__Bacteria;p__Firmicutes;c__Clostridia;o__Clostridiales;f__;g__;s__	1.21E-02	4.39E+00	1.79E+00
293977	k__Bacteria;p__Firmicutes;c__Clostridia;o__Clostridiales;f__Christensenellaceae;g__;s__	1.21E-02	9.86E-02	8.36E-01
4381878	k__Bacteria;p__Firmicutes;c__Bacilli;o__Bacillales;f__Planococcaceae;g__Planomicrobium;s__	1.23E-02	5.38E+00	2.58E+00
New.CleanUp.ReferenceOTU96696	k__Bacteria;p__Verrucomicrobia;c__Verruco-5;o__WCHB1-41;f__RFP12;g__;s__	1.24E-02	4.23E-02	2.84E-01
4296366	k__Bacteria;p__Firmicutes;c__Bacilli;o__Bacillales;f__Planococcaceae;g__;s__	1.25E-02	3.80E-01	1.49E-02
New.CleanUp.ReferenceOTU37986	k__Bacteria;p__Bacteroidetes;c__Bacteroidia;o__Bacteroidales;f__[Paraprevotellaceae];g__;s__	1.25E-02	1.97E-01	1.72E+00
New.ReferenceOTU225	k__Bacteria;p__Firmicutes;c__Bacilli;o__Lactobacillales;f__Carnobacteriaceae;g__Desemzia;s__	1.25E-02	4.79E-01	1.49E-02
New.CleanUp.ReferenceOTU43165	k__Bacteria;p__Firmicutes;c__Clostridia;o__Clostridiales;f__Ruminococcaceae;g__;s__	1.25E-02	6.48E-01	2.99E-02
185411	k__Bacteria;p__Firmicutes;c__Clostridia;o__Clostridiales;f__Lachnospiraceae;g__Roseburia;s__	1.25E-02	6.20E-01	2.99E-02
191052	k__Bacteria;p__Firmicutes;c__Clostridia;o__Clostridiales;f__Lachnospiraceae	1.25E-02	1.10E+00	1.94E-01
New.ReferenceOTU230	k__Bacteria;p__Verrucomicrobia;c__Verrucomicrobiae;o__Verrucomicrobiales;f__Verrucomicrobiaceae;g__Akkermansia;s__	1.25E-02	4.23E-01	1.40E+00
168625	k__Bacteria;p__Firmicutes;c__Clostridia;o__Clostridiales;f__;g__;s__	1.25E-02	1.41E-01	6.57E-01
190913	k__Bacteria;p__Bacteroidetes;c__Bacteroidia;o__Bacteroidales;f__Porphyromonadaceae;g__Parabacteroides;s__	1.26E-02	4.37E-01	1.49E-02
New.CleanUp.ReferenceOTU118339	k__Bacteria;p__Firmicutes;c__Clostridia;o__Clostridiales;f__Lachnospiraceae;g__;s__	1.27E-02	7.04E-02	5.97E-01
238208	k__Bacteria;p__Firmicutes;c__Bacilli;o__Bacillales;f__Planococcaceae;g__Lysinibacillus;s__boronitolerans	1.27E-02	2.74E+01	2.20E+01
New.CleanUp.ReferenceOTU9926	k__Bacteria;p__Firmicutes;c__Clostridia;o__Clostridiales;f__Ruminococcaceae;g__;s__	1.27E-02	1.83E-01	6.57E-01
New.ReferenceOTU134	k__Bacteria;p__Firmicutes;c__Clostridia;o__Clostridiales;f__Ruminococcaceae;g__;s__	1.28E-02	2.54E-01	1.28E+00
New.CleanUp.ReferenceOTU42736	k__Bacteria;p__Firmicutes;c__Clostridia;o__Clostridiales;f__Christensenellaceae;g__;s__	1.28E-02	7.61E-01	1.49E-01

646499	k__Bacteria;p__Firmicutes;c__Clostridia;o__Clostridiales;f__;g__;s__	1.28E-02	8.45E-02	3.73E-01
324112	k__Bacteria;p__Firmicutes;c__Clostridia;o__Clostridiales;f__;g__;s__	1.28E-02	4.97E+00	5.67E-01
New.CleanUp.ReferenceOTU31707	k__Bacteria;p__Firmicutes;c__Clostridia;o__Clostridiales;f__Lachnospiraceae;g__;s__	1.28E-02	1.69E-01	3.43E-01
455746	k__Bacteria;p__Firmicutes;c__Clostridia;o__Clostridiales;f__Lachnospiraceae;g__;s__	1.29E-02	1.03E+00	2.33E+00
518065	k__Bacteria;p__Actinobacteria;c__Actinobacteria;o__Actinomycetales;f__Corynebacteriaceae;g__Corynebacterium;s__	1.29E-02	5.92E-01	1.49E-02
536989	k__Bacteria;p__Firmicutes;c__Clostridia;o__Clostridiales;f__;g__;s__	1.29E-02	2.32E+00	1.49E-02
351126	k__Bacteria;p__Firmicutes;c__Clostridia;o__Clostridiales;f__Ruminococcaceae;g__;s__	1.29E-02	7.04E-02	4.63E-01
New.CleanUp.ReferenceOTU12933	k__Bacteria;p__Verrucomicrobia;c__Verrucomicrobiae;o__Verrucomicrobiales;f__Verrucomicrobiaceae;g__Akkermansia;s__muciniphila	1.30E-02	1.21E+00	2.39E-01
4458227	k__Bacteria;p__Firmicutes;c__Clostridia;o__Clostridiales;f__;g__;s__	1.30E-02	5.49E-01	2.99E-02
New.CleanUp.ReferenceOTU90	k__Bacteria;p__Firmicutes;c__Clostridia;o__Clostridiales;f__Ruminococcaceae;g__Ruminococcus;s__	1.30E-02	3.52E-01	1.49E-02
337246	k__Bacteria;p__Firmicutes;c__Clostridia;o__Clostridiales;f__;g__;s__	1.31E-02	2.11E-01	1.49E-02
342575	k__Bacteria;p__Firmicutes;c__Clostridia;o__Clostridiales;f__Ruminococcaceae;g__;s__	1.32E-02	8.87E-01	1.19E-01
294219	k__Bacteria;p__Firmicutes;c__Clostridia;o__Clostridiales;f__Lachnospiraceae;g__[Ruminococcus];s__gnavus	1.33E-02	7.89E-01	5.97E-02
New.CleanUp.ReferenceOTU33135	k__Bacteria;p__Firmicutes;c__Clostridia;o__Clostridiales;f__Ruminococcaceae;g__;s__	1.34E-02	2.11E-01	5.52E-01
New.CleanUp.ReferenceOTU40927	k__Bacteria;p__Firmicutes;c__Clostridia;o__Clostridiales;f__Ruminococcaceae;g__;s__	1.35E-02	8.03E-01	1.79E-01
337468	k__Bacteria;p__Firmicutes;c__Clostridia;o__Clostridiales;f__Ruminococcaceae;g__Oscillospira;s__	1.36E-02	4.13E+00	2.12E+00
175535	k__Bacteria;p__Bacteroidetes;c__Bacteroidia;o__Bacteroidales;f__Bacteroidaceae;g__Bacteroides;s__	1.36E-02	7.18E-01	8.96E-02
1992	k__Bacteria;p__Bacteroidetes;c__Bacteroidia;o__Bacteroidales;f__Bacteroidaceae;g__Bacteroides;s__ovatus	1.36E-02	7.32E-01	1.19E-01
New.CleanUp.ReferenceOTU3646	k__Bacteria;p__Firmicutes;c__Clostridia;o__Clostridiales;f__Ruminococcaceae;g__;s__	1.39E-02	3.66E-01	9.40E-01
318959	k__Bacteria;p__Firmicutes;c__Clostridia;o__Clostridiales;f__Ruminococcaceae;g__;s__	1.40E-02	2.28E+00	2.54E-01
New.CleanUp.ReferenceOTU154333	k__Bacteria;p__Firmicutes;c__Clostridia;o__Clostridiales;f__Lachnospiraceae;g__Dorea;s__	1.42E-02	6.76E-01	1.79E-01
New.CleanUp.ReferenceOTU151654	k__Bacteria;p__Firmicutes;c__Clostridia;o__Clostridiales;f__Lachnospiraceae;g__Dorea;s__	1.43E-02	4.37E-01	5.97E-02
293575	k__Bacteria;p__Firmicutes;c__Clostridia;o__Clostridiales;f__Ruminococcaceae;g__;s__	1.43E-02	1.13E+00	1.49E-01
New.CleanUp.ReferenceOTU170390	k__Bacteria;p__Firmicutes;c__Clostridia;o__Clostridiales;f__Lachnospiraceae;g__Dorea;s__	1.43E-02	6.90E-01	8.96E-02
New.ReferenceOTU503	k__Bacteria;p__Firmicutes;c__Clostridia;o__Clostridiales;f__Lachnospiraceae;g__;s__	1.44E-02	5.92E-01	1.04E+00
New.CleanUp.ReferenceOTU167381	k__Bacteria;p__Firmicutes;c__Clostridia;o__Clostridiales;f__Ruminococcaceae;g__;s__	1.44E-02	2.39E-01	6.42E-01
588624	k__Bacteria;p__Firmicutes;c__Clostridia;o__Clostridiales;f__Ruminococcaceae;g__Ruminococcus;s__	1.46E-02	2.82E-01	9.85E-01
289388	k__Bacteria;p__Firmicutes;c__Bacilli;o__Bacillales;f__Planococcaceae;g__;s__	1.48E-02	1.76E+00	5.97E-02
568968	k__Bacteria;p__Firmicutes;c__Clostridia;o__Clostridiales;f__;g__;s__	1.48E-02	8.87E-01	7.46E-02
New.CleanUp.ReferenceOTU24612	k__Bacteria;p__Tenericutes;c__Mollicutes;o__RF39;f__;g__;s__	1.48E-02	3.38E-01	2.99E-02
New.ReferenceOTU273	k__Bacteria;p__Bacteroidetes;c__Bacteroidia;o__Bacteroidales;f__;g__;s__	1.48E-02	1.01E+00	3.90E+00
New.ReferenceOTU759	k__Bacteria;p__Firmicutes;c__Clostridia;o__Clostridiales;f__Lachnospiraceae;g__;s__	1.48E-02	4.65E-01	8.96E-02

953855	k__Bacteria;p__Bacteroidetes;c__Bacteroidia;o__Bacteroidales;f__Rikenellaceae;g__;s__	1.48E-02	2.08E+00	9.10E-01
New.CleanUp.ReferenceOTU39278	k__Bacteria;p__Firmicutes;c__Clostridia;o__Clostridiales;f__Lachnospiraceae	1.48E-02	7.04E-02	4.93E-01
298928	k__Bacteria;p__Bacteroidetes;c__Bacteroidia;o__Bacteroidales;f__;g__;s__	1.49E-02	4.79E-01	1.31E+00
New.CleanUp.ReferenceOTU54613	k__Bacteria;p__Firmicutes;c__Clostridia;o__Clostridiales;f__;g__;s__	1.49E-02	1.38E+00	1.49E-01
New.CleanUp.ReferenceOTU8428	k__Bacteria;p__Firmicutes;c__Clostridia;o__Clostridiales;f__Clostridiaceae;g__Sarcina;s__	1.51E-02	2.82E-01	6.87E-01
296342	k__Bacteria;p__Bacteroidetes;c__Bacteroidia;o__Bacteroidales;f__;g__;s__	1.53E-02	1.55E+00	7.16E+00
293666	k__Bacteria;p__Firmicutes;c__Clostridia;o__Clostridiales;f__Lachnospiraceae;g__Blautia;s__	1.53E-02	8.45E-02	7.01E-01
312347	k__Bacteria;p__Firmicutes;c__Clostridia;o__Clostridiales;f__Ruminococcaceae;g__;s__	1.53E-02	1.37E+01	6.81E+00
324236	k__Bacteria;p__Firmicutes;c__Clostridia;o__Clostridiales;f__Lachnospiraceae;g__[Ruminococcus];s__	1.53E-02	5.77E-01	5.97E-02
New.ReferenceOTU509	k__Bacteria;p__Spirochaetes;c__MVP-15;o__PL-11B10;f__;g__;s__	1.53E-02	9.30E+00	1.88E+01
288723	k__Bacteria;p__Firmicutes;c__Bacilli;o__Lactobacillales;f__Lactobacillaceae;g__Lactobacillus;s__	1.54E-02	8.45E-01	2.39E-01
304883	k__Bacteria;p__Firmicutes;c__Clostridia;o__Clostridiales;f__;g__;s__	1.55E-02	1.39E+00	2.76E+00
New.ReferenceOTU733	k__Bacteria;p__Actinobacteria;c__Coriobacteriia;o__Coriobacteriales;f__Coriobacteriaceae;g__Adlercreutzia;s__	1.56E-02	1.55E-01	7.91E-01
New.CleanUp.ReferenceOTU169984	k__Bacteria;p__Proteobacteria;c__Deltaproteobacteria;o__Desulfovibrionales;f__Desulfovibrionaceae;g__Desulfovibrio;s__	1.56E-02	4.23E-02	3.13E-01
New.ReferenceOTU487	Unassigned	1.57E-02	2.82E-02	5.37E-01
313274	k__Bacteria;p__Firmicutes;c__Clostridia;o__Clostridiales;f__Ruminococcaceae;g__Oscillospira;s__	1.59E-02	2.96E-01	7.46E-02
291998	k__Bacteria;p__Firmicutes;c__Clostridia;o__Clostridiales;f__Lachnospiraceae;g__;s__	1.59E-02	3.10E-01	1.00E+00
4317038	k__Bacteria;p__Firmicutes;c__Clostridia;o__Clostridiales;f__;g__;s__	1.59E-02	0.00E+00	1.49E-01
628517	k__Bacteria;p__Lentisphaerae;c__[Lentisphaeria];o__Victivallales;f__Victivallaceae;g__;s__	1.59E-02	0.00E+00	6.72E-01
New.ReferenceOTU410	k__Bacteria;p__Firmicutes;c__Clostridia;o__Clostridiales;f__Ruminococcaceae;g__;s__	1.59E-02	0.00E+00	1.64E-01
New.CleanUp.ReferenceOTU170473	k__Bacteria;p__Firmicutes;c__Clostridia;o__Clostridiales;f__Lachnospiraceae;g__;s__	1.59E-02	0.00E+00	1.79E-01
345788	k__Bacteria;p__Firmicutes;c__Clostridia;o__Clostridiales;f__Christensenellaceae;g__;s__	1.59E-02	0.00E+00	1.79E-01
New.CleanUp.ReferenceOTU98597	k__Bacteria;p__Firmicutes;c__Clostridia;o__Clostridiales;f__Lachnospiraceae;g__Blautia;s__	1.59E-02	0.00E+00	1.79E-01
New.CleanUp.ReferenceOTU76844	k__Bacteria;p__Firmicutes;c__Clostridia;o__Clostridiales;f__[Mogibacteriaceae];g__;s__	1.59E-02	0.00E+00	1.94E-01
New.CleanUp.ReferenceOTU102172	k__Bacteria;p__Firmicutes;c__Clostridia;o__Clostridiales;f__Ruminococcaceae;g__;s__	1.59E-02	0.00E+00	1.94E-01
New.ReferenceOTU697	k__Bacteria;p__Firmicutes;c__Clostridia;o__Clostridiales;f__Veillonellaceae;g__;s__	1.59E-02	0.00E+00	2.54E-01
New.CleanUp.ReferenceOTU37183	k__Bacteria;p__Firmicutes;c__Clostridia;o__Clostridiales;f__Ruminococcaceae;g__;s__	1.59E-02	0.00E+00	2.09E-01
New.CleanUp.ReferenceOTU178742	k__Bacteria;p__Firmicutes;c__Clostridia;o__Clostridiales;f__Dehalobacteriaceae;g__Dehalobacterium;s__	1.59E-02	0.00E+00	2.54E-01
New.CleanUp.ReferenceOTU7146	k__Bacteria;p__Verrucomicrobia;c__[Pedosphaerae];o__[Pedosphaerales];f__R4-41B;g__;s__	1.59E-02	0.00E+00	2.39E-01
New.ReferenceOTU311	k__Bacteria;p__Firmicutes;c__Clostridia;o__Clostridiales;f__Lachnospiraceae;g__;s__	1.59E-02	9.30E-01	1.49E+00
New.CleanUp.ReferenceOTU13952	k__Bacteria;p__Spirochaetes;c__Spirochaetes;o__Spirochaetales;f__Spirochaetaceae;g__Treponema;s__	1.59E-02	0.00E+00	2.39E-01
109496	k__Bacteria;p__Firmicutes;c__Clostridia;o__Clostridiales;f__;g__;s__	1.59E-02	0.00E+00	4.33E-01

New.CleanUp.ReferenceOTU123344	k__Bacteria;p__Firmicutes;c__Clostridia;o__Clostridiales;f__Lachnospiraceae;g__[Ruminococcus];s__gnavus	1.59E-02	0.00E+00	3.73E-01
New.CleanUp.ReferenceOTU166320	k__Bacteria;p__Firmicutes;c__Erysipelotrichi;o__Erysipelotrichales;f__Erysipelotrichaceae;g__p-75-a5;s__	1.59E-02	0.00E+00	4.18E-01
2256432	k__Bacteria;p__Firmicutes;c__Clostridia;o__Clostridiales;f__Lachnospiraceae;g__Dorea;s__	1.59E-02	8.45E-01	4.33E-01
331526	k__Bacteria;p__Verrucomicrobia;c__[Pedosphaerae];o__[Pedosphaerales];f__R4-41B;g__;s__	1.59E-02	0.00E+00	6.42E-01
4421273	k__Bacteria;p__Firmicutes;c__Clostridia;o__Clostridiales;f__Ruminococcaceae;g__Oscillospira;s__	1.59E-02	8.31E-01	1.34E-01
New.CleanUp.ReferenceOTU59399	k__Bacteria;p__Firmicutes;c__Clostridia;o__Clostridiales;f__Lachnospiraceae;g__;s__	1.59E-02	2.82E-02	1.00E+00
307638	k__Bacteria;p__Firmicutes;c__Clostridia;o__Clostridiales;f__;g__;s__	1.59E-02	4.51E-01	1.79E+00
178064	k__Bacteria;p__Bacteroidetes;c__Bacteroidia;o__Bacteroidales;f__Bacteroidaceae;g__Bacteroides;s__	1.61E-02	4.65E-01	8.96E-02
New.CleanUp.ReferenceOTU169899	k__Bacteria;p__Firmicutes;c__Clostridia;o__Clostridiales;f__Ruminococcaceae;g__;s__	1.61E-02	1.27E-01	5.22E-01
New.CleanUp.ReferenceOTU26362	k__Bacteria;p__Firmicutes;c__Clostridia;o__Clostridiales	1.64E-02	4.23E-02	2.69E-01
4318552	k__Bacteria;p__Firmicutes;c__Clostridia;o__Clostridiales;f__;g__;s__	1.64E-02	4.23E-02	2.99E-01
193233	k__Bacteria;p__Bacteroidetes;c__Bacteroidia;o__Bacteroidales;f__Bacteroidaceae;g__Bacteroides;s__	1.64E-02	5.35E-01	1.49E-01
New.CleanUp.ReferenceOTU165242	k__Bacteria;p__Firmicutes;c__Clostridia;o__Clostridiales;f__Lachnospiraceae	1.64E-02	3.66E-01	4.63E-01
350910	k__Bacteria;p__Firmicutes;c__Clostridia;o__Clostridiales;f__Ruminococcaceae;g__;s__	1.64E-02	1.55E-01	7.76E-01
4343627	k__Bacteria;p__Bacteroidetes;c__Bacteroidia;o__Bacteroidales;f__Bacteroidaceae;g__Bacteroides;s__fragilis	1.64E-02	2.59E+01	1.14E+01
New.CleanUp.ReferenceOTU33185	k__Bacteria;p__Firmicutes;c__Clostridia;o__Clostridiales;f__Christensenellaceae;g__;s__	1.65E-02	4.93E-01	1.04E-01
New.ReferenceOTU413	k__Bacteria;p__Spirochaetes;c__Spirochaetes;o__Spirochaetales;f__Spirochaetaceae;g__Treponema;s__	1.66E-02	2.82E-02	6.27E-01
3680006	k__Bacteria;p__Firmicutes;c__Clostridia;o__Clostridiales;f__Lachnospiraceae;g__Dorea;s__	1.67E-02	4.93E-01	1.04E-01
4094866	k__Bacteria;p__Firmicutes;c__Clostridia;o__Clostridiales;f__Lachnospiraceae;g__;s__	1.67E-02	3.38E-01	1.58E+00
115315	k__Bacteria;p__Actinobacteria;c__Actinobacteria;o__Actinomycetales;f__Yaniellaceae;g__Yaniella;s__	1.68E-02	1.01E+00	1.94E-01
172233	k__Bacteria;p__Firmicutes;c__Clostridia;o__Clostridiales;f__Ruminococcaceae;g__;s__	1.68E-02	1.69E-01	0.00E+00
New.CleanUp.ReferenceOTU20734	k__Bacteria;p__Firmicutes;c__Bacilli;o__Lactobacillales;f__Carnobacteriaceae;g__Desemzia;s__	1.68E-02	1.69E-01	0.00E+00
300383	k__Bacteria;p__Firmicutes;c__Clostridia;o__Clostridiales;f__Lachnospiraceae;g__Coprococcus;s__	1.68E-02	3.52E-01	1.81E+00
195035	k__Bacteria;p__Firmicutes;c__Clostridia;o__Clostridiales;f__;g__;s__	1.68E-02	1.97E-01	0.00E+00
New.CleanUp.ReferenceOTU31828	k__Bacteria;p__Firmicutes;c__Bacilli;o__Lactobacillales;f__Lactobacillaceae;g__Lactobacillus	1.68E-02	2.25E-01	0.00E+00
New.CleanUp.ReferenceOTU85922	k__Bacteria;p__Firmicutes;c__Clostridia;o__Clostridiales;f__Lachnospiraceae;g__Blautia;s__	1.68E-02	2.11E-01	0.00E+00
4381749	k__Bacteria;p__Firmicutes;c__Clostridia;o__Clostridiales;f__Ruminococcaceae;g__;s__	1.68E-02	5.77E-01	1.19E-01
New.CleanUp.ReferenceOTU6550	k__Bacteria;p__Firmicutes;c__Clostridia;o__Clostridiales;f__;g__;s__	1.68E-02	2.11E-01	0.00E+00
New.CleanUp.ReferenceOTU97839	k__Bacteria;p__Firmicutes;c__Clostridia;o__Clostridiales;f__Lachnospiraceae;g__;s__	1.68E-02	2.11E-01	0.00E+00
4178726	k__Bacteria;p__Firmicutes;c__Clostridia;o__Clostridiales;f__Lachnospiraceae	1.68E-02	1.48E+00	0.00E+00
New.CleanUp.ReferenceOTU147725	k__Bacteria;p__Firmicutes;c__Clostridia;o__Clostridiales;f__Lachnospiraceae;g__;s__	1.68E-02	2.11E-01	0.00E+00
New.CleanUp.ReferenceOTU176679	k__Bacteria;p__Firmicutes;c__Clostridia;o__Clostridiales;f__Lachnospiraceae;g__Blautia;s__producta	1.68E-02	2.11E-01	0.00E+00

363321	k__Bacteria;p__Firmicutes;c__Clostridia;o__Clostridiales;f__Lachnospiraceae;g__Dorea;s__	1.68E-02	2.68E-01	0.00E+00
New.CleanUp.ReferenceOTU101749	k__Bacteria;p__Firmicutes;c__Clostridia;o__Clostridiales;f__Ruminococcaceae;g__;s__	1.68E-02	2.39E-01	0.00E+00
3855938	k__Bacteria;p__Firmicutes;c__Erysipelotrichi;o__Erysipelotrichales;f__Erysipelotrichaceae;g__Coprobacillus;s__	1.68E-02	2.39E-01	0.00E+00
New.CleanUp.ReferenceOTU2374	k__Bacteria;p__Firmicutes;c__Clostridia;o__Clostridiales;f__;g__;s__	1.68E-02	2.54E-01	0.00E+00
New.CleanUp.ReferenceOTU172657	k__Bacteria;p__Firmicutes;c__Bacilli;o__Bacillales;f__Staphylococcaceae;g__Jeotgalicoccus;s__	1.68E-02	3.66E-01	0.00E+00
550807	k__Bacteria;p__Firmicutes;c__Clostridia;o__Clostridiales;f__;g__;s__	1.68E-02	2.68E-01	0.00E+00
553150	k__Bacteria;p__Firmicutes;c__Clostridia;o__Clostridiales;f__Lachnospiraceae;g__Coprococcus;s__	1.68E-02	2.54E-01	0.00E+00
New.CleanUp.ReferenceOTU104805	k__Bacteria;p__Firmicutes;c__Clostridia;o__Clostridiales;f__Lachnospiraceae;g__Blautia;s__	1.68E-02	3.24E-01	0.00E+00
New.CleanUp.ReferenceOTU24446	k__Bacteria;p__Firmicutes;c__Clostridia;o__Clostridiales;f__Lachnospiraceae	1.68E-02	3.24E-01	0.00E+00
New.CleanUp.ReferenceOTU119524	k__Bacteria;p__Firmicutes;c__Clostridia;o__Clostridiales;f__Ruminococcaceae;g__Oscillospira;s__	1.68E-02	4.51E-01	0.00E+00
190241	k__Bacteria;p__Firmicutes;c__Clostridia;o__Clostridiales;f__Lachnospiraceae;g__;s__	1.68E-02	2.52E+00	0.00E+00
48899	k__Bacteria;p__Firmicutes;c__Clostridia;o__Clostridiales;f__;g__;s__	1.70E-02	2.62E+00	3.73E-01
299875	k__Bacteria;p__Firmicutes;c__Clostridia;o__Clostridiales;f__Ruminococcaceae;g__;s__	1.72E-02	2.82E-02	4.93E-01
302809	k__Bacteria;p__Firmicutes;c__Clostridia;o__Clostridiales;f__Ruminococcaceae;g__;s__	1.72E-02	2.49E+00	1.16E+00
3946926	k__Bacteria;p__Firmicutes;c__Bacilli;o__Lactobacillales;f__Lactobacillaceae;g__Lactobacillus;s__	1.74E-02	1.18E+00	1.19E-01
173245	k__Bacteria;p__Firmicutes;c__Clostridia;o__Clostridiales;f__;g__;s__	1.77E-02	1.07E+00	7.46E-02
290498	k__Bacteria;p__Firmicutes;c__Clostridia;o__Clostridiales;f__;g__;s__	1.78E-02	9.58E-01	1.88E+00
New.CleanUp.ReferenceOTU83219	k__Bacteria;p__Firmicutes;c__Clostridia;o__Clostridiales;f__Ruminococcaceae;g__;s__	1.79E-02	1.55E-01	5.97E-01
New.CleanUp.ReferenceOTU149788	k__Bacteria;p__Firmicutes;c__Clostridia;o__Clostridiales;f__Christensenellaceae;g__;s__	1.79E-02	2.82E-02	2.84E-01
New.CleanUp.ReferenceOTU160291	k__Bacteria;p__Spirochaetes;c__Spirochaetes;o__Spirochaetales;f__Spirochaetaceae;g__Treponema;s__	1.79E-02	2.82E-02	2.99E-01
287875	k__Bacteria;p__Firmicutes;c__Clostridia;o__Clostridiales;f__Ruminococcaceae;g__;s__	1.80E-02	1.41E-02	2.99E-01
New.CleanUp.ReferenceOTU94728	k__Bacteria;p__Firmicutes;c__Clostridia;o__Clostridiales;f__Ruminococcaceae;g__Ruminococcus;s__	1.80E-02	1.41E-02	2.99E-01
New.CleanUp.ReferenceOTU64797	k__Bacteria;p__Bacteroidetes;c__Bacteroidia;o__Bacteroidales;f__;g__;s__	1.80E-02	1.41E-02	4.63E-01
New.CleanUp.ReferenceOTU49075	k__Bacteria;p__Firmicutes;c__Clostridia;o__Clostridiales;f__Lachnospiraceae;g__;s__	1.80E-02	1.83E-01	5.67E-01
New.CleanUp.ReferenceOTU125119	k__Bacteria;p__Bacteroidetes;c__Bacteroidia;o__Bacteroidales;f__;g__;s__	1.82E-02	1.41E-02	2.84E-01
New.CleanUp.ReferenceOTU138962	k__Bacteria;p__Firmicutes;c__Clostridia;o__Clostridiales;f__Ruminococcaceae;g__;s__	1.82E-02	1.41E-02	2.54E-01
New.ReferenceOTU981	k__Bacteria;p__Bacteroidetes;c__Bacteroidia;o__Bacteroidales;f__;g__;s__	1.82E-02	1.41E-02	6.57E-01
270094	k__Bacteria;p__Bacteroidetes;c__Bacteroidia;o__Bacteroidales;f__Bacteroidaceae;g__Bacteroides;s__	1.82E-02	7.89E-01	2.24E-01
New.CleanUp.ReferenceOTU94005	k__Bacteria;p__Planctomycetes;c__Planctomycetia;o__Pirellulales;f__Pirellulaceae;g__;s__	1.83E-02	4.23E-02	6.27E-01
New.ReferenceOTU479	Unassigned	1.83E-02	1.41E-02	2.24E-01
New.CleanUp.ReferenceOTU67908	k__Bacteria;p__Firmicutes;c__Clostridia;o__Clostridiales;f__Ruminococcaceae;g__;s__	1.83E-02	1.41E-02	2.54E-01
New.CleanUp.ReferenceOTU92063	k__Bacteria;p__Tenericutes;c__Mollicutes;o__RF39;f__;g__;s__	1.83E-02	1.41E-02	2.39E-01

New.CleanUp.ReferenceOTU162801	k__Bacteria;p__Firmicutes;c__Clostridia;o__Clostridiales;f__Ruminococcaceae;g__;s__	1.83E-02	1.13E-01	5.67E-01
New.ReferenceOTU75	k__Bacteria;p__Firmicutes;c__Clostridia;o__Clostridiales;f__Christensenellaceae;g__;s__	1.84E-02	5.63E-02	3.88E-01
New.CleanUp.ReferenceOTU43261	k__Bacteria;p__Firmicutes;c__Clostridia;o__Clostridiales;f__Christensenellaceae;g__;s__	1.85E-02	3.80E-01	1.04E-01
New.CleanUp.ReferenceOTU47466	k__Bacteria;p__Bacteroidetes;c__Bacteroidia;o__Bacteroidales;f__;g__;s__	1.85E-02	1.41E-02	2.24E-01
289397	k__Bacteria;p__Firmicutes;c__Clostridia;o__Clostridiales;f__Lachnospiraceae;g__Blautia;s__	1.85E-02	1.68E+00	2.69E-01
New.CleanUp.ReferenceOTU89736	k__Bacteria;p__Bacteroidetes;c__Bacteroidia;o__Bacteroidales;f__[Paraprevotellaceae];g__CF231;s__	1.85E-02	3.38E-01	1.07E+00
New.CleanUp.ReferenceOTU102544	k__Bacteria;p__Firmicutes;c__Clostridia;o__Clostridiales;f__Lachnospiraceae;g__;s__	1.86E-02	2.82E-02	2.24E-01
4446238	k__Bacteria;p__Firmicutes;c__Clostridia;o__Clostridiales;f__Lachnospiraceae;g__;s__	1.86E-02	3.67E+01	1.83E+01
New.ReferenceOTU546	k__Bacteria;p__Firmicutes;c__Clostridia;o__Clostridiales;f__;g__;s__	1.87E-02	1.41E-02	1.94E-01
302690	k__Bacteria;p__Firmicutes;c__Clostridia;o__Clostridiales;f__Lachnospiraceae	1.89E-02	2.82E-02	3.73E-01
New.ReferenceOTU969	k__Bacteria;p__Firmicutes;c__Clostridia;o__Clostridiales;f__Lachnospiraceae;g__;s__	1.89E-02	1.69E-01	3.88E-01
298259	k__Bacteria;p__Verrucomicrobia;c__Verruco-5;o__WCHB1-41;f__RFP12;g__;s__	1.91E-02	7.04E-02	2.99E-01
340852	k__Bacteria;p__Firmicutes;c__Clostridia;o__Clostridiales;f__Lachnospiraceae;g__;s__	1.92E-02	5.92E-01	1.84E+00
New.CleanUp.ReferenceOTU16959	k__Bacteria;p__Firmicutes;c__Clostridia;o__Clostridiales;f__Ruminococcaceae;g__;s__	1.92E-02	1.27E-01	7.31E-01
New.CleanUp.ReferenceOTU144653	k__Bacteria;p__Firmicutes;c__Clostridia;o__Clostridiales;f__Lachnospiraceae;g__;s__	1.93E-02	3.80E-01	1.39E+00
New.CleanUp.ReferenceOTU60855	k__Bacteria;p__Firmicutes;c__Clostridia;o__Clostridiales;f__Christensenellaceae;g__;s__	1.93E-02	4.23E-02	3.58E-01
113542	k__Bacteria;p__Proteobacteria;c__Deltaproteobacteria;o__Desulfovibrionales;f__Desulfovibrionaceae;g__Desulfovibrio;s__	1.94E-02	3.85E+00	1.64E+00
303897	k__Bacteria;p__Firmicutes;c__Clostridia;o__Clostridiales;f__Lachnospiraceae;g__Blautia;s__	1.95E-02	1.55E-01	7.46E-01
New.CleanUp.ReferenceOTU160425	k__Bacteria;p__Bacteroidetes;c__Bacteroidia;o__Bacteroidales;f__[Paraprevotellaceae];g__;s__	1.95E-02	2.11E-01	1.19E+00
291527	k__Bacteria;p__Verrucomicrobia;c__Verruco-5;o__WCHB1-41;f__RFP12;g__;s__	1.96E-02	2.11E-01	8.66E-01
326486	k__Bacteria;p__Firmicutes;c__Clostridia;o__Clostridiales;f__Ruminococcaceae;g__;s__	1.98E-02	1.83E-01	8.06E-01
324857	k__Bacteria;p__Firmicutes;c__Clostridia;o__Clostridiales;f__[Mogibacteriaceae];g__;s__	1.99E-02	3.52E-01	9.25E-01
342716	k__Bacteria;p__Firmicutes;c__Clostridia;o__Clostridiales;f__Lachnospiraceae;g__;s__	1.99E-02	4.96E+00	7.37E+00
New.ReferenceOTU606	k__Bacteria;p__Firmicutes;c__Clostridia;o__Clostridiales;f__[Mogibacteriaceae];g__;s__	2.00E-02	4.23E-02	2.99E-01
New.CleanUp.ReferenceOTU63798	k__Bacteria;p__Bacteroidetes;c__Bacteroidia;o__Bacteroidales;f__;g__;s__	2.00E-02	3.38E-01	1.03E+00
562583	k__Bacteria;p__Firmicutes;c__Clostridia;o__Clostridiales;f__Ruminococcaceae;g__Ruminococcus;s__	2.01E-02	8.45E-02	1.06E+00
4373873	k__Bacteria;p__Firmicutes;c__Clostridia;o__Clostridiales;f__Lachnospiraceae;g__Dorea;s__	2.03E-02	3.52E-01	2.99E-02
317315	k__Bacteria;p__Firmicutes;c__Clostridia;o__Clostridiales;f__;g__;s__	2.04E-02	8.15E+00	1.19E+00
New.ReferenceOTU774	k__Bacteria;p__Bacteroidetes;c__Bacteroidia;o__Bacteroidales;f__Bacteroidaceae;g__Bacteroides;s__	2.05E-02	2.96E-01	1.49E-02
New.CleanUp.ReferenceOTU147585	k__Bacteria;p__Firmicutes;c__Clostridia;o__Clostridiales;f__;g__;s__	2.07E-02	5.63E-02	3.88E-01
302685	k__Bacteria;p__Firmicutes;c__Clostridia;o__Clostridiales;f__;g__;s__	2.07E-02	4.23E-02	4.18E-01
288651	k__Bacteria;p__Firmicutes;c__Clostridia;o__Clostridiales;f__Ruminococcaceae;g__;s__	2.07E-02	3.24E-01	1.49E-02

4338745	k__Bacteria;p__Firmicutes;c__Clostridia;o__Clostridiales;f__Veillonellaceae;g__;s__	2.07E-02	3.66E-01	1.49E-02
4295063	k__Bacteria;p__Firmicutes;c__Clostridia;o__Clostridiales;f__[Mogibacteriaceae];g__;s__	2.07E-02	4.08E-01	1.49E-02
533624	k__Bacteria;p__Firmicutes;c__Erysipelotrichi;o__Erysipelotrichales;f__Erysipelotrichaceae;g__Catenibacterium;s__	2.07E-02	7.32E-01	2.99E-02
182167	k__Bacteria;p__Firmicutes;c__Clostridia;o__Clostridiales;f__Ruminococcaceae;g__;s__	2.07E-02	7.46E-01	2.99E-02
589658	k__Bacteria;p__Firmicutes;c__Clostridia;o__Clostridiales;f__;g__;s__	2.07E-02	3.11E+00	2.24E-01
New.ReferenceOTU834	k__Bacteria;p__Firmicutes;c__Clostridia;o__Clostridiales;f__Ruminococcaceae;g__;s__	2.08E-02	1.15E+00	2.09E+00
346914	k__Bacteria;p__Bacteroidetes;c__Bacteroidia;o__Bacteroidales;f__;g__;s__	2.08E-02	3.24E-01	8.81E-01
New.ReferenceOTU97	k__Bacteria;p__Firmicutes;c__Clostridia;o__Clostridiales;f__Ruminococcaceae;g__;s__	2.08E-02	4.65E-01	1.49E-02
New.CleanUp.ReferenceOTU28336	k__Bacteria;p__Firmicutes;c__Clostridia;o__Clostridiales;f__Lachnospiraceae;g__Dorea;s__	2.08E-02	2.54E-01	1.49E-02
New.CleanUp.ReferenceOTU106366	k__Bacteria;p__Firmicutes;c__Clostridia;o__Clostridiales;f__Ruminococcaceae;g__;s__	2.08E-02	2.82E-01	1.49E-02
New.CleanUp.ReferenceOTU134024	k__Bacteria;p__Firmicutes;c__Clostridia;o__Clostridiales;f__Lachnospiraceae;g__;s__	2.08E-02	2.54E-01	1.49E-02
New.CleanUp.ReferenceOTU176836	k__Bacteria;p__Firmicutes;c__Clostridia;o__Clostridiales;f__Lachnospiraceae;g__Dorea;s__	2.08E-02	3.10E-01	1.49E-02
New.ReferenceOTU744	k__Bacteria;p__Firmicutes;c__Clostridia;o__Clostridiales;f__Lachnospiraceae;g__Dorea;s__	2.10E-02	2.39E-01	1.49E-02
New.CleanUp.ReferenceOTU58691	k__Bacteria;p__Firmicutes;c__Clostridia;o__Clostridiales;f__Lachnospiraceae;g__Blautia;s__producta	2.10E-02	2.39E-01	1.49E-02
213784	k__Bacteria;p__Firmicutes;c__Clostridia;o__Clostridiales;f__;g__;s__	2.10E-02	3.66E-01	1.49E-02
4311620	k__Bacteria;p__Firmicutes;c__Clostridia;o__Clostridiales;f__EtOH8;g__;s__	2.12E-02	8.87E-01	1.19E-01
302001	k__Bacteria;p__Firmicutes;c__Clostridia;o__Clostridiales;f__Veillonellaceae;g__Phascolarctobacterium;s__	2.12E-02	3.38E-01	1.37E+00
344639	k__Bacteria;p__Firmicutes;c__Clostridia;o__Clostridiales;f__;g__;s__	2.12E-02	1.18E+00	1.19E-01
30587	k__Bacteria;p__Firmicutes;c__Clostridia;o__Clostridiales;f__;g__;s__	2.12E-02	2.25E-01	1.49E-02
179572	k__Bacteria;p__Firmicutes;c__Clostridia;o__Clostridiales;f__Ruminococcaceae;g__Ruminococcus;s__	2.12E-02	2.39E-01	1.49E-02
106335	k__Bacteria;p__Firmicutes;c__Clostridia;o__Clostridiales;f__Dehalobacteriaceae;g__;s__	2.12E-02	1.45E+01	1.78E+01
185597	k__Bacteria;p__Firmicutes;c__Clostridia;o__Clostridiales;f__Ruminococcaceae;g__;s__	2.13E-02	5.63E-01	1.19E-01
4438090	k__Bacteria;p__Firmicutes;c__Clostridia;o__Clostridiales;f__Lachnospiraceae	2.14E-02	2.96E-01	2.99E-02
New.CleanUp.ReferenceOTU30930	k__Bacteria;p__Firmicutes;c__Clostridia;o__Clostridiales;f__;g__;s__	2.15E-02	1.83E-01	9.70E-01
New.CleanUp.ReferenceOTU170209	k__Bacteria;p__Firmicutes;c__Clostridia;o__Clostridiales;f__Ruminococcaceae;g__;s__	2.15E-02	1.27E-01	4.78E-01
New.ReferenceOTU491	k__Bacteria;p__Firmicutes;c__Clostridia;o__Clostridiales;f__Ruminococcaceae;g__;s__	2.16E-02	1.97E-01	6.72E-01
New.CleanUp.ReferenceOTU35651	k__Bacteria;p__Firmicutes;c__Bacilli;o__Lactobacillales;f__Lactobacillaceae;g__Lactobacillus;s__	2.19E-02	2.68E-01	2.99E-02
292779	k__Bacteria;p__Bacteroidetes;c__Bacteroidia;o__Bacteroidales;f__[Paraprevotellaceae];g__[Prevotella];s__	2.19E-02	2.25E-01	2.49E+00
784040	k__Bacteria;p__Firmicutes;c__Clostridia;o__Clostridiales;f__;g__;s__	2.19E-02	7.32E-01	2.63E+00
539506	k__Bacteria;p__Firmicutes;c__Clostridia;o__Clostridiales;f__Ruminococcaceae;g__;s__	2.19E-02	1.97E-01	4.03E-01
4354103	k__Bacteria;p__Firmicutes;c__Bacilli;o__Lactobacillales;f__Lactobacillaceae;g__Lactobacillus;s__	2.19E-02	1.43E+01	7.30E+00
842598	k__Archaea;p__Euryarchaeota;c__Methanobacteria;o__Methanobacteriales;f__Methanobacteriaceae;g__Methanobrevibacter;s__	2.21E-02	3.37E+01	8.16E+01

New.CleanUp.ReferenceOTU30250	k__Bacteria;p__Firmicutes;c__Clostridia;o__Clostridiales;f__Lachnospiraceae	2.22E-02	1.55E-01	4.48E-01
New.CleanUp.ReferenceOTU179076	k__Bacteria;p__Firmicutes;c__Clostridia;o__Clostridiales;f__Ruminococcaceae;g__;s__	2.22E-02	7.04E-02	4.33E-01
New.CleanUp.ReferenceOTU119842	k__Bacteria;p__Firmicutes;c__Clostridia;o__Clostridiales;f__Lachnospiraceae;g__[Ruminococcus];s__gnavus	2.25E-02	6.20E-01	8.96E-02
New.ReferenceOTU482	k__Bacteria;p__Spirochaetes;c__Spirochaetes;o__Spirochaetales;f__Spirochaetaceae;g__Treponema;s__	2.27E-02	7.18E-01	2.72E+00
New.CleanUp.ReferenceOTU119509	k__Bacteria;p__Firmicutes;c__Clostridia;o__Clostridiales;f__Lachnospiraceae;g__;s__	2.28E-02	6.90E-01	1.72E+00
196905	k__Bacteria;p__Firmicutes;c__Clostridia;o__Clostridiales;f__Ruminococcaceae;g__;s__	2.29E-02	2.58E+00	6.12E-01
New.ReferenceOTU952	k__Bacteria;p__Firmicutes;c__Bacilli;o__Lactobacillales;f__Lactobacillaceae;g__Lactobacillus;s__	2.29E-02	3.80E-01	5.97E-02
105411	k__Bacteria;p__Firmicutes;c__Clostridia;o__Clostridiales;f__Lachnospiraceae;g__Dorea;s__	2.29E-02	5.39E+00	5.34E+00
New.CleanUp.ReferenceOTU123174	k__Bacteria;p__Firmicutes;c__Clostridia;o__Clostridiales;f__Ruminococcaceae;g__;s__	2.29E-02	5.63E-02	2.84E-01
305924	k__Bacteria;p__Firmicutes;c__Clostridia;o__Clostridiales;f__Lachnospiraceae;g__;s__	2.30E-02	2.68E-01	7.61E-01
New.CleanUp.ReferenceOTU127332	Unassigned	2.30E-02	8.45E-02	4.33E-01
194670	k__Bacteria;p__Bacteroidetes;c__Bacteroidia;o__Bacteroidales;f__Bacteroidaceae;g__Bacteroides;s__	2.30E-02	3.66E-01	2.99E-02
New.ReferenceOTU93	k__Bacteria;p__Firmicutes;c__Clostridia;o__Clostridiales;f__Lachnospiraceae	2.30E-02	4.23E-01	1.03E+00
593686	k__Bacteria;p__Firmicutes;c__Clostridia;o__Clostridiales;f__Lachnospiraceae;g__Coprococcus;s__	2.31E-02	1.55E-01	4.93E-01
176980	k__Bacteria;p__Firmicutes;c__Clostridia;o__Clostridiales;f__Lachnospiraceae;g__Dorea;s__	2.31E-02	6.76E-01	1.49E-01
4468384	k__Bacteria;p__Firmicutes;c__Clostridia;o__Clostridiales;f__Lachnospiraceae;g__Blautia;s__	2.32E-02	4.79E-01	5.97E-02
New.CleanUp.ReferenceOTU146423	k__Bacteria;p__Firmicutes;c__Clostridia;o__Clostridiales;f__Clostridiaceae;g__Clostridium;s__	2.35E-02	5.92E-01	6.72E-01
341885	k__Bacteria;p__Firmicutes;c__Clostridia;o__Clostridiales;f__Christensenellaceae;g__;s__	2.35E-02	3.80E-01	1.19E-01
New.CleanUp.ReferenceOTU146550	k__Bacteria;p__Firmicutes;c__Clostridia;o__Clostridiales;f__Ruminococcaceae	2.35E-02	2.96E-01	2.99E-02
147678	k__Bacteria;p__Firmicutes;c__Clostridia;o__Clostridiales;f__;g__;s__	2.35E-02	1.37E+00	7.46E-02
4383953	k__Bacteria;p__Firmicutes;c__Clostridia;o__Clostridiales;f__Clostridiaceae;g__;s__	2.35E-02	1.69E-01	8.94E+00
653954	k__Bacteria;p__Firmicutes;c__Clostridia;o__Clostridiales;f__Lachnospiraceae;g__Dorea;s__	2.36E-02	8.31E-01	2.31E+00
302943	k__Bacteria;p__Firmicutes;c__Clostridia;o__Clostridiales;f__;g__;s__	2.39E-02	1.83E-01	8.66E-01
3152469	k__Bacteria;p__Firmicutes;c__Clostridia;o__Clostridiales	2.41E-02	2.31E+00	1.07E+00
4354042	k__Bacteria;p__Bacteroidetes;c__Bacteroidia;o__Bacteroidales;f__Bacteroidaceae;g__Bacteroides;s__	2.43E-02	4.65E-01	5.97E-02
New.ReferenceOTU747	k__Bacteria;p__Verrucomicrobia;c__Verruco-5;o__WCHB1-41;f__RFP12;g__;s__	2.44E-02	8.45E-02	9.70E-01
4381553	k__Bacteria;p__Bacteroidetes;c__Bacteroidia;o__Bacteroidales;f__Bacteroidaceae;g__Bacteroides;s__	2.45E-02	1.11E+00	1.94E-01
New.CleanUp.ReferenceOTU48497	k__Bacteria;p__Firmicutes;c__Clostridia;o__Clostridiales;f__Ruminococcaceae;g__;s__	2.47E-02	4.79E-01	8.21E-01
316144	k__Bacteria;p__Firmicutes;c__Clostridia;o__Clostridiales;f__Lachnospiraceae;g__Blautia;s__	2.47E-02	4.23E-02	4.48E-01
New.CleanUp.ReferenceOTU12089	k__Bacteria;p__Firmicutes;c__Clostridia;o__Clostridiales;f__;g__;s__	2.47E-02	1.55E-01	6.27E-01
263036	k__Bacteria;p__Firmicutes;c__Clostridia;o__Clostridiales;f__Clostridiaceae;g__Clostridium;s__	2.49E-02	3.24E-01	2.15E+00
350337	k__Bacteria;p__Firmicutes;c__Clostridia;o__Clostridiales;f__;g__;s__	2.49E-02	7.04E-02	4.48E-01

161423	k__Bacteria;p__Bacteroidetes;c__Bacteroidia;o__Bacteroidales;f__Bacteroidaceae;g__Bacteroides;s__	2.51E-02	1.52E+00	5.52E-01
231510	k__Bacteria;p__Actinobacteria;c__Coriobacteriia;o__Coriobacteriales;f__Coriobacteriaceae;g__Adlercreutzia;s__	2.55E-02	2.25E-01	6.12E-01
New.CleanUp.ReferenceOTU107484	k__Bacteria;p__Spirochaetes;c__Spirochaetes;o__Spirochaetales;f__Spirochaetaceae;g__Treponema;s__	2.55E-02	4.23E-02	3.43E-01
578754	k__Bacteria;p__Firmicutes;c__Clostridia;o__Clostridiales;f__;g__;s__	2.55E-02	2.11E-01	8.96E-02
New.CleanUp.ReferenceOTU50397	k__Bacteria;p__Firmicutes;c__Clostridia;o__Clostridiales;f__;g__;s__	2.57E-02	3.10E-01	1.00E+00
178996	k__Bacteria;p__Firmicutes;c__Clostridia;o__Clostridiales;f__;g__;s__	2.58E-02	8.45E-02	6.42E-01
4473664	k__Bacteria;p__Firmicutes;c__Clostridia;o__Clostridiales;f__Peptostreptococcaceae;g__Peptostreptococcus;s__anaerobius	2.66E-02	8.45E-02	1.18E+00
299673	k__Bacteria;p__Firmicutes;c__Clostridia;o__Clostridiales;f__;g__;s__	2.67E-02	1.48E+02	5.21E+01
4387250	k__Bacteria;p__Bacteroidetes;c__Bacteroidia;o__Bacteroidales;f__Bacteroidaceae;g__Bacteroides;s__	2.67E-02	6.06E-01	2.39E-01
New.ReferenceOTU21	k__Bacteria;p__Verrucomicrobia;c__Verrucomicrobiae;o__Verrucomicrobiales;f__Verrucomicrobiaceae;g__Akkermansia;s__muciniphila	2.69E-02	1.75E+00	3.88E-01
301584	k__Bacteria;p__Fibrobacteres;c__Fibrobacteria;o__Fibrobacterales;f__Fibrobacteraceae;g__Fibrobacter;s__succinogenes	2.69E-02	9.86E-02	8.51E-01
303666	k__Bacteria;p__Firmicutes;c__Clostridia;o__Clostridiales;f__Ruminococcaceae;g__;s__	2.72E-02	0.00E+00	1.34E-01
New.CleanUp.ReferenceOTU166823	k__Bacteria;p__Firmicutes;c__Clostridia;o__Clostridiales;f__Lachnospiraceae;g__;s__	2.72E-02	0.00E+00	1.34E-01
366376	k__Bacteria;p__Firmicutes;c__Clostridia;o__Clostridiales;f__Ruminococcaceae;g__;s__	2.72E-02	5.21E-01	1.34E-01
New.ReferenceOTU976	k__Bacteria;p__Tenericutes;c__RF3;o__ML615J-28;f__;g__;s__	2.72E-02	0.00E+00	2.39E-01
New.CleanUp.ReferenceOTU5619	k__Bacteria;p__Bacteroidetes;c__Bacteroidia;o__Bacteroidales;f__;g__;s__	2.72E-02	0.00E+00	1.49E-01
New.CleanUp.ReferenceOTU11026	k__Bacteria;p__Verrucomicrobia;c__Verrucomicrobiae;o__Verrucomicrobiales;f__Verrucomicrobiaceae;g__Akkermansia;s__	2.72E-02	0.00E+00	1.49E-01
New.CleanUp.ReferenceOTU132897	k__Bacteria;p__Firmicutes;c__Clostridia;o__Clostridiales;f__Lachnospiraceae;g__;s__	2.72E-02	0.00E+00	1.64E-01
New.ReferenceOTU62	k__Bacteria;p__Firmicutes;c__Clostridia;o__Clostridiales;f__Lachnospiraceae;g__;s__	2.72E-02	0.00E+00	1.64E-01
New.CleanUp.ReferenceOTU112927	k__Bacteria;p__Fibrobacteres;c__Fibrobacteria;o__Fibrobacterales;f__Fibrobacteraceae;g__Fibrobacter;s__succinogenes	2.72E-02	0.00E+00	2.09E-01
New.CleanUp.ReferenceOTU54532	k__Bacteria;p__Firmicutes;c__Clostridia;o__Clostridiales;f__;g__;s__	2.72E-02	1.55E-01	3.28E-01
180881	k__Bacteria;p__Firmicutes;c__Clostridia;o__Clostridiales;f__;g__;s__	2.72E-02	0.00E+00	2.69E-01
New.CleanUp.ReferenceOTU39344	Unassigned	2.72E-02	0.00E+00	2.24E-01
213671	k__Bacteria;p__Bacteroidetes;c__Bacteroidia;o__Bacteroidales;f__Rikenellaceae;g__;s__	2.72E-02	1.00E+00	7.46E-02
New.CleanUp.ReferenceOTU151697	k__Bacteria;p__Spirochaetes;c__Spirochaetes;o__Spirochaetales;f__Spirochaetaceae;g__Treponema;s__	2.72E-02	0.00E+00	2.09E-01
296649	k__Bacteria;p__Firmicutes;c__Clostridia;o__Clostridiales;f__Ruminococcaceae;g__;s__	2.72E-02	0.00E+00	2.09E-01
New.CleanUp.ReferenceOTU111775	k__Bacteria;p__Firmicutes;c__Clostridia;o__Clostridiales;f__;g__;s__	2.72E-02	0.00E+00	2.84E-01
New.CleanUp.ReferenceOTU108477	k__Bacteria;p__Spirochaetes;c__Spirochaetes;o__Spirochaetales;f__Spirochaetaceae;g__Treponema;s__	2.72E-02	0.00E+00	5.37E-01
193364	k__Bacteria;p__Firmicutes;c__Clostridia;o__Clostridiales;f__Lachnospiraceae;g__Dorea;s__	2.73E-02	1.55E-01	0.00E+00
215231	k__Bacteria;p__Firmicutes;c__Clostridia;o__Clostridiales;f__Ruminococcaceae;g__;s__	2.73E-02	1.69E-01	0.00E+00
189860	k__Bacteria;p__Firmicutes;c__Clostridia;o__Clostridiales;f__Lachnospiraceae;g__Roseburia;s__	2.73E-02	7.46E-01	0.00E+00
294169	k__Bacteria;p__Firmicutes;c__Clostridia;o__Clostridiales;f__Ruminococcaceae;g__;s__	2.73E-02	1.69E-01	0.00E+00

574038	k__Bacteria;p__Firmicutes;c__Clostridia;o__Clostridiales;f__;g__;s__	2.73E-02	1.69E-01	0.00E+00
New.CleanUp.ReferenceOTU15496	k__Bacteria;p__Proteobacteria;c__Epsilonproteobacteria;o__Campylobacterales;f__Campylobacteraceae;g__Campylobacter;s__	2.73E-02	1.69E-01	0.00E+00
291045	k__Bacteria;p__Firmicutes;c__Clostridia;o__Clostridiales;f__Ruminococcaceae;g__;s__	2.73E-02	5.21E-01	0.00E+00
New.CleanUp.ReferenceOTU105519	k__Bacteria;p__Firmicutes;c__Clostridia;o__Clostridiales	2.73E-02	1.97E-01	0.00E+00
181291	k__Bacteria;p__Firmicutes;c__Clostridia;o__Clostridiales;f__Lachnospiraceae	2.73E-02	1.83E-01	0.00E+00
1105211	k__Bacteria;p__Firmicutes;c__Clostridia;o__Clostridiales;f__Lachnospiraceae	2.73E-02	2.11E-01	0.00E+00
New.CleanUp.ReferenceOTU115020	k__Bacteria;p__Firmicutes;c__Erysipelotrichi;o__Erysipelotrichales;f__Erysipelotrichaceae;g__;s__	2.73E-02	2.11E-01	0.00E+00
355424	k__Bacteria;p__Firmicutes;c__Clostridia;o__Clostridiales;f__Lachnospiraceae;g__;s__	2.73E-02	2.11E-01	0.00E+00
New.CleanUp.ReferenceOTU75959	k__Bacteria;p__Firmicutes;c__Clostridia;o__Clostridiales;f__Ruminococcaceae;g__Ruminococcus;s__	2.73E-02	3.52E-01	0.00E+00
New.CleanUp.ReferenceOTU112122	k__Bacteria;p__Firmicutes;c__Clostridia;o__Clostridiales;f__Lachnospiraceae;g__Blautia;s__	2.73E-02	2.11E-01	0.00E+00
4021516	k__Bacteria;p__Firmicutes;c__Clostridia;o__Clostridiales;f__Peptococcaceae;g__;s__	2.73E-02	3.10E-01	0.00E+00
New.CleanUp.ReferenceOTU14221	k__Bacteria;p__Firmicutes;c__Clostridia;o__Clostridiales;f__;g__;s__	2.73E-02	2.68E-01	0.00E+00
4427459	k__Bacteria;p__Firmicutes;c__Clostridia;o__Clostridiales;f__Ruminococcaceae;g__Ruminococcus;s__	2.73E-02	6.20E-01	0.00E+00
New.CleanUp.ReferenceOTU133213	k__Bacteria;p__Firmicutes;c__Clostridia;o__Clostridiales;f__Lachnospiraceae;g__;s__	2.73E-02	6.48E-01	0.00E+00
New.CleanUp.ReferenceOTU105326	k__Bacteria;p__Firmicutes;c__Clostridia;o__Clostridiales;f__Lachnospiraceae;g__Blautia;s__	2.73E-02	2.68E-01	0.00E+00
182656	k__Bacteria;p__Firmicutes;c__Clostridia;o__Clostridiales;f__;g__;s__	2.73E-02	3.10E-01	0.00E+00
New.CleanUp.ReferenceOTU63362	k__Bacteria;p__Firmicutes;c__Clostridia;o__Clostridiales	2.73E-02	4.37E-01	0.00E+00
136518	k__Bacteria;p__Firmicutes;c__Clostridia;o__Clostridiales;f__Lachnospiraceae;g__Epulopiscium;s__	2.73E-02	5.07E-01	0.00E+00
New.ReferenceOTU54	k__Bacteria;p__Bacteroidetes;c__Bacteroidia;o__Bacteroidales;f__;g__;s__	2.73E-02	2.82E-02	8.21E-01
331156	k__Bacteria;p__Firmicutes;c__Clostridia;o__Clostridiales;f__;g__;s__	2.73E-02	7.32E-01	8.81E-01
174443	k__Bacteria;p__Firmicutes;c__Clostridia;o__Clostridiales;f__Ruminococcaceae;g__;s__	2.75E-02	7.46E-01	1.19E-01
New.CleanUp.ReferenceOTU161235	Unassigned	2.78E-02	3.52E-01	1.22E+00
287506	k__Bacteria;p__Firmicutes;c__Clostridia;o__Clostridiales;f__;g__;s__	2.80E-02	2.82E-02	5.52E-01
813944	k__Bacteria;p__Firmicutes;c__Bacilli;o__Lactobacillales;f__Lactobacillaceae;g__Lactobacillus;s__	2.80E-02	1.34E+00	3.88E-01
356061	k__Bacteria;p__Firmicutes;c__Clostridia;o__Clostridiales;f__Lachnospiraceae;g__;s__	2.81E-02	5.63E-02	7.01E-01
307204	k__Bacteria;p__Firmicutes;c__Clostridia;o__Clostridiales;f__Clostridiaceae;g__Clostridium;s__	2.82E-02	9.15E-01	1.58E+00
New.CleanUp.ReferenceOTU90354	k__Bacteria;p__Firmicutes;c__Clostridia;o__Clostridiales;f__Christensenellaceae;g__;s__	2.84E-02	5.63E-02	5.52E-01
New.CleanUp.ReferenceOTU45506	k__Bacteria;p__Spirochaetes;c__Spirochaetes;o__Spirochaetales;f__Spirochaetaceae;g__Treponema;s__	2.86E-02	2.82E-02	3.28E-01
355959	k__Bacteria;p__Firmicutes;c__Clostridia;o__Clostridiales;f__Ruminococcaceae;g__;s__	2.87E-02	8.45E-02	3.88E-01
324888	k__Bacteria;p__Firmicutes;c__Bacilli;o__Lactobacillales;f__Lactobacillaceae;g__Lactobacillus;s__	2.87E-02	6.90E-01	2.84E-01
302287	k__Bacteria;p__Firmicutes;c__Clostridia;o__Clostridiales;f__Ruminococcaceae;g__;s__	2.88E-02	1.83E-01	6.27E-01
New.ReferenceOTU483	k__Bacteria;p__Firmicutes;c__Clostridia;o__Clostridiales;f__;g__;s__	2.89E-02	5.49E-01	9.85E-01

4363552	k__Bacteria;p__Firmicutes;c__Clostridia;o__Clostridiales;f__Lachnospiraceae;g__;s__	2.90E-02	5.77E-01	7.46E-02
301587	k__Bacteria;p__Spirochaetes;c__Spirochaetes;o__Spirochaetales;f__Spirochaetaceae;g__Treponema;s__	2.93E-02	2.82E-02	3.73E-01
New.ReferenceOTU429	k__Bacteria;p__Bacteroidetes;c__Bacteroidia;o__Bacteroidales;f__;g__;s__	2.93E-02	8.45E-01	2.10E+00
302090	k__Bacteria;p__Firmicutes;c__Clostridia;o__Clostridiales;f__Lachnospiraceae;g__Blautia;s__	2.96E-02	8.45E-01	2.69E-01
New.CleanUp.ReferenceOTU11340	Unassigned	3.00E-02	2.82E-02	3.43E-01
New.CleanUp.ReferenceOTU165491	k__Bacteria;p__Firmicutes;c__Erysipelotrichi;o__Erysipelotrichales;f__Erysipelotrichaceae;g__p-75-a5;s__	3.00E-02	2.82E-01	7.76E-01
175751	k__Bacteria;p__Firmicutes;c__Clostridia;o__Clostridiales;f__Lachnospiraceae;g__;s__	3.00E-02	6.83E+00	1.64E+00
New.ReferenceOTU388	k__Bacteria;p__Bacteroidetes;c__Bacteroidia;o__Bacteroidales;f__S24-7;g__;s__	3.01E-02	1.41E-02	9.40E-01
4298137	k__Bacteria;p__Firmicutes;c__Clostridia;o__Clostridiales;f__Christensenellaceae;g__;s__	3.02E-02	8.87E-01	2.54E-01
15432	k__Bacteria;p__Firmicutes;c__Bacilli;o__Lactobacillales;f__Streptococcaceae;g__Streptococcus;s__	3.05E-02	1.41E-02	3.13E-01
New.CleanUp.ReferenceOTU30741	k__Bacteria;p__Firmicutes;c__Clostridia;o__Clostridiales;f__Ruminococcaceae;g__;s__	3.06E-02	2.82E-02	2.54E-01
New.CleanUp.ReferenceOTU102811	k__Bacteria;p__Firmicutes;c__Clostridia;o__Clostridiales;f__;g__;s__	3.08E-02	5.63E-02	3.13E-01
288682	k__Bacteria;p__Firmicutes;c__Clostridia;o__Clostridiales;f__Lachnospiraceae;g__Blautia;s__	3.08E-02	1.41E-02	3.28E-01
New.CleanUp.ReferenceOTU125847	k__Bacteria;p__Spirochaetes;c__MVP-15;o__PL-11B10;f__;g__;s__	3.08E-02	1.41E-02	4.48E-01
New.CleanUp.ReferenceOTU326	k__Bacteria;p__Bacteroidetes;c__Bacteroidia;o__Bacteroidales;f__[Paraprevotellaceae];g__YRC22;s__	3.08E-02	1.41E-02	4.18E-01
314743	k__Bacteria;p__Firmicutes;c__Clostridia;o__Clostridiales;f__Ruminococcaceae;g__Ruminococcus;s__	3.09E-02	5.63E-02	9.10E-01
4372612	k__Bacteria;p__Bacteroidetes;c__Bacteroidia;o__Bacteroidales;f__Bacteroidaceae;g__Bacteroides;s__	3.10E-02	1.34E+00	2.84E-01
180289	k__Bacteria;p__Firmicutes;c__Clostridia;o__Clostridiales;f__Lachnospiraceae;g__;s__	3.10E-02	5.21E-01	4.48E-02
New.CleanUp.ReferenceOTU67309	k__Bacteria;p__Firmicutes;c__Clostridia;o__Clostridiales;f__Ruminococcaceae;g__;s__	3.11E-02	5.63E-02	2.84E-01
New.CleanUp.ReferenceOTU156092	k__Bacteria;p__Firmicutes;c__Clostridia;o__Clostridiales;f__Dehalobacteriaceae;g__Dehalobacterium;s__	3.11E-02	5.63E-02	2.84E-01
349546	k__Bacteria;p__Firmicutes;c__Clostridia;o__Clostridiales;f__Lachnospiraceae;g__;s__	3.11E-02	1.27E-01	5.37E-01
New.CleanUp.ReferenceOTU80085	k__Bacteria;p__Spirochaetes;c__Spirochaetes;o__Spirochaetales;f__Spirochaetaceae;g__Treponema;s__	3.11E-02	1.41E-02	2.24E-01
568828	k__Bacteria;p__Firmicutes;c__Clostridia;o__Clostridiales;f__Lachnospiraceae;g__;s__	3.11E-02	2.82E-02	2.84E-01
328724	k__Bacteria;p__Firmicutes;c__Clostridia;o__Clostridiales;f__;g__;s__	3.11E-02	1.41E-02	4.18E-01
New.CleanUp.ReferenceOTU169319	k__Bacteria;p__Firmicutes;c__Clostridia;o__Clostridiales;f__Clostridiaceae;g__Clostridium;s__	3.11E-02	1.41E-02	3.88E-01
310662	k__Bacteria;p__Firmicutes;c__Clostridia;o__Clostridiales;f__;g__;s__	3.14E-02	1.41E-02	2.09E-01
329746	k__Bacteria;p__Firmicutes;c__Clostridia;o__Clostridiales;f__Lachnospiraceae;g__;s__	3.14E-02	1.41E-02	2.54E-01
New.CleanUp.ReferenceOTU171161	k__Bacteria;p__Firmicutes;c__Clostridia;o__Clostridiales;f__Ruminococcaceae;g__;s__	3.14E-02	1.41E-02	2.24E-01
New.CleanUp.ReferenceOTU173567	k__Bacteria;p__Firmicutes;c__Clostridia;o__Clostridiales;f__Ruminococcaceae;g__Ruminococcus;s__	3.14E-02	1.41E-02	2.24E-01
New.CleanUp.ReferenceOTU78356	k__Bacteria;p__Firmicutes;c__Clostridia;o__Clostridiales;f__Ruminococcaceae;g__;s__	3.14E-02	1.41E-02	3.13E-01
New.CleanUp.ReferenceOTU123963	k__Bacteria;p__Firmicutes;c__Clostridia;o__Clostridiales;f__Ruminococcaceae;g__Oscillospira;s__	3.17E-02	1.41E-02	1.94E-01
291248	k__Bacteria;p__Firmicutes;c__Clostridia;o__Clostridiales;f__Ruminococcaceae;g__;s__	3.17E-02	1.41E-02	2.24E-01

177230	k__Bacteria;p__Firmicutes;c__Clostridia;o__Clostridiales;f__Lachnospiraceae;g__;s__	3.18E-02	4.23E-01	4.48E-02
291360	k__Bacteria;p__Firmicutes;c__Clostridia;o__Clostridiales;f__Ruminococcaceae;g__Ruminococcus;s__	3.21E-02	4.23E-02	7.91E-01
New.CleanUp.ReferenceOTU27001	k__Bacteria;p__Firmicutes;c__Clostridia;o__Clostridiales;f__Lachnospiraceae;g__Moryella;s__	3.22E-02	3.10E-01	6.12E-01
296790	k__Bacteria;p__Firmicutes;c__Clostridia;o__Clostridiales;f__Lachnospiraceae;g__;s__	3.22E-02	5.63E-02	2.54E-01
New.CleanUp.ReferenceOTU29715	k__Bacteria;p__Firmicutes;c__Clostridia;o__Clostridiales;f__Ruminococcaceae;g__;s__	3.22E-02	5.63E-02	2.54E-01
New.CleanUp.ReferenceOTU157482	k__Bacteria;p__Firmicutes;c__Clostridia;o__Clostridiales;f__Ruminococcaceae;g__Oscillospira;s__	3.24E-02	1.14E+00	3.73E-01
New.ReferenceOTU967	k__Bacteria;p__Firmicutes;c__Clostridia;o__Clostridiales;f__Ruminococcaceae;g__;s__	3.25E-02	1.93E+00	3.63E+00
New.CleanUp.ReferenceOTU169024	k__Bacteria;p__Firmicutes;c__Clostridia;o__Clostridiales;f__Ruminococcaceae;g__;s__	3.25E-02	3.94E-01	1.49E-02
2774254	k__Bacteria;p__Firmicutes;c__Clostridia;o__Clostridiales;f__Lachnospiraceae;g__;s__	3.26E-02	1.39E+00	3.13E-01
294791	k__Bacteria;p__Firmicutes;c__Clostridia;o__Clostridiales;f__Lachnospiraceae;g__Coprococcus;s__	3.29E-02	1.42E+00	1.49E-02
270448	k__Bacteria;p__Firmicutes;c__Clostridia;o__Clostridiales	3.29E-02	4.93E-01	1.49E-02
15431	k__Bacteria;p__Firmicutes;c__Bacilli;o__Lactobacillales;f__Streptococcaceae;g__Streptococcus;s__	3.29E-02	4.08E-01	7.46E-02
New.CleanUp.ReferenceOTU110064	k__Bacteria;p__Firmicutes;c__Clostridia;o__Clostridiales;f__Ruminococcaceae;g__;s__	3.29E-02	7.04E-02	2.54E-01
342110	k__Bacteria;p__Firmicutes;c__Clostridia;o__Clostridiales;f__Lachnospiraceae;g__Dorea;s__	3.29E-02	8.45E-01	7.46E-02
New.CleanUp.ReferenceOTU66811	k__Bacteria;p__Bacteroidetes;c__Bacteroidia;o__Bacteroidales;f__;g__;s__	3.29E-02	2.11E-01	8.51E-01
New.ReferenceOTU358	k__Bacteria;p__Verrucomicrobia;c__Verruco-5;o__WCHB1-41;f__RFP12;g__;s__	3.31E-02	4.23E-02	2.99E-01
New.ReferenceOTU983	k__Bacteria;p__Firmicutes;c__Clostridia;o__Clostridiales;f__Lachnospiraceae;g__Coprococcus;s__	3.32E-02	3.24E-01	1.49E-02
New.CleanUp.ReferenceOTU11071	k__Bacteria;p__Verrucomicrobia;c__Verrucomicrobiae;o__Verrucomicrobiales;f__Verrucomicrobiaceae;g__Akkermansia;s__	3.32E-02	1.13E-01	4.93E-01
New.ReferenceOTU78	k__Bacteria;p__Verrucomicrobia;c__Verrucomicrobiae;o__Verrucomicrobiales;f__Verrucomicrobiaceae;g__Akkermansia;s__	3.33E-02	1.27E-01	5.22E-01
287924	k__Bacteria;p__Firmicutes;c__Clostridia;o__Clostridiales;f__Lachnospiraceae;g__;s__	3.34E-02	7.46E-01	8.96E-02
26608	k__Bacteria;p__Firmicutes;c__Bacilli;o__Lactobacillales;f__Lactobacillaceae;g__Lactobacillus;s__	3.34E-02	4.79E-01	7.46E-02
4470260	k__Bacteria;p__Firmicutes;c__Bacilli;o__Bacillales;f__Bacillaceae;g__Bacillus;s__	3.35E-02	3.24E-01	1.49E-02
193709	k__Bacteria;p__Firmicutes;c__Clostridia;o__Clostridiales;f__Ruminococcaceae;g__;s__	3.35E-02	3.52E-01	4.48E-02
4308793	k__Bacteria;p__Fusobacteria;c__Fusobacteriia;o__Fusobacteriales;f__Fusobacteriaceae;g__Fusobacterium;s__	3.37E-02	5.92E-01	2.15E+00
73371	k__Bacteria;p__Firmicutes;c__Clostridia;o__Clostridiales;f__Lachnospiraceae;g__;s__	3.37E-02	1.28E+00	2.63E+00
3528445	k__Archaea;p__Euryarchaeota;c__Methanobacteria;o__Methanobacteriales;f__Methanobacteriaceae;g__Methanosphaera;s__	3.38E-02	9.30E-01	1.96E+00
526963	k__Bacteria;p__Firmicutes;c__Clostridia;o__Clostridiales;f__;g__;s__	3.40E-02	3.66E-01	1.00E+00
291823	k__Bacteria;p__Firmicutes;c__Clostridia;o__Clostridiales;f__Lachnospiraceae;g__;s__	3.40E-02	4.37E-01	1.57E+00
New.ReferenceOTU801	k__Bacteria;p__Firmicutes;c__Clostridia;o__Clostridiales;f__Ruminococcaceae;g__;s__	3.40E-02	4.08E-01	2.99E-02
190772	k__Bacteria;p__Firmicutes;c__Clostridia;o__Clostridiales;f__Lachnospiraceae;g__Dorea;s__	3.42E-02	2.11E-01	1.49E-02
564400	k__Bacteria;p__Firmicutes;c__Clostridia;o__Clostridiales	3.42E-02	2.11E-01	1.49E-02
New.CleanUp.ReferenceOTU7639	k__Bacteria;p__Spirochaetes;c__Spirochaetes;o__Spirochaetales;f__Spirochaetaceae;g__Treponema;s__	3.43E-02	8.45E-02	3.13E-01

339336	k__Bacteria;p__Firmicutes;c__Clostridia;o__Clostridiales;f__;g__;s__	3.44E-02	2.14E+00	4.67E+00
New.CleanUp.ReferenceOTU180893	k__Bacteria;p__Firmicutes;c__Clostridia;o__Clostridiales;f__;g__;s__	3.44E-02	2.96E-01	4.48E-02
4415390	k__Bacteria;p__Firmicutes;c__Clostridia;o__Clostridiales;f__Lachnospiraceae;g__;s__	3.45E-02	1.00E+00	1.49E-02
New.CleanUp.ReferenceOTU21081	k__Bacteria;p__Bacteroidetes;c__Bacteroidia;o__Bacteroidales;f__Porphyromonadaceae;g__Parabacteroides;s__	3.45E-02	2.68E-01	1.49E-02
4467447	k__Bacteria;p__Bacteroidetes;c__Bacteroidia;o__Bacteroidales;f__Bacteroidaceae;g__Bacteroides;s__	3.47E-02	4.51E-01	2.99E-02
New.CleanUp.ReferenceOTU67812	k__Bacteria;p__Firmicutes;c__Clostridia;o__Clostridiales;f__;g__;s__	3.47E-02	3.80E-01	2.99E-02
297180	k__Bacteria;p__Firmicutes;c__Clostridia;o__Clostridiales;f__;g__;s__	3.48E-02	1.69E-01	5.67E-01
New.CleanUp.ReferenceOTU43007	k__Bacteria;p__Firmicutes;c__Clostridia;o__Clostridiales;f__Lachnospiraceae;g__Blautia;s__	3.48E-02	1.83E-01	1.49E-02
New.CleanUp.ReferenceOTU94576	Unassigned	3.48E-02	2.11E-01	1.49E-02
184864	k__Bacteria;p__Firmicutes;c__Clostridia;o__Clostridiales;f__;g__;s__	3.49E-02	3.18E+00	6.57E-01
4412540	k__Bacteria;p__Firmicutes;c__Clostridia;o__Clostridiales;f__Ruminococcaceae;g__;s__	3.50E-02	1.80E+00	1.34E-01
New.CleanUp.ReferenceOTU35016	k__Bacteria;p__Firmicutes;c__Clostridia;o__Clostridiales;f__Lachnospiraceae;g__Dorea;s__	3.51E-02	1.69E-01	1.49E-02
340711	k__Bacteria;p__Firmicutes;c__Clostridia;o__Clostridiales;f__Ruminococcaceae;g__;s__	3.53E-02	2.96E-01	2.99E-02
329402	k__Bacteria;p__Firmicutes;c__Bacilli;o__Lactobacillales;f__Lactobacillaceae;g__Lactobacillus;s__reuteri	3.53E-02	2.68E-01	2.99E-02
New.CleanUp.ReferenceOTU55926	k__Bacteria;p__Firmicutes;c__Clostridia;o__Clostridiales;f__Lachnospiraceae;g__Dorea;s__	3.53E-02	2.68E-01	2.99E-02
322049	k__Bacteria;p__Bacteroidetes;c__Bacteroidia;o__Bacteroidales;f__Prevotellaceae;g__Prevotella;s__	3.55E-02	5.63E-02	1.04E+00
522138	k__Bacteria;p__Firmicutes;c__Clostridia;o__Clostridiales;f__;g__;s__	3.56E-02	7.04E-02	2.84E-01
294709	k__Bacteria;p__Firmicutes;c__Clostridia;o__Clostridiales;f__Lachnospiraceae;g__Coprococcus;s__	3.58E-02	1.69E-01	6.57E-01
New.ReferenceOTU692	k__Bacteria;p__Firmicutes;c__Clostridia;o__Clostridiales;f__;g__;s__	3.58E-02	2.96E-01	9.40E-01
175922	k__Bacteria;p__Firmicutes;c__Clostridia;o__Clostridiales;f__Ruminococcaceae;g__Oscillospira;s__	3.60E-02	2.96E-01	2.99E-02
New.CleanUp.ReferenceOTU124018	k__Bacteria;p__Firmicutes;c__Clostridia;o__Clostridiales;f__Veillonellaceae;g__Anaerovibrio;s__	3.60E-02	1.55E+00	2.99E-02
560491	k__Bacteria;p__Firmicutes;c__Clostridia;o__Clostridiales;f__Ruminococcaceae;g__;s__	3.61E-02	2.87E+00	7.01E-01
174288	k__Bacteria;p__Firmicutes;c__Clostridia;o__Clostridiales	3.62E-02	2.39E-01	4.48E-02
New.ReferenceOTU218	k__Bacteria;p__Firmicutes;c__Clostridia;o__Clostridiales;f__;g__;s__	3.64E-02	6.20E-01	1.13E+00
4460786	k__Bacteria;p__Firmicutes;c__Clostridia;o__Clostridiales;f__;g__;s__	3.69E-02	4.23E-01	2.99E-02
3359884	k__Bacteria;p__Cyanobacteria;c__Chloroplast;o__Streptophyta;f__;g__;s__	3.70E-02	9.86E-02	3.13E-01
New.ReferenceOTU333	k__Bacteria;p__Bacteroidetes;c__Bacteroidia;o__Bacteroidales;f__;g__;s__	3.71E-02	5.17E+00	6.82E+00
New.CleanUp.ReferenceOTU83921	k__Bacteria;p__Firmicutes;c__Clostridia;o__Clostridiales;f__Ruminococcaceae;g__;s__	3.71E-02	4.23E-02	1.79E-01
New.CleanUp.ReferenceOTU29299	k__Bacteria;p__Verrucomicrobia;c__Verruco-5;o__WCHB1-41;f__RFP12;g__;s__	3.79E-02	7.04E-02	4.63E-01
New.ReferenceOTU214	k__Bacteria;p__Firmicutes;c__Clostridia;o__Clostridiales;f__Lachnospiraceae;g__Coprococcus;s__	3.79E-02	1.44E+01	1.14E+01
1043378	k__Bacteria;p__Firmicutes;c__Clostridia;o__Clostridiales;f__Ruminococcaceae;g__;s__	3.80E-02	5.45E+01	3.57E+01
New.CleanUp.ReferenceOTU85878	k__Bacteria;p__Firmicutes;c__Clostridia;o__Clostridiales;f__;g__;s__	3.81E-02	1.13E-01	6.27E-01

292802	k__Bacteria;p__Firmicutes;c__Clostridia;o__Clostridiales;f__Ruminococcaceae;g__;s__	3.90E-02	8.45E-02	4.78E-01
181871	k__Bacteria;p__Firmicutes;c__Clostridia;o__Clostridiales;f__Lachnospiraceae;g__Dorea;s__	3.93E-02	3.10E-01	5.97E-02
New.ReferenceOTU202	k__Bacteria;p__Bacteroidetes;c__Bacteroidia;o__Bacteroidales;f__[Paraprevotellaceae];g__[Prevotella];s__	3.95E-02	1.41E-01	2.34E+00
334797	k__Bacteria;p__Firmicutes;c__Clostridia;o__Clostridiales;f__Ruminococcaceae;g__Oscillospira;s__	3.96E-02	6.62E-01	2.39E-01
2835813	k__Bacteria;p__Firmicutes;c__Clostridia;o__Clostridiales;f__Ruminococcaceae;g__;s__	3.96E-02	5.35E-01	8.96E-02
510094	k__Bacteria;p__Firmicutes;c__Clostridia;o__Clostridiales;f__[Mogibacteriaceae];g__Mogibacterium;s__	3.96E-02	9.86E-02	4.18E-01
3841096	k__Bacteria;p__Firmicutes;c__Clostridia;o__Clostridiales;f__Ruminococcaceae;g__Oscillospira;s__	3.96E-02	4.72E+00	3.58E-01
580305	k__Bacteria;p__Firmicutes;c__Clostridia;o__Clostridiales;f__Ruminococcaceae;g__;s__	3.96E-02	8.31E-01	1.94E-01
308081	k__Bacteria;p__Fusobacteria;c__Fusobacteriia;o__Fusobacteriales;f__Fusobacteriaceae;g__Fusobacterium;s__	3.96E-02	5.07E-01	1.49E-01
New.CleanUp.ReferenceOTU89663	k__Bacteria;p__Firmicutes;c__Clostridia;o__Clostridiales	3.98E-02	3.80E-01	7.46E-02
New.CleanUp.ReferenceOTU107869	k__Bacteria;p__Bacteroidetes;c__Bacteroidia;o__Bacteroidales;f__Bacteroidaceae;g__Bacteroides;s__	4.02E-02	2.54E-01	4.48E-02
4437814	k__Bacteria;p__Bacteroidetes;c__Bacteroidia;o__Bacteroidales;f__Bacteroidaceae;g__Bacteroides;s__	4.02E-02	2.54E-01	4.48E-02
New.ReferenceOTU593	k__Bacteria;p__Firmicutes;c__Clostridia;o__Clostridiales;f__Lachnospiraceae;g__;s__	4.03E-02	4.79E-01	1.19E-01
New.CleanUp.ReferenceOTU12239	k__Bacteria;p__Firmicutes;c__Clostridia;o__Clostridiales;f__Ruminococcaceae;g__;s__	4.03E-02	4.23E-02	6.12E-01
New.CleanUp.ReferenceOTU19362	k__Bacteria;p__Bacteroidetes;c__Bacteroidia;o__Bacteroidales;f__Bacteroidaceae;g__Bacteroides;s__	4.03E-02	3.10E-01	5.97E-02
293112	k__Bacteria;p__Bacteroidetes;c__Bacteroidia;o__Bacteroidales;f__;g__;s__	4.06E-02	6.62E-01	1.79E+00
179989	k__Bacteria;p__Firmicutes;c__Clostridia;o__Clostridiales;f__Lachnospiraceae;g__Blautia;s__	4.07E-02	3.80E-01	1.10E+00
125624	k__Bacteria;p__Firmicutes;c__Clostridia;o__Clostridiales;f__Lachnospiraceae	4.08E-02	1.06E+00	2.99E-01
578016	k__Bacteria;p__Bacteroidetes;c__Bacteroidia;o__Bacteroidales;f__Porphyromonadaceae;g__Parabacteroides;s__distasonis	4.09E-02	1.14E+00	1.49E-01
1602805	k__Bacteria;p__Firmicutes;c__Clostridia;o__Clostridiales;f__Lachnospiraceae;g__;s__	4.09E-02	1.83E-01	1.49E-01
New.ReferenceOTU888	k__Bacteria;p__Firmicutes;c__Clostridia;o__Clostridiales;f__Ruminococcaceae;g__;s__	4.14E-02	4.08E-01	8.36E-01
269386	k__Bacteria;p__Cyanobacteria;c__4C0d-2;o__YS2;f__;g__;s__	4.17E-02	1.13E+00	1.18E+00
539735	k__Bacteria;p__Actinobacteria;c__Actinobacteria;o__Actinomycetales;f__Dietziaceae;g__Dietzia;s__	4.18E-02	4.23E-01	5.97E-02
4314126	k__Bacteria;p__Firmicutes;c__Clostridia;o__Clostridiales;f__Ruminococcaceae;g__;s__	4.25E-02	7.04E-02	5.22E-01
588065	k__Bacteria;p__Firmicutes;c__Clostridia;o__Clostridiales;f__Ruminococcaceae;g__;s__	4.27E-02	1.69E-01	6.12E-01
3855886	k__Bacteria;p__Firmicutes;c__Clostridia;o__Clostridiales;f__Lachnospiraceae;g__Roseburia;s__	4.30E-02	5.21E-01	4.18E-01
349971	k__Bacteria;p__Firmicutes;c__Clostridia;o__Clostridiales;f__Lachnospiraceae;g__;s__	4.31E-02	6.62E-01	1.30E+00
672442	k__Bacteria;p__Firmicutes;c__Clostridia;o__Clostridiales;f__Ruminococcaceae;g__Ruminococcus;s__	4.32E-02	1.13E-01	6.42E-01
New.CleanUp.ReferenceOTU101660	k__Bacteria;p__Firmicutes;c__Clostridia;o__Clostridiales;f__Ruminococcaceae;g__;s__	4.33E-02	1.55E-01	5.82E-01
4390319	k__Bacteria;p__Bacteroidetes;c__Bacteroidia;o__Bacteroidales;f__Bacteroidaceae;g__Bacteroides;s__ovatus	4.35E-02	3.38E-01	1.34E-01
New.ReferenceOTU58	k__Bacteria;p__Bacteroidetes;c__Bacteroidia;o__Bacteroidales;f__;g__;s__	4.36E-02	9.52E+00	6.55E+00
4300206	k__Bacteria;p__Firmicutes;c__Bacilli;o__Lactobacillales;f__Lactobacillaceae;g__Lactobacillus;s__	4.40E-02	1.45E+00	6.57E-01

326645	k__Bacteria;p__Firmicutes;c__Clostridia;o__Clostridiales;f__Ruminococcaceae;g__;s__	4.42E-02	2.54E+00	5.52E-01
New.ReferenceOTU371	k__Bacteria;p__Firmicutes;c__Clostridia;o__Clostridiales;f__Clostridiaceae;g__Sarcina;s__	4.42E-02	1.97E-01	5.52E-01
2901965	k__Bacteria;p__Firmicutes;c__Bacilli;o__Lactobacillales;f__Streptococcaceae;g__Streptococcus;s__	4.42E-02	8.45E-01	8.96E-02
New.ReferenceOTU274	k__Bacteria;p__Firmicutes;c__Clostridia;o__Clostridiales;f__Ruminococcaceae;g__;s__	4.42E-02	2.82E-01	9.10E-01
New.CleanUp.ReferenceOTU13024	k__Bacteria;p__Firmicutes;c__Clostridia;o__Clostridiales;f__Clostridiaceae;g__Clostridium;s__	4.42E-02	1.27E-01	0.00E+00
4133460	k__Bacteria;p__Firmicutes;c__Clostridia;o__Clostridiales;f__;g__;s__	4.42E-02	1.55E-01	0.00E+00
325295	k__Bacteria;p__Firmicutes;c__Clostridia;o__Clostridiales;f__Lachnospiraceae;g__Blautia;s__producta	4.42E-02	1.41E-01	0.00E+00
198530	k__Bacteria;p__Bacteroidetes;c__Bacteroidia;o__Bacteroidales;f__Bacteroidaceae;g__Bacteroides	4.42E-02	1.69E-01	0.00E+00
145856	k__Bacteria;p__Firmicutes;c__Clostridia;o__Clostridiales;f__[Mogibacteriaceae];g__;s__	4.42E-02	4.65E-01	0.00E+00
128227	k__Bacteria;p__Firmicutes;c__Bacilli;o__Lactobacillales;f__Lactobacillaceae;g__Lactobacillus;s__	4.42E-02	1.41E-01	0.00E+00
4430499	k__Bacteria;p__Firmicutes;c__Clostridia;o__Clostridiales;f__Ruminococcaceae;g__;s__	4.42E-02	1.41E-01	0.00E+00
New.CleanUp.ReferenceOTU134212	k__Bacteria;p__Firmicutes;c__Clostridia;o__Clostridiales;f__Ruminococcaceae;g__;s__	4.42E-02	1.41E-01	0.00E+00
191779	k__Bacteria;p__Firmicutes;c__Clostridia;o__Clostridiales;f__Lachnospiraceae;g__Blautia;s__	4.42E-02	1.55E-01	0.00E+00
New.CleanUp.ReferenceOTU168741	k__Bacteria;p__Firmicutes;c__Clostridia;o__Clostridiales;f__Lachnospiraceae;g__Blautia;s__	4.42E-02	1.55E-01	0.00E+00
New.CleanUp.ReferenceOTU179296	k__Bacteria;p__Bacteroidetes;c__Bacteroidia;o__Bacteroidales;f__[Barnesiellaceae];g__;s__	4.42E-02	1.55E-01	0.00E+00
135956	k__Bacteria;p__Firmicutes;c__Bacilli;o__Lactobacillales;f__Lactobacillaceae;g__Lactobacillus;s__	4.42E-02	3.10E-01	0.00E+00
344259	k__Bacteria;p__Firmicutes;c__Clostridia;o__Clostridiales;f__Ruminococcaceae;g__;s__	4.42E-02	2.25E-01	0.00E+00
New.CleanUp.ReferenceOTU64518	k__Bacteria;p__Bacteroidetes;c__Bacteroidia;o__Bacteroidales;f__Bacteroidaceae;g__Bacteroides;s__uniformis	4.42E-02	2.25E-01	0.00E+00
New.CleanUp.ReferenceOTU130445	k__Bacteria;p__Spirochaetes;c__Spirochaetes;o__Spirochaetales;f__Spirochaetaceae;g__Treponema;s__	4.42E-02	2.25E-01	0.00E+00
4425280	k__Bacteria;p__Tenericutes;c__Mollicutes;o__RF39;f__;g__;s__	4.42E-02	1.69E-01	0.00E+00
New.CleanUp.ReferenceOTU6101	k__Bacteria;p__Firmicutes;c__Clostridia;o__Clostridiales;f__Ruminococcaceae;g__;s__	4.42E-02	1.69E-01	0.00E+00
New.CleanUp.ReferenceOTU6492	k__Bacteria;p__Firmicutes;c__Clostridia;o__Clostridiales;f__Lachnospiraceae;g__[Ruminococcus];s__gnavus	4.42E-02	2.11E-01	0.00E+00
New.CleanUp.ReferenceOTU40900	k__Bacteria;p__Firmicutes;c__Clostridia;o__Clostridiales;f__Lachnospiraceae;g__;s__	4.42E-02	1.69E-01	0.00E+00
New.CleanUp.ReferenceOTU99751	k__Bacteria;p__Firmicutes;c__Clostridia;o__Clostridiales;f__Ruminococcaceae;g__;s__	4.42E-02	1.69E-01	0.00E+00
New.CleanUp.ReferenceOTU153610	k__Bacteria;p__Firmicutes;c__Clostridia;o__Clostridiales	4.42E-02	1.69E-01	0.00E+00
4415649	k__Bacteria;p__Firmicutes;c__Clostridia;o__Clostridiales;f__Lachnospiraceae	4.42E-02	1.97E-01	0.00E+00
181589	k__Bacteria;p__Firmicutes;c__Bacilli;o__Bacillales	4.42E-02	3.10E-01	0.00E+00
997267	k__Bacteria;p__Firmicutes;c__Clostridia;o__Clostridiales;f__Ruminococcaceae;g__;s__	4.42E-02	1.97E-01	0.00E+00
New.CleanUp.ReferenceOTU124558	k__Bacteria;p__Verrucomicrobia;c__Verrucomicrobiae;o__Verrucomicrobiales;f__Verrucomicrobiaceae;g__Akkermansia;s__muciniphila	4.42E-02	2.54E-01	0.00E+00
New.CleanUp.ReferenceOTU134251	k__Bacteria;p__Firmicutes;c__Clostridia;o__Clostridiales;f__Ruminococcaceae;g__;s__	4.42E-02	1.97E-01	0.00E+00
316728	k__Bacteria;p__Firmicutes;c__Clostridia;o__Clostridiales;f__;g__;s__	4.42E-02	1.69E+00	0.00E+00
New.CleanUp.ReferenceOTU41369	k__Bacteria;p__Firmicutes;c__Bacilli;o__Bacillales;f__Staphylococcaceae;g__Jeotgalicoccus;s__	4.42E-02	3.80E-01	0.00E+00

New.CleanUp.ReferenceOTU167178	k__Bacteria;p__Bacteroidetes;c__Bacteroidia;o__Bacteroidales;f__Bacteroidaceae;g__Bacteroides;s__caccae	4.42E-02	2.11E-01	0.00E+00
New.CleanUp.ReferenceOTU36161	k__Bacteria;p__Firmicutes;c__Clostridia;o__Clostridiales;f__Lachnospiraceae;g__Anaerostipes;s__	4.42E-02	1.97E-01	0.00E+00
539688	k__Bacteria;p__Firmicutes;c__Clostridia;o__Clostridiales;f__;g__;s__	4.42E-02	2.96E-01	0.00E+00
188591	k__Bacteria;p__Firmicutes;c__Clostridia;o__Clostridiales;f__Lachnospiraceae;g__Dorea;s__	4.42E-02	2.39E-01	0.00E+00
New.CleanUp.ReferenceOTU102915	k__Bacteria;p__Firmicutes;c__Clostridia;o__Clostridiales;f__Ruminococcaceae;g__Ruminococcus;s__	4.42E-02	5.49E-01	0.00E+00
181961	k__Bacteria;p__Firmicutes;c__Clostridia;o__Clostridiales;f__Ruminococcaceae;g__Ruminococcus;s__	4.42E-02	7.18E-01	0.00E+00
143423	k__Bacteria;p__Firmicutes;c__Clostridia;o__Clostridiales;f__Ruminococcaceae;g__Oscillospira;s__	4.42E-02	2.25E-01	0.00E+00
187505	k__Bacteria;p__Firmicutes;c__Clostridia;o__Clostridiales;f__Lachnospiraceae	4.42E-02	2.54E-01	0.00E+00
New.CleanUp.ReferenceOTU77217	k__Bacteria;p__Firmicutes;c__Clostridia;o__Clostridiales;f__Lachnospiraceae	4.42E-02	3.80E-01	0.00E+00
New.CleanUp.ReferenceOTU72464	k__Bacteria;p__Bacteroidetes;c__Bacteroidia;o__Bacteroidales;f__;g__;s__	4.42E-02	1.27E-01	4.33E-01
New.ReferenceOTU235	k__Bacteria;p__Firmicutes;c__Clostridia;o__Clostridiales;f__Lachnospiraceae;g__Coprococcus;s__	4.42E-02	3.38E-01	0.00E+00
New.CleanUp.ReferenceOTU133907	k__Bacteria;p__Bacteroidetes;c__Bacteroidia;o__Bacteroidales;f__Bacteroidaceae;g__Bacteroides;s__	4.43E-02	3.38E-01	8.96E-02
New.CleanUp.ReferenceOTU128143	k__Bacteria;p__Bacteroidetes;c__Bacteroidia;o__Bacteroidales;f__[Paraprevotellaceae];g__;s__	4.45E-02	2.11E-01	1.09E+00
4442699	k__Bacteria;p__Firmicutes;c__Clostridia;o__Clostridiales;f__Lachnospiraceae;g__;s__	4.46E-02	1.13E+00	2.69E-01
New.ReferenceOTU545	k__Bacteria;p__Actinobacteria;c__Coriobacteriia;o__Coriobacteriales;f__Coriobacteriaceae;g__Adlercreutzia;s__	4.53E-02	4.37E-01	1.31E+00
New.CleanUp.ReferenceOTU63830	k__Bacteria;p__Bacteroidetes;c__Bacteroidia;o__Bacteroidales;f__;g__;s__	4.53E-02	9.86E-02	6.42E-01
731247	k__Bacteria;p__Spirochaetes;c__Spirochaetes;o__Spirochaetales;f__Spirochaetaceae;g__;s__	4.53E-02	0.00E+00	1.19E-01
337086	k__Bacteria;p__Verrucomicrobia;c__Verruco-5;o__WCHB1-41;f__RFP12;g__;s__	4.53E-02	0.00E+00	1.19E-01
New.CleanUp.ReferenceOTU114817	k__Bacteria;p__Firmicutes;c__Clostridia;o__Clostridiales;f__Dehalobacteriaceae;g__Dehalobacterium;s__	4.53E-02	0.00E+00	1.19E-01
569244	k__Bacteria;p__Tenericutes;c__Mollicutes;o__RF39;f__;g__;s__	4.53E-02	1.83E-01	8.66E-01
805699	k__Bacteria;p__Tenericutes;c__Mollicutes;o__RF39;f__;g__;s__	4.53E-02	0.00E+00	1.49E-01
331181	k__Bacteria;p__Bacteroidetes;c__Bacteroidia;o__Bacteroidales;f__Bacteroidaceae;g__BF311;s__	4.53E-02	0.00E+00	1.34E-01
291591	k__Bacteria;p__Firmicutes;c__Clostridia;o__Clostridiales;f__Lachnospiraceae;g__;s__	4.53E-02	0.00E+00	1.49E-01
New.CleanUp.ReferenceOTU35170	k__Bacteria;p__Firmicutes;c__Clostridia;o__Clostridiales;f__Dehalobacteriaceae;g__Dehalobacterium;s__	4.53E-02	0.00E+00	1.34E-01
New.CleanUp.ReferenceOTU41051	k__Bacteria;p__Firmicutes;c__Clostridia;o__Clostridiales;f__Ruminococcaceae;g__;s__	4.53E-02	0.00E+00	1.34E-01
New.CleanUp.ReferenceOTU165628	k__Bacteria;p__Firmicutes;c__Clostridia;o__Clostridiales;f__Ruminococcaceae;g__;s__	4.53E-02	0.00E+00	1.34E-01
New.CleanUp.ReferenceOTU168260	k__Bacteria;p__Spirochaetes;c__Spirochaetes;o__Spirochaetales;f__Spirochaetaceae;g__Treponema;s__	4.53E-02	0.00E+00	1.34E-01
New.CleanUp.ReferenceOTU169041	k__Bacteria;p__Firmicutes;c__Clostridia;o__Clostridiales;f__Lachnospiraceae	4.53E-02	0.00E+00	1.34E-01
New.CleanUp.ReferenceOTU79987	k__Bacteria;p__Firmicutes;c__Clostridia;o__Clostridiales;f__Clostridiaceae;g__;s__	4.53E-02	4.65E-01	5.97E-02
New.CleanUp.ReferenceOTU130256	k__Bacteria;p__Firmicutes;c__Bacilli;o__Lactobacillales;f__Streptococcaceae;g__Streptococcus;s__	4.53E-02	3.52E-01	5.97E-02
New.CleanUp.ReferenceOTU123128	k__Bacteria;p__Firmicutes;c__Clostridia;o__Clostridiales;f__Ruminococcaceae;g__;s__	4.53E-02	0.00E+00	1.49E-01
4359831	k__Bacteria;p__Firmicutes;c__Clostridia;o__Clostridiales;f__;g__;s__	4.53E-02	0.00E+00	1.64E-01

293417	k__Bacteria;p__Firmicutes;c__Clostridia;o__Clostridiales;f__;g__;s__	4.53E-02	0.00E+00	2.69E-01
355309	k__Bacteria;p__Bacteroidetes;c__Bacteroidia;o__Bacteroidales;f__;g__;s__	4.53E-02	0.00E+00	7.01E-01
319544	k__Bacteria;p__Firmicutes;c__Clostridia;o__Clostridiales;f__;g__;s__	4.53E-02	0.00E+00	2.24E-01
296040	k__Bacteria;p__Firmicutes;c__Clostridia;o__Clostridiales;f__Ruminococcaceae;g__;s__	4.53E-02	0.00E+00	1.64E-01
New.CleanUp.ReferenceOTU29685	k__Bacteria;p__Bacteroidetes;c__Bacteroidia;o__Bacteroidales;f__[Paraprevotellaceae];g__CF231;s__	4.53E-02	0.00E+00	3.13E-01
New.CleanUp.ReferenceOTU84233	k__Bacteria;p__Bacteroidetes;c__Bacteroidia;o__Bacteroidales;f__[Paraprevotellaceae];g__;s__	4.53E-02	0.00E+00	1.64E-01
4481975	k__Bacteria;p__Firmicutes;c__Clostridia;o__Clostridiales;f__Lachnospiraceae;g__;s__	4.53E-02	0.00E+00	1.79E-01
New.CleanUp.ReferenceOTU24933	Unassigned	4.53E-02	0.00E+00	2.09E-01
New.CleanUp.ReferenceOTU142829	k__Bacteria;p__Firmicutes;c__Clostridia;o__Clostridiales;f__Ruminococcaceae;g__;s__	4.53E-02	0.00E+00	1.79E-01
New.ReferenceOTU965	k__Bacteria;p__Firmicutes;c__Clostridia;o__Clostridiales;f__;g__;s__	4.53E-02	0.00E+00	2.69E-01
300457	k__Bacteria;p__Firmicutes;c__Clostridia;o__Clostridiales;f__Ruminococcaceae;g__Oscillospira;s__	4.53E-02	0.00E+00	2.39E-01
New.CleanUp.ReferenceOTU37972	k__Bacteria;p__Firmicutes;c__Clostridia;o__Clostridiales;f__;g__;s__	4.53E-02	0.00E+00	2.69E-01
New.CleanUp.ReferenceOTU143005	k__Bacteria;p__Firmicutes;c__Clostridia;o__Clostridiales;f__[Tissierellaceae];g__Helcococcus;s__	4.53E-02	0.00E+00	6.57E-01
172081	k__Bacteria;p__Firmicutes;c__Clostridia;o__Clostridiales;f__;g__;s__	4.53E-02	0.00E+00	2.84E-01
New.CleanUp.ReferenceOTU103833	k__Bacteria;p__Firmicutes;c__Clostridia;o__Clostridiales;f__;g__;s__	4.53E-02	0.00E+00	2.54E-01
New.CleanUp.ReferenceOTU290	k__Bacteria;p__Firmicutes;c__Clostridia;o__Clostridiales;f__[Mogibacteriaceae];g__;s__	4.53E-02	4.23E-02	2.39E-01
3807411	k__Bacteria;p__Firmicutes;c__Clostridia;o__Clostridiales;f__Ruminococcaceae;g__;s__	4.57E-02	6.62E-01	7.46E-02
4345515	k__Bacteria;p__Firmicutes;c__Clostridia;o__Clostridiales;f__Ruminococcaceae;g__;s__	4.57E-02	8.69E+00	1.25E+01
291202	k__Bacteria;p__Firmicutes;c__Clostridia;o__Clostridiales;f__Ruminococcaceae;g__;s__	4.57E-02	1.02E+01	3.79E+00
New.CleanUp.ReferenceOTU71646	k__Bacteria;p__Firmicutes;c__Clostridia;o__Clostridiales;f__Lachnospiraceae;g__;s__	4.63E-02	3.24E-01	7.61E-01
175836	k__Bacteria;p__Firmicutes;c__Clostridia;o__Clostridiales;f__Ruminococcaceae;g__Ruminococcus;s__	4.65E-02	6.06E-01	1.04E-01
330492	k__Bacteria;p__Firmicutes;c__Clostridia;o__Clostridiales;f__[Mogibacteriaceae];g__;s__	4.69E-02	8.45E-02	3.88E-01
New.CleanUp.ReferenceOTU27705	k__Bacteria;p__Firmicutes;c__Clostridia;o__Clostridiales;f__Ruminococcaceae;g__;s__	4.78E-02	2.82E-01	4.93E-01
New.CleanUp.ReferenceOTU34463	k__Bacteria;p__Bacteroidetes;c__Bacteroidia;o__Bacteroidales;f__Porphyromonadaceae;g__Paludibacter;s__	4.80E-02	8.45E-02	5.67E-01
43950	k__Bacteria;p__Firmicutes;c__Clostridia;o__Clostridiales;f__Ruminococcaceae;g__Oscillospira;s__	4.85E-02	4.37E-01	1.79E-01
336149	k__Bacteria;p__Firmicutes;c__Clostridia;o__Clostridiales;f__Lachnospiraceae;g__;s__	4.85E-02	1.69E-01	1.07E+00
New.ReferenceOTU909	k__Bacteria;p__Firmicutes;c__Clostridia;o__Clostridiales;f__Ruminococcaceae;g__Oscillospira;s__	4.86E-02	1.03E+00	2.12E+00
355909	k__Bacteria;p__Firmicutes;c__Clostridia;o__Clostridiales;f__Ruminococcaceae;g__;s__	4.86E-02	1.13E-01	5.22E-01
346455	k__Bacteria;p__Firmicutes;c__Clostridia;o__Clostridiales;f__Lachnospiraceae;g__;s__	4.89E-02	1.70E+00	1.19E-01
New.ReferenceOTU477	k__Bacteria;p__Firmicutes;c__Clostridia;o__Clostridiales;f__Ruminococcaceae;g__;s__	4.90E-02	2.82E-02	7.16E-01
518040	k__Bacteria;p__Firmicutes;c__Clostridia;o__Clostridiales;f__;g__;s__	4.91E-02	5.35E-01	7.46E-02
292977	k__Bacteria;p__Verrucomicrobia;c__Verruco-5;o__WCHB1-41;f__RFP12;g__;s__	4.98E-02	2.96E-01	7.46E-01
