# Supplementary material for: Composition and Diversity of the Fecal Microbiome and Inferred Fecal Metagenome Does Not Predict Subsequent Pneumonia Caused by Rhodococcus equi in Foals
Source: PLoS One. 2015 Aug 25;10(8):e0136586. doi: 10.1371/journal.pone.0136586 (PMC4549325; doi:10.1371/journal.pone.0136586)
Supplement: S3 Table — The first column list increased KEGG orthologs and the secondond column list decreased KEGG orthologs. (PDF) [file pone.0136586.s003.pdf]

KO Decreased >2 fold	KO Increased >2 fold
K11695	K07504
K02545	K00383
K12553	K01563
K10850	K00569
K12552	K03718
K04478	K09125
K01401	K09939
K11694	K03820
K08255	K00333
K13714	K09136
K12551	K06955
K11442	K07068
K09814	K03390
K09813	K01322
K08170	K09007
K06122	K07157
K14333	K01067
K10227	K00156
K01905	K00946
K07535	K09474
K11635	K02191
K01713	K00839
K05988	K01960
K11914	K13953
K11916	K00772
K09982	K10844
K10677	K09459
K02753	K05788
K10908	K03918
K00395	K04127
K11915	K07156
K13892	K03638
K01103	K02067
K12661	K05776
K13637	K07459
K04784	K07245
K00534	K03709
K13651	K12524
K09991	K03215

K10561	K01485
K07683	K00903
K06165	K10985
K10851	K03787
K00094	K00428
K06120	K00441
K01152	K04568
K06163	K06015
K05780	K02427
K06164	K03113
K05781	K03795
K05338	K02763
K05339	K07081
K10202	K03775
K10795	K02303
K10544	K07008
K05304	K09005
K03851	K09797
K10555	K09798
K00329	K00038
K10559	K07739
K06609	K00510
K07482	K05838
K03852	K02639
K10560	K07051
K05905	K03179
K10120	K05884
K06951	K01434
K07469	K07494
K03191	K09011
K03198	K07123
K00689	K06869
K13919	K06992
K08314	K02666
K10711	K09122
K00076	K00641
K10122	K14054
K05305	K12141
K13002	K13015
K10121	K07442
K00355	K09126

K09384	K03592
K13020	K00245
K05352	K13819
K06330	K05575
K07396	K00230
K07248	K12140
K03309	K05966
K12583	K13599
K06418	K02060
K03332	K05979
K00863	K06990
K07705	K06203
K02102	K08481
K05297	K06936
K10556	K05873
K02750	K07151
K08717	K07094
K07257	K07446
K09960	K03568
K00918	K03658
K14591	K11646
K01002	K02064
K08965	K14187
K02786	K06988
K01535	K06048
K10545	K07013
K01210	K02478
K02771	K09141
K01501	K06176
K00803	K03623
K10796	K00729
K09703	K07503
K10793	K12234
K03779	K04566
K08676	K09979
K02509	K15034
K11534	K07161
K07709	K00556
K01220	K02584
K00883	K02063
K15036	K00584

K03780	K11921
K01514	K09809
K07803	K02626
K05822	K01959
K11214	K09120
K05341	K04483
K01629	K14120
K01305	K13820
K10201	K14117
K03444	K03720
K01797	K03433
K06605	K14126
K12240	K03051
K01636	K00809
K06928	K06982
K09961	K07096
K02840	K04794
K13049	K10977
K10794	K02922
K00374	K02889
K01750	K02201
K13891	K09726
K06995	K06944
K03476	K08315
K12527	K14941
K01777	K13990
K05306	K03462
K13890	K06943
K00112	K03432
K06952	K07580
K02574	K00188
K08169	K14112
K02547	K14654
K09696	K03124
K13889	K03265
K01573	K04796
K08358	K02936
K09988	K07064
K08357	K12589
K09697	K02966
K01818	K09124

K01212	K06862
K12700	K03041
K02468	K14118
K07704	K02323
K09706	K14111
K02439	K02322
K00691	K04798
K00881	K02869
K01804	K03627
K00373	K02883
K03457	K07744
K00194	K03047
K06212	K03058
K06310	K07060
K01062	K07728
K06909	K14124
K01036	K03238
K01071	K01728
K12941	K02929
K03299	K13525
K09704	K02685
K09001	K09717
K12136	K03232
K01057	K02991
K07162	K07991
K07219	K09724
K07694	K14128
K02777	K07569
K08369	K03050
K00060	K02984
K06610	K10747
K03931	K07108
K07467	K07499
K02075	K02921
K06162	K14097
K07707	K07558
K13954	K09735
K00055	K07055
K01813	K14127
K00197	K01592
K01139	K02908

K03833	K06914
K01487	K02319
K14977	K11212
K00135	K06984
K02103	K03105
K03480	K09727
K05845	K00201
K01608	K03042
K02074	K14122
K14138	K00320
K13922	K00443
K12992	K00400
K00302	K02974
K00034	K07583
K13678	K00196
K11104	K03231
K00633	K07083
K07160	K11176
K00675	K03552
K03336	K06865
K02083	K14653
K00086	K09142
K01051	K03726
	K03237
	K14116
	K06965
	K05715
	K14100
	K07557
	K07392
	K14656
	K09721
	K00399
	K09739
	K07581
	K07388
	K07178
	K02866
	K11600
	K09713
	K00581

	K02927
	K03421
	K03243
	K13942
	K03422
	K03167
	K06174
	K02924
	K07579
	K13812
	K00555
	K06875
	K14115
	K04802
	K02875
	K01622
	K03234
	K00578
	K02978
	K01499
	K07142
	K03236
	K02912
	K02930
	K04800
	K03264
	K06863
	K07158
	K09728
	K14102
	K06874
	K09119
	K02877
	K00580
	K08979
	K00672
	K14119
	K00402
	K03056
	K07463
	K07103

	K07398
	K04795
	K03263
	K06981
	K02977
	K02683
	K06933
	K07254
	K00204
	K14123
	K04797
	K14101
	K01001
	K07575
	K03053
	K03538
	K14094
	K08096
	K09140
	K07561
	K03537
	K06913
	K09723
	K00579
	K01170
	K02962
	K00200
	K02910
	K03679
	K07730
	K07174
	K08971
	K00202
	K03120
	K07721
	K07072
	K04801
	K10726
	K07572
	K03136
	K03059

	K09741
	K14121
	K03626
	K00186
	K14099
	K11781
	K00021
	K07244
	K03166
	K07466
	K02944
	K00125
	K00442
	K14093
	K00440
	K11260
	K00401
	K00150
	K07573
	K03540
	K14095
	K09730
	K14110
	K03539
	K07134
	K11780
	K00577
	K07144
	K03055
	K00586
	K07562
	K03057
	K02896
	K00583
	K14107
	K03242
	K14125
	K00205
	K07325
	K04799
	K04093

	K09720
	K09722
	K03725
	K07135
	K06961
	K09738
	K09154
	K05716
	K14104
	K07342
	K07159
	K07065
	K14098
	K10896
	K00187
	K02987
	K00203
	K04484
	K09733
	K09482
	K14113
	K07732
	K00319
	K02995
	K07041
	K10725
	K03044
	K02885
	K06983
	K03420
	K14096
	K02915
	K14109
	K02979
	K06932
	K03330
	K03045
	K04035
	K14092
	K14114
	K07585

	K03049
	K07337
	K14108
	K06269
	K00582
	K14106
	K14105
	K03932
	K14103
	K09003
	K08226
	K07049
	K01150
	K13498
	K01906
	K05281
	K06868
	K06946
	K07163
	K09942
	K00309
	K01593
	K01054
	K07333
	K13829
	K01216
	K09859
	K09144
	K09127
	K09714
	K07283
	K09150
	K11443
	K09805
	K02506
	K08139
	K09746
	K13581
	K13001
	K05555
	K07302

	K09950
	K07303
	K01249
	K06986
	K07330
	K12988
	K13587
	K12449
	K09715
	K10843
	K01437
	K10232
	K01655
	K13665
	K11045
	K03715
	K10233
	K08081
	K10234
	K09743
	K01253
	K05588
	K01506
	K02383
	K08988
	K05351
	K05824
	K14257
	K05899
	K00988
	K00288
	K13421
	K14164
	K00011
	K08980
	K06027
	K00178
